# Whole-genome resequencing of common bean elite breeding lines

**DOI:** 10.1038/s41598-023-39399-6

**Published:** 2023-08-05

**Authors:** Isabela Pavanelli de Souza, Beatriz Rosa de Azevedo, Alexandre Siqueira Guedes Coelho, Thiago Lívio Pessoa Oliveira de Souza, Paula Arielle Mendes Ribeiro Valdisser, Lucas Matias Gomes-Messias, Breno Osvaldo Funicheli, Claudio Brondani, Rosana Pereira Vianello

**Affiliations:** 1 Genetics and Plant Breeding, Brazilian Agricultural Research Corporation, Santo Antônio de Goiás, GO Brazil; 2 Biotechnology, Scientific Initiation Scholarship, Brazilian Agricultural Research Corporation, Santo Antônio de Goiás, GO Brazil; 3 Plant Breeding, Universidade Federal de Goiás, Goiânia, GO Brazil; 4 Plant Breeding, Brazilian Agricultural Research Corporation, Santo Antônio de Goiás, GO Brazil; 5 Genetics and Molecular Biology, Brazilian Agricultural Research Corporation, Santo Antônio de Goiás, GO Brazil; 6 Genetics and Plant Breeding, Universidade Federal de Goiás, Goiânia, GO Brazil; 7 Computer Science, Universidade Federal de São Carlos, São Carlos, SP Brazil; 8 Molecular Biology, Brazilian Agricultural Research Corporation, Santo Antônio de Goiás, GO Brazil

**Keywords:** Agricultural genetics, Plant breeding, Next-generation sequencing

## Abstract

The expansion of bean genome technologies has prompted new perspectives on generating resources and knowledge essential to research and implementing biotechnological tools for the practical operations of plant breeding programs. This study aimed to resequence the entire genome (whole genome sequencing—WGS) of 40 bean genotypes selected based on their significance in breeding programs worldwide, with the objective of generating an extensive database for the identification of single nucleotide polymorphisms (SNPs). Over 6 million SNPs were identified, distributed across the 11 bean chromosomes. After quality variant filtering, 420,509 high-quality SNPs were established, with an average of 38,228 SNPs per chromosome. These variants were categorized based on their predicted effects, revealing that the majority exerted a modifier impact on non-coding genome regions (94.68%). Notably, a significant proportion of SNPs occurred in intergenic regions (62.89%) and at least one SNP was identified in 58.63% of the genes annotated in the bean genome. Of particular interest, 7841 SNPs were identified in 85% of the putative plant disease defense-related genes, presenting a valuable resource for crop breeding efforts. These findings provide a foundation for the development of innovative and broadly applicable technologies for the routine selection of superior genotypes in global bean improvement and germplasm characterization programs.

## Introduction

Among pulses, the common bean stands out as one of the most significant food crops globally, with a profound socioeconomic impact^1,2^. Its grains are gluten-free and serve as a valuable nutritional source, abundant in proteins, minerals, vitamins, and bioactive compounds^3^. Additionally, they are high in fiber, low in fat, and rich in slow-digesting carbohydrates, which are associated with a considerably reduced risk of heart disease, obesity, and diabetes^4,5^. Despite its numerous health benefits, bean cultivation presents several challenges, including enhancing yield in the presence of biotic and abiotic stresses, as well as improving grain quality and nutritional value^6,7^. To overcome these challenges, plant breeders have been empowered by the knowledge and understanding of the common bean's genomic resources to conduct more targeted and specific studies that align with global requirements^8^.

The generation of a substantial amount of genomic information has led to a significant transformation in the overall field of plant breeding. The availability of the sequence of the entire genome allows researchers to understand and exploit the genetic variations and their control over complex traits, enabling plant breeders to conduct more targeted and specific studies aligned with the global demands. Among the approaches available for generating a vast amount of genomic information, whole-genome sequencing (WGS) provides an opportunity to analyze and identify genetic variations at the base level throughout the entire genome, such as single nucleotide polymorphisms (SNPs), insertions, deletions, and structural variations^9,10^. For common bean, WGS projects aim to uncover novel insights into evolution and diversity, contributing to the generation of valuable genomic resources that accelerate common bean improvement and enhance its efficiency^11,12^.

By integrating genomic knowledge, such as genomic structure, function, diversity, with quantitative genetics, a solid foundation has been established for detailed dissection of the genomic architecture of genetic variation^13–15^. In addition, this integration has provided valuable insights into the evolutionary and domestication history of plants, as well as the genetic mechanisms of adaptations and selective breeding that have contributed to the environment’s adaptation^16^. This has empowered plant breeders to propose and implement new breeding approaches, such as genome-wide selection (GS) and marker-assisted selection (MAS) specifically aimed at selecting traits of high economic importance^17–19^. The availability of the new genomic tools has increasingly provided improvements in terms of speed, efficiency, accuracy, cost-effectiveness of plant breeding processes opening new ways to improve agronomic traits^20^, addressing the demands of the productive sector^21–26^.

In this study, we performed a comprehensive analysis of genomic variations using WGS in 40 important breeding lines/cultivars from the Middle American and Andean gene pools, developed by Brazilian and international breeding programs. The objective was to assist in the genetic studies of important agronomic traits, addressing the challenges faced in common bean cultivation.

## Results

### Sequencing and quality filters

The WGS of the 40 common bean lines (Supplementary Table S1) generated 7.4 billion paired end read fragments, the equivalent to a raw data set of 1.18 Terabases (Tb), ranging from 5.14 billion (Rosinha G2) to 210.47 billion base pairs (BRS Pérola) (Supplementary Table S2). High-quality data was obtained, with a yield of 97–99% of reads ≥ 151 base pair (pb) after filtering. On average, 98.7% of reads were successfully mapped to the genome (Table 1) resulting in an estimated coverage of 1905 times (Supplementary Table S2), ranging from 8.78 times to 358.56 times, based on the 587 Megabase (Mb) genome of the Andean common bean variety G19833^27^ (Supplementary Table S2).

**Table 1:** Mean coverage, depth, and number of SNPs in each of the 11 common bean chromosomes in the set of 40 accessions.

Chrom	Coverage	Depth	MapQ	Number of SNPs			
				Raw	Repetitive elements	MAF > 0.05 + biallelic SNPs	Hard filtering
1	98.87	208.75	40.5	618,493	369,261	318,929	19,181
2	99.33	257.32	41.6	669,465	436,456	381,685	52,200
3	98.93	233.18	40.4	673,399	436,398	380,622	27,832
4	98.42	205.56	39.0	623,532	368,200	317,348	19,714
5	98.19	192.36	38.9	494,332	271,271	231,642	32,437
6	99.04	187.01	45.4	421,988	276,117	243,029	33,211
7	99.38	197.53	47.4	516,827	344,362	299,235	40,972
8	98.70	199.34	38.4	815,298	485,252	395,452	70,650
9	99.41	287.15	40.3	502,968	348,814	297,178	32,515
10	98.16	242.96	35.0	567,133	319,400	277,149	40,491
11	98.11	208.52	36.2	692,832	415,767	360,490	51,306
Total	98.78	219.97	40.28	6,596,267	4,071,298	3,502,759	420,509

### Variant detection and quality filtering

**Figure 1:**
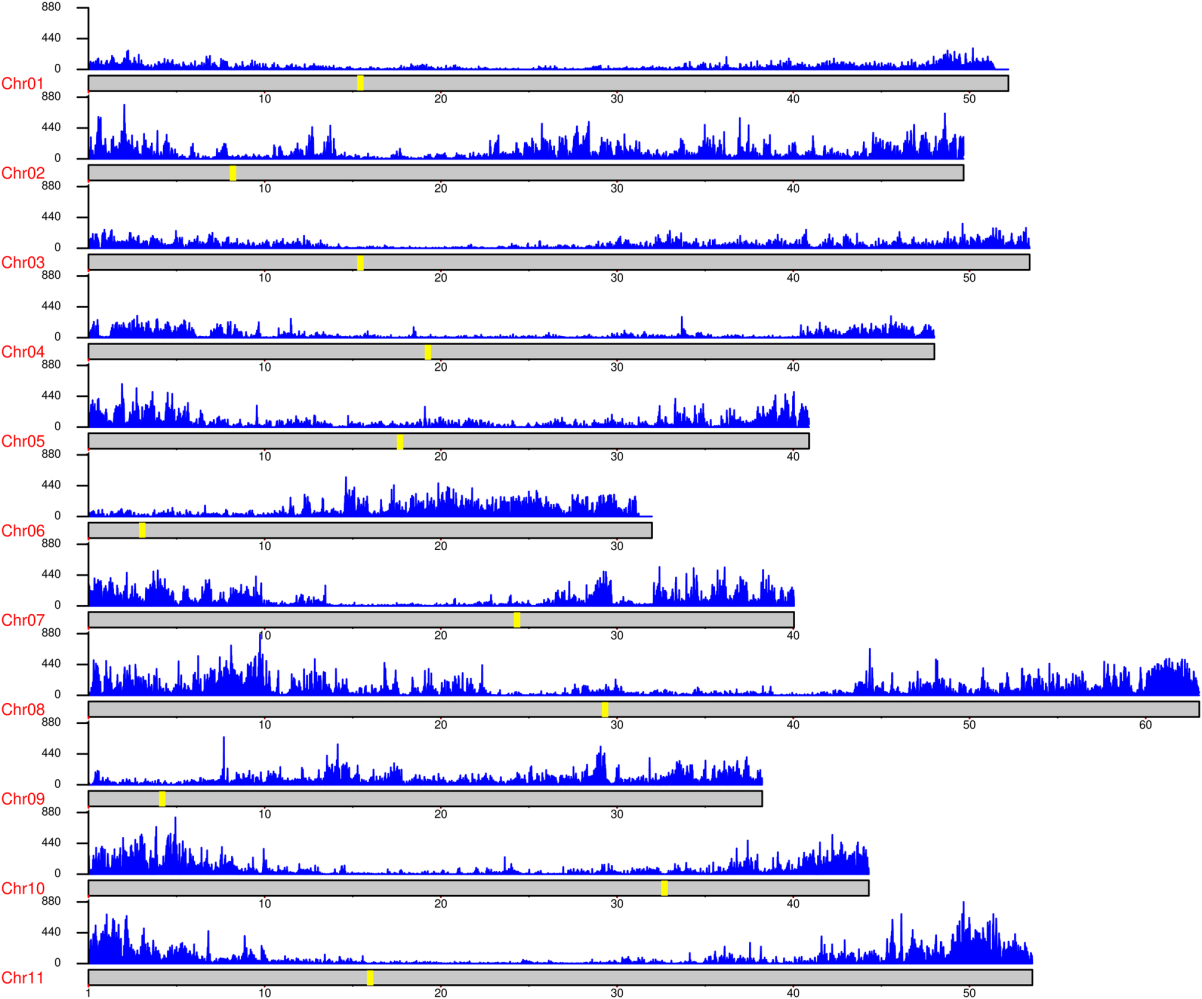
Distribution of SNP density along common bean chromosomes in a 10 Mb sliding window. Yellow bars represent centromeric regions. Figure produced in R v.4.1.1.

More than 6.5 million SNPs were identified in the dataset. After filtering out SNPs in repetitive regions and applying quality metrics, 420,509 (6.37%) with a minor allele frequency (MAF) of ≥ 0.5 were maintained (Table 1). The average number of SNPs per chromosome was 38,228, ranging from 19,181 on chromosome 1 to 70,650 on chromosome 8. The density of SNPs was lower in the centromeric regions (Fig. 1). There was a positive correlation between the physical size of the chromosomes and number of raw SNPs identified on each chromosome (r = 0.982; p-value = 2.39 × 10^–9^). The estimated transition/transversion ratio (Ts/Tv) was 1.29.

**Figure 2:**
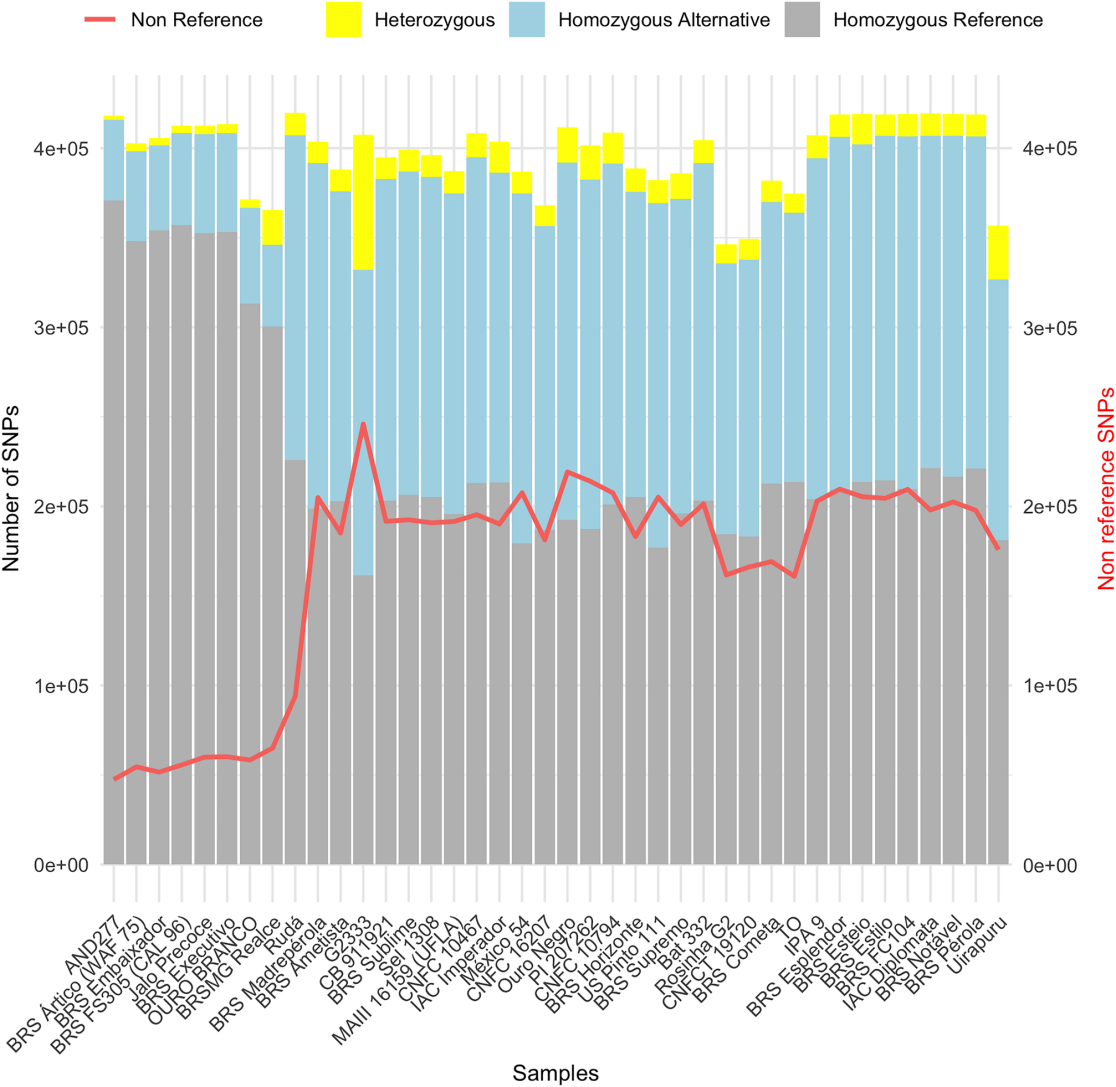
Number of SNPs genotyped in each of the 40 common bean accession from a dataset of filtered SNPs. Genotype calls are discriminated as homozygous for an alternative (nonreference) allele (blue), heterozygous (yellow), and homozygous for the reference allele (gray). The percentage of nonreference genotype calls is displayed as a red line. Figure produced in R v.4.1.1.

The total number of SNPs identified in the 40 genotypes ranged from 346,232 and 419,860 (Supplementary Table S3), with an average distribution of approximately one SNP every 1.4 Kilobases (kb) along the chromosomes (Supplementary Table S4). Homozygous SNPs were more prevalent (96.51%) compared to the heterozygotes SNPs (3.49%), Most variants were identical to the reference genome (G19833) (57.7%), while the remaining variants represented alternative alleles (42.3%), as shown in supplementary table S3 and Fig. 2. Among the Andean group, the average percentage of *non-reference* SNPs was 14.21%, while it was 49.24% for Middle American accessions (Supplementary Table S3).

Regarding the distribution of SNP in the genome, 76.56% (321,925) were detected in intergenic regions, while 23.44% (98,584) were found in gene regions (Supplementary Table S5). Out of the 27,012 genes annotated in the common bean genome, SNPs were identified in 16,413 genes (Table 2), and there was a positive correlation between the number of genes and the number of genes with SNPs sampled per chromosome (r = 0.763; p-value = 4.09 × 10^–3^). Among the SNPs identified in genes, the majority were in introns (58.11%), followed by exons (30.99%), 3′ Untranslated region (UTR) (6.17%), and 5′ UTR (4.73%) regions (Supplementary Table S5).

**Table 2:** Number of SNPs in gene regions in each of the 11 common bean chromosomes in the set of 40 accessions.

Chromosome	Total number of SNPs	Number of genes^a^	Tagged genes
1	3800	2678	1230
2	12,116	3338	2124
3	5514	3023	1447
4	3838	1829	894
5	7725	1759	1191
6	8873	2240	1567
7	10,896	2672	1718
8	16,043	2950	2187
9	6438	2692	1556
10	9618	1672	1219
11	13,723	2159	1431
Total	98,584	27,012	16,564

^a^Number of common bean genes based on the information retrieved at: https://phytozome-next.jgi.doe.gov/info/Pvulgaris_v2_1.

The predicted effects of SNP were categorized based on the variants as follows: modifier (94.68%), moderate (2.69%), low (2.59%) and high (0.05%) (Table 3). Most SNP effects were observed in intergenic regions (62.89%), followed by 15.25% in introns. Additionally, a significant number of effects were detected in the *upstream* and *downstream* regions of genes (up and down 5 kb), accounting for a total of 14.05% of the effects. Furthermore, 2.48% of the effects were in the 5′ and 3′ UTR regions of genes, indicating potential in regulatory regions (Table 3).

**Table 3:** Number and classification of SNP effects predicted for the total set of SNPs for the 40 common bean accessions according to Sequence ontology classification.

Effect type	Number	Percentage	Impact
Intergenic variant	64,482	62.896	MODIFIER
Intron variant	64,122	15.249	MODIFIER
Upstream transcript variant	47,094	11.199	MODIFIER
Downstream transcript variant	12,001	2.854	MODIFIER
Synonymous variant	10,668	2.537	LOW
Missense variant	9175	2.182	MODERATE
3 prime UTR variant	5962	1.418	MODIFIER
5 prime UTR variant	4457	1.060	MODIFIER
Splice region variant	2138	0.508	MODERATE
Exonic splice region variant	206	0.049	LOW
Stop gained	122	0.029	HIGH
Splice acceptor variant	35	0.008	HIGH
Splice donor variant	28	0.007	HIGH
Stop lost	15	0.004	HIGH
Start lost	4	0.001	HIGH

MODIFIER: with impact on no-coding regions, MODERATE: non-synonymous substitution; LOW: synonymous coding/start/stop; HIGH: non-synonymous affecting splice-sites, stop and start codons.

### Intergene pool genetic differentiation

**Figure 3:**
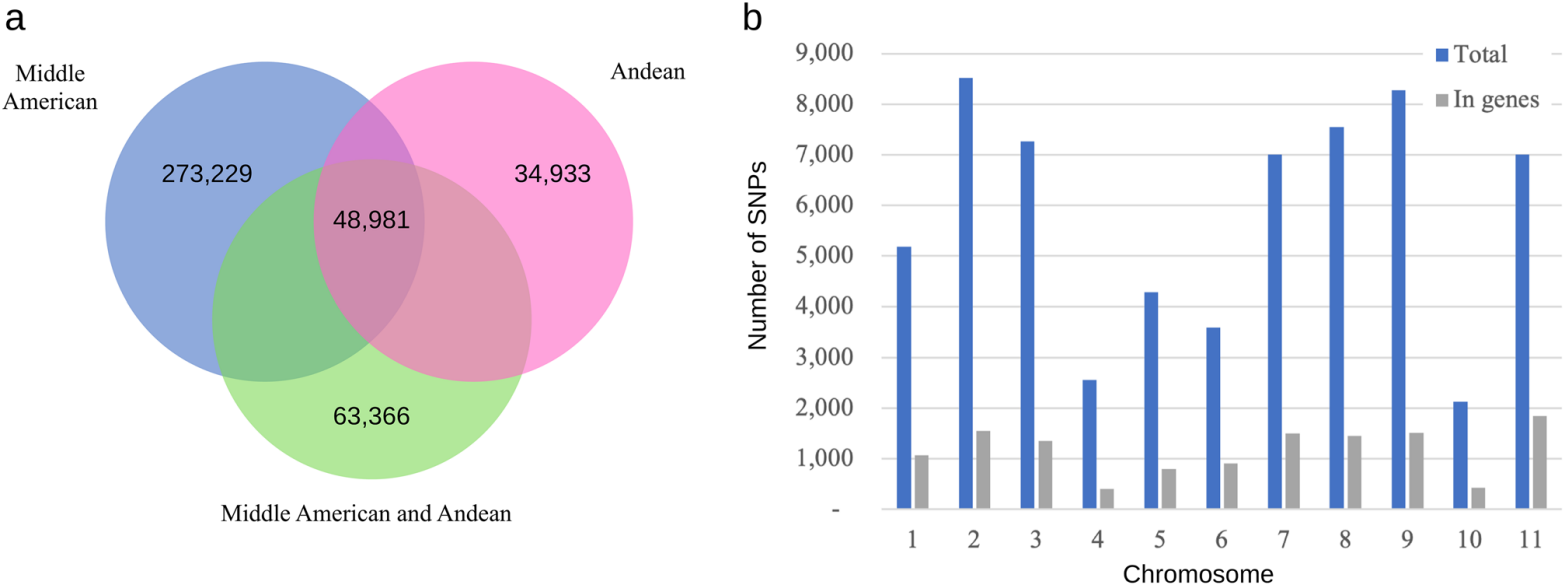
Genetic differentiation between Andean and Middle American gene pools. (**a**) Venn diagram of the set of SNPs from the Andean and Middle American groups produced in JVENN (http://jvenn.toulouse.inra.fr). (**b**) Total number of differentiating SNPs on each of the 11 common bean chromosome and number of differentiating SNPs located within genes produced in R v.4.1.1.

The Middle American and Andean gene pools shared 48,981 SNPs, while 273,229 SNPs were exclusive to the Middle American and 34,933 to the Andean pools (Fig. 3a). The estimated fixation index (F_st_) based on common SNPs (48,981) was 0.27. For the SNPs exclusive to the Middle American germplasm (273,229) and Andean germplasms (34,933) the F_st_ values were 0.31 and 0.85, respectively. The average F_st_ for the total set of SNPs (420,509) was 0.45, with 63,366 differentiating the two groups and resulting in an average F_st_ of 0.97. Chromosome 2 exhibited the highest number of differentiating SNPs (8520), followed by chromosomes 9 (8274) and 8 (7548) (Fig. 3b).

**Figure 4:**
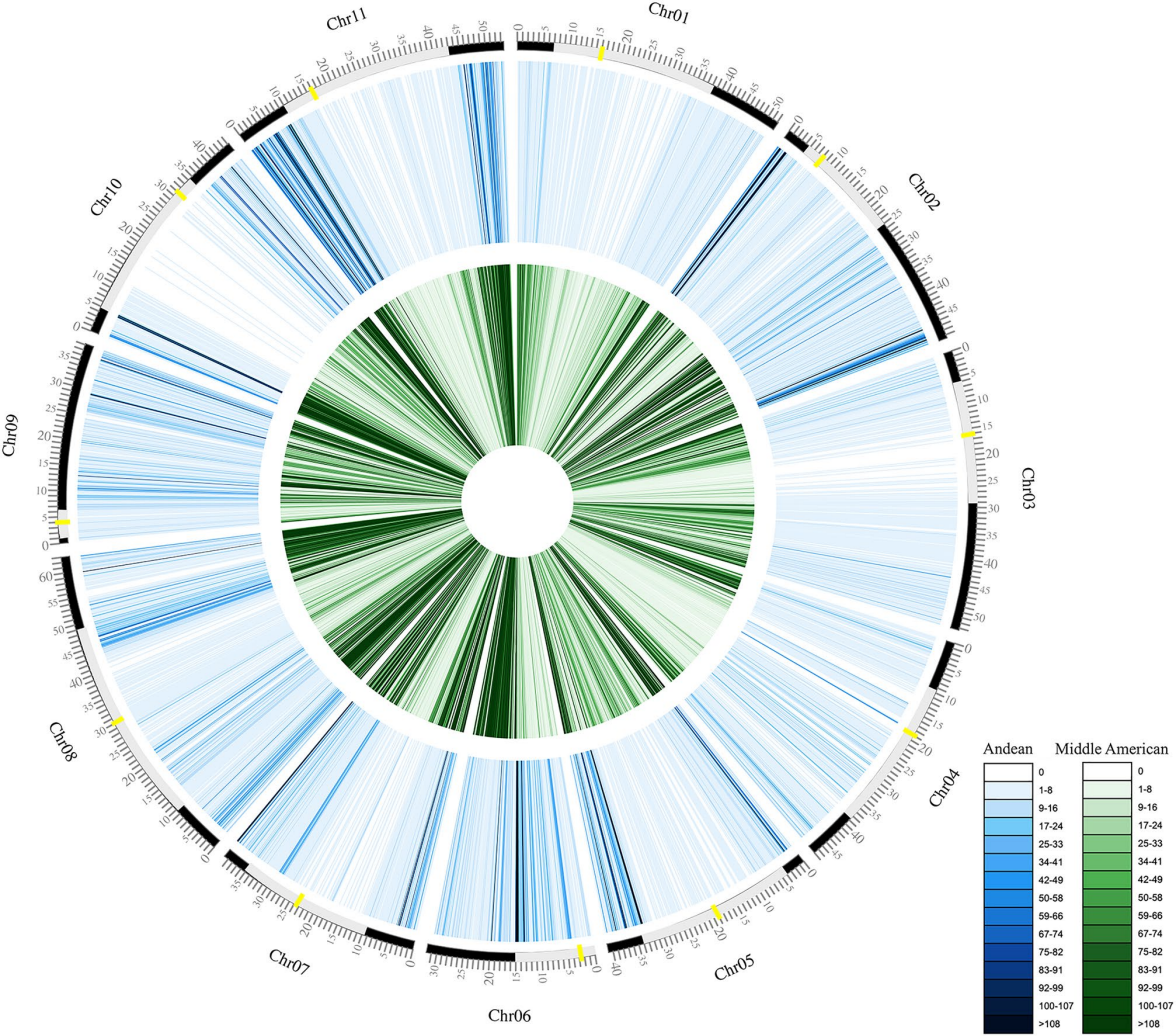
Density of exclusive SNPs detected for each of the 11 chromosome of Andean and Middle American accessions represented by the two rings. Yellow bars represent centromeric regions, and gray bars pericentromeric regions. Each vertical line represents bins of 100 kb. The colors scale is represented on the figure legend. Figure produced in CircosVCF (available at: https://legolas.ariel.ac.il/~tools/CircosVCF/).

**Figure 5:**
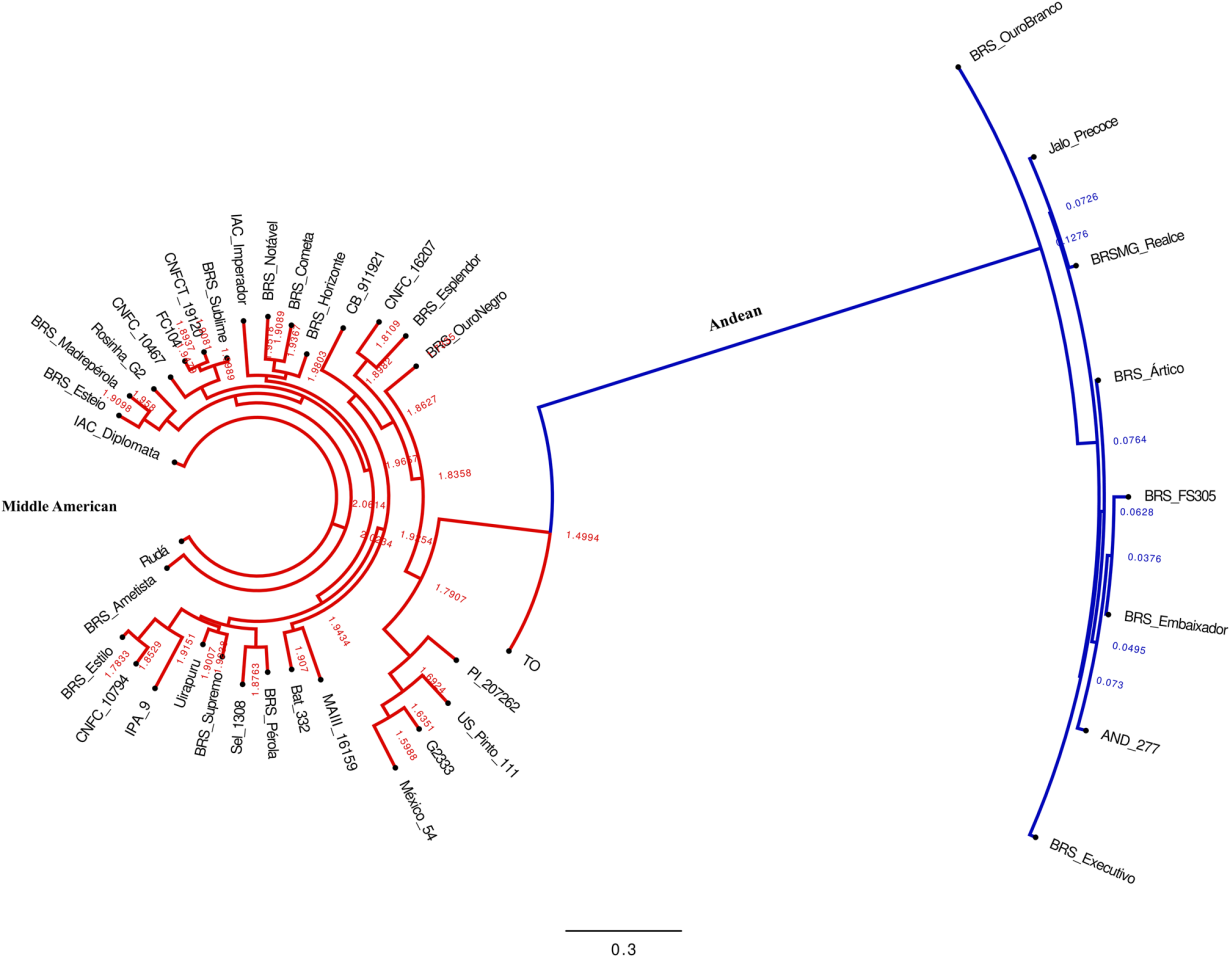
Phylogenetic tree showing the genetic relatedness among 40 common bean accessions based on 30,503 genome-wide high-quality SNP markers. The different line colors identify the accessions according to the gene pool background. Red = Middle American, Blue = Andean. Figure produced in FigTree v1.4.4.

A total of 20.25% of the differentiating SNPs were located within the genes (Fig. 3b). The distribution of SNPs across the chromosomes was observed in both gene pools (Fig. 4; Supplementary Table S6). The average diversity (H_E_) value for the entire sample set was estimated to be 0.36, with 0.29 for the Andean and 0.28 for the Middle American gene pools. The Middle American gene pool exhibited higher nucleotide diversity (π = 0.38; n = 32) compared to its Andean counterpart (π = 0.28; n = 8). Tajima’s D values were estimated as D = 2.46 for the Middle American and D = 0.93 for the Andean germplasms. Phylogenetic analysis based on a set of 30,503 SNPs with a resolution of one bin per 10 kb, revealed a clear division between the Andean and Middle American gene pools, consistent with the well-established classification in the common bean (Fig. 5).

### Introgression analysis

**Figure 6:**
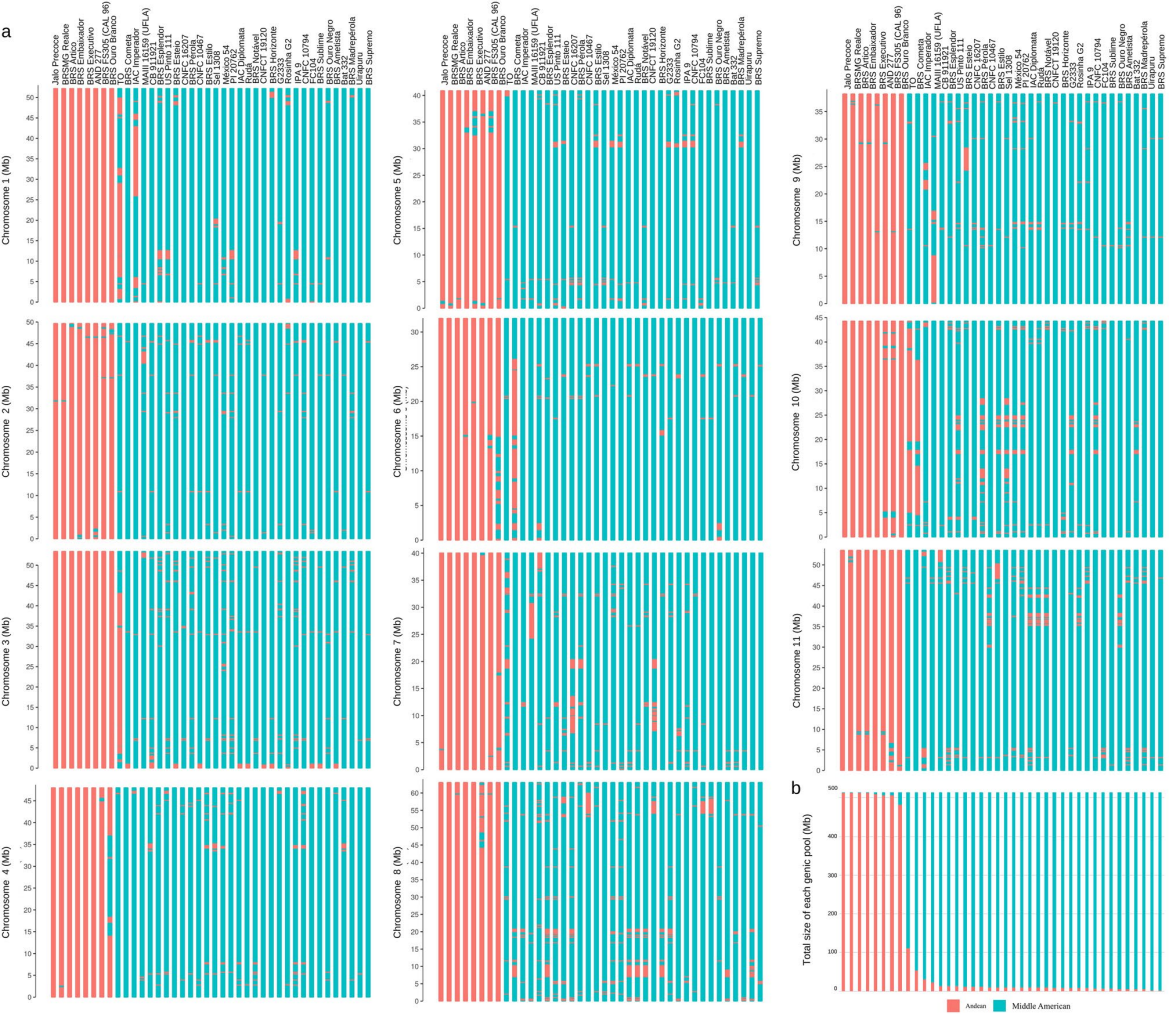
Inter-gene pool introgression in 40 common bean accessions. (**a**) Representation of inter–gene pool introgressions between 8 Andean and 32 Middle American genotypes. The background Andean (salmon), and Middle American haplotypes (turquoise) are represented along the 11 chromosomes. (**b**) Total size of each gene pool. Figures produced in R v.4.1.1.

The analysis of introgression revealed 1497 possible introgression events, with 1387 events corresponding to Andean introgression in 32 cultivars with a Middle American background, and 110 Middle American events in 8 Andean-background cultivars. Among these events, five extended over more than 12 Mb, and 99 extended over more than 1 Mb (Fig. 6, Supplementary Table S7). In general, accessions with an Andean background exhibited a lower number of introgression events, ranging from 0.1% (Jalo Precoce) to 6.16% (Ouro Branco) of the genome. Notably, the Ouro Branco cultivar showed a higher level of introgression compared to others genotypes in the Andean group, which may explain its separation of the phylogenetic tree (Fig. 5). Regarding the varieties with a Middle American background, introgressions ranged from 0.25% (BRS Supremo) to 21.63% (TO).

### R-genes

In total of 660 transcripts were annotated as putative R-genes, out of which 504 were unique genes. Most of these genes were annotated as containing the conserved NBS domain (91.07%), with a predominance of NB-ARC, TIR, CC, kinase, LRR, Serine/threonine-LRR and Kinase-LRR domains. Additionally, 45 genes were classified as putative disease resistance functions, including zinc finger transcription factors and dirigent-like proteins (Supplementary Table S8).

**Figure 7:**
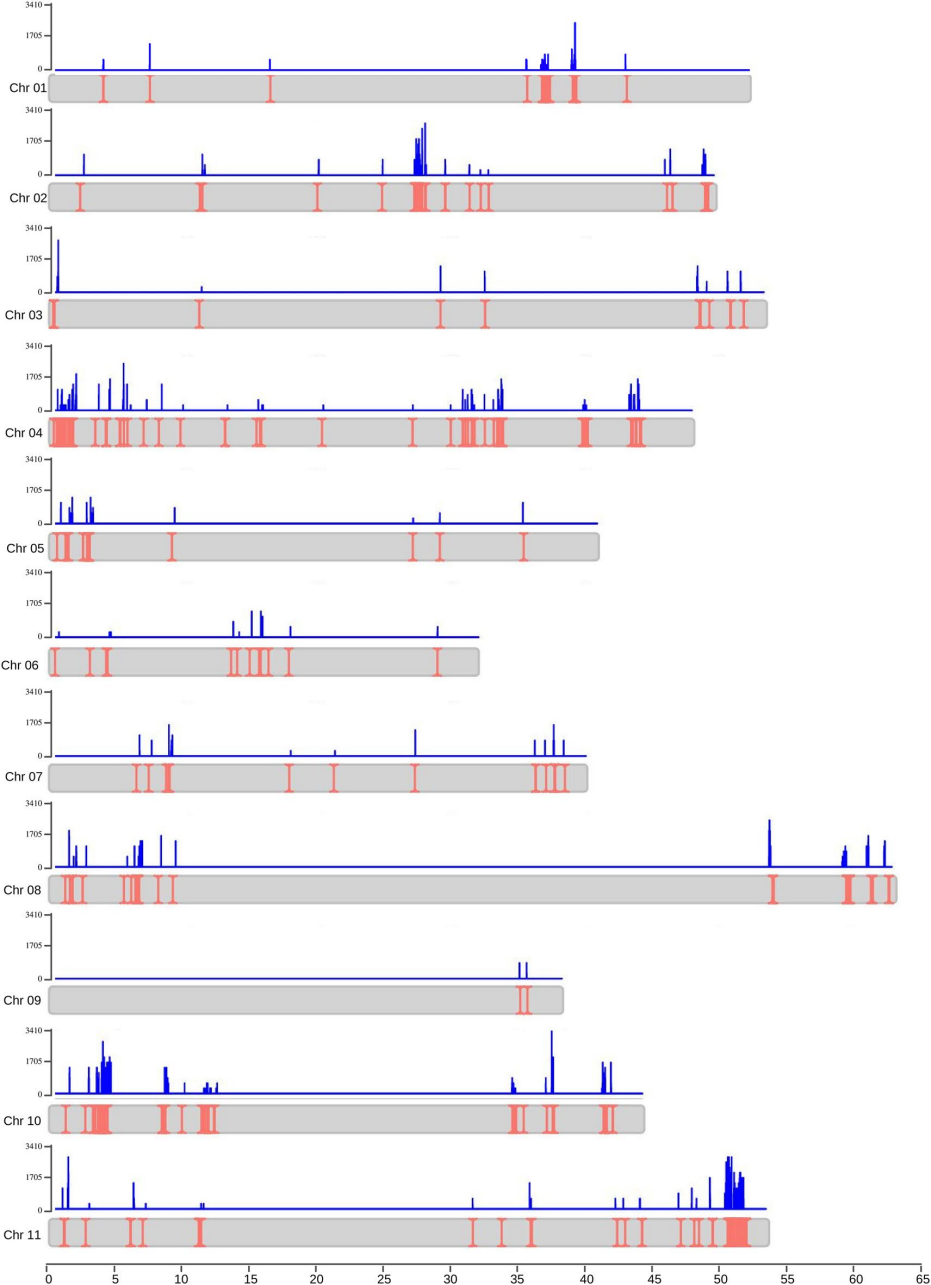
SNPs identified as associated with putative disease genes. Position of disease resistance genes along the common bean chromosomes are depicted by salmon vertical lines and SNP density by blue vertical lines in a 100pb sliding window. Figure produced in R v.4.1.1.

The distribution of R-genes was non-uniform across the genome, with chromosome 4 containing a higher number of genes (115 genes), while chromosome 9 had only two annotated genes, as shown in Fig. 7. Within the R-genes sequences, a total of 7841 SNPs (1.86% of the total number of SNPs) was identified (Supplementary Table S9) with at least one SNP detected in 426 genes (85.03%)*.*

The SNPs identified in disease resistance genes were classified according to their predicted effects. The majority, 75.35%, were categorized as modifier type (impacting non-coding regions), followed by 15.71% classified as moderate (involving non-synonymous substitutions), 8.60% as low (involving synonymous coding/start/stop effects) and a small percentage, 0.34%, fell into the high category (affecting splice sites, stop codons, and start codons) (Supplementary Table S10). Most of the SNPs were in the flanking regions of the genes (66.20%). A considerable number of SNPs was classified as missense variant (15.19%). Among the various types of polymorphisms, transitions (55.03%) were more prevalent than transversions (44.97%), resulting in a Ts/Tv ratio of 1.22.

The Gene Ontology (GO) enrichment analysis was performed for the 27 SNPs predicted to have a high impact, considering both biological process (BP), and molecular function (MF) as shown in Supplementary Fig. S1. In the analysis of BP, a total of nine functions found to be significantly enriched. Among the top-ranked results, it was observed that BP is mainly associated with “signal transduction”, “proteolysis” and “protein phosphorylation”. In the MF analysis, 31 functions were found to be significantly enriched and included “protein binding”, “nucleic acid biding”, “zinc ion biding”, “ADP biding”, “Protein kinase activity” and metallopeptidase activity” (Supplementary Fig. S1).

The analysis of annotated terms showed that the 27 R-genes were overrepresented and played important roles in “protein biding”, ADP biding” and “signal transduction” (Supplementary Fig. S2). Additionally, the KEGG analysis identified an important pathway related to plant-pathogen interaction (K13457). The network former by genes that interact in families or share similar protein domains is depicted in Supplementary Fig. S3.

In the present study, as a practical action, numerous novel SNPs were integrated into target regions, refining the position of important quantitative trait loci (QTL) associated to disease resistance and providing valuable markers for use in marker-assisted breeding programs.

### Validation of the SNPs associated with R-genes

**Figure 8:**
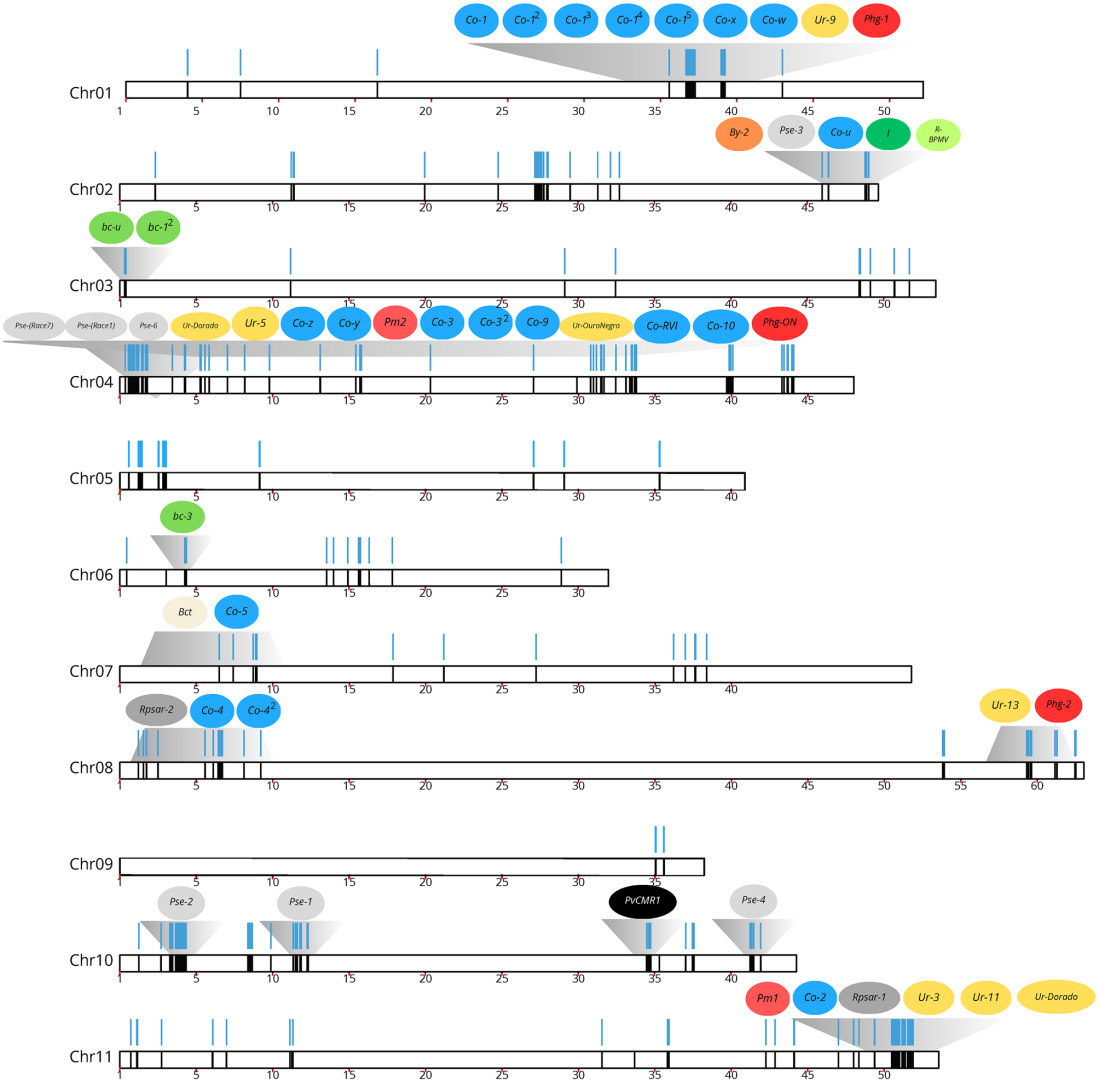
The representation of the 11 common bean chromosomes indicates the location of SNPs within genetically characterized disease resistance genes, as reviewed by Meziadi et al.^10^. The genotyped SNPs related to R-genes are represented by blue vertical lines. The approximate locations of disease resistance genes are indicated by a grey area connecting colored bubbles (R-genes) to their candidate locations on the chromosomes. The *Co* loci represent anthracnose (*Colletotrichum lindemuthianum*) resistance, *Ur* loci represent rust (*Uromyces appendiculatus*) resistance, *Phg* loci represent angular leaf spot (*Pseudocercospora griseola*) resistance, *Pm* loci represent powdery mildew (*Erysiphe diffusa*) resistance, and Pse or *Rpsar* loci represent halo blight (*Pseudomonas syringae*) resistance. The *I* locus represents resistance to BCMNV (*Bean common mosaic necrosis virus*), *By-2* is an R-gene for resistance to BYMV (*Bean yellow mosaic virus*) and CLYVV (*Clover yellow vein virus*), *PvCMR1* is an R-gene (TNL) for resistance to CMV (*Cucumber mosaic virus*), and *R-BPMV* for resistance to BPMV (*Bean pod mottle virus*). The *bc* loci represent recessive genes for resistance to BCMNV and BCMV.

Out of the 438 SNPs associated with R-genes that were evaluated, a total of 356 non-redundant R-genes were sampled (Supplementary Table S11). Among these, 92.61% were successfully genotyped, and 90.64% were found to be polymorphic. In terms of location, 82.9% of the genotyped SNPs were in genic regions, while 17.1% were in intergenic regions (Fig. 8). The Andean group (n = 14) exhibited a polymorphic loci percentage of 54.57%, which was lower compared to the Middle American group (n = 34) with a percentage of 82.42% polymorphic loci. The genetic diversity analysis revealed an average H_E_ of 0.334 (± 0.008) for the entire collection of SNPs. The genotypes from the Middle American gene pool exhibited higher heterozygosity (0.27 ± 0.009) compared to the Andean genotypes (0.19 ± 0.010). The coefficient of gene differentiation between the gene pools (F_*st*_) was 0.386 (± 0.001).

### Identification of tag SNPs in QTL regions

The Table 4 provides key information on QTLs associated with disease resistance in different cross combinations of common bean varieties. The crosses Rosinha G2 X BRS Cometa^28^, BRS Notável X BRS Supremo^29^, BRS Cometa X Sel 1308^30^, and BRS Sublime X CNFCT 16207^31^ were investigated for diseases such as Anthracnose (ANT), Fusarium Wilt (FW), and Cowpea mild mottle virus (CPMMV). QTL positions were identified on chromosomes Pv04, Pv07, and Pv08 at specific positions as reported in the respective references. A total of 110 SNPs were identified in silico within the genomic window containing the QTLs, and 8% of them were annotated to disease genes, with the aim of approaching the disease-causal variants (Supplementary Table S13).

**Table 4:** QTLs associated with fusarium wil (FW), anthracnose (ANT) and Cowpea mild mottle virus (CPMMV) in common bean.

Parents of population	Population type	Trait	Markers type	R2 value	Chromosome	QTL position	References
BRS Cometa (R) × Rosinha G2 (S)	F2 population	Anthracnose (ANT)	SNPs, Sequence-Tagged Sites (STS) and Sequence Characterized Amplified Regions (SCAR)	–	Pv04	354,682	Morais^28^
BRS Notável (R) × BRS Supremo (S)	F2 population	Fusarium Wilt (FW)	SNPs and SilicoDart	44.8%	Pv07	28,294,499	Cavalheiro^29^
BRS Cometa (R) × Sel 1308 (R)	F2 population	Anthracnose (ANT)	Simple Sequence Repeat (SSR), STS and SCAR	–	Pv08	2,368,816	Mota^30^
BRS Sublime (R) × CNFCT 16207 (S)	F2 population	Cowpea mild mottle virus (CPMMV)	SNPs and SilicoDart	~ 77%	Pv08	62,396,711	Silva^31^

The Supplementary Table S13 presents the obtained results, providing information about the location and characteristics of the identified SNPs. The functional annotations indicate the type of genetic variant found, including intergenic variants (27.3%), variants located in gene promoter (20.9%) and intronic regions (32.7%), and synonymous variants (19.1%). The genes associated with each SNP were also identified using their corresponding Transcript Gene Name (TGN) (Supplementary Table S13). These results offer insights into the genetic diversity within the studied QTL regions, contributing to the understanding of the genetic basis of the selected agronomic traits. Moreover, they may provide potential molecular markers to enhance the breeding of commercial bean varieties, aiming to improve the selected agronomic characteristics.

## Discussion

In the present study, we conducted resequencing of 40 agronomically important common bean breeding lines of Middle American and Andean origin, resulting in the identification of nearly 500,000 SNPs. These germplasms have been extensively studied worldwide for their resistance/susceptibility to various diseases, such as anthracnose, angular leaf spot, fusarium wilt, common bacterial blight, bean golden mosaic virus, and cowpea mild mottle virus. In a related study, Lobaton et al.^32^ resequenced 37 varieties of *P. vulgaris*, *P. acutifolius* (A. Gray) and *P. coccineus* L. leading to the identification of over 40 million genomic variants providing comprehensive resources for molecular breeding and further understanding of the genetic diversity within common bean populations. In a more extensive study, Wu et al.^12^ conducted resequencing of 683 accessions from the National Crop Genebank of China and successfully identified variants associated with yield components, of high value for breeding purposes. These collaborative initiatives in the scientific community are significantly contributing to the establishment of a comprehensive knowledge base, paving the way for advancements, facilitating gene discovery, and enhancing our understanding of key agronomic traits.

The analysis of the common bean accessions' raw data in the present study revealed an extensive collection of SNPs, exceeding 6.5 million within the dataset. Among these SNPs, 4.07 million were identified in non-repetitive regions, representing a significant increase of approximately 68% compared to the 1.3 million SNPs reported in non-repetitive regions by Lobaton et al.^32^. Moreover, a remarkable number of SNPs with MAF ≥ 0.05 (420,509) and MAF ≥ 0.1 (412,575) were identified, of which 63,366 SNPs effectively contributed to differentiate the Andean and Middle American groups (F_st_ ≥ 0.97), confirmed by the phylogenetic analysis. These extensively characterized set of SNPs covering the common bean genome holds great potential of applications, mainly if combined with datasets generated by other research groups^12,27,32,33^, as they enable the accurate selection of SNPs for candidate genes, as well as those distributed across the entire genome.

Despite the relatively small sample size in our study (n = 40), we observed higher estimates of nucleotide diversity (MA, n = 32, π = 0.38; and, n = 8, π = 0.28) compared to those reported by Delfini et al.^33^ (MA, n = 207, π = 0.31; And, n = 12, π = 0.22). The displayed higher genetic diversity in this study correlates with the wide range of genetic backgrounds of the sampled accessions, which was crucial for capturing a broader spectrum of genetic variations across the entire genome. The Ts/Tv ratio (1.29) observed exhibited minimal variation compared to other relevant bean-related studies^34,35^, aligning with our expectations^36^ and affirming the high quality of the data. Despite the substantial presence of introgressions in our study, only 99 of them exceeded 1 Mb in length, reflecting the findings of Lobaton et al.^32^, who reported 100 introgressions longer than 1 Mb in the evaluated germplasm. In general, a greater enrichment of the Middle American germplasm was observed, with 92.65% of the introgressions consisting of Andean germplasm segments. These findings are consistent with the history of bean domestication in Brazil, where Middle American germplasm prevails, but there has been a notable focus on hybridization with Andean germplasm to introduce target genes and enhance genetic variability in the development of breeding lines and cultivars^37,38^. Interestingly, the TO cultivar, which has a Middle American background exhibited a larger proportion of Andean introgression (21.63%), as shown on the phylogenetic tree (Fig. 5). Previously research by Valdisser et al.^35^ also reported a significant hybridization rate of 38% between gene pools, highlighting the importance of this process in achieving breeding objectives and accelerating genetic gains in legume cultivars^39^.

Among the 98,584 SNPs identified, 58% fell in introns, as also observed by Delfini et al.^33^. Variation in non-coding regions have an established role in plant diseases and regulatory mechanisms, with growing evidence from many areas, including yield, abiotic stresses, and adaptation to environment^40,41^. Genes conferring resistance to bean diseases have been extensively documented in the literature^11,42,43^. In this study, a total of 501 putative disease resistance genes were selected from the common bean genome obtained from phytozome, surpassing the previously reported numbers for the Andean (376 NB-LRR) and Middle American (234 NBS-LRR) germplasms^27,44^. Moreover, the discovery of SNPs in R-genes (7841 SNPs in 85.03% of the R-genes) uncovers significant polymorphisms associated with the defense response in beans enabling the development of genotyping arrays that target and explore multiple genomic regions^45^. These SNPs can have a functional impact in terms of phenotypic resistance or susceptibility to certain diseases.

The genotyping of 438 SNPs we conducted aimed to operationally assess a set of SNPs associated with resistance genes annotated in common bean. This research contribution is significant and unprecedented as it provides allelic variants located within resistance genes derived from 38 genotypes of great scientific value (Supplementary Table S12). These genotypes, including members of differential series used to differentiate races of *Colletotrichum lindemuthianum*, pathogen causing anthracnose^46^, and *Phaeoisariopsis griseola* which causes angular leaf spot^11^, have demonstrated their importance as valuable sources of resistance genes for breeders in the bean community. The validation and testing of this set of specific SNPs, associated with disease genes, using SNP arrays serves as the foundation for genome association studies and whole-genome-based selection in common beans. There is a growing demand for breeding programs to incorporate these SNPs into genotyping panels for diversity analysis, associative mapping, QTL mapping, and genomic selection^47^. The inclusion of SNPs associated with resistance genes in these genotyping panels enhances the value of molecular tools, enabling the detection of genetic variation in target genes. Our work provides a set of operationally evaluated SNPs that are readily suitable for incorporation into any genotyping chip.

Among these, a subset of 27 SNPs in R-genes with a putative high-impact effect showed to be clustered in a protein–protein interaction network, which may aid in the prioritization of targets regions for marker-assisted breeding and gene editing projects. Hence, this study not only provides a resource of characterized SNPs (Supplementary Table S13), but also offers a detailed view of genetic variations in R-genes, opening new opportunities for genomic research and related studies. Similarly, in a comprehensive review, Lin et al.^48^ provides valuable insights by consolidating information of genetic variants of numerous disease genes in soybean, serving as a toolbox for soybean improvement and providing support for breeding approaches.

Continuing within the scope of this study, the resequencing of inbred lines that incorporate crosses in which important QTLs were mapped^28–31^ provided a valuable opportunity to enhance the resolution of genetic regions associated with diseases. The genotyping of these SNPs in future studies, with increased sample size, when possible, has the potential to improve the resolution of these previously mapped QTLs. A similar approach was taken with soybean, in which the resequencing of contrasting mungbean yellow mosaic India virus (MYMIV) tolerant cultivars led to the identification of SNPs associated with target QTLs^49^, aiming to increase the likelihood and identifying more accurate molecular tools for MAS. Recently, Yan et al.^50^ refined four major QTLs for oil content in *Brassica napus* by integrating resequencing data and transcriptomics. In soybean, whole-genome sequencing allowed the identification of genomic variations and candidate resistance genes for soybean mosaic virus (SMV), opening possibilities for the development of resistant varieties^51^. Additionally, genotyping these target SNPs can optimize genomic selection approaches, enhancing the accuracy of predictions, as demonstrated for fusarium head blight resistance and yield-related traits in wheat^52^.

In conclusion, by re-sequencing and making more variants of *P. vulgaris* publicly available, we can achieve a more comprehensive understanding of the genetic variation within this species. This expanded dataset allows for the identification of both common and rare genetic variants, as well as a deeper understanding of their frequencies, enabling more robust analyses and accurate predictions of agronomic trait mechanisms. Ultimately, the availability of this valuable genetic information contributes to the development of personalized breeding approaches that can lead to improved crop varieties with desired traits. The integration of this dataset with other valuable omics data available for common bean can also drives advancements in various research areas beyond genomics. Additionally, it creates new opportunities for scientific advancement and interdisciplinary collaborations, accelerating progress in common bean genetics, genomics, and related fields.

## Methods

### Plant material and DNA extraction

A germplasm panel comprising 40 common bean lines/cultivars was carefully selected for this study. The genotypes were chosen based on their significance in breeding programs worldwide. Some of these have served as essential background accessions for modern lines (e.g., PI 207262, Bat 332, CB 911921) and have been cultivated in Brazil for many years (e.g., Pérola). To represent the historical breeding genetic diversity of common bean, we sequenced a combination of modern elite cultivars (e.g., BRS Ártico, BRS FS305) and older varieties (e.g., Ruda, Ouro Branco, Jalo Precoce). We also included lines associated with important disease resistance (e.g., AND277, PI 207262, Sel 1308) as well as members of differential series used to differentiate races of pathogens causing anthracnose and angular leaf spot diseases (e.g., G2333, TO, Mexico 54). Additionally, we carefully selected lines with other desirable agronomic traits, such as specific plant architecture and darkening of the tegument (e.g., CNFC 10467, BRS Estilo); adapted to different regions and exhibiting variations in grain types (e.g., BRS Executivo, IAC diplomata, BRSMG Realce); from diverse institutions of origins and representing both the Andean and Mesoamerican gene pools (e.g., México 54, CNFC 10467, BRS Estilo, IAC Imperador, IPA9). The seeds utilized in this study were obtained from the germplasm bank of Embrapa Rice and Beans (Supplementary Table S1).

Total genomic DNA was extracted from leaves at the V4 growth stage (4th trifoliolate unfolded at node 6 and branching), using the Invisorb Spin Plant Mini Kit (Stratec Molecular, Berlin, Germany) following the manufactures’ instructions. For genomic DNA library preparation, the Nextera^®^ DNA flex kit was employed. DNA sequencing was carried out on the Illumina HiSeqX platform using a PE150 (paired-end 150) strategy.

### Sequencing raw data analysis

The raw sequence data obtained were processed using Trimmomatic software v. 0.39^53^ for quality trimming (SLIDINGWINDOW:10:30) and adaptor sequences removal (TruSeq3-PE-2), keeping reads with a length of at least 50 base pairs (bp). The filtered reads were subsequently aligned to the reference bean genome^27^ (https://phytozome-next.jgi.doe.gov/info/Pvulgaris_v2_1) using BWA mem software^54^ using default parameters.

### Variant detection

For variant calling we used FreeBayes v.1.3.1^55^ and VCFTools software v.0.1.16^56^ was utilized for variant filtering, applying the following parameters: Min_Alleles = 2, Max_Alleles = 2; Min_Mean_Depth = 10; Max_Mean_Depth = 750; Mindp = 3; Maf = 0,01, Mac = 1; Perc_Missing = 0.4; Min_Qual = 10.0.

To identify the chromosomal coordinates of repetitive DNA we accessed Phytozome platform using the Andean common bean reference genome^27^. SNPs located within these repetitive regions were filtered out using VCFTools software^56^ with the “–exclude-bed” command. SNPs with a *Minor allele frequency* (MAF) ≥ 0.05 were retained. Ultimately, a hard filtering approach was applied to ensure quality control using the vcfR package^57^ in the R environment^58^, following the parameters: call_rate ≥ *0.8*; Allele frequency for each alternative allele (AF) >  = 0.1 and <  = 0.9; QUAL ≥ 30; Strand balance probability for the alternate allele (SAP) > 20; Strand balance probability for the reference allele (SRP) > 20; End placement probability (EPP) > 20 and Depth(DP) ≤ 1500.

Using the NGSEP software v.4.0.1^59^, genotypes for each accession were determined using the VCF Summary Stats command. The genotypes were classified into three categories for a biallelic site in a diploid individual: (i) Homozygous and identical to the reference allele, (ii) heterozygous, and (iii) homozygous but different from the reference (Homozygous alternative). SNP predictions of functional impact were performed using the command VCF Summary Annotate at NGSEP software^59^.

For SNPs with putative effect predicted to be high, a functional enrichment Tusing the MeSH over-representation analysis (ORA), including gene ontology (GO) and KEGG (Kyoto Encyclopedia of Genes and Genomes) pathway enrichment analysis, were performed using the function g:PROFILER in Python^60^ and Quick GO *web-based tool*^61^. A significance level of p-value < 0.05 was applied, and the results from GO and KEGG were visualized using R with the ggplot2 package^62^. The enrichment in the protein domain was established using the protein family (PFAM) analysis tool available at InterPro website^63^. Additionally, gene–gene interaction prediction was conducted based on the protein sequences alignment (genes and domains) using the resources available at Phytozome.

### Genetic diversity analysis

Genetic diversity (H_E_) was calculated using the complete set of SNPs, filtered at every 50 kb interval using the hierfstat v.0.5-10^64^ and pegas v.1.1 packages^65^ in the R environment^58^. A Venn diagram depicting the distribution of SNPs within and between gene pools was generated using the JVENN tool^66^. Polymorphic SNPs that were found exclusively or common among the gene pools were used to estimate fixation indices (F_st_)^67^, nucleotide diversity (π) and Tajima’s D^68^ within a 100 kb sliding window using the VCFtools software^56^.

Phylogenetic analysis was conducted using the SNPs grouped into bins of 10 kb employing the RAxML v.8.2^69^ and rapid bootstrap algorithms to identify the best score combined with maximum likelihood (ML). The General Time Reversible (GTR) model coupled with a gamma-distribution (G) rate, was applied. Branch consistency was accessed through 10,000 bootstraps. The resulting tree was visualized using FigTree program v.1.4.4 accessible at http://tree.bio.ed.ac.uk/software/figtree/.

### Introgression analysis

Introgressed analysis was performed to identify genomic regions that have undergone introgression between Andean and Middle American. The VCF Introgression Analysis module in the NGSEP software^59^ was utilized for this purpose considering non-overlapping windows of 50 SNPs.

### R-genes

Genes associated with disease resistance, specifically R-genes, were obtained from the Phytozome v.2.1 platform (Phytozome (doe.gov). Furthermore, a search for conserved putative domains for R-genes, well documented in the literature^70^ was conducted. SNPs located within 5 kb upstream/downstream regions of the UTR 5' and 3' of the R-genes were filtered using VCFtools^56^. Pearson's correlation coefficients were calculated in R software^58^ to assess linear relationships between key variables^56^.

### Validation of the SNPs associated with R-genes

A set of 438 SNPs was selected from a total of 7841 SNPs (1.86% of the total) previously identified in the R-gene sequences (Fig. 8). The Axiom GnxUYv1 Affymetrix custom array, which contains these selected SNPs, was manufactured by Thermo Fisher Scientific (Waltham, MA, USA). The array was used for genotyping 48 common bean accessions, including 34 cultivars/lines from the Middle American gene pool and 14 from the Andean gene pool (Supplementary Table S12). Genomic DNA from individual plants was extracted using the Invisorb Spin Plant Mini Kit (Stratec Molecular, Berlin, Germany) and then sent to a Genexa (Genexa—Adn evolutivo) facility for genotyping. Overall genetic parameters were calculated using GenAlex v. 6.502^71^.

### Identification of tag SNPs in QTL regions

The identification of SNPs flanking the disease QTL in four bi-parental crosses Rosinha G2 X BRS Cometa^28^, BRS Notável X BRS Supremo^29^, BRS Cometa X Sel 1308^30^, and BRS Sublime X CNFCT 16207^31^(Table 4) was performed through the alignment of the genetic and physical map. We used the QTL confidence intervals to delimit the flanking sequences within the *P. vulgaris* reference genome (https://phytozome-next.jgi.doe.gov/). Additionally, functional annotations were gathered for each SNP, describing the type of genetic variant found, such as intergenic variants, variants located in gene promoter regions, synonymous variants, and intronic variants.

### Supplementary Information

Supplementary Information.

## Data Availability

The datasets generated during the current study are available under the accession number PRJNA955663 (https://www.ncbi.nlm.nih.gov/bioproject/PRJNA955663).
