# Supplementary material for: Whole-genome resequencing of common bean elite breeding lines
Source: Sci Rep. 2023 Aug 5;13:12721. doi: 10.1038/s41598-023-39399-6 (PMC10404220; doi:10.1038/s41598-023-39399-6)
Supplement: Supplementary file 1 — Supplementary Information. [file 41598_2023_39399_MOESM1_ESM.pdf]

Supplementary Information

Whole-genome resequencing of common bean elite breeding lines

Isabela Pavanelli de Souza[1*], Beatriz Rosa de Azevedo[2], Alexandre Siqueira Guedes Coelho[3], Thiago Lívio Pessoa Oliveira de Souza[4], Paula Arielle Mendes Ribeiro Valdisser[5]; Lucas Matias Gomes Messias[6], Breno Osvaldo Funicheli [7], Claudio Brondani[8], Rosana Pereira Vianello[8]

1 Postdoctoral Student in Biology, Embrapa Arroz e Feijão, Santo Antônio de Goiás, GO. Email: isabela.pavanelli@gmail.com.
2 Undergraduate Student in Biotechnology, Scientific Initiation Scholarship at Embrapa Arroz e Feijão, Santo Antônio de Goiás, GO.
3 PhD in Plant Breeding, Federal University of Goiás, Goiânia, GO.
4 PhD in Plant Breeding, Embrapa Arroz e Feijão, Santo Antônio de Goiás, GO
5 Master in Genetics and Molecular Biology, Embrapa Arroz e Feijão, Santo Antônio de Goiás, Brazil
6 PhD in Genetics and Plant Breeding.
7 Computer Scientist, Master candidate in Computer Science, Universidade Federal de São Carlos, São Carlos, SP
8 PhD in Molecular Biology, Embrapa Arroz e Feijão, Santo Antônio de Goiás, GO.

SUPPLEMENTARY FIGURES

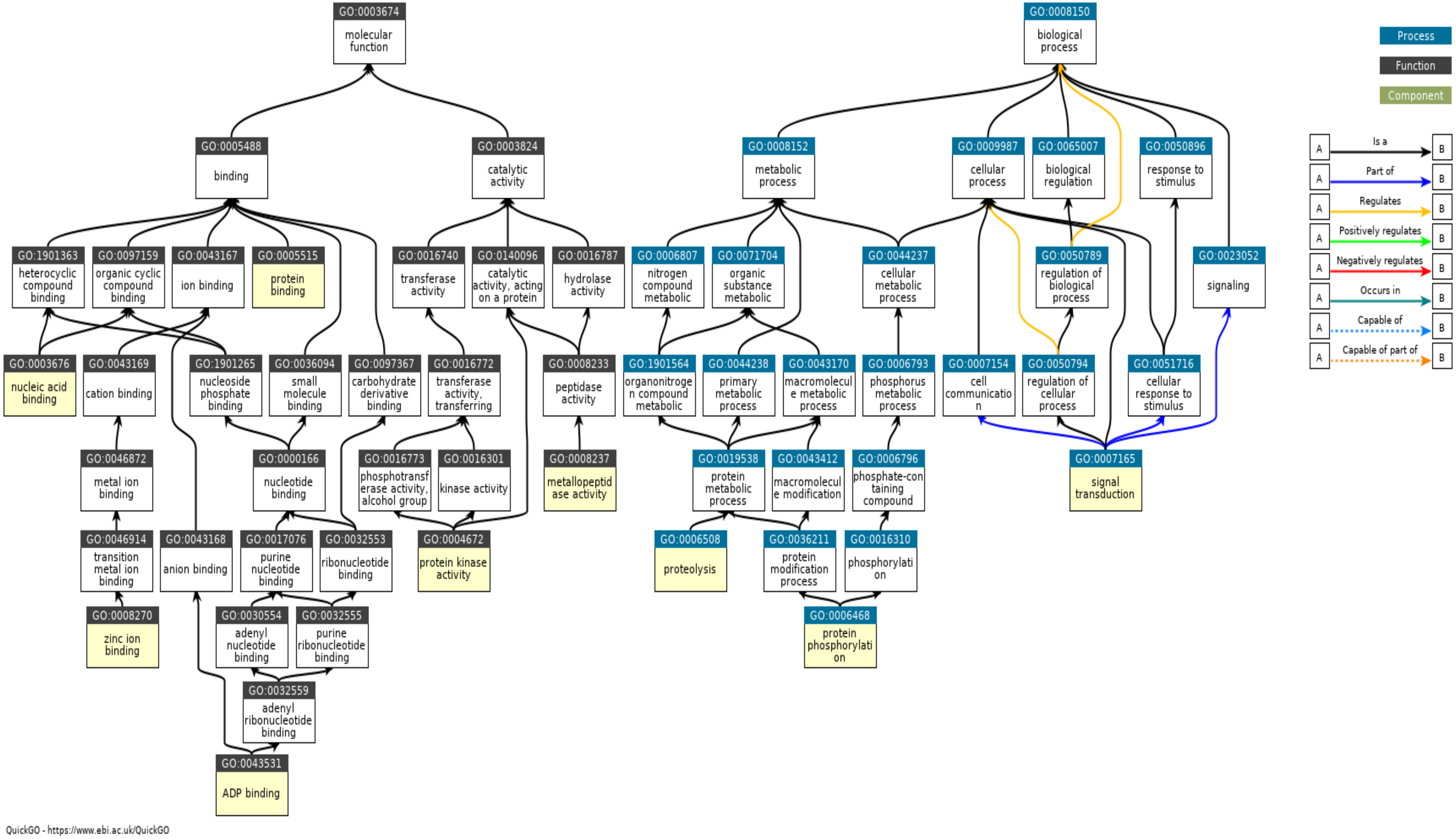

Supplementary Figure S1. Hierarchical tree graph of GO terms for all 27 R-genes related within a Molecular Function (top bar in black) and Biological Process (top bar in blue). Boxes in the graphs represent GO terms labelled by GO number and term definition. The terms highlighted in yellow represents greater level of statistical significance (smaller p-value) of the enrichment. This figure exemplifies all ontological terms that have been linked to resistance genes.

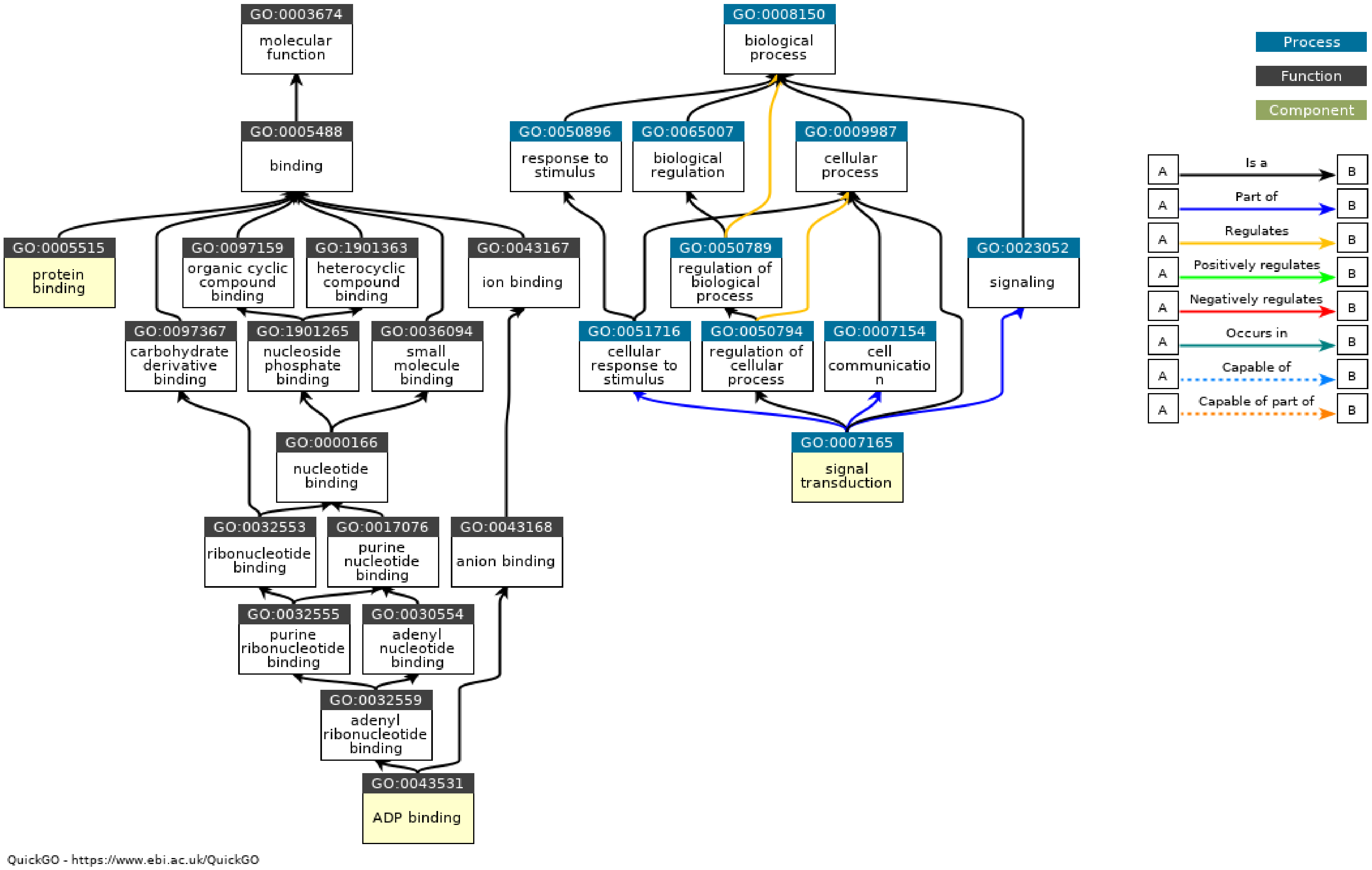

Supplementary Figure S2. Hierarchical tree graph of GO for the overrepresented enriched R-genes obtained from hypergeometric distribution. These terms were related within a Molecular Function (top bar in black) and Biological Process (top bar in blue). Boxes in the graphs represent GO terms labelled by GO number and term definition. The terms highlighted in yellow represents greater level of statistical significance (smaller p-value) of the enrichment.

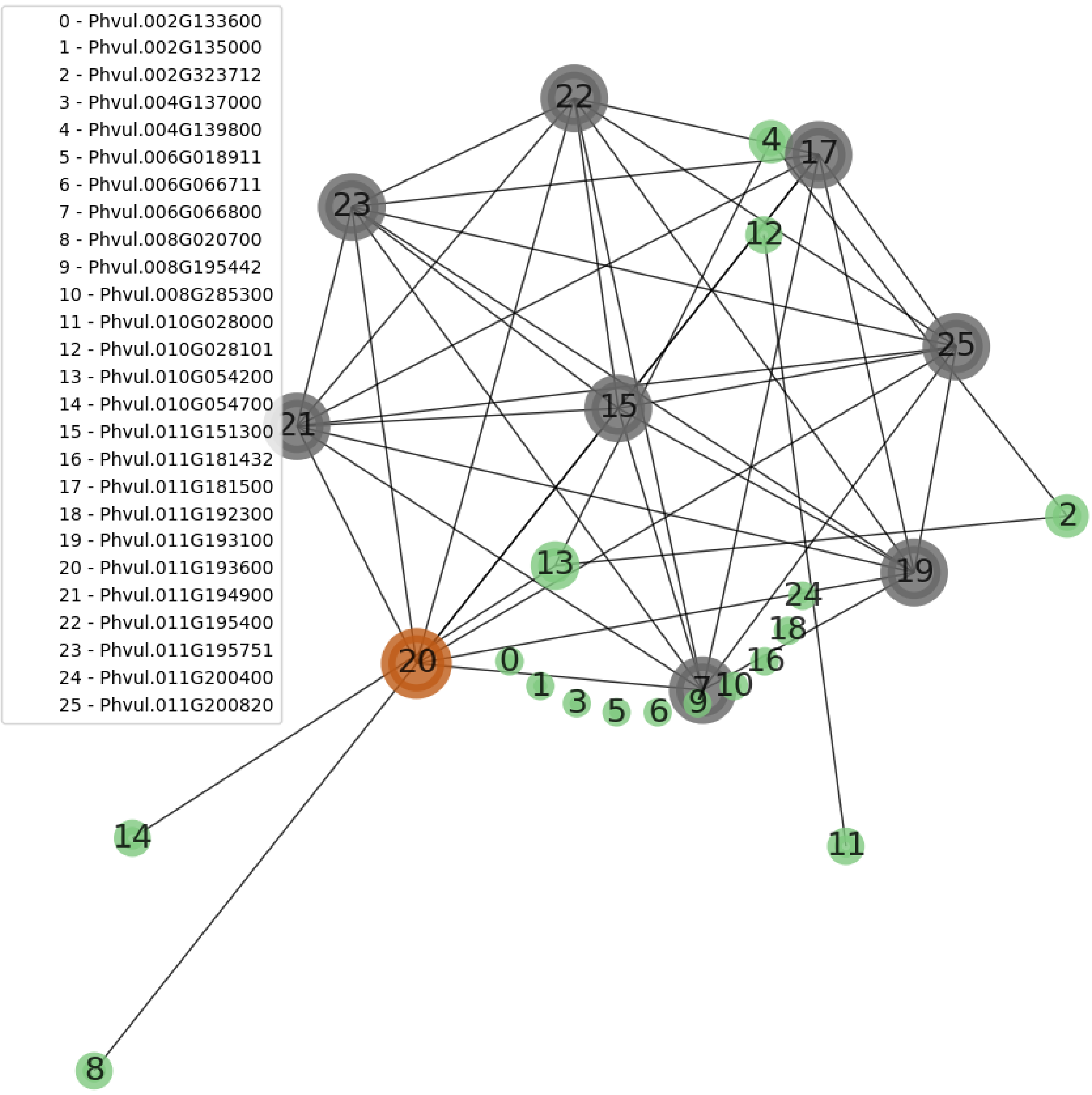

Supplementary Figure S3. Disease genes-gene interaction network. Each node is a gene, with genes being connected if they are annotated in the same protein family. The size of each node is proportional to the number of disorders in which the gene is implicated (see key). Nodes are light gray if the corresponding genes are associated with more than one disorder class. Gene names are represented in the figure legend. The figure was generated with Matplotlib (https://matplotlib.org/) and networkx (https://networkx.org/), both in python.

SUPPLEMENTARTY TABLES

Supplementary Table S1. Information about the 40 common bean genotypes selected for SNP detection using WGS strategy.

Sample	Gene pool	Germoplasm	Release date	Grain type	Institution	Disease reaction*								Resistance reference
						ANT	ALS	FW	CBB	BW	BGMV	CPMMV	AT	
AND277	Andean	Cultivar		Mottled	CIAT	R	R	-	-	-	-	-		LIST OF GENES - *Phaseolus vulgaris* L (uprm.edu)
Bat 332	Middle American	Line		Cream	CIAT	-	R	-	-	-	-	-		LIST OF GENES - *Phaseolus vulgaris* L (uprm.edu)
BRS Ametista	Middle American	Cultivar	2010	Carioca	EMBRAPA	R	S	MR	MR	MR	S	R		Melo et al. (2012)
BRS Ártico (WAF 75)	Andean	Cultivar	2015	White	EMBRAPA	R	S	S	S	MR	S	-		Pereira et al. (2016)
BRS Cometa	Middle American	Cultivar	2005	Carioca	EMBRAPA	R	S	-	S	-	S	-		Faria et al. (2008)
BRS Embaixador	Andean	Cultivar	2008	Red	EMBRAPA	MR	S	MR	S	S	S	-		Aidar et al. (2008a)
BRS Esplendor	Middle American	Cultivar	2008	Black	EMBRAPA	R	S	R	R	-	S	-	EP	Costa et al. (2011)
BRS Esteio	Middle American	Cultivar	2012	Black	EMBRAPA	R	S	S	S	S	S	-		Pereira et al. (2013)
BRS Estilo	Middle American	Cultivar	2009	Carioca	EMBRAPA	R	S	S	S	S	S	-	EP	Melo et al. (2010)
BRS Executivo	Andean	Cultivar	2008	Sugar Bean	EMBRAPA	MR	S	MR	S	MR	S	-		Aidar et al. (2008b)
BRS FC104	Middle American	Cultivar	2016	Carioca	EMBRAPA	S	S	S	S	S	S	-		Melo et al. (2019)
BRS FP403	Middle American	Line	2016	Black	EMBRAPA	S	S	R	S	S	S	-		Souza et al. (2019)
BRS FS305 (CAL 96)	Andean	Cultivar	2018	Calima	EMBRAPA	MR	MR	-	-	S	S	-		Pereira et al. (2020)
BRS Horizonte	Middle American	Cultivar	2004	Carioca	EMBRAPA	R	S	-	S	-	-	-		Melo et al. (2005)
BRS Madrepérola	Middle American	Cultivar	2011	Carioca	EMBRAPA	R	MR	S	S	S	S	-		Carneiro et al. (2012)
BRS Notável	Middle American	Cultivar	2010	Carioca	EMBRAPA	MR	S	R	MR	MR	S	-		Pereira et al. (2012)
BRS Pérola	Middle American	Cultivar	1994	Carioca	EMBRAPA	S	MR	MR	S	S	S	S		Wendland et al. (2018)
BRS Sublime	Middle American	Cultivar	2013	Carioca	EMBRAPA	MR	R	S	S	MR	S	R		Wendland et al. (2018)
BRS Supremo	Middle American	Cultivar	2005	Black	EMBRAPA	R	MR	-	S	-	S	-		Costa et al. (2006)
BRSMG Realce	Andean	Cultivar	2011	Rajado	EMBRAPA	R	S	MR	MR	R	S	-		Melo et al. (2014)
CB 911921	Middle American	Line		Black	EMBRAPA	-	-	-	R	-	-	-		Rava et al. (1996)
CNFC 10467	Middle American	Line		Carioca	EMBRAPA	-	S	-	S	-	-	-	SD	Pontes-Júnior et al. (2012)
CNFCT 16207	Middle American	Line		Carioca	EMBRAPA	-	-	-	-	-	R	S		Silva et al. (2022)
CNFCT 19120	Middle American	Line		Carioca	EMBRAPA	-	-	-	-	-	R	R		Silva et al. (2022)
G2333	Middle American	Cultivar		Red	CIAT	R	-	-	-	-	-	-		LIST OF GENES - *Phaseolus vulgaris* L (uprm.edu)

IAC Diplomata	Middle American	Cultivar	2007	Black	IAC	R	-	-	-	-	-	-	Carbonell et al. (2008)
IAC Imperador	Middle American	Cultivar	2012	Carioca	IAC	R	-	R	-	-	-	-	Chiorato et al. (2012)
IPA 9	Middle American	Cultivar		Cream	IPA	-	-	-	-	R	-	-	Costa et al. (2012)
Jalo Precoce	Andean	Cultivar	1993	Jalo	EMBRAPA	S	MR	-	MR	-	S	-	Embrapa Rice and Beans (2008)
MA III-16.159	Middle American	Line		Carioca	UFLA/EMBRAPA	-	R	-	-	-	-	-	Pádua et al. (2021)
México 54	Middle American	Cultivar		Light pink	CIAT	-	R	-	-	-	-	-	LIST OF GENES - *Phaseolus vulgaris* L (uprm.edu)
Ouro Branco	Andean	Cultivar	1993	White	CIAT	S	S	-	S	-	-	-	Pereira et al. (2016)
Ouro Negro	Middle American	Cultivar	1991	Black	UFV/EPAMIG	R	R	-	-	-	-	-	LIST OF GENES - *Phaseolus vulgaris* L (uprm.edu)
PI 207262	Middle American	Cultivar		Cream	CIAT	R	-	-	-	-	-	-	LIST OF GENES - *Phaseolus vulgaris* L (uprm.edu)
Rosinha G2	Middle American	Cultivar		Rosinha	IAC	S	-	-	-	-	-	-	Vieira et al. (2018)
Rudá	Middle American	Cultivar	1994	Carioca	CIAT	S	S	-	-	-	-	-	Vieira et al. (2018)
Sel 1308	Middle American	Line		Black	CIAT	R	-	-		-	-	-	LIST OF GENES - *Phaseolus vulgaris* L (uprm.edu)
TO	Middle American	Cultivar		Carioca	CIAT	R	-	-	-	-	-	-	LIST OF GENES - *Phaseolus vulgaris* L (uprm.edu)
Uirapuru	Middle American	Cultivar	2000	Black	IAPAR	S	S	S	-	-	-	-	Borba et al. (2017)
US Pinto 111	Middle American	Cultivar		Carioca	CIAT	-	S	-	-	-	-	-	Corrêa et al. (2001)

*For more information about pathotype-specific or field resistance, check the articles (hiperlink) in the resistance reference column.

ANT, Anthracnose; ALS, Angular Leaf Spot; FW, Fusarium Wilt; CBB, Common Bacterial Blight; BW, Bacterial Wilt; BGMV, Bean Golden Mosaic Virus; CPMMV, Cowpea Mild Mottle Virus; AT, Agronomic Trait; SD, Slow-Darkening; EP, Erect Plant

R, Resistant (score 1.0 to 3.0); MR, Moderately Resistant (scores 3.1 to 4.0); S, Susceptible (scores 4.1 to 9).

Supplementary Table S2. WGS sequence performance of the 40 common bean genotypes included in this study.

Sample	Raw Data		Coverage (X)*
	Total reads	Base pairs	
BRS Madrepérola	54,437,952	8,165,692,800	13.91
BRS Ametista	44,436,296	6,665,444,400	11.36
G2333	66,328,078	9,949,211,700	16.95
CB 911921	45,947,746	6,892,161,900	11.74
BRS Sublime	45,937,734	6,890,660,100	11.74
Sel 1308	46,452,708	6,967,906,200	11.87
MAIII 16159 (UFLA)	48,559,540	7,283,931,000	12.41
CNFC 10467	59,732,062	8,959,809,300	15.26
IAC Imperador	52,112,306	7,816,845,900	13.32
México 54	47,322,456	7,098,368,400	12.09
Jalo Precoce	61,563,416	9,234,512,400	15.73
BRS Ártico (WAF 75)	47,390,362	7,108,554,300	12.11
BRSMG Realce	35,421,738	5,313,260,700	9.05
CNFC 16207	39,928,724	5,989,308,600	10.20
BRS Executivo	66,279,032	9,941,854,800	16.94
Ouro Negro	63,872,768	9,580,915,200	16.32
PI 207262	51,450,812	7,717,621,800	13.15
CNFC 10794	69,267,536	10,390,130,400	17.70
BRS Embaixador	53,268,708	7,990,306,200	13.61
BRS Horizonte	51,732,056	7,759,808,400	13.22
US Pinto 111	46,691,380	7,003,707,000	11.93
BRS Supremo	43,702,176	6,555,326,400	11.17
BRS FS305 (CAL 96)	59,219,342	8,882,901,300	15.13
Bat 332	53,534,678	8,030,201,700	13.68
Rosinha G2	34,366,018	5,154,902,700	8.78
CNFCT 19120	35,834,432	5,375,164,800	9.16
OURO BRANCO	40,189,440	6,028,416,000	10.27
BRS Cometa	38,754,228	5,813,134,200	9.90
TO	40,696,348	6,104,452,200	10.40
IPA 9	56,640,402	8,496,060,300	14.47
AND 277	593,086,152	88,962,922,800	151.56
Rudá	968,659,406	145,298,910,900	247.53
BRS Pérola	1,403,183,010	210,477,451,500	358.56
BRS Estilo	620,023,202	93,003,480,300	158.44
BRS Notável	474,551,190	71,182,678,500	121.27
BRS Esteio	533,122,952	79,968,442,800	136.23
BRS Esplendor	454,044,208	68,106,631,200	116.02
FC104	304,649,350	45,697,402,500	77.85
IAC Diplomata	563,146,450	84,471,967,500	143.90
Uirapuru	39,088,608	5,863,291,200	9.99
**Total**	**7,454,625,002**	**1,118,193,750,300**	**1,904.93**

*Estimate coverage was based on a 587 Mb genome size (Schmutz et al., 2014)

Supplementary Table S3. Number of SNPs genotyped according to the type of genotype demonstrated for the 40 accessions of common bean used in this study

**Sample**	**Gene pool**	**Genotyped**	**Homozygous reference**	**Non reference**	
				**Homozygous alternative**	**Heterozygous**
AND 277	Andean	418,254	370,770	45,061	2,423
BRS Embaixador	Andean	405,802	354,178	47,256	4,368
BRS FS305 (CAL 96)	Andean	412,620	356,979	51,297	4,344
BRS Ártico (WAF 75)	Andean	402,588	348,045	50,137	4,406
Jalo Precoce	Andean	412,691	352,738	55,165	4,788
BRS Executivo	Andean	413,364	353,165	55,360	4,839
OURO BRANCO	Andean	371,457	313,111	53,655	4,691
BRSMG Realce	Andean	365,570	300,554	45,540	19,476
TO	Middle American	374,569	213,582	150,427	10,560
BRS Cometa	Middle American	381,750	212,612	157,361	11,777
Rudá	Middle American	419,860	225,845	181,371	12,644
Rosinha G2	Middle American	346,232	184,502	150,974	10,756
IAC Imperador	Middle American	403,587	213,429	172,867	17,291
BRS Horizonte	Middle American	388,607	205,434	170,123	13,050
IAC Diplomata	Middle American	419,476	221,496	185,423	12,557
BRS Pérola	Middle American	418,970	221,210	185,539	12,221
CNFCT 19120	Middle American	349,343	183,186	154,510	11,647
BRS Ametista	Middle American	388,057	202,930	172,831	12,296
CNFC 10467	Middle American	408,439	213,093	181,759	13,587
Sel 1308	Middle American	396,079	205,254	178,666	12,159
BRS Sublime	Middle American	399,106	206,586	180,225	12,295
BRS Notável	Middle American	419,222	216,751	190,290	12,181
CB 911921	Middle American	394,908	203,243	179,601	12,064
BRS Estilo	Middle American	418,956	214,442	192,393	12,121
BRS Esteio	Middle American	419,023	213,635	188,630	16,758
BRS Supremo	Middle American	386,047	196,174	175,621	14,252
CNFC 16207	Middle American	368,210	186,911	169,729	11,570
Uirapuru	Middle American	356,860	181,066	145,621	30,173
MAIII 16159 (UFLA)	Middle American	387,217	195,670	179,035	12,512
Bat 332	Middle American	404,647	203,240	188,306	13,101
IPA 9	Middle American	407,136	204,218	190,287	12,631
FC104	Middle American	419,235	209,726	196,830	12,679
BRS Esplendor	Middle American	418,735	209,112	197,131	12,492
BRS Madrepérola	Middle American	403,753	198,830	192,715	12,208
CNFC 10794	Middle American	408,760	201,225	190,177	17,358
Ouro Negro	Middle American	411,789	192,567	199,440	19,782
PI 207262	Middle American	401,442	187,374	195,214	18,854
México 54	Middle American	386,960	179,359	195,407	12,194
US Pinto 111	Middle American	382,006	176,854	192,561	12,591
G2333	Middle American	407,550	161,581	170,527	75,442

Supplementary Table S4. Distance between pairwise SNPs along each of the 11 common bean chromosomes in the set of 40 common bean accessions.

Chromosome	Distance (bp)		
	No. of SNPs	Mean	Max
Chr01	19,181	2,681.42	783,595
Chr02	52,200	951.35	218,761
Chr03	27,832	1,919.53	406,636
Chr04	19,714	2,435.82	485,536
Chr05	32,437	1,261.49	417,187
Chr06	33,211	940.18	225,747
Chr07	40,972	977.21	344,234
Chr08	70,650	891.89	342,780
Chr09	32,515	1,175.43	187,241
Chr10	40,491	1,093.67	454,038
Chr11	51,306	1,044.23	529,768
**Overall**	**420,509**	**1,397.47**	**783,595**

Supplementary Table S5. Number of SNPs in gene regions in each of the 11 common bean chromosomes in the set of 40 common bean accessions.

Chrom	Intergenic	Gene	5 prime UTR	Exon	Intron	3 prime UTR
Chr01	15,381	3,800	195	1,110	2,230	265
Chr02	40,084	12,116	619	3,877	6,739	881
Chr03	22,318	5,514	281	1,428	3,507	298
Chr04	15,876	3,838	178	1,098	2,365	197
Chr05	24,712	7,725	387	2,361	4,512	465
Chr06	24,338	8,873	495	2,823	5,012	543
Chr07	30,076	10,896	486	3,277	6,404	729
Chr08	54,607	16,043	759	5,340	8,955	989
Chr09	26,077	6,438	341	1,919	3,761	417
Chr10	30,873	9,618	391	2,905	5,810	512
Chr11	37,583	13,723	530	4,417	7,990	786
**Total**	**321,925**	**98,584**	**4,662**	**30,555**	**57,285**	**6,082**

Supplementary Table 6. Number of common and exclusive SNPs between gene pools in each of the 11 common bean chromosomes in the set of 40 common bean accessions

Chromosome	Differentiating	Common	Exclusives	
			Andean	Middle American
Chr01	5,186	1,408	1,105	11,482
Chr02	8,520	6,544	5,900	31,236
Chr03	7,259	2,044	1,229	17,300
Chr04	2,558	4,971	1,660	10,525
Chr05	4,283	4,630	2,996	20,528
Chr06	3,595	3,624	4,443	21,549
Chr07	7,009	3,283	2,567	28,113
Chr08	7,548	6,429	3,839	52,834
Chr09	8,274	1,771	3,252	19,218
Chr10	2,126	5,592	2,097	30,676
Chr11	7,008	8,685	5,845	29,768
**Total**	**63,366**	**48,981**	**34,933**	**273,229**

Supplementary Table S7. List of inter-gene pool introgressions based on the 40 common bean accessions.

Chr	Start	End	Length	Sample	Background group	Haplotype group	Total variants	Genotyped variants	Score Andean	Score Middle
Chr01	11,503,873	29,038,023	17,534,150	TO	Middle	Andean	1450	1347	1320	-1320
Chr01	25,934,137	42,917,486	16,983,349	IAC Imperador	Middle	Andean	3000	2945	2824	-2824
Chr01	6,165,652	11,274,394	5,108,742	TO	Middle	Andean	1450	1331	1315	-1315
Chr01	3,506,058	6,025,128	2,519,070	IAC Imperador	Middle	Andean	800	782	742	-742
Chr01	10,527,670	12,616,540	2,088,870	ESPLENDOR	Middle	Andean	350	349	303	-303
Chr01	10,527,670	12,616,540	2,088,870	IPA 9	Middle	Andean	350	339	290	-290
Chr01	10,527,670	12,616,540	2,088,870	PI 207262	Middle	Andean	350	338	285	-285
Chr01	10,527,670	12,616,540	2,088,870	US Pinto 111	Middle	Andean	350	323	279	-279
Chr01	47,811,416	49,900,199	2,088,783	TO	Middle	Andean	1800	1674	1627	-1627
Chr01	31,008,107	32,690,783	1,682,676	TO	Middle	Andean	150	135	131	-131
Chr01	49,964,228	51,294,732	1,330,504	BRS Horizonte	Middle	Andean	1200	1136	1079	-1079
Chr01	44,616,817	45,936,069	1,319,252	IAC Imperador	Middle	Andean	500	475	449	-449
Chr01	19,130,361	20,297,933	1,167,572	Sel 1308	Middle	Andean	100	95	79	-79
Chr01	1,024,728	2,104,801	1,080,073	TO	Middle	Andean	750	698	684	-684
Chr01	2,154,826	3,146,760	991,934	TO	Middle	Andean	600	549	502	-502
Chr01	46,767,728	47,659,694	891,966	TO	Middle	Andean	400	369	361	-361
Chr01	48,216,596	48,997,238	780,642	ESTEIO	Middle	Andean	1050	1050	1036	-1036
Chr01	45,936,485	46,682,384	745,899	TO	Middle	Andean	250	238	218	-218
Chr01	655	518,927	518,272	Rosinha G2	Middle	Andean	350	326	318	-318
Chr01	50,109,844	50,465,673	355,829	BRS Madrepérola	Middle	Andean	500	492	485	-485
Chr01	50,109,844	50,465,673	355,829	ESTEIO	Middle	Andean	500	500	496	-496
Chr01	19,130,361	19,467,336	336,975	G2333	Middle	Andean	50	47	32	-32
Chr01	10,527,670	10,846,056	318,386	México 54	Middle	Andean	50	49	43	-43
Chr01	10,527,670	10,846,056	318,386	Ouro Negro	Middle	Andean	50	50	43	-43
Chr01	50,109,844	50,406,909	297,065	Rosinha G2	Middle	Andean	400	360	349	-349
Chr01	8,180,429	8,416,528	236,099	ESPLENDOR	Middle	Andean	50	50	31	-31
Chr01	8,180,429	8,416,528	236,099	México 54	Middle	Andean	50	48	32	-32
Chr01	6,756,947	6,988,763	231,816	ESPLENDOR	Middle	Andean	150	150	132	-132
Chr01	48,766,677	48,997,238	230,561	Rosinha G2	Middle	Andean	300	269	251	-251
Chr01	18,320,643	18,547,396	226,753	Sel 1308	Middle	Andean	50	48	35	-35
Chr01	48,360,076	48,549,998	189,922	Rosinha G2	Middle	Andean	250	216	216	-216
Chr01	50,109,844	50,281,706	171,862	ESPLENDOR	Middle	Andean	250	250	244	-244
Chr01	7,390,186	7,561,821	171,635	ESPLENDOR	Middle	Andean	50	49	35	-35
Chr01	828,089	978,655	150,566	TO	Middle	Andean	100	93	93	-93
Chr01	45,043,573	45,186,649	143,076	TO	Middle	Andean	50	44	30	-30
Chr01	1,275,393	1,408,119	132,726	IAC Imperador	Middle	Andean	100	98	92	-92
Chr01	564,239	686,313	122,074	Rosinha G2	Middle	Andean	50	47	47	-47
Chr01	48,216,596	48,323,067	106,471	Rosinha G2	Middle	Andean	100	85	85	-85
Chr01	6,756,947	6,853,880	96,933	IPA 9	Middle	Andean	50	49	31	-31
Chr01	6,756,947	6,853,880	96,933	US Pinto 111	Middle	Andean	50	49	39	-39
Chr01	6,756,947	6,853,880	96,933	México 54	Middle	Andean	50	46	36	-36
Chr01	48,638,821	48,729,918	91,097	Rosinha G2	Middle	Andean	150	133	133	-133
Chr01	48,998,389	49,088,313	89,924	IAC Imperador	Middle	Andean	50	49	33	-33
Chr01	48,998,389	49,088,313	89,924	ESPLENDOR	Middle	Andean	50	50	34	-34
Chr01	48,998,389	49,088,313	89,924	Ouro Negro	Middle	Andean	50	50	35	-35
Chr01	7,674,934	7,764,814	89,880	ESPLENDOR	Middle	Andean	50	49	35	-35
Chr01	655	87,877	87,222	IAC Imperador	Middle	Andean	50	48	30	-30
Chr01	655	87,877	87,222	ESPLENDOR	Middle	Andean	50	50	30	-30
Chr01	655	87,877	87,222	FC104	Middle	Andean	50	50	30	-30
Chr01	4,431,211	4,505,425	74,214	TO	Middle	Andean	50	46	40	-40
Chr01	4,431,211	4,505,425	74,214	IPA 9	Middle	Andean	50	49	33	-33
Chr01	4,431,211	4,505,425	74,214	Sel 1308	Middle	Andean	50	50	34	-34
Chr01	4,431,211	4,505,425	74,214	PI 207262	Middle	Andean	50	50	34	-34
Chr01	4,431,211	4,505,425	74,214	G2333	Middle	Andean	50	50	33	-33
Chr01	4,431,211	4,505,425	74,214	FC104	Middle	Andean	50	50	34	-34
Chr01	4,431,211	4,505,425	74,214	BRS Sublime	Middle	Andean	50	47	31	-31
Chr01	4,431,211	4,505,425	74,214	MAIII 16159 (UFLA)	Middle	Andean	50	48	33	-33
Chr01	4,431,211	4,505,425	74,214	CNFC 10467	Middle	Andean	50	47	31	-31
Chr01	1,024,728	1,065,036	40,308	PI 207262	Middle	Andean	50	49	33	-33
Chr01	50,431,286	50,465,673	34,387	Rosinha G2	Middle	Andean	50	48	48	-48
Chr01	48,798,598	48,830,994	32,396	ESPLENDOR	Middle	Andean	50	50	30	-30
Chr01	48,798,598	48,830,994	32,396	Ouro Negro	Middle	Andean	50	49	30	-30
Chr01	6,680,298	6,698,738	18,440	ESPLENDOR	Middle	Andean	50	50	40	-40
Chr01	6,680,298	6,698,738	18,440	IPA 9	Middle	Andean	50	48	34	-34
Chr01	6,680,298	6,698,738	18,440	US Pinto 111	Middle	Andean	50	48	38	-38
Chr01	6,680,298	6,698,738	18,440	México 54	Middle	Andean	50	48	38	-38
Chr02	40,460,267	43,136,720	2,676,453	MAIII 16159 (UFLA)	Middle	Andean	1000	935	926	-926
Chr02	47,560,378	48,243,917	683,539	OURO BRANCO	Andean	Middle	450	407	-383	383
Chr02	48,967,056	49,454,704	487,648	BRS Ártico (WAF 75)	Andean	Middle	50	49	-48	48
Chr02	48,967,056	49,454,704	487,648	Rosinha G2	Middle	Andean	50	45	32	-32
Chr02	1,871,509	2,239,606	368,097	AND277	Andean	Middle	1050	1050	-978	978
Chr02	481,619	848,122	366,503	BRS Embaixador	Andean	Middle	950	934	-914	914

Chr	Start	End	Length	Sample	Background group	Haplotype group	Total variants	Genotyped variants	Score Andean	Score Middle
Chr02	28,865,566	29,120,405	254,839	México 54	Middle	Andean	100	98	81	-81
Chr02	510,129	722,594	212,465	AND277	Andean	Middle	800	800	-800	800
Chr02	43,914,483	44,112,431	197,948	MAIII 16159 (UFLA)	Middle	Andean	150	144	136	-136
Chr02	48,542,553	48,718,459	175,906	BRS Embaixador	Andean	Middle	650	639	-614	614
Chr02	48,542,553	48,718,459	175,906	BRS FS305 (CAL 96)	Andean	Middle	650	648	-624	624
Chr02	10,812,840	10,934,582	121,742	TO	Middle	Andean	100	93	83	-83
Chr02	10,812,840	10,934,582	121,742	ESTILO	Middle	Andean	100	100	92	-92
Chr02	10,812,840	10,934,582	121,742	BRS Supremo	Middle	Andean	100	94	85	-85
Chr02	10,812,840	10,934,582	121,742	RUDA	Middle	Andean	100	100	92	-92
Chr02	10,812,840	10,934,582	121,742	PEROLA	Middle	Andean	100	100	92	-92
Chr02	10,812,840	10,934,582	121,742	IAC Diplomata	Middle	Andean	100	100	92	-92
Chr02	10,812,840	10,934,582	121,742	CNFC 10467	Middle	Andean	100	98	90	-90
Chr02	10,812,840	10,934,582	121,742	CB 911921	Middle	Andean	100	99	90	-90
Chr02	10,812,840	10,934,582	121,742	BRS Ametista	Middle	Andean	100	94	83	-83
Chr02	29,009,986	29,120,405	110,419	US Pinto 111	Middle	Andean	50	48	33	-33
Chr02	10,246	110,183	99,937	BRS Embaixador	Andean	Middle	50	49	-45	45
Chr02	29,330,987	29,429,376	98,389	PI 207262	Middle	Andean	50	48	30	-30
Chr02	29,330,987	29,429,376	98,389	BRS Madrepérola	Middle	Andean	50	50	32	-32
Chr02	29,330,987	29,429,376	98,389	México 54	Middle	Andean	50	46	30	-30
Chr02	29,330,987	29,429,376	98,389	ESTEIO	Middle	Andean	50	50	32	-32
Chr02	29,330,987	29,429,376	98,389	G2333	Middle	Andean	50	47	33	-33
Chr02	29,330,987	29,429,376	98,389	MAIII 16159 (UFLA)	Middle	Andean	50	49	30	-30
Chr02	29,330,987	29,429,376	98,389	CNFC 10794	Middle	Andean	50	50	32	-32
Chr02	3,256,244	3,344,844	88,600	US Pinto 111	Middle	Andean	50	50	36	-36
Chr02	3,256,244	3,344,844	88,600	México 54	Middle	Andean	50	48	34	-34
Chr02	3,256,244	3,344,844	88,600	G2333	Middle	Andean	50	50	36	-36
Chr02	3,256,244	3,344,844	88,600	ESTILO	Middle	Andean	50	50	36	-36
Chr02	3,256,244	3,344,844	88,600	CNFC 10794	Middle	Andean	50	50	36	-36
Chr02	45,308,386	45,386,596	78,210	IPA 9	Middle	Andean	100	99	74	-74
Chr02	45,308,386	45,386,596	78,210	ESTILO	Middle	Andean	100	100	76	-76
Chr02	45,308,386	45,386,596	78,210	PEROLA	Middle	Andean	100	100	76	-76
Chr02	45,308,386	45,386,596	78,210	CNFC 10794	Middle	Andean	100	98	74	-74
Chr02	45,308,386	45,386,596	78,210	BRS Ametista	Middle	Andean	100	97	75	-75
Chr02	33,514,108	33,589,454	75,346	Rosinha G2	Middle	Andean	50	40	30	-30
Chr02	33,514,108	33,589,454	75,346	PI 207262	Middle	Andean	50	50	32	-32
Chr02	33,514,108	33,589,454	75,346	BRS Madrepérola	Middle	Andean	50	47	31	-31
Chr02	33,514,108	33,589,454	75,346	US Pinto 111	Middle	Andean	50	49	30	-30
Chr02	33,514,108	33,589,454	75,346	México 54	Middle	Andean	50	47	31	-31
Chr02	33,514,108	33,589,454	75,346	ESTEIO	Middle	Andean	50	50	32	-32
Chr02	33,514,108	33,589,454	75,346	MAIII 16159 (UFLA)	Middle	Andean	50	49	31	-31
Chr02	33,514,108	33,589,454	75,346	CNFC 10794	Middle	Andean	50	48	30	-30
Chr02	37,709,785	37,775,158	65,373	TO	Middle	Andean	50	47	37	-37
Chr02	37,709,785	37,775,158	65,373	IPA 9	Middle	Andean	50	50	40	-40
Chr02	37,709,785	37,775,158	65,373	Sel 1308	Middle	Andean	50	47	37	-37
Chr02	37,709,785	37,775,158	65,373	PI 207262	Middle	Andean	50	49	39	-39
Chr02	37,709,785	37,775,158	65,373	BRS Sublime	Middle	Andean	50	49	39	-39
Chr02	37,709,785	37,775,158	65,373	PEROLA	Middle	Andean	50	50	40	-40
Chr02	37,709,785	37,775,158	65,373	Bat 332	Middle	Andean	50	49	37	-37
Chr02	37,709,785	37,775,158	65,373	Ouro Negro	Middle	Andean	50	50	40	-40
Chr02	37,709,785	37,775,158	65,373	CNFCT 19120	Middle	Andean	50	40	30	-30
Chr02	37,709,785	37,775,158	65,373	CB 911921	Middle	Andean	50	46	36	-36
Chr02	45,464,661	45,523,907	59,246	IPA 9	Middle	Andean	50	49	33	-33
Chr02	45,464,661	45,523,907	59,246	Sel 1308	Middle	Andean	50	50	31	-31
Chr02	45,464,661	45,523,907	59,246	ESTILO	Middle	Andean	50	50	32	-32
Chr02	45,464,661	45,523,907	59,246	BRS Supremo	Middle	Andean	50	49	31	-31
Chr02	45,464,661	45,523,907	59,246	PEROLA	Middle	Andean	50	50	32	-32
Chr02	45,464,661	45,523,907	59,246	CNFC 10794	Middle	Andean	50	50	32	-32
Chr02	45,464,661	45,523,907	59,246	CB 911921	Middle	Andean	50	48	32	-32
Chr02	45,464,661	45,523,907	59,246	BRS Cometa	Middle	Andean	50	48	30	-30
Chr02	45,464,661	45,523,907	59,246	BRS Ametista	Middle	Andean	50	50	32	-32
Chr02	29,121,595	29,180,721	59,126	BRS Madrepérola	Middle	Andean	50	50	30	-30
Chr02	29,121,595	29,180,721	59,126	ESTEIO	Middle	Andean	50	50	30	-30
Chr02	46,676,940	46,730,424	53,484	TO	Middle	Andean	50	41	31	-31
Chr02	46,676,940	46,730,424	53,484	ESPLENDOR	Middle	Andean	50	50	34	-34
Chr02	46,676,940	46,730,424	53,484	MAIII 16159 (UFLA)	Middle	Andean	50	45	33	-33
Chr02	46,676,940	46,730,424	53,484	IAC Diplomata	Middle	Andean	50	50	34	-34
Chr02	46,676,940	46,730,424	53,484	CNFC 10467	Middle	Andean	50	47	35	-35
Chr02	48,667,886	48,718,459	50,573	Rosinha G2	Middle	Andean	100	85	84	-84
Chr02	47,210,694	47,259,106	48,412	OURO BRANCO	Andean	Middle	50	45	-35	35
Chr02	37,148,129	37,195,631	47,502	BRS FS305 (CAL 96)	Andean	Middle	50	50	-30	30
Chr02	37,148,129	37,195,631	47,502	OURO BRANCO	Andean	Middle	50	46	-32	32
Chr02	31,779,612	31,823,466	43,854	Jalo Precoce	Andean	Middle	50	50	-33	33
Chr02	31,779,612	31,823,466	43,854	BRSMG Realce	Andean	Middle	50	48	-32	32
Chr02	27,915,626	27,956,870	41,244	PI 207262	Middle	Andean	50	48	32	-32
Chr02	27,915,626	27,956,870	41,244	BRS Madrepérola	Middle	Andean	50	47	31	-31
Chr02	27,915,626	27,956,870	41,244	ESTEIO	Middle	Andean	50	49	31	-31

Chr	Start	End	Length	Sample	Background group	Haplotype group	Total variants	Genotyped variants	Score Andean	Score Middle
Chr02	27,915,626	27,956,870	41,244	CNFC 10794	Middle	Andean	50	47	32	-32
Chr02	46,512,615	46,550,600	37,985	BRS Executivo	Andean	Middle	50	50	-34	34
Chr02	46,512,615	46,550,600	37,985	BRS FS305 (CAL 96)	Andean	Middle	50	50	-34	34
Chr02	46,512,615	46,550,600	37,985	AND277	Andean	Middle	50	50	-34	34
Chr02	1,740,031	1,777,040	37,009	AND277	Andean	Middle	50	50	-36	36
Chr02	45,724,565	45,757,229	32,664	Sel 1308	Middle	Andean	50	49	31	-31
Chr02	45,724,565	45,757,229	32,664	US Pinto 111	Middle	Andean	50	44	34	-34
Chr02	45,724,565	45,757,229	32,664	ESTILO	Middle	Andean	50	50	32	-32
Chr02	45,724,565	45,757,229	32,664	RUDA	Middle	Andean	50	50	32	-32
Chr02	45,724,565	45,757,229	32,664	PEROLA	Middle	Andean	50	50	32	-32
Chr02	45,724,565	45,757,229	32,664	CNFC 10794	Middle	Andean	50	48	32	-32
Chr02	45,354,513	45,386,596	32,083	Sel 1308	Middle	Andean	50	48	44	-44
Chr02	45,354,513	45,386,596	32,083	BRS Supremo	Middle	Andean	50	46	42	-42
Chr02	45,354,513	45,386,596	32,083	CB 911921	Middle	Andean	50	48	44	-44
Chr02	1,387,942	1,418,532	30,590	Sel 1308	Middle	Andean	50	48	47	-47
Chr02	1,387,942	1,418,532	30,590	US Pinto 111	Middle	Andean	50	47	43	-43
Chr02	1,387,942	1,418,532	30,590	FC104	Middle	Andean	50	50	48	-48
Chr02	1,387,942	1,418,532	30,590	CNFCT 19120	Middle	Andean	50	40	37	-37
Chr02	44,898,857	44,928,844	29,987	ESPLENDOR	Middle	Andean	50	50	30	-30
Chr02	44,898,857	44,928,844	29,987	Rosinha G2	Middle	Andean	50	42	32	-32
Chr02	44,898,857	44,928,844	29,987	ESTEIO	Middle	Andean	50	50	30	-30
Chr02	44,898,857	44,928,844	29,987	RUDA	Middle	Andean	50	50	30	-30
Chr02	44,898,857	44,928,844	29,987	IAC Diplomata	Middle	Andean	50	50	30	-30
Chr02	44,898,857	44,928,844	29,987	CNFC 10467	Middle	Andean	50	49	30	-30
Chr02	1,806,212	1,833,933	27,721	AND277	Andean	Middle	50	50	-38	38
Chr02	48,631,272	48,654,888	23,616	Rosinha G2	Middle	Andean	100	89	88	-88
Chr02	46,464,366	46,482,979	18,613	BRS Executivo	Andean	Middle	100	100	-81	81
Chr02	46,464,366	46,482,979	18,613	BRS FS305 (CAL 96)	Andean	Middle	100	100	-80	80
Chr02	46,464,366	46,482,979	18,613	AND277	Andean	Middle	100	100	-80	80
Chr02	1,959,539	1,972,248	12,709	México 54	Middle	Andean	50	40	36	-36
Chr02	1,959,539	1,972,248	12,709	FC104	Middle	Andean	50	50	46	-46
Chr02	1,959,539	1,972,248	12,709	Bat 332	Middle	Andean	50	49	45	-45
Chr02	1,959,539	1,972,248	12,709	NOTAVEL	Middle	Andean	50	50	46	-46
Chr02	48,606,397	48,616,161	9,764	Rosinha G2	Middle	Andean	50	40	40	-40
Chr02	48,593,624	48,601,081	7,457	Rosinha G2	Middle	Andean	50	46	46	-46
Chr02	563,680	568,397	4,717	PI 207262	Middle	Andean	50	46	30	-30
Chr02	563,680	568,397	4,717	MAIII 16159 (UFLA)	Middle	Andean	50	49	33	-33
Chr03	10,766,072	34,551,727	23,785,655	TO	Middle	Andean	2800	2543	2509	-2509
Chr03	37,346,162	43,098,035	5,751,873	TO	Middle	Andean	2200	2012	1984	-1984
Chr03	8,117,228	10,570,521	2,453,293	TO	Middle	Andean	750	709	704	-704
Chr03	5,562,334	8,003,203	2,440,869	TO	Middle	Andean	1350	1253	1236	-1236
Chr03	35,179,017	37,301,685	2,122,668	TO	Middle	Andean	900	812	807	-807
Chr03	10,763	1,350,370	1,339,607	CB 911921	Middle	Andean	400	383	383	-383
Chr03	4,110,751	5,306,499	1,195,748	TO	Middle	Andean	650	594	592	-592
Chr03	10,763	1,018,537	1,007,774	BRS Horizonte	Middle	Andean	250	230	219	-219
Chr03	10,763	1,018,537	1,007,774	ESTEIO	Middle	Andean	250	250	240	-240
Chr03	10,763	1,018,537	1,007,774	FC104	Middle	Andean	250	250	240	-240
Chr03	10,763	1,018,537	1,007,774	BRS Sublime	Middle	Andean	250	235	224	-224
Chr03	10,763	1,018,537	1,007,774	RUDA	Middle	Andean	250	250	240	-240
Chr03	10,763	1,018,537	1,007,774	IAC Diplomata	Middle	Andean	250	250	240	-240
Chr03	10,763	1,018,537	1,007,774	CNFC 10467	Middle	Andean	250	243	233	-233
Chr03	10,763	1,018,537	1,007,774	BRS Cometa	Middle	Andean	250	221	208	-208
Chr03	10,763	1,018,537	1,007,774	BRS Ametista	Middle	Andean	250	235	224	-224
Chr03	2,155,060	3,021,521	866,461	CB 911921	Middle	Andean	450	434	430	-430
Chr03	10,763	818,114	807,351	CNFCT 19120	Middle	Andean	100	86	81	-81
Chr03	52,527,211	53,050,320	523,109	MAIII 16159 (UFLA)	Middle	Andean	350	329	316	-316
Chr03	51,881,494	52,398,267	516,773	MAIII 16159 (UFLA)	Middle	Andean	300	290	272	-272
Chr03	25,144,968	25,622,462	477,494	México 54	Middle	Andean	50	46	36	-36
Chr03	1,600,272	2,049,436	449,164	TO	Middle	Andean	300	274	274	-274
Chr03	42,906,123	43,335,612	429,489	PEROLA	Middle	Andean	400	400	385	-385
Chr03	34,551,753	34,899,424	347,671	CNFC 16207	Middle	Andean	50	42	34	-34
Chr03	10,763	243,620	232,857	Rosinha G2	Middle	Andean	50	41	41	-41
Chr03	48,520,681	48,733,267	212,586	ESPLENDOR	Middle	Andean	150	150	102	-102
Chr03	48,520,681	48,733,267	212,586	IPA 9	Middle	Andean	150	149	100	-100
Chr03	48,520,681	48,733,267	212,586	BRS Madrepérola	Middle	Andean	150	146	99	-99
Chr03	48,520,681	48,733,267	212,586	ESTEIO	Middle	Andean	150	150	102	-102
Chr03	38,972,597	39,158,200	185,603	US Pinto 111	Middle	Andean	150	146	124	-124
Chr03	38,972,597	39,158,200	185,603	G2333	Middle	Andean	150	147	121	-121
Chr03	30,826,076	31,010,004	183,928	México 54	Middle	Andean	50	50	30	-30
Chr03	30,826,076	31,010,004	183,928	Ouro Negro	Middle	Andean	50	49	31	-31
Chr03	3,837,347	4,020,394	183,047	TO	Middle	Andean	150	146	145	-145
Chr03	45,937,540	46,103,369	165,829	IPA 9	Middle	Andean	150	147	123	-123
Chr03	45,937,540	46,103,369	165,829	PI 207262	Middle	Andean	150	142	118	-118
Chr03	45,937,540	46,103,369	165,829	BRS Madrepérola	Middle	Andean	150	149	125	-125
Chr03	45,937,540	46,103,369	165,829	US Pinto 111	Middle	Andean	150	146	127	-127
Chr03	45,937,540	46,103,369	165,829	ESTEIO	Middle	Andean	150	150	126	-126

Chr	Start	End	Length	Sample	Background group	Haplotype group	Total variants	Genotyped variants	Score Andean	Score Middle
Chr03	45,937,540	46,103,369	165,829	BRS Ametista	Middle	Andean	150	148	123	-123
Chr03	23,997,236	24,151,017	153,781	México 54	Middle	Andean	50	47	39	-39
Chr03	7,129,992	7,278,033	148,041	G2333	Middle	Andean	100	99	65	-65
Chr03	39,018,530	39,158,200	139,670	PEROLA	Middle	Andean	100	100	76	-76
Chr03	39,018,530	39,158,200	139,670	CB 911921	Middle	Andean	100	97	75	-75
Chr03	37,301,908	37,424,162	122,254	ESPLENDOR	Middle	Andean	100	100	68	-68
Chr03	37,301,908	37,424,162	122,254	IPA 9	Middle	Andean	100	100	68	-68
Chr03	33,786,338	33,901,866	115,528	PI 207262	Middle	Andean	50	49	43	-43
Chr03	48,628,699	48,733,267	104,568	México 54	Middle	Andean	50	46	34	-34
Chr03	33,529,481	33,634,018	104,537	BRS Madrepérola	Middle	Andean	50	48	34	-34
Chr03	33,529,481	33,634,018	104,537	ESTEIO	Middle	Andean	50	50	33	-33
Chr03	33,529,481	33,634,018	104,537	FC104	Middle	Andean	50	50	33	-33
Chr03	33,529,481	33,634,018	104,537	BRS Sublime	Middle	Andean	50	48	30	-30
Chr03	33,529,481	33,634,018	104,537	RUDA	Middle	Andean	50	50	33	-33
Chr03	33,529,481	33,634,018	104,537	NOTAVEL	Middle	Andean	50	50	33	-33
Chr03	33,529,481	33,634,018	104,537	IAC Diplomata	Middle	Andean	50	50	33	-33
Chr03	33,529,481	33,634,018	104,537	CNFC 10467	Middle	Andean	50	49	34	-34
Chr03	33,529,481	33,634,018	104,537	BRS Cometa	Middle	Andean	50	45	31	-31
Chr03	33,529,481	33,634,018	104,537	BRS Ametista	Middle	Andean	50	49	34	-34
Chr03	32,913,323	33,011,662	98,339	Sel 1308	Middle	Andean	100	98	78	-78
Chr03	32,913,323	33,011,662	98,339	Ouro Negro	Middle	Andean	100	95	71	-71
Chr03	50,886,526	50,975,707	89,181	ESPLENDOR	Middle	Andean	50	50	38	-38
Chr03	50,886,526	50,975,707	89,181	IPA 9	Middle	Andean	50	48	36	-36
Chr03	50,886,526	50,975,707	89,181	BRS Madrepérola	Middle	Andean	50	50	38	-38
Chr03	50,886,526	50,975,707	89,181	ESTEIO	Middle	Andean	50	50	38	-38
Chr03	50,886,526	50,975,707	89,181	ESTILO	Middle	Andean	50	50	38	-38
Chr03	50,886,526	50,975,707	89,181	CNFC 10794	Middle	Andean	50	50	37	-37
Chr03	8,117,228	8,205,851	88,623	IPA 9	Middle	Andean	50	45	30	-30
Chr03	8,117,228	8,205,851	88,623	PI 207262	Middle	Andean	50	43	33	-33
Chr03	8,117,228	8,205,851	88,623	México 54	Middle	Andean	50	42	30	-30
Chr03	8,117,228	8,205,851	88,623	G2333	Middle	Andean	50	46	40	-40
Chr03	34,045,472	34,132,438	86,966	PI 207262	Middle	Andean	50	44	40	-40
Chr03	40,327,417	40,413,582	86,165	G2333	Middle	Andean	50	50	30	-30
Chr03	30,020,644	30,103,903	83,259	ESPLENDOR	Middle	Andean	50	50	30	-30
Chr03	30,020,644	30,103,903	83,259	IPA 9	Middle	Andean	50	48	30	-30
Chr03	37,346,162	37,424,162	78,000	PI 207262	Middle	Andean	50	48	30	-30
Chr03	12,159,587	12,235,012	75,425	Sel 1308	Middle	Andean	50	50	50	-50
Chr03	12,159,587	12,235,012	75,425	PI 207262	Middle	Andean	50	50	50	-50
Chr03	12,159,587	12,235,012	75,425	BRS Horizonte	Middle	Andean	50	45	45	-45
Chr03	12,159,587	12,235,012	75,425	PEROLA	Middle	Andean	50	50	50	-50
Chr03	12,159,587	12,235,012	75,425	Bat 332	Middle	Andean	50	50	49	-49
Chr03	12,159,587	12,235,012	75,425	Ouro Negro	Middle	Andean	50	49	49	-49
Chr03	12,159,587	12,235,012	75,425	MAIII 16159 (UFLA)	Middle	Andean	50	50	47	-47
Chr03	12,159,587	12,235,012	75,425	CB 911921	Middle	Andean	50	49	48	-48
Chr03	49,482,417	49,556,470	74,053	ESTILO	Middle	Andean	50	50	36	-36
Chr03	49,482,417	49,556,470	74,053	BRS Sublime	Middle	Andean	50	46	30	-30
Chr03	49,482,417	49,556,470	74,053	PEROLA	Middle	Andean	50	50	36	-36
Chr03	49,482,417	49,556,470	74,053	Bat 332	Middle	Andean	50	49	35	-35
Chr03	49,482,417	49,556,470	74,053	NOTAVEL	Middle	Andean	50	50	36	-36
Chr03	49,482,417	49,556,470	74,053	CNFC 10794	Middle	Andean	50	47	35	-35
Chr03	36,638,976	36,711,202	72,226	PI 207262	Middle	Andean	50	50	33	-33
Chr03	6,806,828	6,866,255	59,427	IAC Imperador	Middle	Andean	100	95	73	-73
Chr03	6,806,828	6,866,255	59,427	IPA 9	Middle	Andean	100	100	81	-81
Chr03	6,806,828	6,866,255	59,427	PI 207262	Middle	Andean	100	100	83	-83
Chr03	6,806,828	6,866,255	59,427	ESTILO	Middle	Andean	100	100	82	-82
Chr03	6,806,828	6,866,255	59,427	NOTAVEL	Middle	Andean	100	100	82	-82
Chr03	38,567,946	38,623,341	55,395	ESPLENDOR	Middle	Andean	50	50	34	-34
Chr03	38,567,946	38,623,341	55,395	IPA 9	Middle	Andean	50	50	34	-34
Chr03	39,103,597	39,158,200	54,603	ESPLENDOR	Middle	Andean	50	50	36	-36
Chr03	39,103,597	39,158,200	54,603	IPA 9	Middle	Andean	50	50	35	-35
Chr03	39,103,597	39,158,200	54,603	México 54	Middle	Andean	50	49	33	-33
Chr03	818,256	872,430	54,174	Rosinha G2	Middle	Andean	50	44	43	-43
Chr03	7,224,226	7,278,033	53,807	IAC Imperador	Middle	Andean	50	49	37	-37
Chr03	7,224,226	7,278,033	53,807	IPA 9	Middle	Andean	50	50	38	-38
Chr03	7,224,226	7,278,033	53,807	PI 207262	Middle	Andean	50	50	38	-38
Chr03	7,224,226	7,278,033	53,807	US Pinto 111	Middle	Andean	50	47	37	-37
Chr03	7,224,226	7,278,033	53,807	México 54	Middle	Andean	50	50	38	-38
Chr03	7,224,226	7,278,033	53,807	ESTILO	Middle	Andean	50	50	38	-38
Chr03	7,224,226	7,278,033	53,807	BRS Supremo	Middle	Andean	50	48	36	-36
Chr03	7,224,226	7,278,033	53,807	NOTAVEL	Middle	Andean	50	50	38	-38
Chr03	7,224,226	7,278,033	53,807	CNFC 16207	Middle	Andean	50	47	35	-35
Chr03	7,224,226	7,278,033	53,807	CNFC 10794	Middle	Andean	50	47	37	-37
Chr03	48,520,681	48,569,478	48,797	TO	Middle	Andean	50	49	34	-34
Chr03	37,301,908	37,346,161	44,253	US Pinto 111	Middle	Andean	50	44	34	-34
Chr03	37,301,908	37,346,161	44,253	G2333	Middle	Andean	50	49	37	-37
Chr03	52,778,536	52,822,246	43,710	PI 207262	Middle	Andean	50	49	37	-37

Chr	Start	End	Length	Sample	Background group	Haplotype group	Total variants	Genotyped variants	Score Andean	Score Middle
Chr03	52,778,536	52,822,246	43,710	US Pinto 111	Middle	Andean	50	49	35	-35
Chr03	52,778,536	52,822,246	43,710	México 54	Middle	Andean	50	49	35	-35
Chr03	52,778,536	52,822,246	43,710	G2333	Middle	Andean	50	46	32	-32
Chr03	52,915,284	52,958,174	42,890	US Pinto 111	Middle	Andean	50	44	30	-30
Chr03	52,915,284	52,958,174	42,890	G2333	Middle	Andean	50	50	31	-31
Chr03	32,871,940	32,913,021	41,081	IAC Imperador	Middle	Andean	50	50	32	-32
Chr03	32,871,940	32,913,021	41,081	ESTILO	Middle	Andean	50	50	32	-32
Chr03	32,871,940	32,913,021	41,081	BRS Supremo	Middle	Andean	50	46	31	-31
Chr03	32,871,940	32,913,021	41,081	CNFC 10794	Middle	Andean	50	50	32	-32
Chr03	3,639,871	3,679,966	40,095	TO	Middle	Andean	50	47	36	-36
Chr03	3,639,871	3,679,966	40,095	PI 207262	Middle	Andean	50	50	40	-40
Chr03	3,639,871	3,679,966	40,095	BRS Horizonte	Middle	Andean	50	46	36	-36
Chr03	3,639,871	3,679,966	40,095	US Pinto 111	Middle	Andean	50	41	33	-33
Chr03	3,639,871	3,679,966	40,095	México 54	Middle	Andean	50	43	33	-33
Chr03	3,639,871	3,679,966	40,095	G2333	Middle	Andean	50	50	40	-40
Chr03	3,639,871	3,679,966	40,095	PEROLA	Middle	Andean	50	50	40	-40
Chr03	3,639,871	3,679,966	40,095	Ouro Negro	Middle	Andean	50	49	35	-35
Chr03	3,639,871	3,679,966	40,095	NOTAVEL	Middle	Andean	50	50	40	-40
Chr03	3,639,871	3,679,966	40,095	CB 911921	Middle	Andean	50	48	37	-37
Chr03	50,073,867	50,112,502	38,635	ESPLENDOR	Middle	Andean	50	48	40	-40
Chr03	50,073,867	50,112,502	38,635	IPA 9	Middle	Andean	50	47	39	-39
Chr03	50,073,867	50,112,502	38,635	BRS Madrepérola	Middle	Andean	50	48	40	-40
Chr03	50,073,867	50,112,502	38,635	México 54	Middle	Andean	50	47	39	-39
Chr03	50,073,867	50,112,502	38,635	ESTEIO	Middle	Andean	50	48	40	-40
Chr03	51,881,494	51,918,214	36,720	ESPLENDOR	Middle	Andean	50	50	40	-40
Chr03	51,881,494	51,918,214	36,720	IPA 9	Middle	Andean	50	48	38	-38
Chr03	51,881,494	51,918,214	36,720	ESTILO	Middle	Andean	50	50	40	-40
Chr03	51,881,494	51,918,214	36,720	CNFC 10794	Middle	Andean	50	49	37	-37
Chr03	46,022,323	46,054,271	31,948	México 54	Middle	Andean	50	47	41	-41
Chr03	46,022,323	46,054,271	31,948	G2333	Middle	Andean	50	48	42	-42
Chr03	32,990,568	33,011,662	21,094	México 54	Middle	Andean	50	40	32	-32
Chr03	32,990,568	33,011,662	21,094	PEROLA	Middle	Andean	50	50	42	-42
Chr03	32,990,568	33,011,662	21,094	CB 911921	Middle	Andean	50	46	38	-38
Chr03	4,967,075	4,987,403	20,328	IPA 9	Middle	Andean	50	48	36	-36
Chr03	4,967,075	4,987,403	20,328	PI 207262	Middle	Andean	50	50	37	-37
Chr03	4,967,075	4,987,403	20,328	US Pinto 111	Middle	Andean	50	47	37	-37
Chr03	4,967,075	4,987,403	20,328	PEROLA	Middle	Andean	50	50	38	-38
Chr03	4,967,075	4,987,403	20,328	Ouro Negro	Middle	Andean	50	50	38	-38
Chr03	4,967,075	4,987,403	20,328	CB 911921	Middle	Andean	50	50	38	-38
Chr03	6,852,019	6,866,255	14,236	US Pinto 111	Middle	Andean	50	50	50	-50
Chr03	6,852,019	6,866,255	14,236	México 54	Middle	Andean	50	46	46	-46
Chr03	6,852,019	6,866,255	14,236	BRS Supremo	Middle	Andean	50	50	50	-50
Chr03	6,852,019	6,866,255	14,236	UIRAPURU	Middle	Andean	50	50	39	-39
Chr03	6,852,019	6,866,255	14,236	CNFC 16207	Middle	Andean	50	47	47	-47
Chr03	6,852,019	6,866,255	14,236	CNFC 10794	Middle	Andean	50	49	49	-49
Chr03	51,731,197	51,745,284	14,087	Sel 1308	Middle	Andean	50	50	33	-33
Chr03	51,731,197	51,745,284	14,087	PEROLA	Middle	Andean	50	50	34	-34
Chr03	51,731,197	51,745,284	14,087	CB 911921	Middle	Andean	50	49	33	-33
Chr04	18,521,145	31,651,392	13,130,247	OURO BRANCO	Andean	Middle	600	536	-511	511
Chr04	33,687,761	36,986,762	3,299,001	OURO BRANCO	Andean	Middle	400	344	-341	341
Chr04	14,168,110	16,945,833	2,777,723	OURO BRANCO	Andean	Middle	150	140	-134	134
Chr04	32,217,468	33,455,181	1,237,713	OURO BRANCO	Andean	Middle	100	94	-93	93
Chr04	34,112,484	35,169,548	1,057,064	Sel 1308	Middle	Andean	150	148	107	-107
Chr04	34,112,484	35,169,548	1,057,064	Bat 332	Middle	Andean	150	149	108	-108
Chr04	34,112,484	35,169,548	1,057,064	CB 911921	Middle	Andean	150	146	104	-104
Chr04	34,112,484	34,769,645	657,161	IPA 9	Middle	Andean	100	99	73	-73
Chr04	34,112,484	34,769,645	657,161	ESTILO	Middle	Andean	100	100	73	-73
Chr04	34,112,484	34,769,645	657,161	CNFC 10794	Middle	Andean	100	97	74	-74
Chr04	46,592,529	47,200,018	607,489	CNFC 10794	Middle	Andean	150	145	114	-114
Chr04	46,679,044	47,200,018	520,974	US Pinto 111	Middle	Andean	100	92	78	-78
Chr04	46,679,044	47,200,018	520,974	G2333	Middle	Andean	100	99	83	-83
Chr04	34,112,484	34,581,284	468,800	México 54	Middle	Andean	50	45	34	-34
Chr04	46,746,615	47,200,018	453,403	IAC Imperador	Middle	Andean	50	47	33	-33
Chr04	45,136,031	45,560,006	423,975	BRS FS305 (CAL 96)	Andean	Middle	100	100	-90	90
Chr04	7,599,472	7,925,493	326,021	IPA 9	Middle	Andean	100	97	67	-67
Chr04	7,599,472	7,925,493	326,021	México 54	Middle	Andean	100	97	73	-73
Chr04	7,599,472	7,925,493	326,021	ESTILO	Middle	Andean	100	100	70	-70
Chr04	7,599,472	7,925,493	326,021	NOTAVEL	Middle	Andean	100	100	70	-70
Chr04	7,599,472	7,925,493	326,021	IAC Diplomata	Middle	Andean	100	100	70	-70
Chr04	7,599,472	7,925,493	326,021	CNFC 10794	Middle	Andean	100	96	68	-68
Chr04	43,891,501	44,200,241	308,740	CNFC 10794	Middle	Andean	100	95	76	-76
Chr04	33,470,247	33,687,748	217,501	Sel 1308	Middle	Andean	50	50	33	-33
Chr04	33,470,247	33,687,748	217,501	Bat 332	Middle	Andean	50	47	35	-35
Chr04	33,470,247	33,687,748	217,501	CB 911921	Middle	Andean	50	49	34	-34
Chr04	41,993,835	42,198,012	204,177	PI 207262	Middle	Andean	100	92	83	-83
Chr04	2,386,386	2,555,534	169,148	BRSMG Realce	Andean	Middle	50	45	-44	44

Chr	Start	End	Length	Sample	Background group	Haplotype group	Total variants	Genotyped variants	Score Andean	Score Middle
Chr04	4,464,575	4,621,770	157,195	MAIII 16159 (UFLA)	Middle	Andean	50	46	45	-45
Chr04	46,592,529	46,744,537	152,008	TO	Middle	Andean	100	86	75	-75
Chr04	46,592,529	46,744,537	152,008	PI 207262	Middle	Andean	100	98	80	-80
Chr04	46,592,529	46,744,537	152,008	México 54	Middle	Andean	100	91	79	-79
Chr04	46,592,529	46,744,537	152,008	ESTILO	Middle	Andean	100	100	88	-88
Chr04	40,565,961	40,710,961	145,000	ESTILO	Middle	Andean	50	50	44	-44
Chr04	40,565,961	40,710,961	145,000	CNFC 10794	Middle	Andean	50	49	43	-43
Chr04	2,777,985	2,902,976	124,991	Rosinha G2	Middle	Andean	200	179	172	-172
Chr04	2,777,985	2,902,976	124,991	RUDA	Middle	Andean	200	200	194	-194
Chr04	5,280,405	5,397,072	116,667	ESPLENDOR	Middle	Andean	50	50	36	-36
Chr04	5,280,405	5,397,072	116,667	IPA 9	Middle	Andean	50	46	33	-33
Chr04	5,280,405	5,397,072	116,667	Sel 1308	Middle	Andean	50	50	36	-36
Chr04	5,280,405	5,397,072	116,667	US Pinto 111	Middle	Andean	50	48	33	-33
Chr04	5,280,405	5,397,072	116,667	ESTILO	Middle	Andean	50	50	36	-36
Chr04	5,280,405	5,397,072	116,667	UIRAPURU	Middle	Andean	50	42	30	-30
Chr04	5,280,405	5,397,072	116,667	PEROLA	Middle	Andean	50	50	36	-36
Chr04	5,280,405	5,397,072	116,667	NOTAVEL	Middle	Andean	50	50	36	-36
Chr04	5,280,405	5,397,072	116,667	CNFC 10794	Middle	Andean	50	49	35	-35
Chr04	5,280,405	5,397,072	116,667	CB 911921	Middle	Andean	50	46	33	-33
Chr04	44,979,134	45,071,314	92,180	BRS FS305 (CAL 96)	Andean	Middle	50	50	-50	50
Chr04	46,679,044	46,744,537	65,493	BRS Ametista	Middle	Andean	50	48	42	-42
Chr04	41,993,835	42,052,725	58,890	ESPLENDOR	Middle	Andean	50	50	42	-42
Chr04	41,993,835	42,052,725	58,890	México 54	Middle	Andean	50	46	36	-36
Chr04	41,993,835	42,052,725	58,890	ESTILO	Middle	Andean	50	50	42	-42
Chr04	41,993,835	42,052,725	58,890	CNFC 16207	Middle	Andean	50	47	38	-38
Chr04	41,993,835	42,052,725	58,890	CNFC 10794	Middle	Andean	50	50	42	-42
Chr04	43,665,135	43,722,082	56,947	Sel 1308	Middle	Andean	50	48	32	-32
Chr04	43,665,135	43,722,082	56,947	PEROLA	Middle	Andean	50	50	30	-30
Chr04	45,078,005	45,125,324	47,319	US Pinto 111	Middle	Andean	50	50	36	-36
Chr04	45,078,005	45,125,324	47,319	Bat 332	Middle	Andean	50	49	34	-34
Chr04	45,078,005	45,125,324	47,319	IAC Diplomata	Middle	Andean	50	50	36	-36
Chr04	45,078,005	45,125,324	47,319	CNFC 10467	Middle	Andean	50	49	35	-35
Chr04	5,698,148	5,742,146	43,998	IPA 9	Middle	Andean	100	100	97	-97
Chr04	5,698,148	5,742,146	43,998	PI 207262	Middle	Andean	100	100	98	-98
Chr04	5,698,148	5,742,146	43,998	US Pinto 111	Middle	Andean	100	100	96	-96
Chr04	5,698,148	5,742,146	43,998	México 54	Middle	Andean	100	99	97	-97
Chr04	5,698,148	5,742,146	43,998	Ouro Negro	Middle	Andean	100	100	98	-98
Chr04	5,698,148	5,742,146	43,998	NOTAVEL	Middle	Andean	100	100	98	-98
Chr04	44,421,046	44,457,449	36,403	PI 207262	Middle	Andean	50	49	32	-32
Chr04	44,421,046	44,457,449	36,403	PEROLA	Middle	Andean	50	50	32	-32
Chr04	2,742,038	2,777,389	35,351	ESPLENDOR	Middle	Andean	50	50	32	-32
Chr04	2,742,038	2,777,389	35,351	IPA 9	Middle	Andean	50	49	31	-31
Chr04	2,742,038	2,777,389	35,351	NOTAVEL	Middle	Andean	50	50	32	-32
Chr04	2,742,038	2,777,389	35,351	IAC Diplomata	Middle	Andean	50	50	32	-32
Chr04	2,742,038	2,777,389	35,351	CNFC 10467	Middle	Andean	50	50	31	-31
Chr04	3,940,913	3,975,850	34,937	IPA 9	Middle	Andean	50	50	44	-44
Chr04	3,940,913	3,975,850	34,937	BRS Madrepérola	Middle	Andean	50	50	46	-46
Chr04	3,940,913	3,975,850	34,937	Ouro Negro	Middle	Andean	50	50	46	-46
Chr04	3,940,913	3,975,850	34,937	NOTAVEL	Middle	Andean	50	50	46	-46
Chr04	3,940,913	3,975,850	34,937	MAIII 16159 (UFLA)	Middle	Andean	50	49	45	-45
Chr04	3,940,913	3,975,850	34,937	IAC Diplomata	Middle	Andean	50	50	46	-46
Chr04	3,940,913	3,975,850	34,937	CNFC 16207	Middle	Andean	50	46	42	-42
Chr04	3,940,913	3,975,850	34,937	CNFC 10467	Middle	Andean	50	49	45	-45
Chr04	43,891,501	43,916,486	24,985	ESPLENDOR	Middle	Andean	50	50	48	-48
Chr04	43,891,501	43,916,486	24,985	ESTILO	Middle	Andean	50	50	48	-48
Chr04	43,891,501	43,916,486	24,985	CNFC 16207	Middle	Andean	50	50	47	-47
Chr05	34,335,816	35,698,816	1,363,000	BRS Executivo	Andean	Middle	300	295	-236	236
Chr05	34,335,816	35,698,816	1,363,000	BRS FS305 (CAL 96)	Andean	Middle	300	298	-237	237
Chr05	32,573,239	33,888,124	1,314,885	BRS Executivo	Andean	Middle	400	397	-372	372
Chr05	30,300,031	31,414,348	1,114,317	IPA 9	Middle	Andean	150	150	114	-114
Chr05	30,300,031	31,414,348	1,114,317	México 54	Middle	Andean	150	145	112	-112
Chr05	30,300,031	31,414,348	1,114,317	ESTILO	Middle	Andean	150	150	114	-114
Chr05	30,300,031	31,414,348	1,114,317	CNFC 10794	Middle	Andean	150	150	114	-114
Chr05	39,906,695	40,866,482	959,787	TO	Middle	Andean	750	696	692	-692
Chr05	30,300,031	31,232,929	932,898	PI 207262	Middle	Andean	100	99	83	-83
Chr05	30,300,031	31,232,929	932,898	BRS Madrepérola	Middle	Andean	100	97	81	-81
Chr05	30,300,031	31,232,929	932,898	US Pinto 111	Middle	Andean	100	92	76	-76
Chr05	30,300,031	31,232,929	932,898	G2333	Middle	Andean	100	96	80	-80
Chr05	33,093,943	33,888,124	794,181	BRS Embaixador	Andean	Middle	350	344	-318	318
Chr05	33,093,943	33,888,124	794,181	BRS FS305 (CAL 96)	Andean	Middle	350	347	-341	341
Chr05	36,192,972	36,972,854	779,882	BRS Executivo	Andean	Middle	450	448	-412	412
Chr05	36,192,972	36,972,854	779,882	BRS FS305 (CAL 96)	Andean	Middle	450	448	-416	416
Chr05	30,300,031	30,944,751	644,720	Rosinha G2	Middle	Andean	50	46	36	-36
Chr05	30,944,757	31,414,348	469,591	ESTEIO	Middle	Andean	100	100	71	-71
Chr05	907,531	1,308,694	401,163	BRS Executivo	Andean	Middle	150	147	-113	113
Chr05	907,531	1,308,694	401,163	Jalo Precoce	Andean	Middle	150	150	-136	136

Chr	Start	End	Length	Sample	Background group	Haplotype group	Total variants	Genotyped variants	Score Andean	Score Middle
Chr05	586,190	905,211	319,021	AND277	Andean	Middle	100	100	-83	83
Chr05	586,190	905,211	319,021	US Pinto 111	Middle	Andean	100	91	73	-73
Chr05	586,190	905,211	319,021	FC104	Middle	Andean	100	100	84	-84
Chr05	586,190	905,211	319,021	NOTAVEL	Middle	Andean	100	100	84	-84
Chr05	40,060,364	40,364,088	303,724	Rosinha G2	Middle	Andean	150	134	130	-130
Chr05	7,928	274,494	266,566	ESTEIO	Middle	Andean	200	200	184	-184
Chr05	36,192,972	36,453,680	260,708	AND277	Andean	Middle	50	50	-32	32
Chr05	586,190	831,591	245,401	BRSMG Realce	Andean	Middle	50	42	-33	33
Chr05	586,190	831,591	245,401	CB 911921	Middle	Andean	50	47	34	-34
Chr05	15,248,100	15,429,604	181,504	Sel 1308	Middle	Andean	50	49	42	-42
Chr05	15,248,100	15,429,604	181,504	BRS Supremo	Middle	Andean	50	47	41	-41
Chr05	15,248,100	15,429,604	181,504	Bat 332	Middle	Andean	50	49	42	-42
Chr05	15,248,100	15,429,604	181,504	NOTAVEL	Middle	Andean	50	50	45	-45
Chr05	15,248,100	15,429,604	181,504	CNFC 16207	Middle	Andean	50	44	38	-38
Chr05	15,248,100	15,429,604	181,504	BRS Cometa	Middle	Andean	50	46	41	-41
Chr05	32,415,336	32,572,012	156,676	IPA 9	Middle	Andean	100	99	81	-81
Chr05	32,415,336	32,572,012	156,676	PI 207262	Middle	Andean	100	95	76	-76
Chr05	32,415,336	32,572,012	156,676	BRS Madrepérola	Middle	Andean	100	98	82	-82
Chr05	32,415,336	32,572,012	156,676	ESTILO	Middle	Andean	100	100	82	-82
Chr05	32,415,336	32,572,012	156,676	CNFC 10794	Middle	Andean	100	98	80	-80
Chr05	1,443,752	1,571,566	127,814	PI 207262	Middle	Andean	50	46	30	-30
Chr05	40,455,336	40,573,545	118,209	Rosinha G2	Middle	Andean	100	85	85	-85
Chr05	5,518,453	5,624,106	105,653	Ouro Negro	Middle	Andean	100	100	82	-82
Chr05	38,360,165	38,447,467	87,302	ESPLENDOR	Middle	Andean	50	50	30	-30
Chr05	4,575,629	4,662,746	87,117	Sel 1308	Middle	Andean	100	94	72	-72
Chr05	4,575,629	4,662,746	87,117	BRS Supremo	Middle	Andean	100	95	72	-72
Chr05	4,575,629	4,662,746	87,117	PEROLA	Middle	Andean	100	99	75	-75
Chr05	4,426,250	4,506,067	79,817	Sel 1308	Middle	Andean	200	196	165	-165
Chr05	4,426,250	4,506,067	79,817	ESTILO	Middle	Andean	200	200	170	-170
Chr05	4,426,250	4,506,067	79,817	BRS Supremo	Middle	Andean	200	195	169	-169
Chr05	4,426,250	4,506,067	79,817	PEROLA	Middle	Andean	200	200	170	-170
Chr05	4,426,250	4,506,067	79,817	CNFC 16207	Middle	Andean	200	178	155	-155
Chr05	4,426,250	4,506,067	79,817	CNFC 10794	Middle	Andean	200	196	167	-167
Chr05	3,669,320	3,747,904	78,584	IAC Imperador	Middle	Andean	50	47	31	-31
Chr05	38,237,342	38,309,043	71,701	US Pinto 111	Middle	Andean	50	47	32	-32
Chr05	38,237,342	38,309,043	71,701	ESTILO	Middle	Andean	50	50	34	-34
Chr05	38,237,342	38,309,043	71,701	Ouro Negro	Middle	Andean	50	50	32	-32
Chr05	38,237,342	38,309,043	71,701	CNFC 10794	Middle	Andean	50	50	34	-34
Chr05	5,121,976	5,181,302	59,326	Sel 1308	Middle	Andean	100	99	80	-80
Chr05	5,121,976	5,181,302	59,326	BRS Supremo	Middle	Andean	100	90	74	-74
Chr05	5,121,976	5,181,302	59,326	PEROLA	Middle	Andean	100	100	81	-81
Chr05	5,121,976	5,181,302	59,326	CNFC 16207	Middle	Andean	100	92	77	-77
Chr05	40,060,364	40,106,717	46,353	US Pinto 111	Middle	Andean	50	41	31	-31
Chr05	40,060,364	40,106,717	46,353	G2333	Middle	Andean	50	49	39	-39
Chr05	40,060,364	40,106,717	46,353	PEROLA	Middle	Andean	50	50	40	-40
Chr05	40,060,364	40,106,717	46,353	CB 911921	Middle	Andean	50	48	37	-37
Chr05	1,661,530	1,705,982	44,452	PI 207262	Middle	Andean	50	50	43	-43
Chr05	1,661,530	1,705,982	44,452	US Pinto 111	Middle	Andean	50	47	39	-39
Chr05	1,661,530	1,705,982	44,452	México 54	Middle	Andean	50	50	42	-42
Chr05	1,661,530	1,705,982	44,452	G2333	Middle	Andean	50	50	42	-42
Chr05	1,661,530	1,705,982	44,452	FC104	Middle	Andean	50	50	42	-42
Chr05	1,661,530	1,705,982	44,452	PEROLA	Middle	Andean	50	50	42	-42
Chr05	1,661,530	1,705,982	44,452	NOTAVEL	Middle	Andean	50	50	42	-42
Chr05	1,661,530	1,705,982	44,452	MAIII 16159 (UFLA)	Middle	Andean	50	48	42	-42
Chr05	4,575,629	4,615,233	39,604	CNFC 16207	Middle	Andean	50	48	37	-37
Chr05	39,367,792	39,401,675	33,883	PEROLA	Middle	Andean	50	50	38	-38
Chr05	39,367,792	39,401,675	33,883	CB 911921	Middle	Andean	50	44	34	-34
Chr05	5,387,055	5,416,517	29,462	TO	Middle	Andean	50	44	44	-44
Chr05	5,387,055	5,416,517	29,462	ESPLENDOR	Middle	Andean	50	50	50	-50
Chr05	5,387,055	5,416,517	29,462	México 54	Middle	Andean	50	50	50	-50
Chr05	5,387,055	5,416,517	29,462	ESTEIO	Middle	Andean	50	50	50	-50
Chr05	5,387,055	5,416,517	29,462	Ouro Negro	Middle	Andean	50	50	50	-50
Chr05	5,387,055	5,416,517	29,462	MAIII 16159 (UFLA)	Middle	Andean	50	44	44	-44
Chr05	5,387,055	5,416,517	29,462	CB 911921	Middle	Andean	50	50	50	-50
Chr05	4,734,041	4,756,681	22,640	Bat 332	Middle	Andean	100	97	73	-73
Chr05	5,603,464	5,624,106	20,642	Sel 1308	Middle	Andean	50	50	44	-44
Chr05	5,603,464	5,624,106	20,642	BRS Supremo	Middle	Andean	50	49	43	-43
Chr05	5,603,464	5,624,106	20,642	PEROLA	Middle	Andean	50	50	44	-44
Chr05	5,603,464	5,624,106	20,642	CNFC 16207	Middle	Andean	50	47	41	-41
Chr05	1,745,071	1,765,486	20,415	IAC Imperador	Middle	Andean	50	50	50	-50
Chr05	1,745,071	1,765,486	20,415	IPA 9	Middle	Andean	50	50	50	-50
Chr05	1,745,071	1,765,486	20,415	PI 207262	Middle	Andean	50	50	50	-50
Chr05	1,745,071	1,765,486	20,415	US Pinto 111	Middle	Andean	50	49	49	-49
Chr05	1,745,071	1,765,486	20,415	México 54	Middle	Andean	50	50	49	-49
Chr05	1,745,071	1,765,486	20,415	G2333	Middle	Andean	50	50	50	-50
Chr05	1,745,071	1,765,486	20,415	FC104	Middle	Andean	50	50	50	-50

Chr	Start	End	Length	Sample	Background group	Haplotype group	Total variants	Genotyped variants	Score Andean	Score Middle
Chr05	1,745,071	1,765,486	20,415	PEROLA	Middle	Andean	50	50	50	-50
Chr05	1,745,071	1,765,486	20,415	NOTAVEL	Middle	Andean	50	50	50	-50
Chr05	1,745,071	1,765,486	20,415	MAIII 16159 (UFLA)	Middle	Andean	50	49	49	-49
Chr05	4,487,842	4,506,067	18,225	IPA 9	Middle	Andean	100	100	74	-74
Chr05	4,487,842	4,506,067	18,225	PI 207262	Middle	Andean	100	99	72	-72
Chr05	4,487,842	4,506,067	18,225	US Pinto 111	Middle	Andean	100	96	71	-71
Chr05	1,780,465	1,791,759	11,294	BRS Ártico (WAF 75)	Andean	Middle	50	50	-50	50
Chr05	1,780,465	1,791,759	11,294	OURO BRANCO	Andean	Middle	50	46	-46	46
Chr05	4,753,143	4,756,681	3,538	IAC Imperador	Middle	Andean	50	50	48	-48
Chr05	4,753,143	4,756,681	3,538	Sel 1308	Middle	Andean	50	50	50	-50
Chr05	4,753,143	4,756,681	3,538	BRS Supremo	Middle	Andean	50	48	48	-48
Chr05	4,753,143	4,756,681	3,538	RUDA	Middle	Andean	50	50	50	-50
Chr05	4,753,143	4,756,681	3,538	PEROLA	Middle	Andean	50	50	50	-50
Chr05	4,753,143	4,756,681	3,538	Ouro Negro	Middle	Andean	50	50	50	-50
Chr05	4,753,143	4,756,681	3,538	IAC Diplomata	Middle	Andean	50	50	50	-50
Chr05	4,753,143	4,756,681	3,538	CNFC 16207	Middle	Andean	50	45	45	-45
Chr05	4,753,143	4,756,681	3,538	BRS Ametista	Middle	Andean	50	50	50	-50
Chr06	16,270,038	21,442,056	5,172,018	BRS Cometa	Middle	Andean	2650	2445	2374	-2374
Chr06	8,944,566	13,118,118	4,173,552	BRS Cometa	Middle	Andean	750	691	664	-664
Chr06	4,620,897	8,380,186	3,759,289	BRS Cometa	Middle	Andean	550	504	486	-486
Chr06	21,468,305	24,433,124	2,964,819	BRS Cometa	Middle	Andean	1500	1407	1384	-1384
Chr06	2,397,273	5,152,499	2,755,226	OURO BRANCO	Andean	Middle	450	405	-387	387
Chr06	12,465,604	14,608,975	2,143,371	OURO BRANCO	Andean	Middle	750	679	-671	671
Chr06	10,027,402	11,929,579	1,902,177	OURO BRANCO	Andean	Middle	350	322	-317	317
Chr06	24,641,364	26,078,802	1,437,438	BRS Cometa	Middle	Andean	850	786	758	-758
Chr06	250,056	1,443,733	1,193,677	OURO BRANCO	Andean	Middle	100	85	-67	67
Chr06	7,275,079	8,380,186	1,105,107	OURO BRANCO	Andean	Middle	150	128	-117	117
Chr06	15,112,180	16,201,058	1,088,878	BRS Cometa	Middle	Andean	450	409	371	-371
Chr06	1,503,073	2,394,348	891,275	Ouro Negro	Middle	Andean	100	99	95	-95
Chr06	1,503,073	2,394,348	891,275	CB 911921	Middle	Andean	100	99	95	-95
Chr06	1,503,073	2,394,348	891,275	BRS Cometa	Middle	Andean	100	91	89	-89
Chr06	13,736,526	14,486,919	750,393	BRS Cometa	Middle	Andean	150	139	122	-122
Chr06	15,112,180	15,806,800	694,620	BRS Horizonte	Middle	Andean	300	279	274	-274
Chr06	14,489,190	15,112,062	622,872	BRS FS305 (CAL 96)	Andean	Middle	1550	1540	-1518	1518
Chr06	3,892,818	4,357,873	465,055	BRS Cometa	Middle	Andean	150	143	100	-100
Chr06	23,389,222	23,837,544	448,322	Rosinha G2	Middle	Andean	400	345	344	-344
Chr06	5,825	411,022	405,197	Ouro Negro	Middle	Andean	100	99	77	-77
Chr06	9,270,158	9,619,610	349,452	OURO BRANCO	Andean	Middle	50	43	-43	43
Chr06	13,287,591	13,546,501	258,910	BRS FS305 (CAL 96)	Andean	Middle	150	150	-148	148
Chr06	5,825	249,848	244,023	CB 911921	Middle	Andean	50	48	40	-40
Chr06	5,825	249,848	244,023	BRS Cometa	Middle	Andean	50	44	41	-41
Chr06	23,596,097	23,837,544	241,447	BRS Madrepérola	Middle	Andean	300	298	281	-281
Chr06	23,596,097	23,837,544	241,447	ESTEIO	Middle	Andean	300	300	284	-284
Chr06	23,596,097	23,837,544	241,447	FC104	Middle	Andean	300	300	284	-284
Chr06	23,596,097	23,837,544	241,447	NOTAVEL	Middle	Andean	300	300	284	-284
Chr06	14,614,973	14,826,361	211,388	OURO BRANCO	Andean	Middle	550	509	-508	508
Chr06	5,744,760	5,920,528	175,768	OURO BRANCO	Andean	Middle	50	49	-31	31
Chr06	14,944,848	15,112,062	167,214	BRS Embaixador	Andean	Middle	550	543	-539	539
Chr06	23,689,628	23,837,544	147,916	CNFCT 19120	Middle	Andean	200	175	175	-175
Chr06	23,596,097	23,719,434	123,337	CNFC 10467	Middle	Andean	150	148	128	-128
Chr06	20,739,819	20,848,794	108,975	ESTILO	Middle	Andean	100	100	74	-74
Chr06	20,739,819	20,848,794	108,975	UIRAPURU	Middle	Andean	100	90	64	-64
Chr06	20,739,819	20,848,794	108,975	Bat 332	Middle	Andean	100	98	72	-72
Chr06	20,739,819	20,848,794	108,975	IAC Diplomata	Middle	Andean	100	100	74	-74
Chr06	17,492,395	17,593,691	101,296	US Pinto 111	Middle	Andean	50	45	33	-33
Chr06	17,492,395	17,593,691	101,296	G2333	Middle	Andean	50	50	30	-30
Chr06	17,492,395	17,593,691	101,296	FC104	Middle	Andean	50	50	31	-31
Chr06	17,492,395	17,593,691	101,296	BRS Sublime	Middle	Andean	50	49	30	-30
Chr06	25,104,990	25,196,405	91,415	ESTILO	Middle	Andean	100	100	82	-82
Chr06	25,104,990	25,196,405	91,415	BRS Supremo	Middle	Andean	100	89	76	-76
Chr06	25,104,990	25,196,405	91,415	RUDA	Middle	Andean	100	100	82	-82
Chr06	25,104,990	25,196,405	91,415	Bat 332	Middle	Andean	100	97	78	-78
Chr06	25,104,990	25,196,405	91,415	Ouro Negro	Middle	Andean	100	99	83	-83
Chr06	25,104,990	25,196,405	91,415	IAC Diplomata	Middle	Andean	100	100	82	-82
Chr06	25,104,990	25,196,405	91,415	CNFC 10467	Middle	Andean	100	96	78	-78
Chr06	25,104,990	25,196,405	91,415	CB 911921	Middle	Andean	100	97	79	-79
Chr06	20,411,041	20,492,167	81,126	TO	Middle	Andean	100	93	76	-76
Chr06	20,411,041	20,492,167	81,126	ESPLENDOR	Middle	Andean	100	100	82	-82
Chr06	20,411,041	20,492,167	81,126	PEROLA	Middle	Andean	100	100	82	-82
Chr06	20,411,041	20,492,167	81,126	CB 911921	Middle	Andean	100	98	82	-82
Chr06	23,254,573	23,319,277	64,704	ESPLENDOR	Middle	Andean	50	50	30	-30
Chr06	22,241,810	22,305,049	63,239	México 54	Middle	Andean	50	48	30	-30
Chr06	20,411,041	20,471,756	60,715	CNFC 16207	Middle	Andean	50	43	36	-36
Chr06	19,815,209	19,863,197	47,988	BRS Executivo	Andean	Middle	150	148	-122	122
Chr06	25,152,365	25,196,405	44,040	BRS Horizonte	Middle	Andean	50	50	34	-34

Chr	Start	End	Length	Sample	Background group	Haplotype group	Total variants	Genotyped variants	Score Andean	Score Middle
Chr06	25,275,139	25,317,801	42,662	BRS Horizonte	Middle	Andean	50	49	30	-30
Chr06	25,275,139	25,317,801	42,662	ESTILO	Middle	Andean	50	50	32	-32
Chr06	25,275,139	25,317,801	42,662	RUDA	Middle	Andean	50	50	32	-32
Chr06	25,275,139	25,317,801	42,662	Bat 332	Middle	Andean	50	50	32	-32
Chr06	25,275,139	25,317,801	42,662	Ouro Negro	Middle	Andean	50	49	33	-33
Chr06	25,275,139	25,317,801	42,662	IAC Diplomata	Middle	Andean	50	50	32	-32
Chr06	25,275,139	25,317,801	42,662	CNFC 10467	Middle	Andean	50	50	30	-30
Chr06	25,275,139	25,317,801	42,662	CB 911921	Middle	Andean	50	48	32	-32
Chr06	20,739,819	20,768,959	29,140	TO	Middle	Andean	50	45	32	-32
Chr06	20,739,819	20,768,959	29,140	PEROLA	Middle	Andean	50	50	38	-38
Chr06	20,739,819	20,768,959	29,140	CNFC 16207	Middle	Andean	50	45	36	-36
Chr06	20,739,819	20,768,959	29,140	CB 911921	Middle	Andean	50	50	38	-38
Chr07	24,268,110	28,776,732	4,508,622	MAIII 16159 (UFLA)	Middle	Andean	1600	1495	1461	-1461
Chr07	37,211,391	40,027,021	2,815,630	CB 911921	Middle	Andean	1450	1393	1340	-1340
Chr07	7,476,796	9,874,414	2,397,618	CNFC 16207	Middle	Andean	1500	1354	1238	-1238
Chr07	11,707,870	13,554,976	1,847,106	CNFC 16207	Middle	Andean	350	321	290	-290
Chr07	18,729,817	20,372,271	1,642,454	TO	Middle	Andean	50	43	31	-31
Chr07	18,729,817	20,372,271	1,642,454	PEROLA	Middle	Andean	50	49	35	-35
Chr07	18,729,817	20,372,271	1,642,454	CNFCT 19120	Middle	Andean	50	44	36	-36
Chr07	18,729,817	20,372,271	1,642,454	CNFC 16207	Middle	Andean	50	43	37	-37
Chr07	29,429,688	30,713,905	1,284,217	MAIII 16159 (UFLA)	Middle	Andean	750	700	689	-689
Chr07	10,109,574	11,216,944	1,107,370	CNFC 16207	Middle	Andean	250	233	178	-178
Chr07	35,449,808	36,446,351	996,543	TO	Middle	Andean	600	560	556	-556
Chr07	32,641,917	33,577,107	935,190	TO	Middle	Andean	400	366	316	-316
Chr07	7,198,707	8,094,760	896,053	CNFCT 19120	Middle	Andean	400	352	313	-313
Chr07	6,281,365	6,694,296	412,931	Rosinha G2	Middle	Andean	300	258	255	-255
Chr07	12,009,041	12,405,019	395,978	CNFCT 19120	Middle	Andean	150	131	121	-121
Chr07	8,360,386	8,756,300	395,914	CNFCT 19120	Middle	Andean	150	129	115	-115
Chr07	17,487,151	17,856,924	369,773	OURO BRANCO	Andean	Middle	50	46	-40	40
Chr07	9,071,746	9,403,697	331,951	CNFCT 19120	Middle	Andean	200	181	176	-176
Chr07	12,078,018	12,405,019	327,001	US Pinto 111	Middle	Andean	100	95	70	-70
Chr07	6,792,804	7,096,813	304,009	Rosinha G2	Middle	Andean	100	85	74	-74
Chr07	28,785,268	29,084,416	299,148	MAIII 16159 (UFLA)	Middle	Andean	600	554	547	-547
Chr07	9,505,830	9,803,693	297,863	CNFCT 19120	Middle	Andean	450	412	394	-394
Chr07	29,116,424	29,411,241	294,817	MAIII 16159 (UFLA)	Middle	Andean	700	653	647	-647
Chr07	7,098,229	7,387,452	289,223	CNFC 16207	Middle	Andean	100	89	84	-84
Chr07	10,375,771	10,658,083	282,312	CNFCT 19120	Middle	Andean	50	44	30	-30
Chr07	11,707,870	11,964,663	256,793	IAC Imperador	Middle	Andean	100	98	83	-83
Chr07	11,707,870	11,964,663	256,793	US Pinto 111	Middle	Andean	100	96	84	-84
Chr07	11,707,870	11,964,663	256,793	NOTAVEL	Middle	Andean	100	100	87	-87
Chr07	11,707,870	11,964,663	256,793	CNFCT 19120	Middle	Andean	100	89	85	-85
Chr07	10,971,293	11,216,944	245,651	CNFCT 19120	Middle	Andean	50	42	36	-36
Chr07	12,164,938	12,405,019	240,081	IAC Imperador	Middle	Andean	50	50	40	-40
Chr07	12,164,938	12,405,019	240,081	TO	Middle	Andean	50	47	39	-39
Chr07	12,164,938	12,405,019	240,081	PEROLA	Middle	Andean	50	50	40	-40
Chr07	12,164,938	12,405,019	240,081	NOTAVEL	Middle	Andean	50	50	40	-40
Chr07	36,653,777	36,856,817	203,040	CB 911921	Middle	Andean	100	99	98	-98
Chr07	7,387,458	7,583,199	195,741	Rosinha G2	Middle	Andean	150	121	107	-107
Chr07	6,792,804	6,957,265	164,461	CNFC 16207	Middle	Andean	50	45	39	-39
Chr07	39,706,469	39,851,081	144,612	AND277	Andean	Middle	200	200	-200	200
Chr07	28,268,264	28,410,510	142,246	US Pinto 111	Middle	Andean	100	96	86	-86
Chr07	28,268,264	28,410,510	142,246	México 54	Middle	Andean	100	100	89	-89
Chr07	28,268,264	28,410,510	142,246	G2333	Middle	Andean	100	100	89	-89
Chr07	28,268,264	28,410,510	142,246	NOTAVEL	Middle	Andean	100	100	90	-90
Chr07	6,388,867	6,522,937	134,070	TO	Middle	Andean	100	99	73	-73
Chr07	6,388,867	6,522,937	134,070	ESTEIO	Middle	Andean	100	100	80	-80
Chr07	39,896,512	40,027,021	130,509	AND277	Andean	Middle	200	200	-136	136
Chr07	10,109,574	10,233,568	123,994	CNFCT 19120	Middle	Andean	50	41	33	-33
Chr07	8,360,386	8,482,448	122,062	Sel 1308	Middle	Andean	50	50	32	-32
Chr07	8,360,386	8,482,448	122,062	FC104	Middle	Andean	50	50	32	-32
Chr07	8,360,386	8,482,448	122,062	ESTILO	Middle	Andean	50	50	32	-32
Chr07	8,360,386	8,482,448	122,062	RUDA	Middle	Andean	50	50	32	-32
Chr07	8,360,386	8,482,448	122,062	CB 911921	Middle	Andean	50	49	31	-31
Chr07	33,467,348	33,577,107	109,759	PI 207262	Middle	Andean	50	50	33	-33
Chr07	11,707,870	11,814,825	106,955	IPA 9	Middle	Andean	50	50	47	-47
Chr07	29,116,424	29,213,943	97,519	TO	Middle	Andean	200	179	159	-159
Chr07	29,116,424	29,213,943	97,519	IPA 9	Middle	Andean	200	196	174	-174
Chr07	29,116,424	29,213,943	97,519	México 54	Middle	Andean	200	188	165	-165
Chr07	38,837,802	38,926,492	88,690	TO	Middle	Andean	50	45	37	-37
Chr07	7,387,458	7,474,570	87,112	ESTEIO	Middle	Andean	50	50	32	-32
Chr07	37,527,615	37,607,705	80,090	México 54	Middle	Andean	50	40	32	-32
Chr07	22,789,839	22,869,692	79,853	TO	Middle	Andean	50	49	40	-40
Chr07	22,789,839	22,869,692	79,853	PEROLA	Middle	Andean	50	50	41	-41
Chr07	34,194,563	34,267,770	73,207	IPA 9	Middle	Andean	50	49	31	-31
Chr07	34,194,563	34,267,770	73,207	PI 207262	Middle	Andean	50	48	32	-32
Chr07	34,194,563	34,267,770	73,207	México 54	Middle	Andean	50	48	30	-30

Chr	Start	End	Length	Sample	Background group	Haplotype group	Total variants	Genotyped variants	Score Andean	Score Middle
Chr07	34,194,563	34,267,770	73,207	G2333	Middle	Andean	50	49	30	-30
Chr07	34,194,563	34,267,770	73,207	NOTAVEL	Middle	Andean	50	50	32	-32
Chr07	26,523,178	26,590,051	66,873	RUDA	Middle	Andean	50	50	30	-30
Chr07	26,523,178	26,590,051	66,873	IAC Diplomata	Middle	Andean	50	50	30	-30
Chr07	9,633,923	9,697,997	64,074	TO	Middle	Andean	50	46	36	-36
Chr07	9,633,923	9,697,997	64,074	PEROLA	Middle	Andean	50	50	40	-40
Chr07	9,810,631	9,874,414	63,783	CNFCT 19120	Middle	Andean	50	41	37	-37
Chr07	597,296	652,471	55,175	TO	Middle	Andean	100	94	74	-74
Chr07	597,296	652,471	55,175	PI 207262	Middle	Andean	100	92	81	-81
Chr07	597,296	652,471	55,175	FC104	Middle	Andean	100	100	80	-80
Chr07	597,296	652,471	55,175	ESTILO	Middle	Andean	100	100	80	-80
Chr07	597,296	652,471	55,175	PEROLA	Middle	Andean	100	100	80	-80
Chr07	597,296	652,471	55,175	Bat 332	Middle	Andean	100	100	80	-80
Chr07	597,296	652,471	55,175	CB 911921	Middle	Andean	100	89	78	-78
Chr07	8,178,324	8,231,204	52,880	CNFCT 19120	Middle	Andean	50	43	37	-37
Chr07	6,643,856	6,694,296	50,440	TO	Middle	Andean	50	47	30	-30
Chr07	6,643,856	6,694,296	50,440	ESTEIO	Middle	Andean	50	50	34	-34
Chr07	3,682,645	3,732,649	50,004	Jalo Precoce	Andean	Middle	100	100	-66	66
Chr07	9,505,830	9,553,657	47,827	TO	Middle	Andean	100	90	87	-87
Chr07	9,505,830	9,553,657	47,827	ESPLENDOR	Middle	Andean	100	100	95	-95
Chr07	9,505,830	9,553,657	47,827	IPA 9	Middle	Andean	100	100	95	-95
Chr07	9,505,830	9,553,657	47,827	ESTEIO	Middle	Andean	100	100	97	-97
Chr07	9,505,830	9,553,657	47,827	PEROLA	Middle	Andean	100	100	97	-97
Chr07	32,094,306	32,141,195	46,889	BRS Horizonte	Middle	Andean	50	43	33	-33
Chr07	32,094,306	32,141,195	46,889	México 54	Middle	Andean	50	46	35	-35
Chr07	32,094,306	32,141,195	46,889	ESTEIO	Middle	Andean	50	50	40	-40
Chr07	32,094,306	32,141,195	46,889	ESTILO	Middle	Andean	50	50	40	-40
Chr07	32,094,306	32,141,195	46,889	PEROLA	Middle	Andean	50	50	40	-40
Chr07	32,094,306	32,141,195	46,889	MAIII 16159 (UFLA)	Middle	Andean	50	47	39	-39
Chr07	32,094,306	32,141,195	46,889	CNFC 10794	Middle	Andean	50	50	40	-40
Chr07	32,094,306	32,141,195	46,889	CB 911921	Middle	Andean	50	47	37	-37
Chr07	1,198,559	1,243,705	45,146	IPA 9	Middle	Andean	50	50	40	-40
Chr07	1,198,559	1,243,705	45,146	PI 207262	Middle	Andean	50	49	37	-37
Chr07	1,198,559	1,243,705	45,146	México 54	Middle	Andean	50	50	38	-38
Chr07	1,198,559	1,243,705	45,146	G2333	Middle	Andean	50	50	36	-36
Chr07	1,198,559	1,243,705	45,146	IAC Diplomata	Middle	Andean	50	50	40	-40
Chr07	1,198,559	1,243,705	45,146	CNFC 16207	Middle	Andean	50	44	34	-34
Chr07	1,198,559	1,243,705	45,146	CNFC 10467	Middle	Andean	50	44	34	-34
Chr07	1,198,559	1,243,705	45,146	BRS Ametista	Middle	Andean	50	50	40	-40
Chr07	11,964,926	12,008,502	43,576	PEROLA	Middle	Andean	50	50	36	-36
Chr07	66,412	109,982	43,570	PI 207262	Middle	Andean	50	48	32	-32
Chr07	3,654,070	3,697,508	43,438	OURO BRANCO	Andean	Middle	100	91	-81	81
Chr07	32,387,787	32,427,539	39,752	BRS Horizonte	Middle	Andean	100	93	66	-66
Chr07	32,387,787	32,427,539	39,752	México 54	Middle	Andean	100	93	72	-72
Chr07	32,387,787	32,427,539	39,752	ESTEIO	Middle	Andean	100	100	72	-72
Chr07	32,387,787	32,427,539	39,752	ESTILO	Middle	Andean	100	100	72	-72
Chr07	32,387,787	32,427,539	39,752	PEROLA	Middle	Andean	100	100	72	-72
Chr07	32,387,787	32,427,539	39,752	MAIII 16159 (UFLA)	Middle	Andean	100	98	75	-75
Chr07	32,387,787	32,427,539	39,752	CNFC 10794	Middle	Andean	100	100	73	-73
Chr07	32,387,787	32,427,539	39,752	CB 911921	Middle	Andean	100	100	72	-72
Chr07	29,085,027	29,123,755	38,728	G2333	Middle	Andean	100	99	79	-79
Chr07	29,085,027	29,123,755	38,728	NOTAVEL	Middle	Andean	100	100	80	-80
Chr07	3,452,035	3,489,286	37,251	ESPLENDOR	Middle	Andean	50	50	38	-38
Chr07	3,452,035	3,489,286	37,251	IPA 9	Middle	Andean	50	50	38	-38
Chr07	3,452,035	3,489,286	37,251	Rosinha G2	Middle	Andean	50	42	32	-32
Chr07	3,452,035	3,489,286	37,251	BRS Madrepérola	Middle	Andean	50	48	36	-36
Chr07	3,452,035	3,489,286	37,251	ESTEIO	Middle	Andean	50	50	38	-38
Chr07	3,452,035	3,489,286	37,251	ESTILO	Middle	Andean	50	50	38	-38
Chr07	3,452,035	3,489,286	37,251	RUDA	Middle	Andean	50	50	38	-38
Chr07	3,452,035	3,489,286	37,251	Bat 332	Middle	Andean	50	50	30	-30
Chr07	3,452,035	3,489,286	37,251	IAC Diplomata	Middle	Andean	50	50	38	-38
Chr07	3,452,035	3,489,286	37,251	CNFC 10794	Middle	Andean	50	50	38	-38
Chr07	3,452,035	3,489,286	37,251	BRS Ametista	Middle	Andean	50	50	38	-38
Chr07	1,308,297	1,341,144	32,847	TO	Middle	Andean	50	49	35	-35
Chr07	1,308,297	1,341,144	32,847	IPA 9	Middle	Andean	50	47	35	-35
Chr07	1,308,297	1,341,144	32,847	US Pinto 111	Middle	Andean	50	48	36	-36
Chr07	1,308,297	1,341,144	32,847	G2333	Middle	Andean	50	50	35	-35
Chr07	1,308,297	1,341,144	32,847	Bat 332	Middle	Andean	50	50	36	-36
Chr07	1,308,297	1,341,144	32,847	IAC Diplomata	Middle	Andean	50	50	36	-36
Chr07	1,308,297	1,341,144	32,847	CNFC 10467	Middle	Andean	50	50	36	-36
Chr07	1,308,297	1,341,144	32,847	BRS Ametista	Middle	Andean	50	45	33	-33
Chr07	3,921,927	3,952,981	31,054	OURO BRANCO	Andean	Middle	200	185	-173	173
Chr07	625,649	652,471	26,822	IPA 9	Middle	Andean	50	50	46	-46
Chr07	625,649	652,471	26,822	IAC Diplomata	Middle	Andean	50	50	46	-46
Chr07	625,649	652,471	26,822	CNFC 16207	Middle	Andean	50	45	43	-43
Chr07	625,649	652,471	26,822	CNFC 10467	Middle	Andean	50	49	43	-43

Chr	Start	End	Length	Sample	Background group	Haplotype group	Total variants	Genotyped variants	Score Andean	Score Middle
Chr07	625,649	652,471	26,822	BRS Ametista	Middle	Andean	50	50	46	-46
Chr07	29,027,577	29,049,527	21,950	IPA 9	Middle	Andean	100	100	67	-67
Chr07	29,365,679	29,385,510	19,831	México 54	Middle	Andean	50	48	36	-36
Chr07	29,411,891	29,429,664	17,773	TO	Middle	Andean	50	44	32	-32
Chr07	29,411,891	29,429,664	17,773	México 54	Middle	Andean	50	47	35	-35
Chr07	32,387,787	32,404,709	16,922	TO	Middle	Andean	50	48	40	-40
Chr07	2,470,772	2,483,549	12,777	BRS FS305 (CAL 96)	Andean	Middle	50	50	-32	32
Chr07	29,038,993	29,049,527	10,534	TO	Middle	Andean	50	50	36	-36
Chr07	29,640,564	29,650,131	9,567	México 54	Middle	Andean	50	40	32	-32
Chr07	29,116,424	29,123,755	7,331	US Pinto 111	Middle	Andean	50	48	47	-47
Chr07	29,231,316	29,233,247	1,931	IPA 9	Middle	Andean	50	50	48	-48
Chr07	29,231,316	29,233,247	1,931	México 54	Middle	Andean	50	50	48	-48
Chr08	54,097,669	59,660,513	5,562,844	CNFC 10467	Middle	Andean	3300	3256	3108	-3108
Chr08	54,518,385	58,235,682	3,717,297	BRS Sublime	Middle	Andean	2250	2186	2087	-2087
Chr08	54,097,669	57,551,293	3,453,624	FC104	Middle	Andean	2100	2100	2023	-2023
Chr08	46,584,260	48,481,862	1,897,602	AND277	Andean	Middle	450	450	-446	446
Chr08	6,987,411	8,748,003	1,760,592	ESPLENDOR	Middle	Andean	1500	1500	1343	-1343
Chr08	6,987,411	8,748,003	1,760,592	RUDA	Middle	Andean	1500	1500	1343	-1343
Chr08	6,987,411	8,748,003	1,760,592	NOTAVEL	Middle	Andean	1500	1500	1345	-1345
Chr08	6,987,411	8,748,003	1,760,592	IAC Diplomata	Middle	Andean	1500	1500	1343	-1343
Chr08	6,987,411	8,748,003	1,760,592	BRS Ametista	Middle	Andean	1500	1410	1238	-1238
Chr08	57,115,750	58,813,829	1,698,079	ESTEIO	Middle	Andean	1250	1250	1214	-1214
Chr08	8,867,131	10,492,571	1,625,440	ESPLENDOR	Middle	Andean	1000	1000	951	-951
Chr08	44,346,234	45,940,411	1,594,177	AND277	Andean	Middle	500	500	-438	438
Chr08	6,987,411	8,543,967	1,556,556	BRS Horizonte	Middle	Andean	1400	1321	1185	-1185
Chr08	6,987,411	8,543,967	1,556,556	BRS Cometa	Middle	Andean	1400	1282	1150	-1150
Chr08	8,867,131	10,264,728	1,397,597	BRS Horizonte	Middle	Andean	950	920	872	-872
Chr08	8,867,131	10,264,728	1,397,597	RUDA	Middle	Andean	950	950	912	-912
Chr08	8,867,131	10,264,728	1,397,597	NOTAVEL	Middle	Andean	950	950	912	-912
Chr08	8,867,131	10,264,728	1,397,597	IAC Diplomata	Middle	Andean	950	950	913	-913
Chr08	8,867,131	10,264,728	1,397,597	BRS Cometa	Middle	Andean	950	897	843	-843
Chr08	55,557,906	56,441,705	883,799	CNFCT 19120	Middle	Andean	450	399	373	-373
Chr08	19,404,733	20,158,651	753,918	ESPLENDOR	Middle	Andean	250	250	210	-210
Chr08	19,404,733	20,158,651	753,918	US Pinto 111	Middle	Andean	250	230	188	-188
Chr08	19,404,733	20,158,651	753,918	G2333	Middle	Andean	250	246	204	-204
Chr08	19,404,733	20,158,651	753,918	CNFC 16207	Middle	Andean	250	220	182	-182
Chr08	19,404,733	20,007,174	602,441	México 54	Middle	Andean	150	146	111	-111
Chr08	19,404,733	20,007,174	602,441	Bat 332	Middle	Andean	150	143	111	-111
Chr08	54,097,669	54,653,668	555,999	CNFCT 19120	Middle	Andean	300	252	245	-245
Chr08	20,013,139	20,441,367	428,228	BRS Cometa	Middle	Andean	250	243	189	-189
Chr08	53,122,561	53,544,798	422,237	ESTEIO	Middle	Andean	300	299	262	-262
Chr08	167,271	587,118	419,847	ESPLENDOR	Middle	Andean	700	700	699	-699
Chr08	167,271	587,118	419,847	BRS Madrepérola	Middle	Andean	700	677	669	-669
Chr08	167,271	587,118	419,847	ESTEIO	Middle	Andean	700	700	698	-698
Chr08	167,271	587,118	419,847	RUDA	Middle	Andean	700	700	698	-698
Chr08	167,271	587,118	419,847	MAIII 16159 (UFLA)	Middle	Andean	700	664	660	-660
Chr08	167,271	587,118	419,847	IAC Diplomata	Middle	Andean	700	700	698	-698
Chr08	167,271	587,118	419,847	CNFC 16207	Middle	Andean	700	642	631	-631
Chr08	62,431,193	62,846,666	415,473	AND277	Andean	Middle	200	200	-186	186
Chr08	11,418,097	11,807,682	389,585	RUDA	Middle	Andean	400	400	392	-392
Chr08	11,418,097	11,807,682	389,585	NOTAVEL	Middle	Andean	400	400	393	-393
Chr08	11,418,097	11,807,682	389,585	IAC Diplomata	Middle	Andean	400	400	392	-392
Chr08	11,418,097	11,807,682	389,585	BRS Cometa	Middle	Andean	400	365	357	-357
Chr08	53,122,561	53,495,796	373,235	CNFC 10467	Middle	Andean	250	248	231	-231
Chr08	167,271	507,842	340,571	BRS Ametista	Middle	Andean	500	469	464	-464
Chr08	263,044	587,118	324,074	Rosinha G2	Middle	Andean	550	481	478	-478
Chr08	281,867	587,118	305,251	IAC Imperador	Middle	Andean	500	490	474	-474
Chr08	54,097,669	54,385,856	288,187	BRS Sublime	Middle	Andean	200	189	184	-184
Chr08	11,534,450	11,807,682	273,232	BRS Horizonte	Middle	Andean	350	335	327	-327
Chr08	54,691,444	54,964,155	272,711	CNFCT 19120	Middle	Andean	150	130	127	-127
Chr08	56,708,912	56,979,309	270,397	CNFCT 19120	Middle	Andean	150	122	110	-110
Chr08	57,115,750	57,374,090	258,340	CNFCT 19120	Middle	Andean	300	272	265	-265
Chr08	51,492,149	51,728,393	236,244	CB 911921	Middle	Andean	50	50	32	-32
Chr08	62,431,193	62,658,814	227,621	PEROLA	Middle	Andean	100	98	68	-68
Chr08	8,158,067	8,379,308	221,241	UIRAPURU	Middle	Andean	100	89	62	-62
Chr08	20,220,842	20,441,367	220,525	IAC Imperador	Middle	Andean	100	98	83	-83
Chr08	20,220,842	20,441,367	220,525	ESPLENDOR	Middle	Andean	100	100	83	-83
Chr08	20,220,842	20,441,367	220,525	BRS Horizonte	Middle	Andean	100	91	72	-72
Chr08	20,220,842	20,441,367	220,525	US Pinto 111	Middle	Andean	100	86	74	-74
Chr08	20,220,842	20,441,367	220,525	G2333	Middle	Andean	100	96	84	-84
Chr08	20,220,842	20,441,367	220,525	RUDA	Middle	Andean	100	100	83	-83
Chr08	20,220,842	20,441,367	220,525	NOTAVEL	Middle	Andean	100	100	83	-83
Chr08	20,220,842	20,441,367	220,525	IAC Diplomata	Middle	Andean	100	100	83	-83
Chr08	20,220,842	20,441,367	220,525	CNFC 16207	Middle	Andean	100	100	89	-89
Chr08	20,584,043	20,794,998	210,955	IAC Imperador	Middle	Andean	100	96	78	-78
Chr08	20,584,043	20,794,998	210,955	ESPLENDOR	Middle	Andean	100	100	82	-82

Chr	Start	End	Length	Sample	Background group	Haplotype group	Total variants	Genotyped variants	Score Andean	Score Middle
Chr08	20,584,043	20,794,998	210,955	BRS Horizonte	Middle	Andean	100	92	70	-70
Chr08	20,584,043	20,794,998	210,955	G2333	Middle	Andean	100	95	76	-76
Chr08	20,584,043	20,794,998	210,955	RUDA	Middle	Andean	100	100	82	-82
Chr08	20,584,043	20,794,998	210,955	NOTAVEL	Middle	Andean	100	100	81	-81
Chr08	20,584,043	20,794,998	210,955	IAC Diplomata	Middle	Andean	100	100	82	-82
Chr08	20,584,043	20,794,998	210,955	BRS Cometa	Middle	Andean	100	96	71	-71
Chr08	59,771,829	59,970,715	198,886	CNFC 10467	Middle	Andean	100	99	98	-98
Chr08	346,234	535,142	188,908	UIRAPURU	Middle	Andean	250	233	171	-171
Chr08	11,418,097	11,590,098	172,001	ESPLENDOR	Middle	Andean	150	150	128	-128
Chr08	55,311,466	55,480,129	168,663	CNFCT 19120	Middle	Andean	100	86	85	-85
Chr08	29,334,368	29,501,590	167,222	TO	Middle	Andean	100	94	67	-67
Chr08	29,334,368	29,501,590	167,222	ESPLENDOR	Middle	Andean	100	100	72	-72
Chr08	29,334,368	29,501,590	167,222	PI 207262	Middle	Andean	100	97	67	-67
Chr08	29,334,368	29,501,590	167,222	México 54	Middle	Andean	100	97	69	-69
Chr08	29,334,368	29,501,590	167,222	G2333	Middle	Andean	100	100	70	-70
Chr08	29,334,368	29,501,590	167,222	Ouro Negro	Middle	Andean	100	100	72	-72
Chr08	19,404,733	19,566,083	161,350	PI 207262	Middle	Andean	100	96	78	-78
Chr08	19,404,733	19,566,083	161,350	MAIII 16159 (UFLA)	Middle	Andean	100	83	67	-67
Chr08	53,656,458	53,811,748	155,290	IAC Imperador	Middle	Andean	150	145	129	-129
Chr08	53,656,458	53,811,748	155,290	ESPLENDOR	Middle	Andean	150	150	144	-144
Chr08	53,656,458	53,811,748	155,290	BRS Madrepérola	Middle	Andean	150	147	141	-141
Chr08	53,656,458	53,811,748	155,290	ESTEIO	Middle	Andean	150	150	144	-144
Chr08	53,656,458	53,811,748	155,290	RUDA	Middle	Andean	150	150	144	-144
Chr08	53,656,458	53,811,748	155,290	Bat 332	Middle	Andean	150	149	143	-143
Chr08	53,656,458	53,811,748	155,290	CB 911921	Middle	Andean	150	147	126	-126
Chr08	29,917,616	30,071,741	154,125	TO	Middle	Andean	100	93	70	-70
Chr08	29,917,616	30,071,741	154,125	ESPLENDOR	Middle	Andean	100	100	79	-79
Chr08	29,917,616	30,071,741	154,125	PI 207262	Middle	Andean	100	96	74	-74
Chr08	29,917,616	30,071,741	154,125	US Pinto 111	Middle	Andean	100	99	76	-76
Chr08	29,917,616	30,071,741	154,125	México 54	Middle	Andean	100	95	72	-72
Chr08	29,917,616	30,071,741	154,125	G2333	Middle	Andean	100	100	79	-79
Chr08	29,917,616	30,071,741	154,125	Ouro Negro	Middle	Andean	100	97	74	-74
Chr08	29,917,616	30,071,741	154,125	CNFC 16207	Middle	Andean	100	100	80	-80
Chr08	20,013,139	20,158,651	145,512	IAC Imperador	Middle	Andean	100	97	78	-78
Chr08	20,013,139	20,158,651	145,512	BRS Horizonte	Middle	Andean	100	94	72	-72
Chr08	20,013,139	20,158,651	145,512	RUDA	Middle	Andean	100	100	80	-80
Chr08	20,013,139	20,158,651	145,512	NOTAVEL	Middle	Andean	100	100	80	-80
Chr08	20,013,139	20,158,651	145,512	IAC Diplomata	Middle	Andean	100	100	80	-80
Chr08	9,920,908	10,057,783	136,875	UIRAPURU	Middle	Andean	50	44	35	-35
Chr08	50,356,530	50,492,239	135,709	BRS Supremo	Middle	Andean	50	45	35	-35
Chr08	57,419,182	57,551,293	132,111	CNFCT 19120	Middle	Andean	100	82	80	-80
Chr08	55,075,826	55,205,279	129,453	CNFCT 19120	Middle	Andean	50	46	46	-46
Chr08	7,217,164	7,346,343	129,179	UIRAPURU	Middle	Andean	100	83	68	-68
Chr08	18,363,235	18,490,158	126,923	IAC Imperador	Middle	Andean	150	139	116	-116
Chr08	18,363,235	18,490,158	126,923	ESPLENDOR	Middle	Andean	150	150	120	-120
Chr08	18,363,235	18,490,158	126,923	BRS Horizonte	Middle	Andean	150	138	106	-106
Chr08	18,363,235	18,490,158	126,923	US Pinto 111	Middle	Andean	150	142	114	-114
Chr08	18,363,235	18,490,158	126,923	G2333	Middle	Andean	150	150	116	-116
Chr08	18,363,235	18,490,158	126,923	RUDA	Middle	Andean	150	150	120	-120
Chr08	18,363,235	18,490,158	126,923	NOTAVEL	Middle	Andean	150	150	120	-120
Chr08	18,363,235	18,490,158	126,923	IAC Diplomata	Middle	Andean	150	150	120	-120
Chr08	18,363,235	18,490,158	126,923	CNFC 16207	Middle	Andean	150	131	108	-108
Chr08	20,220,842	20,345,062	124,220	UIRAPURU	Middle	Andean	50	48	35	-35
Chr08	21,791,364	21,913,146	121,782	CB 911921	Middle	Andean	50	47	33	-33
Chr08	8,626,331	8,748,003	121,672	BRS Horizonte	Middle	Andean	50	50	36	-36
Chr08	8,626,331	8,748,003	121,672	BRS Cometa	Middle	Andean	50	46	34	-34
Chr08	53,693,223	53,811,748	118,525	Rosinha G2	Middle	Andean	100	82	76	-76
Chr08	9,480,577	9,597,805	117,228	UIRAPURU	Middle	Andean	50	48	37	-37
Chr08	8,867,131	8,984,133	117,002	BRS Ametista	Middle	Andean	50	50	44	-44
Chr08	11,418,097	11,534,442	116,345	UIRAPURU	Middle	Andean	50	41	31	-31
Chr08	53,000,376	53,115,420	115,044	AND277	Andean	Middle	50	50	-30	30
Chr08	53,205,479	53,311,803	106,324	BRS Sublime	Middle	Andean	100	95	90	-90
Chr08	20,584,043	20,687,365	103,322	US Pinto 111	Middle	Andean	50	45	37	-37
Chr08	20,584,043	20,687,365	103,322	UIRAPURU	Middle	Andean	50	46	30	-30
Chr08	20,584,043	20,687,365	103,322	CNFC 16207	Middle	Andean	50	44	36	-36
Chr08	56,167,785	56,264,809	97,024	TO	Middle	Andean	50	41	31	-31
Chr08	56,167,785	56,264,809	97,024	ESTILO	Middle	Andean	50	49	39	-39
Chr08	56,167,785	56,264,809	97,024	PEROLA	Middle	Andean	50	50	39	-39
Chr08	56,167,785	56,264,809	97,024	CNFC 10794	Middle	Andean	50	48	38	-38
Chr08	56,167,785	56,264,809	97,024	CB 911921	Middle	Andean	50	48	30	-30
Chr08	5,005,268	5,094,763	89,495	US Pinto 111	Middle	Andean	50	46	35	-35
Chr08	5,005,268	5,094,763	89,495	México 54	Middle	Andean	50	49	40	-40
Chr08	5,005,268	5,094,763	89,495	ESTEIO	Middle	Andean	50	50	39	-39
Chr08	5,005,268	5,094,763	89,495	BRS Supremo	Middle	Andean	50	46	36	-36
Chr08	18,953,550	19,042,252	88,702	IAC Imperador	Middle	Andean	50	50	31	-31
Chr08	18,953,550	19,042,252	88,702	ESPLENDOR	Middle	Andean	50	50	34	-34

Chr	Start	End	Length	Sample	Background group	Haplotype group	Total variants	Genotyped variants	Score Andean	Score Middle
Chr08	18,953,550	19,042,252	88,702	BRS Horizonte	Middle	Andean	50	47	31	-31
Chr08	18,953,550	19,042,252	88,702	US Pinto 111	Middle	Andean	50	45	31	-31
Chr08	18,953,550	19,042,252	88,702	G2333	Middle	Andean	50	50	34	-34
Chr08	18,953,550	19,042,252	88,702	RUDA	Middle	Andean	50	50	32	-32
Chr08	18,953,550	19,042,252	88,702	NOTAVEL	Middle	Andean	50	50	32	-32
Chr08	18,953,550	19,042,252	88,702	IAC Diplomata	Middle	Andean	50	50	32	-32
Chr08	18,953,550	19,042,252	88,702	CNFC 16207	Middle	Andean	50	44	30	-30
Chr08	9,671,939	9,759,742	87,803	UIRAPURU	Middle	Andean	100	94	66	-66
Chr08	20,013,139	20,099,353	86,214	UIRAPURU	Middle	Andean	50	50	33	-33
Chr08	11,534,450	11,619,151	84,701	PI 207262	Middle	Andean	150	148	134	-134
Chr08	11,534,450	11,619,151	84,701	México 54	Middle	Andean	150	146	138	-138
Chr08	11,534,450	11,619,151	84,701	Ouro Negro	Middle	Andean	150	147	137	-137
Chr08	7,459,252	7,542,071	82,819	UIRAPURU	Middle	Andean	150	139	105	-105
Chr08	58,737,496	58,813,829	76,333	US Pinto 111	Middle	Andean	50	49	31	-31
Chr08	58,737,496	58,813,829	76,333	FC104	Middle	Andean	50	50	32	-32
Chr08	58,737,496	58,813,829	76,333	BRS Sublime	Middle	Andean	50	48	30	-30
Chr08	58,737,496	58,813,829	76,333	CNFCT 19120	Middle	Andean	50	47	30	-30
Chr08	58,737,496	58,813,829	76,333	CNFC 16207	Middle	Andean	50	42	30	-30
Chr08	6,328,216	6,400,430	72,214	CB 911921	Middle	Andean	50	40	35	-35
Chr08	61,579,130	61,645,426	66,296	ESTILO	Middle	Andean	50	50	36	-36
Chr08	61,579,130	61,645,426	66,296	RUDA	Middle	Andean	50	50	36	-36
Chr08	61,579,130	61,645,426	66,296	CNFC 10794	Middle	Andean	50	48	35	-35
Chr08	7,713,041	7,778,348	65,307	UIRAPURU	Middle	Andean	50	46	32	-32
Chr08	38,646,650	38,711,937	65,287	TO	Middle	Andean	50	49	38	-38
Chr08	38,646,650	38,711,937	65,287	ESPLENDOR	Middle	Andean	50	50	40	-40
Chr08	38,646,650	38,711,937	65,287	PI 207262	Middle	Andean	50	49	38	-38
Chr08	38,646,650	38,711,937	65,287	US Pinto 111	Middle	Andean	50	50	38	-38
Chr08	38,646,650	38,711,937	65,287	México 54	Middle	Andean	50	50	40	-40
Chr08	38,646,650	38,711,937	65,287	G2333	Middle	Andean	50	50	38	-38
Chr08	38,646,650	38,711,937	65,287	Ouro Negro	Middle	Andean	50	50	40	-40
Chr08	38,646,650	38,711,937	65,287	CNFC 16207	Middle	Andean	50	45	35	-35
Chr08	11,590,399	11,655,003	64,604	UIRAPURU	Middle	Andean	100	97	77	-77
Chr08	43,847,229	43,910,747	63,518	US Pinto 111	Middle	Andean	50	45	42	-42
Chr08	43,847,229	43,910,747	63,518	CNFC 16207	Middle	Andean	50	46	44	-44
Chr08	51,986,065	52,045,390	59,325	TO	Middle	Andean	50	46	38	-38
Chr08	51,986,065	52,045,390	59,325	ESPLENDOR	Middle	Andean	50	50	40	-40
Chr08	51,986,065	52,045,390	59,325	PI 207262	Middle	Andean	50	44	36	-36
Chr08	51,986,065	52,045,390	59,325	México 54	Middle	Andean	50	47	41	-41
Chr08	11,560,315	11,619,151	58,836	TO	Middle	Andean	100	91	82	-82
Chr08	62,600,556	62,658,814	58,258	CB 911921	Middle	Andean	50	42	36	-36
Chr08	7,642,353	7,699,141	56,788	UIRAPURU	Middle	Andean	150	133	99	-99
Chr08	11,755,415	11,807,682	52,267	UIRAPURU	Middle	Andean	50	40	35	-35
Chr08	535,259	587,118	51,859	BRS Ametista	Middle	Andean	150	139	135	-135
Chr08	7,793,219	7,844,863	51,644	UIRAPURU	Middle	Andean	100	86	66	-66
Chr08	51,863,357	51,911,032	47,675	TO	Middle	Andean	50	50	42	-42
Chr08	51,863,357	51,911,032	47,675	ESPLENDOR	Middle	Andean	50	50	42	-42
Chr08	51,863,357	51,911,032	47,675	PI 207262	Middle	Andean	50	49	41	-41
Chr08	51,863,357	51,911,032	47,675	México 54	Middle	Andean	50	47	41	-41
Chr08	51,863,357	51,911,032	47,675	CB 911921	Middle	Andean	50	45	37	-37
Chr08	5,649,976	5,696,143	46,167	IPA 9	Middle	Andean	50	50	34	-34
Chr08	5,649,976	5,696,143	46,167	Sel 1308	Middle	Andean	50	49	35	-35
Chr08	5,649,976	5,696,143	46,167	PI 207262	Middle	Andean	50	48	31	-31
Chr08	5,649,976	5,696,143	46,167	US Pinto 111	Middle	Andean	50	41	33	-33
Chr08	5,649,976	5,696,143	46,167	México 54	Middle	Andean	50	48	32	-32
Chr08	5,649,976	5,696,143	46,167	ESTEIO	Middle	Andean	50	50	34	-34
Chr08	5,649,976	5,696,143	46,167	G2333	Middle	Andean	50	50	34	-34
Chr08	5,649,976	5,696,143	46,167	BRS Supremo	Middle	Andean	50	46	32	-32
Chr08	167,271	213,330	46,059	Rosinha G2	Middle	Andean	50	42	40	-40
Chr08	6,987,411	7,032,705	45,294	IPA 9	Middle	Andean	50	49	47	-47
Chr08	6,987,411	7,032,705	45,294	UIRAPURU	Middle	Andean	50	47	34	-34
Chr08	9,114,752	9,156,514	41,762	UIRAPURU	Middle	Andean	100	89	68	-68
Chr08	53,770,280	53,811,748	41,468	UIRAPURU	Middle	Andean	50	44	33	-33
Chr08	57,795,931	57,836,118	40,187	PI 207262	Middle	Andean	50	50	37	-37
Chr08	57,795,931	57,836,118	40,187	CB 911921	Middle	Andean	50	50	40	-40
Chr08	57,795,931	57,836,118	40,187	BRS Ametista	Middle	Andean	50	49	39	-39
Chr08	29,334,368	29,372,305	37,937	US Pinto 111	Middle	Andean	50	48	36	-36
Chr08	29,334,368	29,372,305	37,937	CB 911921	Middle	Andean	50	48	34	-34
Chr08	5,419,564	5,455,775	36,211	IAC Imperador	Middle	Andean	50	50	34	-34
Chr08	5,419,564	5,455,775	36,211	IPA 9	Middle	Andean	50	50	48	-48
Chr08	5,419,564	5,455,775	36,211	Sel 1308	Middle	Andean	50	48	46	-46
Chr08	5,419,564	5,455,775	36,211	US Pinto 111	Middle	Andean	50	46	44	-44
Chr08	5,419,564	5,455,775	36,211	México 54	Middle	Andean	50	49	46	-46
Chr08	5,419,564	5,455,775	36,211	ESTEIO	Middle	Andean	50	50	48	-48
Chr08	5,419,564	5,455,775	36,211	G2333	Middle	Andean	50	50	47	-47
Chr08	5,419,564	5,455,775	36,211	BRS Supremo	Middle	Andean	50	48	46	-46
Chr08	54,122,112	54,158,246	36,134	TO	Middle	Andean	50	46	34	-34

Chr	Start	End	Length	Sample	Background group	Haplotype group	Total variants	Genotyped variants	Score Andean	Score Middle
Chr08	54,122,112	54,158,246	36,134	PI 207262	Middle	Andean	50	50	36	-36
Chr08	54,122,112	54,158,246	36,134	IAC Diplomata	Middle	Andean	50	50	36	-36
Chr08	53,656,458	53,691,894	35,436	UIRAPURU	Middle	Andean	50	47	41	-41
Chr08	16,806,679	16,841,408	34,729	ESPLENDOR	Middle	Andean	150	150	134	-134
Chr08	16,806,679	16,841,408	34,729	PI 207262	Middle	Andean	150	141	126	-126
Chr08	16,806,679	16,841,408	34,729	US Pinto 111	Middle	Andean	150	135	121	-121
Chr08	16,806,679	16,841,408	34,729	México 54	Middle	Andean	150	143	127	-127
Chr08	16,806,679	16,841,408	34,729	G2333	Middle	Andean	150	148	126	-126
Chr08	16,806,679	16,841,408	34,729	Bat 332	Middle	Andean	150	146	131	-131
Chr08	16,806,679	16,841,408	34,729	MAIII 16159 (UFLA)	Middle	Andean	150	135	119	-119
Chr08	1,736,893	1,768,893	32,000	PEROLA	Middle	Andean	50	50	31	-31
Chr08	1,736,893	1,768,893	32,000	CB 911921	Middle	Andean	50	49	32	-32
Chr08	46,584,260	46,616,048	31,788	PI 207262	Middle	Andean	50	47	30	-30
Chr08	57,252,748	57,284,459	31,711	CB 911921	Middle	Andean	50	48	44	-44
Chr08	2,741,121	2,772,737	31,616	ESTILO	Middle	Andean	50	49	31	-31
Chr08	2,741,121	2,772,737	31,616	PEROLA	Middle	Andean	50	49	31	-31
Chr08	2,741,121	2,772,737	31,616	CNFC 10794	Middle	Andean	50	49	31	-31
Chr08	2,741,121	2,772,737	31,616	CB 911921	Middle	Andean	50	49	31	-31
Chr08	7,579,360	7,610,297	30,937	UIRAPURU	Middle	Andean	50	49	38	-38
Chr08	2,109,312	2,139,721	30,409	Sel 1308	Middle	Andean	50	49	47	-47
Chr08	53,205,479	53,235,412	29,933	CB 911921	Middle	Andean	50	48	32	-32
Chr08	11,590,399	11,619,151	28,752	CB 911921	Middle	Andean	50	48	44	-44
Chr08	1,003,805	1,029,182	25,377	ESPLENDOR	Middle	Andean	50	50	32	-32
Chr08	1,003,805	1,029,182	25,377	BRS Madrepérola	Middle	Andean	50	50	32	-32
Chr08	1,003,805	1,029,182	25,377	US Pinto 111	Middle	Andean	50	47	31	-31
Chr08	1,003,805	1,029,182	25,377	RUDA	Middle	Andean	50	50	32	-32
Chr08	1,003,805	1,029,182	25,377	IAC Diplomata	Middle	Andean	50	50	32	-32
Chr08	1,003,805	1,029,182	25,377	CNFC 16207	Middle	Andean	50	46	30	-30
Chr08	18,389,387	18,414,667	25,280	BRS Cometa	Middle	Andean	50	44	36	-36
Chr08	2,283,663	2,306,676	23,013	México 54	Middle	Andean	50	49	41	-41
Chr08	9,048,060	9,069,625	21,565	UIRAPURU	Middle	Andean	50	46	36	-36
Chr08	281,867	302,193	20,326	UIRAPURU	Middle	Andean	50	41	30	-30
Chr08	21,210,315	21,229,775	19,460	CB 911921	Middle	Andean	50	48	32	-32
Chr08	59,705,959	59,722,997	17,038	BRS Ártico (WAF 75)	Andean	Middle	50	49	-45	45
Chr08	59,705,959	59,722,997	17,038	BRS FS305 (CAL 96)	Andean	Middle	50	50	-46	46
Chr08	59,705,959	59,722,997	17,038	AND277	Andean	Middle	50	50	-46	46
Chr08	16,825,234	16,841,408	16,174	CNFC 16207	Middle	Andean	50	49	39	-39
Chr08	2,054,227	2,063,556	9,329	TO	Middle	Andean	50	48	38	-38
Chr08	2,054,227	2,063,556	9,329	Sel 1308	Middle	Andean	50	48	38	-38
Chr08	2,054,227	2,063,556	9,329	PI 207262	Middle	Andean	50	47	35	-35
Chr08	2,054,227	2,063,556	9,329	US Pinto 111	Middle	Andean	50	47	34	-34
Chr08	2,054,227	2,063,556	9,329	G2333	Middle	Andean	50	50	38	-38
Chr08	2,680,601	2,687,832	7,231	Sel 1308	Middle	Andean	50	46	34	-34
Chr08	16,806,679	16,811,985	5,306	CNFC 16207	Middle	Andean	50	40	38	-38
Chr09	241,347	8,753,486	8,512,139	MAIII 16159 (UFLA)	Middle	Andean	1600	1513	1485	-1485
Chr09	24,318,170	28,386,857	4,068,687	ESTEIO	Middle	Andean	2900	2900	2847	-2847
Chr09	20,838,013	22,481,014	1,643,001	IAC Imperador	Middle	Andean	500	496	445	-445
Chr09	15,322,380	16,868,217	1,545,837	MAIII 16159 (UFLA)	Middle	Andean	550	515	493	-493
Chr09	24,420,207	25,565,833	1,145,626	IAC Imperador	Middle	Andean	850	823	801	-801
Chr09	33,078,979	33,253,537	174,558	ESPLENDOR	Middle	Andean	150	149	113	-113
Chr09	33,078,979	33,253,537	174,558	México 54	Middle	Andean	150	130	99	-99
Chr09	33,755,807	33,897,532	141,725	México 54	Middle	Andean	100	93	75	-75
Chr09	29,166,474	29,305,462	138,988	BRS Ártico (WAF 75)	Andean	Middle	250	247	-198	198
Chr09	29,166,474	29,305,462	138,988	BRS Embaixador	Andean	Middle	250	247	-196	196
Chr09	29,166,474	29,305,462	138,988	AND277	Andean	Middle	250	250	-198	198
Chr09	14,783,059	14,896,300	113,241	PI 207262	Middle	Andean	100	98	87	-87
Chr09	14,545,176	14,648,179	103,003	México 54	Middle	Andean	150	142	118	-118
Chr09	22,092,398	22,179,272	86,874	IPA 9	Middle	Andean	50	50	32	-32
Chr09	22,092,398	22,179,272	86,874	Rosinha G2	Middle	Andean	50	46	32	-32
Chr09	22,092,398	22,179,272	86,874	US Pinto 111	Middle	Andean	50	47	33	-33
Chr09	33,168,438	33,253,537	85,099	PI 207262	Middle	Andean	50	46	35	-35
Chr09	33,168,438	33,253,537	85,099	US Pinto 111	Middle	Andean	50	48	37	-37
Chr09	33,168,438	33,253,537	85,099	G2333	Middle	Andean	50	50	40	-40
Chr09	30,442,241	30,520,937	78,696	MAIII 16159 (UFLA)	Middle	Andean	50	49	49	-49
Chr09	36,527,186	36,596,724	69,538	México 54	Middle	Andean	50	48	32	-32
Chr09	36,527,186	36,596,724	69,538	ESTEIO	Middle	Andean	50	50	30	-30
Chr09	36,527,186	36,596,724	69,538	FC104	Middle	Andean	50	50	30	-30
Chr09	36,527,186	36,596,724	69,538	CNFCT 19120	Middle	Andean	50	45	33	-33
Chr09	10,502,368	10,569,359	66,991	IAC Imperador	Middle	Andean	50	49	32	-32
Chr09	10,502,368	10,569,359	66,991	ESTEIO	Middle	Andean	50	50	34	-34
Chr09	10,502,368	10,569,359	66,991	FC104	Middle	Andean	50	50	34	-34
Chr09	10,502,368	10,569,359	66,991	BRS Sublime	Middle	Andean	50	49	33	-33
Chr09	10,502,368	10,569,359	66,991	PEROLA	Middle	Andean	50	50	34	-34
Chr09	10,502,368	10,569,359	66,991	Ouro Negro	Middle	Andean	50	49	33	-33
Chr09	10,502,368	10,569,359	66,991	CNFC 10467	Middle	Andean	50	50	34	-34
Chr09	10,502,368	10,569,359	66,991	BRS Ametista	Middle	Andean	50	49	32	-32

Chr	Start	End	Length	Sample	Background group	Haplotype group	Total variants	Genotyped variants	Score Andean	Score Middle
Chr09	14,591,183	14,648,179	56,996	Rosinha G2	Middle	Andean	50	42	40	-40
Chr09	14,591,183	14,648,179	56,996	PI 207262	Middle	Andean	50	49	46	-46
Chr09	14,591,183	14,648,179	56,996	BRS Horizonte	Middle	Andean	50	47	43	-43
Chr09	14,591,183	14,648,179	56,996	US Pinto 111	Middle	Andean	50	46	42	-42
Chr09	14,591,183	14,648,179	56,996	G2333	Middle	Andean	50	49	45	-45
Chr09	14,591,183	14,648,179	56,996	RUDA	Middle	Andean	50	50	46	-46
Chr09	14,591,183	14,648,179	56,996	MAIII 16159 (UFLA)	Middle	Andean	50	50	37	-37
Chr09	14,591,183	14,648,179	56,996	IAC Diplomata	Middle	Andean	50	50	46	-46
Chr09	14,591,183	14,648,179	56,996	CNFC 16207	Middle	Andean	50	46	42	-42
Chr09	14,591,183	14,648,179	56,996	BRS Ametista	Middle	Andean	50	47	45	-45
Chr09	33,078,979	33,124,467	45,488	TO	Middle	Andean	50	45	39	-39
Chr09	33,078,979	33,124,467	45,488	Rosinha G2	Middle	Andean	50	46	38	-38
Chr09	36,482,772	36,527,107	44,335	PI 207262	Middle	Andean	50	48	31	-31
Chr09	12,118,798	12,161,506	42,708	ESPLENDOR	Middle	Andean	50	50	43	-43
Chr09	12,118,798	12,161,506	42,708	Sel 1308	Middle	Andean	50	48	40	-40
Chr09	12,118,798	12,161,506	42,708	BRS Madrepérola	Middle	Andean	50	48	41	-41
Chr09	12,118,798	12,161,506	42,708	BRS Supremo	Middle	Andean	50	48	38	-38
Chr09	12,118,798	12,161,506	42,708	UIRAPURU	Middle	Andean	50	48	41	-41
Chr09	12,118,798	12,161,506	42,708	PEROLA	Middle	Andean	50	50	43	-43
Chr09	12,118,798	12,161,506	42,708	CNFC 10794	Middle	Andean	50	49	45	-45
Chr09	12,118,798	12,161,506	42,708	BRS Ametista	Middle	Andean	50	49	40	-40
Chr09	36,202,715	36,240,307	37,592	BRS Executivo	Andean	Middle	50	50	-30	30
Chr09	13,732,723	13,768,129	35,406	BRS Horizonte	Middle	Andean	50	46	30	-30
Chr09	13,732,723	13,768,129	35,406	US Pinto 111	Middle	Andean	50	46	30	-30
Chr09	13,732,723	13,768,129	35,406	México 54	Middle	Andean	50	48	34	-34
Chr09	13,732,723	13,768,129	35,406	G2333	Middle	Andean	50	50	34	-34
Chr09	13,732,723	13,768,129	35,406	RUDA	Middle	Andean	50	50	34	-34
Chr09	13,732,723	13,768,129	35,406	PEROLA	Middle	Andean	50	50	34	-34
Chr09	13,732,723	13,768,129	35,406	Ouro Negro	Middle	Andean	50	50	34	-34
Chr09	13,732,723	13,768,129	35,406	IAC Diplomata	Middle	Andean	50	50	34	-34
Chr09	13,732,723	13,768,129	35,406	CB 911921	Middle	Andean	50	49	33	-33
Chr09	36,743,935	36,778,620	34,685	ESPLENDOR	Middle	Andean	50	50	36	-36
Chr09	36,743,935	36,778,620	34,685	IPA 9	Middle	Andean	50	49	34	-34
Chr09	36,743,935	36,778,620	34,685	ESTILO	Middle	Andean	50	50	36	-36
Chr09	36,743,935	36,778,620	34,685	RUDA	Middle	Andean	50	50	36	-36
Chr09	36,743,935	36,778,620	34,685	PEROLA	Middle	Andean	50	50	36	-36
Chr09	36,743,935	36,778,620	34,685	Ouro Negro	Middle	Andean	50	50	36	-36
Chr09	36,743,935	36,778,620	34,685	CNFC 10794	Middle	Andean	50	50	36	-36
Chr09	36,743,935	36,778,620	34,685	CB 911921	Middle	Andean	50	50	36	-36
Chr09	32,889,758	32,921,311	31,553	TO	Middle	Andean	50	50	45	-45
Chr09	32,889,758	32,921,311	31,553	ESPLENDOR	Middle	Andean	50	50	44	-44
Chr09	32,889,758	32,921,311	31,553	Rosinha G2	Middle	Andean	50	49	45	-45
Chr09	14,749,894	14,781,145	31,251	Rosinha G2	Middle	Andean	50	47	34	-34
Chr09	14,749,894	14,781,145	31,251	México 54	Middle	Andean	50	48	34	-34
Chr09	14,749,894	14,781,145	31,251	RUDA	Middle	Andean	50	50	36	-36
Chr09	14,749,894	14,781,145	31,251	MAIII 16159 (UFLA)	Middle	Andean	50	47	33	-33
Chr09	14,749,894	14,781,145	31,251	IAC Diplomata	Middle	Andean	50	50	36	-36
Chr09	14,749,894	14,781,145	31,251	BRS Ametista	Middle	Andean	50	50	34	-34
Chr09	35,523,001	35,554,240	31,239	BRS Madrepérola	Middle	Andean	50	48	30	-30
Chr09	35,523,001	35,554,240	31,239	US Pinto 111	Middle	Andean	50	48	30	-30
Chr09	35,523,001	35,554,240	31,239	ESTEIO	Middle	Andean	50	50	30	-30
Chr09	36,859,389	36,890,137	30,748	BRSMG Realce	Andean	Middle	50	45	-33	33
Chr09	13,525,118	13,553,057	27,939	TO	Middle	Andean	50	44	44	-44
Chr09	13,525,118	13,553,057	27,939	IPA 9	Middle	Andean	50	47	47	-47
Chr09	13,525,118	13,553,057	27,939	Rosinha G2	Middle	Andean	50	44	44	-44
Chr09	13,525,118	13,553,057	27,939	ESTILO	Middle	Andean	50	50	50	-50
Chr09	13,525,118	13,553,057	27,939	RUDA	Middle	Andean	50	50	50	-50
Chr09	13,525,118	13,553,057	27,939	PEROLA	Middle	Andean	50	50	50	-50
Chr09	13,525,118	13,553,057	27,939	IAC Diplomata	Middle	Andean	50	50	50	-50
Chr09	13,525,118	13,553,057	27,939	CNFC 16207	Middle	Andean	50	40	40	-40
Chr09	13,525,118	13,553,057	27,939	CB 911921	Middle	Andean	50	45	45	-45
Chr09	13,525,118	13,553,057	27,939	BRS Ametista	Middle	Andean	50	50	50	-50
Chr09	28,230,653	28,257,964	27,311	IPA 9	Middle	Andean	50	49	43	-43
Chr09	28,230,653	28,257,964	27,311	PI 207262	Middle	Andean	50	50	44	-44
Chr09	28,230,653	28,257,964	27,311	BRS Madrepérola	Middle	Andean	50	47	41	-41
Chr09	28,230,653	28,257,964	27,311	México 54	Middle	Andean	50	49	43	-43
Chr09	10,195,131	10,219,975	24,844	PI 207262	Middle	Andean	50	48	38	-38
Chr09	10,195,131	10,219,975	24,844	PEROLA	Middle	Andean	50	50	35	-35
Chr09	10,195,131	10,219,975	24,844	Ouro Negro	Middle	Andean	50	50	35	-35
Chr09	30,089,475	30,110,708	21,233	Sel 1308	Middle	Andean	50	49	49	-49
Chr09	30,089,475	30,110,708	21,233	BRS Supremo	Middle	Andean	50	45	42	-42
Chr09	30,089,475	30,110,708	21,233	UIRAPURU	Middle	Andean	50	49	30	-30
Chr09	30,089,475	30,110,708	21,233	PEROLA	Middle	Andean	50	49	47	-47
Chr09	30,089,475	30,110,708	21,233	IAC Diplomata	Middle	Andean	50	49	47	-47
Chr09	30,089,475	30,110,708	21,233	CNFC 16207	Middle	Andean	50	48	47	-47
Chr09	13,069,842	13,088,503	18,661	BRS Executivo	Andean	Middle	50	50	-36	36

Chr	Start	End	Length	Sample	Background group	Haplotype group	Total variants	Genotyped variants	Score Andean	Score Middle
Chr09	13,069,842	13,088,503	18,661	BRS FS305 (CAL 96)	Andean	Middle	50	50	-37	37
Chr09	14,545,176	14,563,798	18,622	PI 207262	Middle	Andean	50	50	44	-44
Chr09	14,545,176	14,563,798	18,622	BRS Horizonte	Middle	Andean	50	46	40	-40
Chr09	14,545,176	14,563,798	18,622	US Pinto 111	Middle	Andean	50	48	42	-42
Chr09	14,545,176	14,563,798	18,622	G2333	Middle	Andean	50	50	44	-44
Chr09	14,545,176	14,563,798	18,622	RUDA	Middle	Andean	50	50	44	-44
Chr09	14,545,176	14,563,798	18,622	IAC Diplomata	Middle	Andean	50	50	44	-44
Chr09	14,545,176	14,563,798	18,622	CNFC 16207	Middle	Andean	50	48	42	-42
Chr09	14,545,176	14,563,798	18,622	BRS Ametista	Middle	Andean	50	48	42	-42
Chr09	13,818,381	13,833,870	15,489	México 54	Middle	Andean	50	44	30	-30
Chr09	13,818,381	13,833,870	15,489	RUDA	Middle	Andean	50	50	32	-32
Chr09	13,818,381	13,833,870	15,489	IAC Diplomata	Middle	Andean	50	50	32	-32
Chr09	13,818,381	13,833,870	15,489	CNFC 16207	Middle	Andean	50	49	31	-31
Chr09	36,345,703	36,360,354	14,651	BRSMG Realce	Andean	Middle	50	46	-45	45
Chr10	19,661,901	38,233,672	18,571,771	TO	Middle	Andean	2450	2257	2210	-2210
Chr10	19,661,901	36,264,435	16,602,534	BRS Cometa	Middle	Andean	1600	1472	1437	-1437
Chr10	5,059,554	17,799,276	12,739,722	TO	Middle	Andean	2050	1911	1858	-1858
Chr10	4,912,011	14,537,870	9,625,859	BRS Cometa	Middle	Andean	2150	2004	1955	-1955
Chr10	39,768,850	42,670,687	2,901,837	TO	Middle	Andean	2100	1908	1868	-1868
Chr10	12,119,803	13,983,460	1,863,657	Sel 1308	Middle	Andean	350	338	306	-306
Chr10	12,119,803	13,983,460	1,863,657	PEROLA	Middle	Andean	350	350	323	-323
Chr10	27,219,878	28,398,508	1,178,630	Sel 1308	Middle	Andean	250	245	217	-217
Chr10	27,219,878	28,398,508	1,178,630	PEROLA	Middle	Andean	250	249	218	-218
Chr10	22,586,198	23,632,535	1,046,337	Sel 1308	Middle	Andean	150	144	101	-101
Chr10	22,007,455	23,042,069	1,034,614	US Pinto 111	Middle	Andean	100	94	62	-62
Chr10	22,007,455	23,042,069	1,034,614	PEROLA	Middle	Andean	100	100	64	-64
Chr10	38,823,943	39,701,418	877,475	TO	Middle	Andean	600	557	525	-525
Chr10	43,181,217	43,921,281	740,064	IAC Imperador	Middle	Andean	600	588	576	-576
Chr10	24,136,407	24,874,026	737,619	Sel 1308	Middle	Andean	50	48	30	-30
Chr10	24,136,407	24,874,026	737,619	PI 207262	Middle	Andean	50	50	34	-34
Chr10	24,136,407	24,874,026	737,619	US Pinto 111	Middle	Andean	50	48	30	-30
Chr10	24,136,407	24,874,026	737,619	México 54	Middle	Andean	50	50	30	-30
Chr10	24,136,407	24,874,026	737,619	G2333	Middle	Andean	50	50	31	-31
Chr10	24,136,407	24,874,026	737,619	ESTILO	Middle	Andean	50	50	31	-31
Chr10	24,136,407	24,874,026	737,619	PEROLA	Middle	Andean	50	49	30	-30
Chr10	24,136,407	24,874,026	737,619	CNFC 10794	Middle	Andean	50	50	31	-31
Chr10	17,080,327	17,799,276	718,949	Sel 1308	Middle	Andean	50	48	41	-41
Chr10	17,080,327	17,799,276	718,949	PI 207262	Middle	Andean	50	49	41	-41
Chr10	17,080,327	17,799,276	718,949	US Pinto 111	Middle	Andean	50	45	42	-42
Chr10	17,080,327	17,799,276	718,949	México 54	Middle	Andean	50	50	42	-42
Chr10	17,080,327	17,799,276	718,949	G2333	Middle	Andean	50	49	42	-42
Chr10	17,080,327	17,799,276	718,949	ESTILO	Middle	Andean	50	50	43	-43
Chr10	17,080,327	17,799,276	718,949	PEROLA	Middle	Andean	50	50	43	-43
Chr10	17,080,327	17,799,276	718,949	Bat 332	Middle	Andean	50	49	41	-41
Chr10	17,080,327	17,799,276	718,949	CNFC 10794	Middle	Andean	50	49	40	-40
Chr10	17,080,327	17,799,276	718,949	BRS Cometa	Middle	Andean	50	49	49	-49
Chr10	10,735,997	11,226,478	490,481	PEROLA	Middle	Andean	100	98	62	-62
Chr10	3,623,317	4,081,197	457,880	ESPLENDOR	Middle	Andean	50	50	38	-38
Chr10	3,623,317	4,081,197	457,880	BRS Horizonte	Middle	Andean	50	48	36	-36
Chr10	3,623,317	4,081,197	457,880	US Pinto 111	Middle	Andean	50	44	36	-36
Chr10	3,623,317	4,081,197	457,880	CNFC 16207	Middle	Andean	50	46	34	-34
Chr10	22,586,198	23,042,069	455,871	PI 207262	Middle	Andean	50	50	33	-33
Chr10	22,586,198	23,042,069	455,871	México 54	Middle	Andean	50	48	32	-32
Chr10	22,586,198	23,042,069	455,871	G2333	Middle	Andean	50	49	35	-35
Chr10	22,586,198	23,042,069	455,871	ESTILO	Middle	Andean	50	50	34	-34
Chr10	22,586,198	23,042,069	455,871	Bat 332	Middle	Andean	50	50	33	-33
Chr10	22,586,198	23,042,069	455,871	CNFC 10794	Middle	Andean	50	50	33	-33
Chr10	4,081,261	4,491,615	410,354	BRS FS305 (CAL 96)	Andean	Middle	50	49	-31	31
Chr10	4,081,261	4,491,615	410,354	AND277	Andean	Middle	50	50	-32	32
Chr10	4,760,000	5,169,489	409,489	BRS FS305 (CAL 96)	Andean	Middle	450	447	-424	424
Chr10	4,760,000	5,169,489	409,489	AND277	Andean	Middle	450	450	-428	428
Chr10	4,560,579	4,877,871	317,292	BRS Cometa	Middle	Andean	350	328	321	-321
Chr10	27,219,878	27,536,830	316,952	PI 207262	Middle	Andean	50	48	30	-30
Chr10	27,219,878	27,536,830	316,952	CNFC 10794	Middle	Andean	50	48	32	-32
Chr10	10,735,997	11,038,073	302,076	G2333	Middle	Andean	50	50	33	-33
Chr10	426,382	632,092	205,710	BRS FS305 (CAL 96)	Andean	Middle	150	145	-131	131
Chr10	11,040,531	11,226,478	185,947	Sel 1308	Middle	Andean	50	49	31	-31
Chr10	11,040,531	11,226,478	185,947	US Pinto 111	Middle	Andean	50	46	30	-30
Chr10	4,514,812	4,681,227	166,415	BRS FS305 (CAL 96)	Andean	Middle	250	249	-193	193
Chr10	4,514,812	4,681,227	166,415	AND277	Andean	Middle	250	250	-194	194
Chr10	44,111,451	44,245,922	134,471	TO	Middle	Andean	100	94	92	-92
Chr10	9,021,130	9,141,407	120,277	Sel 1308	Middle	Andean	50	50	46	-46
Chr10	9,021,130	9,141,407	120,277	PEROLA	Middle	Andean	50	50	46	-46
Chr10	11,847,741	11,964,816	117,075	US Pinto 111	Middle	Andean	50	47	37	-37
Chr10	41,820,593	41,926,520	105,927	BRS FS305 (CAL 96)	Andean	Middle	150	150	-96	96
Chr10	41,820,593	41,926,520	105,927	AND277	Andean	Middle	150	150	-96	96

Chr	Start	End	Length	Sample	Background group	Haplotype group	Total variants	Genotyped variants	Score Andean	Score Middle
Chr10	2,909,549	2,985,149	75,600	IAC Imperador	Middle	Andean	50	47	31	-31
Chr10	2,909,549	2,985,149	75,600	PEROLA	Middle	Andean	50	50	34	-34
Chr10	39,705,150	39,768,840	63,690	US Pinto 111	Middle	Andean	50	47	32	-32
Chr10	39,705,150	39,768,840	63,690	RUDA	Middle	Andean	50	50	32	-32
Chr10	39,705,150	39,768,840	63,690	IAC Diplomata	Middle	Andean	50	50	32	-32
Chr10	44,183,980	44,245,922	61,942	FC104	Middle	Andean	50	50	30	-30
Chr10	42,583,390	42,643,075	59,685	ESPLENDOR	Middle	Andean	100	100	68	-68
Chr10	42,583,390	42,643,075	59,685	PI 207262	Middle	Andean	100	99	66	-66
Chr10	42,583,390	42,643,075	59,685	BRS Madrepérola	Middle	Andean	100	99	64	-64
Chr10	42,583,390	42,643,075	59,685	ESTILO	Middle	Andean	100	100	68	-68
Chr10	42,583,390	42,643,075	59,685	NOTAVEL	Middle	Andean	100	100	68	-68
Chr10	42,583,390	42,643,075	59,685	CNFC 10794	Middle	Andean	100	96	68	-68
Chr10	39,035,992	39,095,295	59,303	BRS FS305 (CAL 96)	Andean	Middle	50	50	-39	39
Chr10	39,035,992	39,095,295	59,303	AND277	Andean	Middle	50	50	-44	44
Chr10	2,465,241	2,522,632	57,391	TO	Middle	Andean	100	90	86	-86
Chr10	2,465,241	2,522,632	57,391	México 54	Middle	Andean	100	89	84	-84
Chr10	2,465,241	2,522,632	57,391	RUDA	Middle	Andean	100	100	96	-96
Chr10	2,465,241	2,522,632	57,391	NOTAVEL	Middle	Andean	100	100	96	-96
Chr10	2,465,241	2,522,632	57,391	IAC Diplomata	Middle	Andean	100	100	96	-96
Chr10	42,288,984	42,344,400	55,416	BRS Horizonte	Middle	Andean	50	49	43	-43
Chr10	42,288,984	42,344,400	55,416	US Pinto 111	Middle	Andean	50	48	42	-42
Chr10	42,288,984	42,344,400	55,416	G2333	Middle	Andean	50	50	44	-44
Chr10	38,823,943	38,873,538	49,595	BRS FS305 (CAL 96)	Andean	Middle	50	49	-31	31
Chr10	38,823,943	38,873,538	49,595	AND277	Andean	Middle	50	50	-30	30
Chr10	1,069,009	1,108,205	39,196	ESPLENDOR	Middle	Andean	100	100	70	-70
Chr10	1,069,009	1,108,205	39,196	PI 207262	Middle	Andean	100	100	66	-66
Chr10	1,069,009	1,108,205	39,196	G2333	Middle	Andean	100	97	66	-66
Chr10	1,069,009	1,108,205	39,196	ESTILO	Middle	Andean	100	100	70	-70
Chr10	1,069,009	1,108,205	39,196	PEROLA	Middle	Andean	100	100	64	-64
Chr10	2,465,241	2,502,764	37,523	Rosinha G2	Middle	Andean	50	42	38	-38
Chr10	36,491,165	36,525,989	34,824	BRS FS305 (CAL 96)	Andean	Middle	50	50	-44	44
Chr10	36,491,165	36,525,989	34,824	AND277	Andean	Middle	50	50	-44	44
Chr10	4,877,876	4,912,010	34,134	BRS Horizonte	Middle	Andean	50	47	33	-33
Chr10	42,612,032	42,643,075	31,043	BRS Cometa	Middle	Andean	50	42	30	-30
Chr10	43,149,978	43,180,861	30,883	ESPLENDOR	Middle	Andean	50	50	34	-34
Chr10	43,149,978	43,180,861	30,883	IPA 9	Middle	Andean	50	50	34	-34
Chr10	43,149,978	43,180,861	30,883	PI 207262	Middle	Andean	50	49	33	-33
Chr10	43,149,978	43,180,861	30,883	México 54	Middle	Andean	50	47	31	-31
Chr10	43,149,978	43,180,861	30,883	ESTILO	Middle	Andean	50	50	34	-34
Chr10	43,149,978	43,180,861	30,883	NOTAVEL	Middle	Andean	50	50	34	-34
Chr10	43,149,978	43,180,861	30,883	CNFC 10794	Middle	Andean	50	50	34	-34
Chr10	40,581,249	40,609,385	28,136	RUDA	Middle	Andean	50	50	40	-40
Chr10	40,581,249	40,609,385	28,136	IAC Diplomata	Middle	Andean	50	50	40	-40
Chr10	40,581,249	40,609,385	28,136	BRS Ametista	Middle	Andean	50	49	41	-41
Chr10	42,747,517	42,775,190	27,673	BRS Horizonte	Middle	Andean	50	49	42	-42
Chr10	42,747,517	42,775,190	27,673	US Pinto 111	Middle	Andean	50	48	42	-42
Chr10	42,747,517	42,775,190	27,673	ESTEIO	Middle	Andean	50	50	44	-44
Chr10	42,747,517	42,775,190	27,673	FC104	Middle	Andean	50	50	44	-44
Chr10	42,747,517	42,775,190	27,673	RUDA	Middle	Andean	50	50	44	-44
Chr10	7,213,792	7,240,825	27,033	IAC Imperador	Middle	Andean	50	50	44	-44
Chr10	7,213,792	7,240,825	27,033	Sel 1308	Middle	Andean	50	49	42	-42
Chr10	7,213,792	7,240,825	27,033	México 54	Middle	Andean	50	46	36	-36
Chr10	7,213,792	7,240,825	27,033	PEROLA	Middle	Andean	50	50	44	-44
Chr10	7,213,792	7,240,825	27,033	Bat 332	Middle	Andean	50	48	42	-42
Chr10	23,606,258	23,632,535	26,277	PI 207262	Middle	Andean	50	44	34	-34
Chr10	23,606,258	23,632,535	26,277	US Pinto 111	Middle	Andean	50	48	38	-38
Chr10	23,606,258	23,632,535	26,277	México 54	Middle	Andean	50	47	37	-37
Chr10	23,606,258	23,632,535	26,277	G2333	Middle	Andean	50	50	39	-39
Chr10	23,606,258	23,632,535	26,277	ESTILO	Middle	Andean	50	49	39	-39
Chr10	23,606,258	23,632,535	26,277	PEROLA	Middle	Andean	50	50	40	-40
Chr10	23,606,258	23,632,535	26,277	Bat 332	Middle	Andean	50	47	37	-37
Chr10	23,606,258	23,632,535	26,277	CNFC 10794	Middle	Andean	50	48	38	-38
Chr10	1,069,009	1,094,580	25,571	BRS Horizonte	Middle	Andean	50	47	36	-36
Chr10	1,069,009	1,094,580	25,571	US Pinto 111	Middle	Andean	50	50	38	-38
Chr10	1,069,009	1,094,580	25,571	México 54	Middle	Andean	50	47	37	-37
Chr10	1,069,009	1,094,580	25,571	CNFC 10794	Middle	Andean	50	48	38	-38
Chr10	1,069,009	1,094,580	25,571	BRS Ametista	Middle	Andean	50	49	33	-33
Chr10	42,645,584	42,670,687	25,103	México 54	Middle	Andean	50	49	35	-35
Chr10	43,899,667	43,921,281	21,614	ESPLENDOR	Middle	Andean	50	50	36	-36
Chr10	43,899,667	43,921,281	21,614	IPA 9	Middle	Andean	50	48	34	-34
Chr10	43,899,667	43,921,281	21,614	BRS Madrepérola	Middle	Andean	50	49	35	-35
Chr10	43,899,667	43,921,281	21,614	ESTEIO	Middle	Andean	50	50	35	-35
Chr10	43,899,667	43,921,281	21,614	FC104	Middle	Andean	50	50	34	-34
Chr10	43,899,667	43,921,281	21,614	NOTAVEL	Middle	Andean	50	50	36	-36
Chr10	2,351,470	2,372,229	20,759	IAC Imperador	Middle	Andean	50	50	37	-37
Chr10	2,351,470	2,372,229	20,759	IPA 9	Middle	Andean	50	50	36	-36

Chr	Start	End	Length	Sample	Background group	Haplotype group	Total variants	Genotyped variants	Score Andean	Score Middle
Chr10	2,351,470	2,372,229	20,759	Sel 1308	Middle	Andean	50	49	38	-38
Chr10	2,351,470	2,372,229	20,759	PEROLA	Middle	Andean	50	50	38	-38
Chr10	2,351,470	2,372,229	20,759	CB 911921	Middle	Andean	50	50	39	-39
Chr10	2,351,470	2,372,229	20,759	BRS Cometa	Middle	Andean	50	50	38	-38
Chr10	42,797,898	42,818,366	20,468	ESPLENDOR	Middle	Andean	50	50	44	-44
Chr10	42,797,898	42,818,366	20,468	IPA 9	Middle	Andean	50	50	44	-44
Chr10	42,797,898	42,818,366	20,468	PI 207262	Middle	Andean	50	48	42	-42
Chr10	42,797,898	42,818,366	20,468	BRS Madrepérola	Middle	Andean	50	50	44	-44
Chr10	42,797,898	42,818,366	20,468	México 54	Middle	Andean	50	50	44	-44
Chr10	42,797,898	42,818,366	20,468	ESTILO	Middle	Andean	50	50	44	-44
Chr10	42,797,898	42,818,366	20,468	NOTAVEL	Middle	Andean	50	50	44	-44
Chr10	42,797,898	42,818,366	20,468	CNFC 10794	Middle	Andean	50	50	44	-44
Chr10	39,109,984	39,127,202	17,218	BRS FS305 (CAL 96)	Andean	Middle	50	48	-44	44
Chr10	39,109,984	39,127,202	17,218	AND277	Andean	Middle	50	50	-46	46
Chr10	782,016	799,045	17,029	IAC Imperador	Middle	Andean	50	49	37	-37
Chr10	782,016	799,045	17,029	IPA 9	Middle	Andean	50	50	32	-32
Chr10	782,016	799,045	17,029	Sel 1308	Middle	Andean	50	50	32	-32
Chr10	782,016	799,045	17,029	BRS Horizonte	Middle	Andean	50	46	32	-32
Chr10	782,016	799,045	17,029	ESTILO	Middle	Andean	50	50	32	-32
Chr10	782,016	799,045	17,029	PEROLA	Middle	Andean	50	50	32	-32
Chr10	782,016	799,045	17,029	CNFC 10794	Middle	Andean	50	50	32	-32
Chr11	46,657,382	50,287,386	3,630,004	ESTILO	Middle	Andean	4200	4200	4150	-4150
Chr11	50,763,061	53,534,911	2,771,850	CB 911921	Middle	Andean	1300	1246	1222	-1222
Chr11	3,211,755	5,355,627	2,143,872	IAC Imperador	Middle	Andean	1450	1428	1395	-1395
Chr11	5,545,169	6,577,554	1,032,385	BRS FS305 (CAL 96)	Andean	Middle	750	744	-741	741
Chr11	52,220,653	53,116,453	895,800	IAC Imperador	Middle	Andean	500	481	456	-456
Chr11	37,695,031	38,218,318	523,287	RUDA	Middle	Andean	150	150	124	-124
Chr11	37,695,031	38,218,318	523,287	Ouro Negro	Middle	Andean	150	149	122	-122
Chr11	37,695,031	38,218,318	523,287	NOTAVEL	Middle	Andean	150	148	123	-123
Chr11	37,695,031	38,218,318	523,287	IAC Diplomata	Middle	Andean	150	150	124	-124
Chr11	37,695,031	38,218,318	523,287	CNFC 10467	Middle	Andean	150	149	120	-120
Chr11	959,676	1,462,848	503,172	IAC Imperador	Middle	Andean	1000	944	887	-887
Chr11	30,001,447	30,475,011	473,564	Ouro Negro	Middle	Andean	50	50	30	-30
Chr11	30,001,447	30,475,011	473,564	CNFC 10467	Middle	Andean	50	50	31	-31
Chr11	37,695,031	38,163,526	468,495	Rosinha G2	Middle	Andean	100	97	85	-85
Chr11	4,050,656	4,463,995	413,339	BRS FS305 (CAL 96)	Andean	Middle	250	246	-241	241
Chr11	42,106,457	42,507,764	401,307	Rosinha G2	Middle	Andean	100	83	62	-62
Chr11	42,106,457	42,507,764	401,307	RUDA	Middle	Andean	100	100	70	-70
Chr11	42,106,457	42,507,764	401,307	Ouro Negro	Middle	Andean	100	100	67	-67
Chr11	42,106,457	42,507,764	401,307	NOTAVEL	Middle	Andean	100	100	70	-70
Chr11	42,106,457	42,507,764	401,307	IAC Diplomata	Middle	Andean	100	100	70	-70
Chr11	42,106,457	42,507,764	401,307	CNFC 10467	Middle	Andean	100	98	69	-69
Chr11	35,255,991	35,577,515	321,524	RUDA	Middle	Andean	100	100	86	-86
Chr11	35,255,991	35,577,515	321,524	Ouro Negro	Middle	Andean	100	99	87	-87
Chr11	35,255,991	35,577,515	321,524	NOTAVEL	Middle	Andean	100	100	86	-86
Chr11	35,255,991	35,577,515	321,524	IAC Diplomata	Middle	Andean	100	100	86	-86
Chr11	35,255,991	35,577,515	321,524	CNFC 10467	Middle	Andean	100	93	80	-80
Chr11	45,765,208	46,076,117	310,909	ESPLENDOR	Middle	Andean	150	150	111	-111
Chr11	45,765,208	46,076,117	310,909	IPA 9	Middle	Andean	150	148	111	-111
Chr11	45,765,208	46,076,117	310,909	PI 207262	Middle	Andean	150	149	106	-106
Chr11	45,765,208	46,076,117	310,909	BRS Madrepérola	Middle	Andean	150	147	109	-109
Chr11	35,255,991	35,534,071	278,080	Rosinha G2	Middle	Andean	50	42	30	-30
Chr11	670,263	927,430	257,167	IAC Imperador	Middle	Andean	500	487	453	-453
Chr11	51,691,538	51,919,096	227,558	BRSMG Realce	Andean	Middle	50	41	-30	30
Chr11	3,569,950	3,750,486	180,536	FC104	Middle	Andean	200	198	147	-147
Chr11	8,830,779	8,985,338	154,559	BRS Ártico (WAF 75)	Andean	Middle	300	297	-243	243
Chr11	8,830,779	8,985,338	154,559	BRS Embaixador	Andean	Middle	300	293	-236	236
Chr11	8,830,779	8,985,338	154,559	AND277	Andean	Middle	300	300	-248	248
Chr11	4,934,527	5,084,377	149,850	ESPLENDOR	Middle	Andean	100	100	70	-70
Chr11	4,934,527	5,084,377	149,850	BRS Madrepérola	Middle	Andean	100	98	66	-66
Chr11	4,934,527	5,084,377	149,850	México 54	Middle	Andean	100	98	68	-68
Chr11	4,934,527	5,084,377	149,850	FC104	Middle	Andean	100	100	70	-70
Chr11	4,934,527	5,084,377	149,850	IAC Diplomata	Middle	Andean	100	100	70	-70
Chr11	1,103,883	1,252,197	148,314	OURO BRANCO	Andean	Middle	200	175	-153	153
Chr11	45,765,208	45,905,405	140,197	BRS Ametista	Middle	Andean	50	46	30	-30
Chr11	37,408,013	37,547,992	139,979	México 54	Middle	Andean	50	44	40	-40
Chr11	37,408,013	37,547,992	139,979	RUDA	Middle	Andean	50	50	33	-33
Chr11	37,408,013	37,547,992	139,979	Ouro Negro	Middle	Andean	50	47	32	-32
Chr11	37,408,013	37,547,992	139,979	NOTAVEL	Middle	Andean	50	50	32	-32
Chr11	37,408,013	37,547,992	139,979	IAC Diplomata	Middle	Andean	50	50	33	-33
Chr11	36,387,873	36,526,682	138,809	Rosinha G2	Middle	Andean	50	42	40	-40
Chr11	36,387,873	36,526,682	138,809	RUDA	Middle	Andean	50	50	46	-46
Chr11	36,387,873	36,526,682	138,809	Ouro Negro	Middle	Andean	50	50	45	-45
Chr11	36,387,873	36,526,682	138,809	NOTAVEL	Middle	Andean	50	50	46	-46
Chr11	36,387,873	36,526,682	138,809	IAC Diplomata	Middle	Andean	50	50	46	-46
Chr11	36,387,873	36,526,682	138,809	CNFC 10467	Middle	Andean	50	50	46	-46

Chr	Start	End	Length	Sample	Background group	Haplotype group	Total variants	Genotyped variants	Score Andean	Score Middle
Chr11	36,863,075	36,999,937	136,862	Rosinha G2	Middle	Andean	50	42	33	-33
Chr11	36,863,075	36,999,937	136,862	RUDA	Middle	Andean	50	50	41	-41
Chr11	36,863,075	36,999,937	136,862	NOTAVEL	Middle	Andean	50	50	41	-41
Chr11	36,863,075	36,999,937	136,862	IAC Diplomata	Middle	Andean	50	50	41	-41
Chr11	36,863,075	36,999,937	136,862	CNFC 10467	Middle	Andean	50	50	43	-43
Chr11	36,087,091	36,218,650	131,559	Rosinha G2	Middle	Andean	50	41	31	-31
Chr11	36,087,091	36,218,650	131,559	RUDA	Middle	Andean	50	50	40	-40
Chr11	36,087,091	36,218,650	131,559	Ouro Negro	Middle	Andean	50	49	39	-39
Chr11	36,087,091	36,218,650	131,559	NOTAVEL	Middle	Andean	50	50	40	-40
Chr11	36,087,091	36,218,650	131,559	IAC Diplomata	Middle	Andean	50	50	40	-40
Chr11	36,087,091	36,218,650	131,559	CNFC 10467	Middle	Andean	50	50	39	-39
Chr11	3,569,950	3,694,086	124,136	ESPLENDOR	Middle	Andean	150	148	114	-114
Chr11	3,569,950	3,694,086	124,136	Sel 1308	Middle	Andean	150	144	106	-106
Chr11	3,569,950	3,694,086	124,136	PI 207262	Middle	Andean	150	146	112	-112
Chr11	3,569,950	3,694,086	124,136	US Pinto 111	Middle	Andean	150	138	104	-104
Chr11	3,569,950	3,694,086	124,136	G2333	Middle	Andean	150	148	109	-109
Chr11	44,272,458	44,388,689	116,231	RUDA	Middle	Andean	100	100	75	-75
Chr11	44,272,458	44,388,689	116,231	NOTAVEL	Middle	Andean	100	100	76	-76
Chr11	44,272,458	44,388,689	116,231	IAC Diplomata	Middle	Andean	100	100	76	-76
Chr11	44,272,458	44,388,689	116,231	CNFC 10467	Middle	Andean	100	99	74	-74
Chr11	42,508,153	42,620,438	112,285	PI 207262	Middle	Andean	50	41	34	-34
Chr11	42,980,873	43,087,992	107,119	US Pinto 111	Middle	Andean	50	43	31	-31
Chr11	42,980,873	43,087,992	107,119	G2333	Middle	Andean	50	49	37	-37
Chr11	4,987,811	5,084,377	96,566	US Pinto 111	Middle	Andean	50	45	33	-33
Chr11	4,987,811	5,084,377	96,566	BRS Ametista	Middle	Andean	50	49	35	-35
Chr11	9,315,004	9,408,934	93,930	BRS Ártico (WAF 75)	Andean	Middle	100	96	-68	68
Chr11	9,315,004	9,408,934	93,930	BRS Embaixador	Andean	Middle	100	99	-70	70
Chr11	9,315,004	9,408,934	93,930	AND277	Andean	Middle	100	100	-72	72
Chr11	35,894,183	35,980,412	86,229	RUDA	Middle	Andean	50	50	34	-34
Chr11	35,894,183	35,980,412	86,229	Ouro Negro	Middle	Andean	50	50	33	-33
Chr11	35,894,183	35,980,412	86,229	NOTAVEL	Middle	Andean	50	50	34	-34
Chr11	35,894,183	35,980,412	86,229	IAC Diplomata	Middle	Andean	50	50	34	-34
Chr11	35,894,183	35,980,412	86,229	CNFC 10467	Middle	Andean	50	49	35	-35
Chr11	2,361,205	2,439,601	78,396	ESPLENDOR	Middle	Andean	50	48	36	-36
Chr11	2,361,205	2,439,601	78,396	IAC Diplomata	Middle	Andean	50	50	34	-34
Chr11	2,361,205	2,439,601	78,396	BRS Ametista	Middle	Andean	50	50	34	-34
Chr11	42,620,635	42,689,207	68,572	RUDA	Middle	Andean	50	50	34	-34
Chr11	42,620,635	42,689,207	68,572	Ouro Negro	Middle	Andean	50	49	32	-32
Chr11	42,620,635	42,689,207	68,572	NOTAVEL	Middle	Andean	50	50	34	-34
Chr11	42,620,635	42,689,207	68,572	IAC Diplomata	Middle	Andean	50	50	34	-34
Chr11	42,620,635	42,689,207	68,572	CNFC 10467	Middle	Andean	50	50	34	-34
Chr11	44,272,458	44,333,139	60,681	Rosinha G2	Middle	Andean	50	42	30	-30
Chr11	44,272,458	44,333,139	60,681	PI 207262	Middle	Andean	50	48	30	-30
Chr11	3,694,376	3,750,486	56,110	BRS FS305 (CAL 96)	Andean	Middle	50	48	-48	48
Chr11	45,565,828	45,619,973	54,145	ESPLENDOR	Middle	Andean	100	100	66	-66
Chr11	45,565,828	45,619,973	54,145	BRS Madrepérola	Middle	Andean	100	100	66	-66
Chr11	45,565,828	45,619,973	54,145	ESTEIO	Middle	Andean	100	100	66	-66
Chr11	45,565,828	45,619,973	54,145	Bat 332	Middle	Andean	100	100	66	-66
Chr11	45,565,828	45,619,973	54,145	MAIII 16159 (UFLA)	Middle	Andean	100	100	66	-66
Chr11	45,565,828	45,619,973	54,145	CB 911921	Middle	Andean	100	99	65	-65
Chr11	3,305,052	3,358,507	53,455	México 54	Middle	Andean	50	47	30	-30
Chr11	3,305,052	3,358,507	53,455	FC104	Middle	Andean	50	50	33	-33
Chr11	4,521,740	4,571,321	49,581	ESPLENDOR	Middle	Andean	50	50	42	-42
Chr11	4,521,740	4,571,321	49,581	BRS Madrepérola	Middle	Andean	50	49	41	-41
Chr11	4,521,740	4,571,321	49,581	FC104	Middle	Andean	50	49	43	-43
Chr11	4,521,740	4,571,321	49,581	IAC Diplomata	Middle	Andean	50	50	42	-42
Chr11	4,521,740	4,571,321	49,581	BRS Ametista	Middle	Andean	50	45	37	-37
Chr11	47,233,417	47,282,021	48,604	ESPLENDOR	Middle	Andean	50	50	38	-38
Chr11	47,233,417	47,282,021	48,604	IPA 9	Middle	Andean	50	49	37	-37
Chr11	47,233,417	47,282,021	48,604	BRS Madrepérola	Middle	Andean	50	50	38	-38
Chr11	47,233,417	47,282,021	48,604	México 54	Middle	Andean	50	50	38	-38
Chr11	47,233,417	47,282,021	48,604	BRS Ametista	Middle	Andean	50	45	38	-38
Chr11	46,767,175	46,815,503	48,328	TO	Middle	Andean	50	44	40	-40
Chr11	46,767,175	46,815,503	48,328	PI 207262	Middle	Andean	50	47	37	-37
Chr11	46,767,175	46,815,503	48,328	ESTEIO	Middle	Andean	50	50	46	-46
Chr11	46,767,175	46,815,503	48,328	BRS Supremo	Middle	Andean	50	50	46	-46
Chr11	46,767,175	46,815,503	48,328	Bat 332	Middle	Andean	50	50	46	-46
Chr11	46,767,175	46,815,503	48,328	MAIII 16159 (UFLA)	Middle	Andean	50	50	46	-46
Chr11	46,767,175	46,815,503	48,328	CB 911921	Middle	Andean	50	50	46	-46
Chr11	2,147,007	2,194,883	47,876	BRS FS305 (CAL 96)	Andean	Middle	200	200	-180	180
Chr11	48,001,598	48,043,042	41,444	CNFC 16207	Middle	Andean	50	47	30	-30
Chr11	46,035,506	46,076,117	40,611	México 54	Middle	Andean	50	47	45	-45
Chr11	46,035,506	46,076,117	40,611	BRS Ametista	Middle	Andean	50	45	41	-41
Chr11	3,858,015	3,895,018	37,003	BRS FS305 (CAL 96)	Andean	Middle	50	50	-42	42
Chr11	3,211,755	3,243,895	32,140	ESPLENDOR	Middle	Andean	50	50	44	-44
Chr11	3,211,755	3,243,895	32,140	PI 207262	Middle	Andean	50	50	44	-44

Chr	Start	End	Length	Sample	Background group	Haplotype group	Total variants	Genotyped variants	Score Andean	Score Middle
Chr11	3,211,755	3,243,895	32,140	US Pinto 111	Middle	Andean	50	40	36	-36
Chr11	3,211,755	3,243,895	32,140	FC104	Middle	Andean	50	50	44	-44
Chr11	45,565,828	45,596,665	30,837	TO	Middle	Andean	50	45	33	-33
Chr11	45,565,828	45,596,665	30,837	IPA 9	Middle	Andean	50	49	35	-35
Chr11	45,565,828	45,596,665	30,837	PI 207262	Middle	Andean	50	48	33	-33
Chr11	45,565,828	45,596,665	30,837	CNFC 16207	Middle	Andean	50	47	33	-33
Chr11	48,397,449	48,423,514	26,065	ESPLENDOR	Middle	Andean	50	50	42	-42
Chr11	48,397,449	48,423,514	26,065	IPA 9	Middle	Andean	50	49	41	-41
Chr11	48,397,449	48,423,514	26,065	PI 207262	Middle	Andean	50	48	40	-40
Chr11	48,397,449	48,423,514	26,065	BRS Madrepérola	Middle	Andean	50	45	37	-37
Chr11	48,397,449	48,423,514	26,065	ESTEIO	Middle	Andean	50	50	42	-42
Chr11	5,329,817	5,355,627	25,810	ESPLENDOR	Middle	Andean	50	50	50	-50
Chr11	5,329,817	5,355,627	25,810	BRS Madrepérola	Middle	Andean	50	50	50	-50
Chr11	5,329,817	5,355,627	25,810	US Pinto 111	Middle	Andean	50	44	41	-41
Chr11	5,329,817	5,355,627	25,810	México 54	Middle	Andean	50	46	46	-46
Chr11	5,329,817	5,355,627	25,810	G2333	Middle	Andean	50	50	50	-50
Chr11	5,329,817	5,355,627	25,810	FC104	Middle	Andean	50	50	50	-50
Chr11	5,329,817	5,355,627	25,810	IAC Diplomata	Middle	Andean	50	50	50	-50
Chr11	5,329,817	5,355,627	25,810	BRS Ametista	Middle	Andean	50	48	48	-48
Chr11	3,075,355	3,100,825	25,470	TO	Middle	Andean	50	47	41	-41
Chr11	3,075,355	3,100,825	25,470	ESTEIO	Middle	Andean	50	50	44	-44
Chr11	3,075,355	3,100,825	25,470	FC104	Middle	Andean	50	50	44	-44
Chr11	3,075,355	3,100,825	25,470	ESTILO	Middle	Andean	50	50	44	-44
Chr11	3,075,355	3,100,825	25,470	BRS Supremo	Middle	Andean	50	48	41	-41
Chr11	3,075,355	3,100,825	25,470	UIRAPURU	Middle	Andean	50	40	30	-30
Chr11	3,075,355	3,100,825	25,470	PEROLA	Middle	Andean	50	50	44	-44
Chr11	3,075,355	3,100,825	25,470	CNFC 10794	Middle	Andean	50	49	42	-42
Chr11	3,075,355	3,100,825	25,470	CB 911921	Middle	Andean	50	49	43	-43
Chr11	2,335,880	2,361,201	25,321	BRS FS305 (CAL 96)	Andean	Middle	50	50	-50	50
Chr11	2,310,909	2,335,261	24,352	ESPLENDOR	Middle	Andean	50	50	32	-32
Chr11	2,310,909	2,335,261	24,352	IAC Diplomata	Middle	Andean	50	50	32	-32
Chr11	2,310,909	2,335,261	24,352	BRS Ametista	Middle	Andean	50	49	31	-31
Chr11	3,131,738	3,156,010	24,272	BRS Madrepérola	Middle	Andean	50	46	30	-30
Chr11	3,131,738	3,156,010	24,272	RUDA	Middle	Andean	50	50	32	-32
Chr11	3,131,738	3,156,010	24,272	NOTAVEL	Middle	Andean	50	50	32	-32
Chr11	3,131,738	3,156,010	24,272	IAC Diplomata	Middle	Andean	50	50	32	-32
Chr11	3,131,738	3,156,010	24,272	CNFC 10467	Middle	Andean	50	49	31	-31
Chr11	3,131,738	3,156,010	24,272	BRS Ametista	Middle	Andean	50	48	32	-32
Chr11	45,597,152	45,619,973	22,821	ESTILO	Middle	Andean	50	50	34	-34
Chr11	50,763,061	50,784,271	21,210	BRSMG Realce	Andean	Middle	50	45	-38	38
Chr11	51,032,450	51,052,226	19,776	BRSMG Realce	Andean	Middle	50	41	-33	33
Chr11	2,253,499	2,271,510	18,011	BRS FS305 (CAL 96)	Andean	Middle	50	50	-38	38
Chr11	49,343,096	49,356,992	13,896	PI 207262	Middle	Andean	50	49	38	-38
Chr11	1,373,197	1,384,275	11,078	ESPLENDOR	Middle	Andean	50	50	34	-34
Chr11	1,373,197	1,384,275	11,078	BRS Madrepérola	Middle	Andean	50	49	32	-32
Chr11	1,373,197	1,384,275	11,078	IAC Diplomata	Middle	Andean	50	50	34	-34
Chr11	1,373,197	1,384,275	11,078	BRS Ametista	Middle	Andean	50	46	31	-31
Chr11	1,340,227	1,349,627	9,400	BRS Supremo	Middle	Andean	50	43	37	-37
Chr11	1,340,227	1,349,627	9,400	BRS Sublime	Middle	Andean	50	45	39	-39
Chr11	49,413,480	49,422,665	9,185	IAC Imperador	Middle	Andean	50	49	41	-41
Chr11	49,334,593	49,343,067	8,474	ESPLENDOR	Middle	Andean	50	50	40	-40
Chr11	49,334,593	49,343,067	8,474	IPA 9	Middle	Andean	50	49	39	-39
Chr11	49,334,593	49,343,067	8,474	BRS Madrepérola	Middle	Andean	50	48	38	-38
Chr11	49,334,593	49,343,067	8,474	México 54	Middle	Andean	50	50	39	-39
Chr11	49,334,593	49,343,067	8,474	ESTEIO	Middle	Andean	50	50	40	-40
Chr11	49,334,593	49,343,067	8,474	CNFC 10467	Middle	Andean	50	50	40	-40
Chr11	49,334,593	49,343,067	8,474	CB 911921	Middle	Andean	50	49	39	-39
Chr11	49,334,593	49,343,067	8,474	BRS Ametista	Middle	Andean	50	49	39	-39

Supplementary Table S8. Position and annotation of the 504 common bean disease genes retrieved at phytozome

Gene	Chrom	Start	End	Phytozome anotation
Phvul.001G042800	Chr01	4,050,181	4,052,709	disease_resistance_family_protein_/_LRR_family_protein
Phvul.001G043000	Chr01	4,030,665	4,033,321	disease_resistance_family_protein_/_LRR_family_protein
Phvul.001G062600	Chr01	7,506,465	7,510,678	LRR_and_NB-ARC_domains-containing_disease_resistance_protein
Phvul.001G093900	Chr01	16,463,979	16,469,620	disease_resistance_protein_(TIR-NBS-LRR_class),_putative
Phvul.001G128200	Chr01	35,568,119	35,575,452	disease_resistance_protein_(TIR-NBS-LRR_class),_putative
Phvul.001G132301	Chr01	36,668,704	36,670,649	NB-ARC_domain-containing_disease_resistance_protein
Phvul.001G132516	Chr01	36,711,067	36,713,855	NB-ARC_domain-containing_disease_resistance_protein
Phvul.001G132600	Chr01	36,843,403	36,846,206	LRR_and_NB-ARC_domains-containing_disease_resistance_protein
Phvul.001G132632	Chr01	36,746,099	36,748,726	NB-ARC_domain-containing_disease_resistance_protein
Phvul.001G132701	Chr01	36,886,262	36,888,199	NB-ARC_domain-containing_disease_resistance_protein
Phvul.001G132748	Chr01	36,754,526	36,762,677	NB-ARC_domain-containing_disease_resistance_protein
Phvul.001G132800	Chr01	36,921,109	36,923,833	NB-ARC_domain-containing_disease_resistance_protein
Phvul.001G132864	Chr01	36,778,384	36,786,193	NB-ARC_domain-containing_disease_resistance_protein
Phvul.001G132980	Chr01	36,798,913	36,800,852	disease_resistance_protein_(TIR-NBS-LRR_class)
Phvul.001G133000	Chr01	36,940,700	36,943,333	NB-ARC_domain-containing_disease_resistance_protein
Phvul.001G133100	Chr01	36,814,122	36,817,985	NB-ARC_domain-containing_disease_resistance_protein
Phvul.001G133101	Chr01	36,952,670	36,955,214	NB-ARC_domain-containing_disease_resistance_protein
Phvul.001G133400	Chr01	37,039,856	37,042,483	NB-ARC_domain-containing_disease_resistance_protein
Phvul.001G133601	Chr01	37,073,007	37,075,671	NB-ARC_domain-containing_disease_resistance_protein
Phvul.001G134000	Chr01	37,162,358	37,165,125	NB-ARC_domain-containing_disease_resistance_protein
Phvul.001G134100	Chr01	37,171,535	37,176,462	NB-ARC_domain-containing_disease_resistance_protein
Phvul.001G134300	Chr01	37,195,150	37,197,732	LRR_and_NB-ARC_domains-containing_disease_resistance_protein
Phvul.001G134400	Chr01	37,236,728	37,239,572	LRR_and_NB-ARC_domains-containing_disease_resistance_protein
Phvul.001G134500	Chr01	37,266,065	37,268,962	NB-ARC_domain-containing_disease_resistance_protein
Phvul.001G145000	Chr01	38,968,769	38,969,347	Disease_resistance-responsive_(dirigent-like_protein)_family_protein
Phvul.001G145100	Chr01	38,976,134	38,976,928	Disease_resistance-responsive_(dirigent-like_protein)_family_protein
Phvul.001G145200	Chr01	38,985,654	38,986,232	Disease_resistance-responsive_(dirigent-like_protein)_family_protein
Phvul.001G145300	Chr01	39,008,950	39,010,208	Disease_resistance-responsive_(dirigent-like_protein)_family_protein
Phvul.001G145500	Chr01	39,062,907	39,063,464	Disease_resistance-responsive_(dirigent-like_protein)_family_protein
Phvul.001G145600	Chr01	39,112,918	39,113,907	Disease_resistance-responsive_(dirigent-like_protein)_family_protein
Phvul.001G145700	Chr01	39,197,886	39,198,785	Disease_resistance-responsive_(dirigent-like_protein)_family_protein
Phvul.001G145800	Chr01	39,210,683	39,211,696	Disease_resistance-responsive_(dirigent-like_protein)_family_protein
Phvul.001G145900	Chr01	39,230,611	39,231,258	Disease_resistance-responsive_(dirigent-like_protein)_family_protein
Phvul.001G173000	Chr01	42,988,471	42,990,051	Disease_resistance-responsive_(dirigent-like_protein)_family_protein
Phvul.002G021700	Chr02	2,314,077	2,319,454	Disease_resistance_protein_(CC-NBS-LRR_class)_family
Phvul.002G074700	Chr02	11,217,976	11,220,600	NB-ARC_domain-containing_disease_resistance_protein
Phvul.002G075000	Chr02	11,366,868	11,369,872	NB-ARC_domain-containing_disease_resistance_protein
Phvul.002G075300	Chr02	11,407,609	11,410,626	NB-ARC_domain-containing_disease_resistance_protein
Phvul.002G098200	Chr02	19,961,334	19,966,696	disease_resistance_protein_(TIR-NBS-LRR_class),_putative
Phvul.002G115700	Chr02	24,770,509	24,771,096	Disease_resistance-responsive_(dirigent-like_protein)_family_protein
Phvul.002G129200	Chr02	27,169,060	27,172,869	NB-ARC_domain-containing_disease_resistance_protein
Phvul.002G129500	Chr02	27,235,064	27,238,243	NB-ARC_domain-containing_disease_resistance_protein
Phvul.002G129566	Chr02	27,257,427	27,258,875	LRR_and_NB-ARC_domains-containing_disease_resistance_protein
Phvul.002G129632	Chr02	27,259,041	27,260,780	NB-ARC_domain-containing_disease_resistance_protein
Phvul.002G129700	Chr02	27,268,820	27,272,399	LRR_and_NB-ARC_domains-containing_disease_resistance_protein
Phvul.002G129800	Chr02	27,290,920	27,291,258	NB-ARC_domain-containing_disease_resistance_protein
Phvul.002G129900	Chr02	27,295,732	27,299,541	NB-ARC_domain-containing_disease_resistance_protein
Phvul.002G130000	Chr02	27,306,626	27,310,531	NB-ARC_domain-containing_disease_resistance_protein
Phvul.002G130100	Chr02	27,318,168	27,321,670	NB-ARC_domain-containing_disease_resistance_protein
Phvul.002G130300	Chr02	27,338,024	27,340,744	NB-ARC_domain-containing_disease_resistance_protein
Phvul.002G130400	Chr02	27,367,582	27,370,366	NB-ARC_domain-containing_disease_resistance_protein
Phvul.002G130500	Chr02	27,398,103	27,401,760	NB-ARC_domain-containing_disease_resistance_protein
Phvul.002G130600	Chr02	27,418,264	27,425,398	NB-ARC_domain-containing_disease_resistance_protein
Phvul.002G130666	Chr02	27,445,769	27,447,599	LRR_and_NB-ARC_domains-containing_disease_resistance_protein
Phvul.002G130732	Chr02	27,447,674	27,449,203	NB-ARC_domain-containing_disease_resistance_protein
Phvul.002G131000	Chr02	27,489,779	27,493,234	LRR_and_NB-ARC_domains-containing_disease_resistance_protein
Phvul.002G131100	Chr02	27,498,551	27,502,193	LRR_and_NB-ARC_domains-containing_disease_resistance_protein

Gene	Chrom	Start	End	Phytozome anotation
Phvul.002G131200	Chr02	27,529,053	27,534,907	LRR_and_NB-ARC_domains-containing_disease_resistance_protein
Phvul.002G131500	Chr02	27,548,221	27,555,364	DZC_(Disease_resistance/zinc_finger/chromosome_condensation-like_region)_domain_containing_protein
Phvul.002G132000	Chr02	27,591,850	27,609,596	ENHANCED_DISEASE_RESISTANCE_2
Phvul.002G133400	Chr02	27,738,031	27,741,306	NB-ARC_domain-containing_disease_resistance_protein
Phvul.002G133600	Chr02	27,748,451	27,752,520	NB-ARC_domain-containing_disease_resistance_protein
Phvul.002G135100	Chr02	27,962,205	27,965,945	NB-ARC_domain-containing_disease_resistance_protein
Phvul.002G135650	Chr02	28,035,444	28,036,847	NB-ARC_domain-containing_disease_resistance_protein
Phvul.002G145600	Chr02	29,467,208	29,470,006	Disease_resistance_protein_(TIR-NBS-LRR_class)_family
Phvul.002G145700	Chr02	29,481,871	29,484,485	disease_resistance_protein_(TIR-NBS-LRR_class),_putative
Phvul.002G158300	Chr02	31,285,217	31,289,350	Disease_resistance_protein_(TIR-NBS_class)
Phvul.002G166400	Chr02	32,114,725	32,118,147	Disease_resistance_protein_(CC-NBS-LRR_class)_family
Phvul.002G171400	Chr02	32,705,205	32,709,817	disease_resistance_protein_(TIR-NBS-LRR_class),_putative
Phvul.002G291100	Chr02	45,974,920	45,979,447	Disease_resistance_protein_(TIR-NBS_class)
Phvul.002G294800	Chr02	46,387,359	46,388,032	Disease_resistance-responsive_(dirigent-like_protein)_family_protein
Phvul.002G294900	Chr02	46,395,250	46,395,921	Disease_resistance-responsive_(dirigent-like_protein)_family_protein
Phvul.002G323000	Chr02	48,786,657	48,794,177	disease_resistance_protein_(TIR-NBS-LRR_class),_putative
Phvul.002G323100	Chr02	48,796,609	48,803,146	disease_resistance_protein_(TIR-NBS-LRR_class),_putative
Phvul.002G323200	Chr02	48,805,820	48,810,839	disease_resistance_protein_(TIR-NBS-LRR_class)
Phvul.002G323300	Chr02	48,814,395	48,821,321	disease_resistance_protein_(TIR-NBS-LRR_class)
Phvul.002G323400	Chr02	48,827,778	48,834,046	disease_resistance_protein_(TIR-NBS-LRR_class)
Phvul.002G323404	Chr02	48,844,446	48,845,392	disease_resistance_protein_(TIR-NBS-LRR_class)
Phvul.002G323704	Chr02	48,862,995	48,868,411	disease_resistance_protein_(TIR-NBS-LRR_class)
Phvul.002G323708	Chr02	48,873,058	48,884,543	disease_resistance_protein_(TIR-NBS-LRR_class)
Phvul.002G323712	Chr02	48,888,578	48,895,769	disease_resistance_protein_(TIR-NBS-LRR_class)
Phvul.002G325400	Chr02	49,030,523	49,031,053	Disease_resistance-responsive_(dirigent-like_protein)_family_protein
Phvul.002G325500	Chr02	49,039,240	49,039,764	Disease_resistance-responsive_(dirigent-like_protein)_family_protein
Phvul.003G002300	Chr03	380,416	398,569	NB-ARC_domain-containing_disease_resistance_protein
Phvul.003G002400	Chr03	371,651	379,317	Disease_resistance_protein_(TIR-NBS-LRR_class)_family
Phvul.003G002600	Chr03	335,314	348,092	NB-ARC_domain-containing_disease_resistance_protein
Phvul.003G002700	Chr03	323,767	334,130	NB-ARC_domain-containing_disease_resistance_protein
Phvul.003G002926	Chr03	311,239	319,359	NB-ARC_domain-containing_disease_resistance_protein
Phvul.003G072400	Chr03	11,174,187	11,177,735	disease_resistance_protein_(TIR-NBS-LRR_class),_putative
Phvul.003G114200	Chr03	29,120,919	29,123,167	Disease_resistance-responsive_(dirigent-like_protein)_family_protein
Phvul.003G129700	Chr03	32,438,934	32,444,712	Disease_resistance-responsive_(dirigent-like_protein)_family_protein
Phvul.003G247200	Chr03	48,389,850	48,395,608	Disease_resistance_protein_(CC-NBS-LRR_class)_family
Phvul.003G247300	Chr03	48,400,642	48,403,758	Disease_resistance_protein_(CC-NBS-LRR_class)_family
Phvul.003G247400	Chr03	48,409,980	48,413,080	Disease_resistance_protein_(CC-NBS-LRR_class)_family
Phvul.003G247500	Chr03	48,419,142	48,423,051	Disease_resistance_protein_(CC-NBS-LRR_class)_family
Phvul.003G247550	Chr03	48,431,827	48,437,545	Disease_resistance_protein_(CC-NBS-LRR_class)_family
Phvul.003G247601	Chr03	48,450,911	48,455,276	Disease_resistance_protein_(CC-NBS-LRR_class)_family
Phvul.003G247651	Chr03	48,459,023	48,463,312	Disease_resistance_protein_(CC-NBS-LRR_class)_family
Phvul.003G252100	Chr03	49,124,934	49,125,774	Disease_resistance-responsive_(dirigent-like_protein)_family_protein
Phvul.003G269200	Chr03	50,682,731	50,686,033	disease_resistance_protein_(TIR-NBS-LRR_class),_putative
Phvul.003G269500	Chr03	50,717,198	50,717,755	Disease_resistance-responsive_(dirigent-like_protein)_family_protein
Phvul.003G279500	Chr03	51,680,520	51,686,288	DZC_(Disease_resistance/zinc_finger/chromosome_condensation-like_region)_domain_containing_protein
Phvul.004G005600	Chr04	345,784	350,309	NB-ARC_domain-containing_disease_resistance_protein
Phvul.004G007750	Chr04	567,896	570,454	NB-ARC_domain-containing_disease_resistance_protein
Phvul.004G007900	Chr04	618,293	622,960	NB-ARC_domain-containing_disease_resistance_protein
Phvul.004G008001	Chr04	634,170	639,473	NB-ARC_domain-containing_disease_resistance_protein
Phvul.004G008101	Chr04	657,081	659,750	NB-ARC_domain-containing_disease_resistance_protein
Phvul.004G008200	Chr04	671,773	674,107	NB-ARC_domain-containing_disease_resistance_protein
Phvul.004G008250	Chr04	677,817	680,151	LRR_and_NB-ARC_domains-containing_disease_resistance_protein
Phvul.004G008351	Chr04	706,950	711,423	NB-ARC_domain-containing_disease_resistance_protein
Phvul.004G008400	Chr04	732,245	733,453	LRR_and_NB-ARC_domains-containing_disease_resistance_protein
Phvul.004G008450	Chr04	733,519	734,080	NB-ARC_domain-containing_disease_resistance_protein
Phvul.004G008560	Chr04	747,914	753,358	LRR_and_NB-ARC_domains-containing_disease_resistance_protein
Phvul.004G008620	Chr04	762,295	765,654	NB-ARC_domain-containing_disease_resistance_protein
Phvul.004G008680	Chr04	768,791	773,818	NB-ARC_domain-containing_disease_resistance_protein
Phvul.004G008740	Chr04	796,775	801,277	NB-ARC_domain-containing_disease_resistance_protein
Phvul.004G008900	Chr04	1,129,267	1,132,629	NB-ARC_domain-containing_disease_resistance_protein
Phvul.004G008909	Chr04	1,137,597	1,139,195	NB-ARC_domain-containing_disease_resistance_protein
Phvul.004G008918	Chr04	1,146,369	1,149,704	LRR_and_NB-ARC_domains-containing_disease_resistance_protein
Phvul.004G008921	Chr04	819,458	826,737	LRR_and_NB-ARC_domains-containing_disease_resistance_protein
Phvul.004G008981	Chr04	833,532	836,825	NB-ARC_domain-containing_disease_resistance_protein
Phvul.004G009041	Chr04	838,678	843,077	LRR_and_NB-ARC_domains-containing_disease_resistance_protein

Gene	Chrom	Start	End	Phytozome anotation
Phvul.004G009100	Chr04	1,043,536	1,044,075	NB-ARC_domain-containing_disease_resistance_protein
Phvul.004G009101	Chr04	847,290	848,413	LRR_and_NB-ARC_domains-containing_disease_resistance_protein
Phvul.004G009136	Chr04	1,063,726	1,067,743	NB-ARC_domain-containing_disease_resistance_protein
Phvul.004G009154	Chr04	1,103,140	1,118,146	NB-ARC_domain-containing_disease_resistance_protein
Phvul.004G009221	Chr04	863,672	865,511	LRR_and_NB-ARC_domains-containing_disease_resistance_protein
Phvul.004G009281	Chr04	879,439	884,653	NB-ARC_domain-containing_disease_resistance_protein
Phvul.004G009300	Chr04	1,156,680	1,160,125	NB-ARC_domain-containing_disease_resistance_protein
Phvul.004G009461	Chr04	906,948	907,805	NB-ARC_domain-containing_disease_resistance_protein
Phvul.004G009500	Chr04	1,172,345	1,176,922	NB-ARC_domain-containing_disease_resistance_protein
Phvul.004G009518	Chr04	1,191,387	1,192,524	LRR_and_NB-ARC_domains-containing_disease_resistance_protein
Phvul.004G009521	Chr04	909,231	916,054	NB-ARC_domain-containing_disease_resistance_protein
Phvul.004G009527	Chr04	1,203,535	1,207,848	NB-ARC_domain-containing_disease_resistance_protein
Phvul.004G009581	Chr04	920,780	922,280	LRR_and_NB-ARC_domains-containing_disease_resistance_protein
Phvul.004G009800	Chr04	1,228,652	1,233,028	NB-ARC_domain-containing_disease_resistance_protein
Phvul.004G009821	Chr04	939,934	943,174	NB-ARC_domain-containing_disease_resistance_protein
Phvul.004G009909	Chr04	961,070	964,494	LRR_and_NB-ARC_domains-containing_disease_resistance_protein
Phvul.004G009918	Chr04	964,943	966,979	NB-ARC_domain-containing_disease_resistance_protein
Phvul.004G009927	Chr04	967,700	972,035	NB-ARC_domain-containing_disease_resistance_protein
Phvul.004G009936	Chr04	989,654	993,499	LRR_and_NB-ARC_domains-containing_disease_resistance_protein
Phvul.004G012801	Chr04	1,430,069	1,433,227	NB-ARC_domain-containing_disease_resistance_protein
Phvul.004G012900	Chr04	1,434,735	1,440,120	NB-ARC_domain-containing_disease_resistance_protein
Phvul.004G013000	Chr04	1,450,903	1,456,425	NB-ARC_domain-containing_disease_resistance_protein
Phvul.004G013100	Chr04	1,461,865	1,466,462	NB-ARC_domain-containing_disease_resistance_protein
Phvul.004G013200	Chr04	1,482,045	1,486,960	NB-ARC_domain-containing_disease_resistance_protein
Phvul.004G013300	Chr04	1,489,645	1,493,416	NB-ARC_domain-containing_disease_resistance_protein
Phvul.004G013350	Chr04	1,506,401	1,512,428	NB-ARC_domain-containing_disease_resistance_protein
Phvul.004G015600	Chr04	1,678,112	1,683,180	NB-ARC_domain-containing_disease_resistance_protein
Phvul.004G015666	Chr04	1,685,085	1,687,198	LRR_and_NB-ARC_domains-containing_disease_resistance_protein
Phvul.004G015732	Chr04	1,687,342	1,689,053	NB-ARC_domain-containing_disease_resistance_protein
Phvul.004G015800	Chr04	1,718,700	1,734,046	NB-ARC_domain-containing_disease_resistance_protein
Phvul.004G015900	Chr04	1,726,367	1,726,954	NB-ARC_domain-containing_disease_resistance_protein
Phvul.004G016000	Chr04	1,735,410	1,741,124	NB-ARC_domain-containing_disease_resistance_protein
Phvul.004G016532	Chr04	1,795,047	1,799,504	NB-ARC_domain-containing_disease_resistance_protein
Phvul.004G028900	Chr04	3,435,360	3,439,245	Disease_resistance_protein_(TIR-NBS-LRR_class)_family
Phvul.004G036200	Chr04	4,228,884	4,232,558	NB-ARC_domain-containing_disease_resistance_protein
Phvul.004G036300	Chr04	4,235,034	4,239,570	NB-ARC_domain-containing_disease_resistance_protein
Phvul.004G036600	Chr04	4,260,075	4,263,947	NB-ARC_domain-containing_disease_resistance_protein
Phvul.004G036700	Chr04	4,267,564	4,270,968	NB-ARC_domain-containing_disease_resistance_protein
Phvul.004G036800	Chr04	4,273,495	4,278,920	NB-ARC_domain-containing_disease_resistance_protein
Phvul.004G036900	Chr04	4,281,080	4,285,120	NB-ARC_domain-containing_disease_resistance_protein
Phvul.004G044301	Chr04	5,248,943	5,251,301	NB-ARC_domain-containing_disease_resistance_protein
Phvul.004G044800	Chr04	5,299,149	5,302,463	disease_resistance_family_protein_/_LRR_family_protein
Phvul.004G044900	Chr04	5,313,005	5,316,547	disease_resistance_family_protein_/_LRR_family_protein
Phvul.004G046400	Chr04	5,566,355	5,569,927	disease_resistance_protein_(TIR-NBS-LRR_class),_putative
Phvul.004G048000	Chr04	5,828,215	5,843,631	NB-ARC_domain-containing_disease_resistance_protein
Phvul.004G053000	Chr04	7,037,305	7,038,943	NB-ARC_domain-containing_disease_resistance_protein
Phvul.004G058700	Chr04	8,165,840	8,169,721	disease_resistance_protein_(TIR-NBS-LRR_class),_putative
Phvul.004G066125	Chr04	9,774,734	9,777,608	NB-ARC_domain-containing_disease_resistance_protein
Phvul.004G075900	Chr04	13,090,090	13,093,485	NB-ARC_domain-containing_disease_resistance_protein
Phvul.004G076001	Chr04	13,108,566	13,108,961	NB-ARC_domain-containing_disease_resistance_protein
Phvul.004G076100	Chr04	13,111,962	13,114,661	NB-ARC_domain-containing_disease_resistance_protein
Phvul.004G084600	Chr04	20,304,661	20,308,569	disease_resistance_family_protein_/_LRR_family_protein
Phvul.004G094094	Chr04	27,044,130	27,048,091	disease_resistance_family_protein_/_LRR_family_protein
Phvul.004G095900	Chr04	15,429,464	15,431,344	disease_resistance_family_protein_/_LRR_family_protein
Phvul.004G096200	Chr04	15,700,870	15,704,262	disease_resistance_family_protein_/_LRR_family_protein
Phvul.004G096400	Chr04	15,785,898	15,790,443	disease_resistance_family_protein_/_LRR_family_protein
Phvul.004G099300	Chr04	30,768,823	30,772,357	disease_resistance_family_protein_/_LRR_family_protein
Phvul.004G099400	Chr04	30,786,229	30,788,822	disease_resistance_family_protein_/_LRR_family_protein
Phvul.004G099700	Chr04	30,970,336	30,972,669	disease_resistance_family_protein_/_LRR_family_protein
Phvul.004G099900	Chr04	33,661,914	33,665,612	disease_resistance_family_protein_/_LRR_family_protein
Phvul.004G100000	Chr04	33,687,765	33,691,079	disease_resistance_family_protein_/_LRR_family_protein
Phvul.004G100014	Chr04	33,696,352	33,708,373	disease_resistance_family_protein_/_LRR_family_protein
Phvul.004G100200	Chr04	33,728,983	33,732,496	disease_resistance_family_protein_/_LRR_family_protein
Phvul.004G100300	Chr04	33,779,782	33,782,695	disease_resistance_family_protein_/_LRR_family_protein
Phvul.004G103000	Chr04	33,501,154	33,506,139	disease_resistance_family_protein_/_LRR_family_protein
Phvul.004G103200	Chr04	33,445,670	33,448,400	disease_resistance_family_protein_/_LRR_family_protein
Phvul.004G103300	Chr04	33,337,926	33,341,472	disease_resistance_family_protein_/_LRR_family_protein
Phvul.004G103600	Chr04	33,088,618	33,093,955	disease_resistance_family_protein_/_LRR_family_protein
Phvul.004G104700	Chr04	32,418,499	32,422,098	disease_resistance_family_protein_/_LRR_family_protein
Phvul.004G104900	Chr04	31,654,840	31,658,444	disease_resistance_family_protein_/_LRR_family_protein
Phvul.004G105100	Chr04	29,885,064	29,888,921	disease_resistance_family_protein_/_LRR_family_protein
Phvul.004G105400	Chr04	31,162,290	31,165,715	disease_resistance_family_protein_/_LRR_family_protein
Phvul.004G105501	Chr04	31,450,019	31,456,746	disease_resistance_family_protein_/_LRR_family_protein
Phvul.004G105600	Chr04	31,516,409	31,519,187	disease_resistance_family_protein_/_LRR_family_protein
Phvul.004G114700	Chr04	39,678,596	39,681,553	disease_resistance_family_protein_/_LRR_family_protein

Gene	Chrom	Start	End	Phytozome anotation
Phvul.004G114800	Chr04	39,697,982	39,699,957	disease_resistance_family_protein_/_LRR_family_protein
Phvul.004G114900	Chr04	39,705,233	39,706,334	disease_resistance_family_protein_/_LRR_family_protein
Phvul.004G115100	Chr04	39,731,138	39,733,508	disease_resistance_family_protein_/_LRR_family_protein
Phvul.004G115300	Chr04	39,835,618	39,840,026	disease_resistance_family_protein_/_LRR_family_protein
Phvul.004G115400	Chr04	39,871,813	39,875,207	disease_resistance_family_protein_/_LRR_family_protein
Phvul.004G115600	Chr04	39,931,719	39,934,538	disease_resistance_family_protein_/_LRR_family_protein
Phvul.004G115700	Chr04	39,956,633	39,959,521	disease_resistance_family_protein_/_LRR_family_protein
Phvul.004G116600	Chr04	40,074,548	40,077,622	disease_resistance_family_protein_/_LRR_family_protein
Phvul.004G134300	Chr04	43,296,842	43,301,022	disease_resistance_protein_(TIR-NBS-LRR_class),_putative
Phvul.004G135100	Chr04	43,429,334	43,436,923	disease_resistance_protein_(TIR-NBS-LRR_class),_putative
Phvul.004G136800	Chr04	43,645,253	43,645,726	Disease_resistance_protein_(TIR-NBS-LRR_class)_family
Phvul.004G137300	Chr04	43,694,998	43,701,041	Disease_resistance_protein_(TIR-NBS-LRR_class)_family
Phvul.004G139700	Chr04	43,948,712	43,952,664	Disease_resistance_protein_(TIR-NBS-LRR_class)_family
Phvul.004G139800	Chr04	43,966,778	43,970,654	disease_resistance_protein_(TIR-NBS-LRR_class),_putative
Phvul.004G139900	Chr04	43,979,236	43,989,728	disease_resistance_protein_(TIR-NBS-LRR_class),_putative
Phvul.004G140100	Chr04	43,996,431	44,000,472	disease_resistance_protein_(TIR-NBS-LRR_class),_putative
Phvul.004G140400	Chr04	44,023,931	44,026,000	disease_resistance_protein_(TIR-NBS-LRR_class),_putative
Phvul.004G140500	Chr04	44,028,764	44,036,048	disease_resistance_protein_(TIR-NBS-LRR_class),_putative
Phvul.004G140700	Chr04	44,043,302	44,047,287	disease_resistance_protein_(TIR-NBS-LRR_class),_putative
Phvul.004G140800	Chr04	44,053,656	44,057,736	disease_resistance_protein_(TIR-NBS-LRR_class),_putative
Phvul.005G007200	Chr05	588,339	590,099	disease_resistance_family_protein_/_LRR_family_protein
Phvul.005G014200	Chr05	1,337,185	1,343,069	NB-ARC_domain-containing_disease_resistance_protein
Phvul.005G014701	Chr05	1,227,809	1,232,595	NB-ARC_domain-containing_disease_resistance_protein
Phvul.005G016500	Chr05	1,443,971	1,449,481	disease_resistance_protein_(TIR-NBS-LRR_class),_putative
Phvul.005G027200	Chr05	2,531,034	2,534,630	NB-ARC_domain-containing_disease_resistance_protein
Phvul.005G030500	Chr05	2,821,209	2,825,448	disease_resistance_protein_(TIR-NBS-LRR_class),_putative
Phvul.005G031200	Chr05	2,895,711	2,899,880	LRR_and_NB-ARC_domains-containing_disease_resistance_protein
Phvul.005G032400	Chr05	2,993,278	2,993,718	Disease_resistance-responsive_(dirigent-like_protein)_family_protein
Phvul.005G032500	Chr05	3,004,781	3,005,350	Disease_resistance-responsive_(dirigent-like_protein)_family_protein
Phvul.005G032600	Chr05	3,005,604	3,006,439	Disease_resistance-responsive_(dirigent-like_protein)_family_protein
Phvul.005G032700	Chr05	3,014,210	3,015,142	Disease_resistance-responsive_(dirigent-like_protein)_family_protein
Phvul.005G062300	Chr05	9,137,989	9,142,030	Disease_resistance_protein_(CC-NBS-LRR_class)_family
Phvul.005G087100	Chr05	27,066,056	27,069,037	NB-ARC_domain-containing_disease_resistance_protein
Phvul.005G093400	Chr05	29,068,328	29,070,000	disease_resistance_protein_(TIR-NBS-LRR_class)
Phvul.005G117900	Chr05	35,309,250	35,314,337	NB-ARC_domain-containing_disease_resistance_protein
Phvul.006G001500	Chr06	439,226	441,872	disease_resistance_family_protein_/_LRR_family_protein
Phvul.006G003400	Chr06	3,033,325	3,035,007	disease_resistance_family_protein_/_LRR_family_protein
Phvul.006G018911	Chr06	4,347,888	4,349,636	LRR_and_NB-ARC_domains-containing_disease_resistance_protein
Phvul.006G019022	Chr06	4,349,690	4,351,601	NB-ARC_domain-containing_disease_resistance_protein
Phvul.006G019300	Chr06	4,251,555	4,255,792	disease_resistance_protein_(TIR-NBS-LRR_class),_putative
Phvul.006G038100	Chr06	13,989,213	13,990,910	NB-ARC_domain-containing_disease_resistance_protein
Phvul.006G041400	Chr06	13,540,288	13,545,325	NB-ARC_domain-containing_disease_resistance_protein
Phvul.006G045400	Chr06	14,924,175	14,928,537	NB-ARC_domain-containing_disease_resistance_protein
Phvul.006G051500	Chr06	15,625,130	15,628,201	NB-ARC_domain-containing_disease_resistance_protein
Phvul.006G052300	Chr06	15,687,137	15,690,481	NB-ARC_domain-containing_disease_resistance_protein
Phvul.006G052400	Chr06	15,697,039	15,701,289	NB-ARC_domain-containing_disease_resistance_protein
Phvul.006G052500	Chr06	15,715,642	15,718,314	NB-ARC_domain-containing_disease_resistance_protein
Phvul.006G052600	Chr06	15,734,265	15,744,891	NB-ARC_domain-containing_disease_resistance_protein
Phvul.006G056500	Chr06	16,323,530	16,325,642	NB-ARC_domain-containing_disease_resistance_protein
Phvul.006G066800	Chr06	17,835,035	17,838,657	NB-ARC_domain-containing_disease_resistance_protein
Phvul.006G188700	Chr06	28,897,165	28,902,481	disease_resistance_protein_(TIR_class),_putative
Phvul.007G070400	Chr07	6,498,346	6,501,177	disease_resistance_family_protein_/_LRR_family_protein
Phvul.007G077500	Chr07	7,408,695	7,411,352	disease_resistance_family_protein_/_LRR_family_protein
Phvul.007G086300	Chr07	8,714,332	8,718,886	LRR_and_NB-ARC_domains-containing_disease_resistance_protein
Phvul.007G087200	Chr07	8,883,450	8,886,262	disease_resistance_family_protein_/_LRR_family_protein
Phvul.007G087300	Chr07	8,892,399	8,894,864	disease_resistance_family_protein_/_LRR_family_protein
Phvul.007G087500	Chr07	8,901,763	8,904,340	disease_resistance_family_protein_/_LRR_family_protein
Phvul.007G087550	Chr07	8,909,309	8,915,952	disease_resistance_family_protein_/_LRR_family_protein
Phvul.007G087800	Chr07	8,961,279	8,964,683	disease_resistance_family_protein_/_LRR_family_protein
Phvul.007G112200	Chr07	17,864,100	17,866,855	NB-ARC_domain-containing_disease_resistance_protein
Phvul.007G135201	Chr07	21,184,778	21,187,826	disease_resistance_family_protein_/_LRR_family_protein
Phvul.007G160901	Chr07	27,214,200	27,220,354	disease_resistance_protein_(TIR-NBS-LRR_class),_putative
Phvul.007G238100	Chr07	36,202,757	36,203,500	Disease_resistance-responsive_(dirigent-like_protein)_family_protein
Phvul.007G238200	Chr07	36,204,941	36,205,843	Disease_resistance-responsive_(dirigent-like_protein)_family_protein
Phvul.007G246600	Chr07	36,975,500	36,978,572	disease_resistance_family_protein_/_LRR_family_protein
Phvul.007G254200	Chr07	37,605,116	37,607,674	NB-ARC_domain-containing_disease_resistance_protein
Phvul.007G254300	Chr07	37,610,835	37,613,384	NB-ARC_domain-containing_disease_resistance_protein
Phvul.007G254400	Chr07	37,621,409	37,623,661	NB-ARC_domain-containing_disease_resistance_protein
Phvul.007G254501	Chr07	37,627,825	37,628,211	NB-ARC_domain-containing_disease_resistance_protein
Phvul.007G254633	Chr07	37,631,711	37,636,947	LRR_and_NB-ARC_domains-containing_disease_resistance_protein
Phvul.007G254666	Chr07	37,633,349	37,634,242	NB-ARC_domain-containing_disease_resistance_protein
Phvul.007G254700	Chr07	37,638,022	37,640,574	NB-ARC_domain-containing_disease_resistance_protein

Gene	Chrom	Start	End	Phytozome anotation
Phvul.007G254900	Chr07	37,652,491	37,655,983	NB-ARC_domain-containing_disease_resistance_protein
Phvul.007G261700	Chr07	38,373,507	38,374,058	Disease_resistance-responsive_(dirigent-like_protein)_family_protein
Phvul.008G014700	Chr08	1,206,027	1,209,066	NB-ARC_domain-containing_disease_resistance_protein
Phvul.008G018700	Chr08	1,543,619	1,545,356	Disease_resistance-responsive_(dirigent-like_protein)_family_protein
Phvul.008G020700	Chr08	1,735,768	1,747,788	LRR_and_NB-ARC_domains-containing_disease_resistance_protein
Phvul.008G020750	Chr08	1,743,459	1,745,204	NB-ARC_domain-containing_disease_resistance_protein
Phvul.008G020900	Chr08	1,755,656	1,757,267	Disease_resistance_protein_(CC-NBS-LRR_class)_family
Phvul.008G031200	Chr08	2,493,248	2,498,389	NB-ARC_domain-containing_disease_resistance_protein
Phvul.008G061300	Chr08	5,568,478	5,573,415	Disease_resistance_protein_(CC-NBS-LRR_class)_family
Phvul.008G066901	Chr08	6,111,979	6,113,177	Disease_resistance_protein_(CC-NBS-LRR_class)_family
Phvul.008G070800	Chr08	6,421,789	6,441,314	NB-ARC_domain-containing_disease_resistance_protein
Phvul.008G071200	Chr08	6,479,259	6,486,418	NB-ARC_domain-containing_disease_resistance_protein
Phvul.008G071300	Chr08	6,507,613	6,525,729	NB-ARC_domain-containing_disease_resistance_protein
Phvul.008G071500	Chr08	6,553,131	6,566,131	NB-ARC_domain-containing_disease_resistance_protein
Phvul.008G071966	Chr08	6,616,733	6,623,179	LRR_and_NB-ARC_domains-containing_disease_resistance_protein
Phvul.008G072032	Chr08	6,623,308	6,626,498	NB-ARC_domain-containing_disease_resistance_protein
Phvul.008G072100	Chr08	6,631,648	6,642,636	NB-ARC_domain-containing_disease_resistance_protein
Phvul.008G072200	Chr08	6,663,129	6,672,952	NB-ARC_domain-containing_disease_resistance_protein
Phvul.008G072300	Chr08	6,690,017	6,700,524	Disease_resistance_protein_(CC-NBS-LRR_class)_family
Phvul.008G083300	Chr08	8,122,857	8,123,583	Disease_resistance-responsive_(dirigent-like_protein)_family_protein
Phvul.008G091600	Chr08	9,210,333	9,211,508	Disease_resistance-responsive_(dirigent-like_protein)_family_protein
Phvul.008G194828	Chr08	53,808,150	53,817,557	Disease_resistance_protein_(TIR-NBS-LRR_class)_family
Phvul.008G194900	Chr08	53,834,145	53,838,071	disease_resistance_protein_(TIR-NBS-LRR_class),_putative
Phvul.008G195000	Chr08	53,846,593	53,847,090	Disease_resistance_protein_(TIR-NBS-LRR_class)_family
Phvul.008G195100	Chr08	53,872,671	53,881,362	disease_resistance_protein_(TIR-NBS-LRR_class),_putative
Phvul.008G195214	Chr08	53,901,589	53,918,582	disease_resistance_protein_(TIR-NBS-LRR_class),_putative
Phvul.008G244500	Chr08	59,300,910	59,306,506	NB-ARC_domain-containing_disease_resistance_protein
Phvul.008G244900	Chr08	59,349,070	59,353,730	NB-ARC_domain-containing_disease_resistance_protein
Phvul.008G245100	Chr08	59,376,027	59,380,574	NB-ARC_domain-containing_disease_resistance_protein
Phvul.008G245200	Chr08	59,398,563	59,406,233	NB-ARC_domain-containing_disease_resistance_protein
Phvul.008G245400	Chr08	59,422,743	59,429,327	Disease_resistance_protein_(CC-NBS-LRR_class)_family
Phvul.008G246500	Chr08	59,548,955	59,554,607	NB-ARC_domain-containing_disease_resistance_protein
Phvul.008G246800	Chr08	59,564,606	59,574,197	NB-ARC_domain-containing_disease_resistance_protein
Phvul.008G247000	Chr08	59,583,627	59,589,291	NB-ARC_domain-containing_disease_resistance_protein
Phvul.008G247200	Chr08	59,605,290	59,610,687	NB-ARC_domain-containing_disease_resistance_protein
Phvul.008G247300	Chr08	59,616,843	59,622,239	NB-ARC_domain-containing_disease_resistance_protein
Phvul.008G265900	Chr08	61,145,417	61,147,356	disease_resistance_protein_(TIR-NBS-LRR_class),_putative
Phvul.008G267600	Chr08	61,269,168	61,271,742	disease_resistance_protein_(TIR-NBS-LRR_class),_putative
Phvul.008G267700	Chr08	61,284,038	61,286,276	disease_resistance_protein_(TIR-NBS-LRR_class),_putative
Phvul.008G284500	Chr08	62,454,696	62,464,470	NB-ARC_domain-containing_disease_resistance_protein
Phvul.008G285300	Chr08	62,514,556	62,520,686	NB-ARC_domain-containing_disease_resistance_protein
Phvul.009G233700	Chr09	35,054,720	35,058,602	NB-ARC_domain-containing_disease_resistance_protein
Phvul.009G237600	Chr09	35,588,161	35,594,392	disease_resistance_protein_(TIR-NBS-LRR_class),_putative
Phvul.010G008700	Chr10	1,236,830	1,248,545	Disease_resistance_protein_(TIR-NBS-LRR_class)_family
Phvul.010G018400	Chr10	2,689,239	2,693,034	Disease_resistance_protein_(TIR-NBS-LRR_class)_family
Phvul.010G023100	Chr10	3,272,172	3,280,327	LRR_and_NB-ARC_domains-containing_disease_resistance_protein
Phvul.010G023200	Chr10	3,283,510	3,296,126	LRR_and_NB-ARC_domains-containing_disease_resistance_protein
Phvul.010G023500	Chr10	3,306,753	3,310,882	Disease_resistance_protein_(TIR-NBS-LRR_class)_family
Phvul.010G024000	Chr10	3,382,255	3,384,686	Disease_resistance_protein_(TIR-NBS-LRR_class)_family
Phvul.010G024100	Chr10	3,388,369	3,391,852	Disease_resistance_protein_(TIR-NBS-LRR_class)_family
Phvul.010G024200	Chr10	3,404,232	3,407,834	Disease_resistance_protein_(TIR-NBS-LRR_class)_family
Phvul.010G024250	Chr10	3,412,387	3,416,106	Disease_resistance_protein_(TIR-NBS-LRR_class)_family
Phvul.010G024301	Chr10	3,434,532	3,438,142	Disease_resistance_protein_(TIR-NBS-LRR_class)_family
Phvul.010G024351	Chr10	3,445,959	3,449,123	Disease_resistance_protein_(TIR-NBS-LRR_class)_family
Phvul.010G025000	Chr10	3,647,546	3,654,388	Disease_resistance_protein_(TIR-NBS-LRR_class)_family
Phvul.010G025100	Chr10	3,666,324	3,671,253	NB-ARC_domain-containing_disease_resistance_protein
Phvul.010G025166	Chr10	3,689,276	3,690,546	Disease_resistance_protein_(TIR-NBS-LRR_class)_family
Phvul.010G025232	Chr10	3,690,624	3,692,978	Disease_resistance_protein_(TIR-NBS-LRR_class)_family
Phvul.010G025300	Chr10	3,700,659	3,703,172	Disease_resistance_protein_(TIR-NBS-LRR_class)_family
Phvul.010G025400	Chr10	3,703,928	3,707,892	Disease_resistance_protein_(TIR-NBS-LRR_class)_family
Phvul.010G025500	Chr10	3,724,245	3,728,360	Disease_resistance_protein_(TIR-NBS-LRR_class)_family
Phvul.010G025700	Chr10	3,749,905	3,754,066	Disease_resistance_protein_(TIR-NBS-LRR_class)_family
Phvul.010G026000	Chr10	3,820,734	3,825,338	Disease_resistance_protein_(TIR-NBS-LRR_class)_family
Phvul.010G026100	Chr10	3,834,094	3,838,540	Disease_resistance_protein_(TIR-NBS-LRR_class)_family
Phvul.010G026400	Chr10	3,849,130	3,857,638	Disease_resistance_protein_(TIR-NBS-LRR_class)_family
Phvul.010G026601	Chr10	3,870,527	3,872,003	Disease_resistance_protein_(TIR-NBS-LRR_class)_family
Phvul.010G026700	Chr10	3,881,858	3,885,549	Disease_resistance_protein_(TIR-NBS-LRR_class)_family
Phvul.010G026900	Chr10	3,896,238	3,898,513	Disease_resistance_protein_(TIR-NBS-LRR_class)_family
Phvul.010G027100	Chr10	3,919,222	3,921,838	Disease_resistance_protein_(TIR-NBS-LRR_class)_family
Phvul.010G027166	Chr10	3,961,149	3,963,083	Disease_resistance_protein_(TIR-NBS-LRR_class)_family
Phvul.010G027300	Chr10	3,977,961	3,981,921	Disease_resistance_protein_(TIR-NBS-LRR_class)_family
Phvul.010G027450	Chr10	3,988,944	3,993,169	Disease_resistance_protein_(TIR-NBS-LRR_class)_family
Phvul.010G027500	Chr10	3,996,414	3,998,324	Disease_resistance_protein_(TIR-NBS-LRR_class)_family
Phvul.010G027700	Chr10	4,027,628	4,031,503	Disease_resistance_protein_(TIR-NBS-LRR_class)_family

Gene	Chrom	Start	End	Phytozome anotation
Phvul.010G027801	Chr10	4,032,824	4,036,930	Disease_resistance_protein_(TIR-NBS-LRR_class)_family
Phvul.010G027900	Chr10	4,039,062	4,043,307	Disease_resistance_protein_(TIR-NBS-LRR_class)_family
Phvul.010G028000	Chr10	4,048,869	4,053,085	Disease_resistance_protein_(TIR-NBS-LRR_class)_family
Phvul.010G028101	Chr10	4,061,622	4,065,402	Disease_resistance_protein_(TIR-NBS-LRR_class)_family
Phvul.010G028200	Chr10	4,078,657	4,085,388	Disease_resistance_protein_(TIR-NBS-LRR_class)_family
Phvul.010G028400	Chr10	4,109,335	4,110,841	Disease_resistance_protein_(TIR-NBS-LRR_class)_family
Phvul.010G028500	Chr10	4,114,303	4,117,007	Disease_resistance_protein_(TIR-NBS-LRR_class)_family
Phvul.010G028600	Chr10	4,119,479	4,121,559	Disease_resistance_protein_(TIR-NBS-LRR_class)_family
Phvul.010G028700	Chr10	4,142,109	4,146,008	Disease_resistance_protein_(TIR-NBS-LRR_class)_family
Phvul.010G028800	Chr10	4,152,908	4,156,501	Disease_resistance_protein_(TIR-NBS-LRR_class)_family
Phvul.010G028900	Chr10	4,206,402	4,209,649	Disease_resistance_protein_(TIR-NBS-LRR_class)_family
Phvul.010G029000	Chr10	4,210,553	4,214,276	Disease_resistance_protein_(TIR-NBS-LRR_class)_family
Phvul.010G029100	Chr10	4,241,041	4,252,247	Disease_resistance_protein_(TIR-NBS-LRR_class)_family
Phvul.010G029201	Chr10	4,248,551	4,252,234	Disease_resistance_protein_(TIR-NBS-LRR_class)_family
Phvul.010G029300	Chr10	4,255,868	4,257,716	Disease_resistance_protein_(TIR-NBS-LRR_class)_family
Phvul.010G029400	Chr10	4,290,681	4,296,621	Disease_resistance_protein_(TIR-NBS-LRR_class)_family
Phvul.010G029600	Chr10	4,327,159	4,330,947	Disease_resistance_protein_(TIR-NBS-LRR_class)_family
Phvul.010G029700	Chr10	4,336,669	4,340,928	Disease_resistance_protein_(TIR-NBS-LRR_class)_family
Phvul.010G029800	Chr10	4,341,837	4,343,673	Disease_resistance_protein_(TIR-NBS-LRR_class)_family
Phvul.010G054200	Chr10	8,380,105	8,385,877	Disease_resistance_protein_(TIR-NBS-LRR_class)_family
Phvul.010G054300	Chr10	8,409,164	8,410,227	Disease_resistance_protein_(TIR-NBS-LRR_class)_family
Phvul.010G054400	Chr10	8,413,737	8,415,563	Disease_resistance_protein_(TIR-NBS-LRR_class)_family
Phvul.010G054500	Chr10	8,426,454	8,429,905	Disease_resistance_protein_(TIR-NBS-LRR_class)_family
Phvul.010G054600	Chr10	8,437,692	8,439,754	Disease_resistance_protein_(TIR-NBS-LRR_class)_family
Phvul.010G054700	Chr10	8,449,702	8,453,580	Disease_resistance_protein_(TIR-NBS-LRR_class)_family
Phvul.010G054800	Chr10	8,478,392	8,482,655	Disease_resistance_protein_(TIR-NBS-LRR_class)_family
Phvul.010G055100	Chr10	8,512,688	8,516,389	Disease_resistance_protein_(TIR-NBS-LRR_class)_family
Phvul.010G055200	Chr10	8,600,902	8,606,805	Disease_resistance_protein_(TIR-NBS-LRR_class)_family
Phvul.010G055300	Chr10	8,665,818	8,668,931	Disease_resistance_protein_(TIR-NBS-LRR_class)_family
Phvul.010G061333	Chr10	9,885,787	9,887,412	Disease_resistance_protein_(TIR-NBS-LRR_class)_family
Phvul.010G063000	Chr10	11,340,502	11,346,267	NB-ARC_domain-containing_disease_resistance_protein
Phvul.010G063100	Chr10	11,364,027	11,368,326	LRR_and_NB-ARC_domains-containing_disease_resistance_protein
Phvul.010G063400	Chr10	11,509,942	11,510,846	Disease_resistance-responsive_(dirigent-like_protein)_family_protein
Phvul.010G063500	Chr10	11,550,385	11,550,948	Disease_resistance-responsive_(dirigent-like_protein)_family_protein
Phvul.010G063600	Chr10	11,613,891	11,614,460	Disease_resistance-responsive_(dirigent-like_protein)_family_protein
Phvul.010G063700	Chr10	11,792,600	11,808,472	LRR_and_NB-ARC_domains-containing_disease_resistance_protein
Phvul.010G063800	Chr10	11,819,853	11,820,789	Disease_resistance-responsive_(dirigent-like_protein)_family_protein
Phvul.010G063900	Chr10	11,871,120	11,871,689	Disease_resistance-responsive_(dirigent-like_protein)_family_protein
Phvul.010G064700	Chr10	12,257,527	12,260,383	NB-ARC_domain-containing_disease_resistance_protein
Phvul.010G064800	Chr10	12,320,957	12,326,026	disease_resistance_protein_(TIR-NBS-LRR_class),_putative
Phvul.010G091100	Chr10	34,475,362	34,492,105	NB-ARC_domain-containing_disease_resistance_protein
Phvul.010G091200	Chr10	34,504,191	34,515,966	NB-ARC_domain-containing_disease_resistance_protein
Phvul.010G091500	Chr10	34,613,095	34,624,939	Disease_resistance_protein_(CC-NBS-LRR_class)_family
Phvul.010G091650	Chr10	34,713,367	34,715,677	NB-ARC_domain-containing_disease_resistance_protein
Phvul.010G091701	Chr10	34,716,112	34,720,651	NB-ARC_domain-containing_disease_resistance_protein
Phvul.010G091800	Chr10	34,744,680	34,754,984	NB-ARC_domain-containing_disease_resistance_protein
Phvul.010G091900	Chr10	35,306,140	35,310,341	NB-ARC_domain-containing_disease_resistance_protein
Phvul.010G101200	Chr10	37,031,154	37,041,065	disease_resistance_protein_(TIR-NBS-LRR_class),_putative
Phvul.010G104300	Chr10	37,472,766	37,482,584	NB-ARC_domain-containing_disease_resistance_protein
Phvul.010G104500	Chr10	37,573,211	37,580,034	NB-ARC_domain-containing_disease_resistance_protein
Phvul.010G131200	Chr10	41,251,316	41,252,543	Disease_resistance_protein_(TIR-NBS-LRR_class)_family
Phvul.010G131500	Chr10	41,292,778	41,293,854	Disease_resistance_protein_(TIR-NBS-LRR_class)_family
Phvul.010G131600	Chr10	41,303,078	41,303,937	Disease_resistance_protein_(TIR-NBS-LRR_class)_family
Phvul.010G131650	Chr10	41,309,147	41,312,819	Disease_resistance_protein_(TIR-NBS-LRR_class)_family
Phvul.010G131700	Chr10	41,315,127	41,315,658	Disease_resistance_protein_(TIR-NBS-LRR_class)_family
Phvul.010G131800	Chr10	41,375,711	41,377,793	Disease_resistance_protein_(TIR-NBS-LRR_class)_family
Phvul.010G131950	Chr10	41,343,542	41,347,265	Disease_resistance_protein_(TIR-NBS-LRR_class)_family
Phvul.010G132000	Chr10	41,364,146	41,368,254	Disease_resistance_protein_(TIR-NBS-LRR_class)_family
Phvul.010G132200	Chr10	41,348,116	41,352,321	Disease_resistance_protein_(TIR-NBS-LRR_class)_family
Phvul.010G132333	Chr10	41,368,695	41,372,423	Disease_resistance_protein_(TIR-NBS-LRR_class)_family
Phvul.010G132433	Chr10	41,356,175	41,361,275	Disease_resistance_protein_(TIR-NBS-LRR_class)_family
Phvul.010G132800	Chr10	41,489,936	41,494,654	DZC_(Disease_resistance/zinc_finger/chromosome_condensation-like_region)_domain_containing_protein
Phvul.010G136700	Chr10	41,931,480	41,939,070	disease_resistance_protein_(TIR-NBS-LRR_class),_putative
Phvul.010G136800	Chr10	41,940,820	41,950,595	disease_resistance_protein_(TIR-NBS-LRR_class),_putative
Phvul.011G009400	Chr11	707,737	709,796	LRR_and_NB-ARC_domains-containing_disease_resistance_protein
Phvul.011G014200	Chr11	1,106,474	1,109,471	NB-ARC_domain-containing_disease_resistance_protein
Phvul.011G014301	Chr11	1,129,547	1,132,371	NB-ARC_domain-containing_disease_resistance_protein
Phvul.011G014400	Chr11	1,142,010	1,144,947	NB-ARC_domain-containing_disease_resistance_protein
Phvul.011G014450	Chr11	1,148,574	1,149,437	Disease_resistance_protein_(CC-NBS-LRR_class)_family
Phvul.011G014500	Chr11	1,149,480	1,151,453	Disease_resistance_protein_(CC-NBS-LRR_class)_family
Phvul.011G030000	Chr11	2,721,518	2,726,069	disease_resistance_protein_(TIR-NBS-LRR_class),_putative
Phvul.011G068000	Chr11	6,046,365	6,047,261	Disease_resistance-responsive_(dirigent-like_protein)_family_protein

Gene	Chrom	Start	End	Phytozome anotation
Phvul.011G068300	Chr11	6,082,517	6,083,565	Disease_resistance-responsive_(dirigent-like_protein)_family_protein
Phvul.011G068400	Chr11	6,084,603	6,085,340	Disease_resistance-responsive_(dirigent-like_protein)_family_protein
Phvul.011G074800	Chr11	6,969,389	6,974,063	LRR_and_NB-ARC_domains-containing_disease_resistance_protein
Phvul.011G098600	Chr11	11,239,583	11,240,223	Disease_resistance-responsive_(dirigent-like_protein)_family_protein
Phvul.011G098700	Chr11	11,126,802	11,127,332	Disease_resistance-responsive_(dirigent-like_protein)_family_protein
Phvul.011G100450	Chr11	11,131,483	11,132,037	Disease_resistance-responsive_(dirigent-like_protein)_family_protein
Phvul.011G102300	Chr11	11,316,638	11,317,168	Disease_resistance-responsive_(dirigent-like_protein)_family_protein
Phvul.011G136130	Chr11	31,531,281	31,535,473	Disease_resistance_protein_(TIR-NBS-LRR_class)_family
Phvul.011G136300	Chr11	33,675,533	33,676,214	Disease_resistance_protein_(TIR-NBS-LRR_class)_family
Phvul.011G140300	Chr11	35,823,935	35,832,817	disease_resistance_protein_(TIR-NBS-LRR_class),_putative
Phvul.011G140400	Chr11	35,920,752	35,924,497	disease_resistance_protein_(TIR-NBS-LRR_class),_putative
Phvul.011G149400	Chr11	42,263,345	42,268,487	NB-ARC_domain-containing_disease_resistance_protein
Phvul.011G151300	Chr11	42,856,022	42,859,590	NB-ARC_domain-containing_disease_resistance_protein
Phvul.011G154000	Chr11	44,100,225	44,103,014	NB-ARC_domain-containing_disease_resistance_protein
Phvul.011G154032	Chr11	44,125,008	44,127,797	NB-ARC_domain-containing_disease_resistance_protein
Phvul.011G166100	Chr11	47,003,206	47,005,542	NB-ARC_domain-containing_disease_resistance_protein
Phvul.011G172100	Chr11	47,994,232	48,004,529	NB-ARC_domain-containing_disease_resistance_protein
Phvul.011G173801	Chr11	48,350,423	48,351,775	NB-ARC_domain-containing_disease_resistance_protein
Phvul.011G181366	Chr11	49,362,934	49,365,756	disease_resistance_protein_(TIR-NBS-LRR_class),_putative
Phvul.011G181432	Chr11	49,365,758	49,366,961	Disease_resistance_protein_(TIR-NBS-LRR_class)
Phvul.011G181500	Chr11	49,372,211	49,376,151	NB-ARC_domain-containing_disease_resistance_protein
Phvul.011G181600	Chr11	49,377,236	49,381,158	NB-ARC_domain-containing_disease_resistance_protein
Phvul.011G181700	Chr11	49,382,769	49,386,443	NB-ARC_domain-containing_disease_resistance_protein
Phvul.011G191400	Chr11	50,491,751	50,497,068	NB-ARC_domain-containing_disease_resistance_protein
Phvul.011G191466	Chr11	50,499,423	50,500,535	NB-ARC_domain-containing_disease_resistance_protein
Phvul.011G191532	Chr11	50,501,193	50,502,011	LRR_and_NB-ARC_domains-containing_disease_resistance_protein
Phvul.011G191600	Chr11	50,505,430	50,508,984	NB-ARC_domain-containing_disease_resistance_protein
Phvul.011G191700	Chr11	50,531,564	50,533,010	LRR_and_NB-ARC_domains-containing_disease_resistance_protein
Phvul.011G191800	Chr11	50,551,551	50,556,295	NB-ARC_domain-containing_disease_resistance_protein
Phvul.011G192100	Chr11	50,574,091	50,577,711	NB-ARC_domain-containing_disease_resistance_protein
Phvul.011G192200	Chr11	50,578,832	50,582,397	LRR_and_NB-ARC_domains-containing_disease_resistance_protein
Phvul.011G192400	Chr11	50,585,012	50,588,803	NB-ARC_domain-containing_disease_resistance_protein
Phvul.011G192500	Chr11	50,591,778	50,595,579	NB-ARC_domain-containing_disease_resistance_protein
Phvul.011G192600	Chr11	50,611,703	50,615,617	NB-ARC_domain-containing_disease_resistance_protein
Phvul.011G192900	Chr11	50,645,753	50,649,822	LRR_and_NB-ARC_domains-containing_disease_resistance_protein
Phvul.011G193001	Chr11	50,653,977	50,657,704	LRR_and_NB-ARC_domains-containing_disease_resistance_protein
Phvul.011G193100	Chr11	50,670,842	50,678,683	NB-ARC_domain-containing_disease_resistance_protein
Phvul.011G193500	Chr11	50,689,241	50,694,419	NB-ARC_domain-containing_disease_resistance_protein
Phvul.011G193600	Chr11	50,712,009	50,725,630	NB-ARC_domain-containing_disease_resistance_protein
Phvul.011G193800	Chr11	50,732,318	50,735,308	LRR_and_NB-ARC_domains-containing_disease_resistance_protein
Phvul.011G193966	Chr11	50,748,435	50,749,217	NB-ARC_domain-containing_disease_resistance_protein
Phvul.011G194032	Chr11	50,750,228	50,751,196	LRR_and_NB-ARC_domains-containing_disease_resistance_protein
Phvul.011G194800	Chr11	50,800,418	50,803,999	LRR_and_NB-ARC_domains-containing_disease_resistance_protein
Phvul.011G194900	Chr11	50,811,977	50,817,887	NB-ARC_domain-containing_disease_resistance_protein
Phvul.011G195000	Chr11	50,825,721	50,829,750	LRR_and_NB-ARC_domains-containing_disease_resistance_protein
Phvul.011G195100	Chr11	50,837,588	50,840,275	LRR_and_NB-ARC_domains-containing_disease_resistance_protein
Phvul.011G195200	Chr11	50,842,886	50,846,671	NB-ARC_domain-containing_disease_resistance_protein
Phvul.011G195300	Chr11	50,850,267	50,854,335	LRR_and_NB-ARC_domains-containing_disease_resistance_protein
Phvul.011G195400	Chr11	50,863,583	50,867,140	NB-ARC_domain-containing_disease_resistance_protein
Phvul.011G195500	Chr11	50,871,923	50,875,536	NB-ARC_domain-containing_disease_resistance_protein
Phvul.011G195600	Chr11	50,888,605	50,893,894	NB-ARC_domain-containing_disease_resistance_protein
Phvul.011G195701	Chr11	50,923,232	50,924,834	LRR_and_NB-ARC_domains-containing_disease_resistance_protein
Phvul.011G195751	Chr11	50,924,988	50,926,982	NB-ARC_domain-containing_disease_resistance_protein
Phvul.011G195800	Chr11	50,930,294	50,934,413	disease_resistance_protein_(TIR-NBS-LRR_class),_putative
Phvul.011G195900	Chr11	50,938,132	50,938,734	NB-ARC_domain-containing_disease_resistance_protein
Phvul.011G196000	Chr11	50,975,654	50,980,955	NB-ARC_domain-containing_disease_resistance_protein
Phvul.011G196066	Chr11	50,995,417	50,997,278	NB-ARC_domain-containing_disease_resistance_protein
Phvul.011G196132	Chr11	50,997,352	50,998,440	LRR_and_NB-ARC_domains-containing_disease_resistance_protein
Phvul.011G197400	Chr11	51,169,033	51,171,836	NB-ARC_domain-containing_disease_resistance_protein
Phvul.011G197600	Chr11	51,184,891	51,188,442	NB-ARC_domain-containing_disease_resistance_protein
Phvul.011G197800	Chr11	51,208,688	51,212,987	LRR_and_NB-ARC_domains-containing_disease_resistance_protein

Gene	Chrom	Start	End	Phytozome anotation
Phvul.011G197900	Chr11	51,215,918	51,219,666	LRR_and_NB-ARC_domains-containing_disease_resistance_protein
Phvul.011G198000	Chr11	51,229,273	51,233,275	NB-ARC_domain-containing_disease_resistance_protein
Phvul.011G198100	Chr11	51,236,752	51,244,320	LRR_and_NB-ARC_domains-containing_disease_resistance_protein
Phvul.011G198300	Chr11	51,260,706	51,264,428	NB-ARC_domain-containing_disease_resistance_protein
Phvul.011G198400	Chr11	51,285,218	51,289,726	NB-ARC_domain-containing_disease_resistance_protein
Phvul.011G198800	Chr11	51,381,557	51,385,164	LRR_and_NB-ARC_domains-containing_disease_resistance_protein
Phvul.011G200300	Chr11	51,535,944	51,539,825	NB-ARC_domain-containing_disease_resistance_protein
Phvul.011G200800	Chr11	51,569,595	51,573,958	LRR_and_NB-ARC_domains-containing_disease_resistance_protein
Phvul.011G200820	Chr11	51,603,470	51,607,198	NB-ARC_domain-containing_disease_resistance_protein
Phvul.011G200840	Chr11	51,609,209	51,618,344	LRR_and_NB-ARC_domains-containing_disease_resistance_protein
Phvul.011G200860	Chr11	51,611,990	51,612,571	NB-ARC_domain-containing_disease_resistance_protein
Phvul.011G200880	Chr11	51,622,221	51,627,939	LRR_and_NB-ARC_domains-containing_disease_resistance_protein
Phvul.011G200900	Chr11	51,641,728	51,645,663	NB-ARC_domain-containing_disease_resistance_protein
Phvul.011G201000	Chr11	51,651,213	51,654,723	LRR_and_NB-ARC_domains-containing_disease_resistance_protein
Phvul.011G201101	Chr11	51,659,478	51,660,497	NB-ARC_domain-containing_disease_resistance_protein
Phvul.011G201900	Chr11	51,742,208	51,744,207	NB-ARC_domain-containing_disease_resistance_protein
Phvul.011G202100	Chr11	51,754,939	51,759,045	LRR_and_NB-ARC_domains-containing_disease_resistance_protein
Phvul.011G202300	Chr11	51,790,068	51,793,730	NB-ARC_domain-containing_disease_resistance_protein
Phvul.011G202366	Chr11	51,795,804	51,796,622	NB-ARC_domain-containing_disease_resistance_protein
Phvul.011G202432	Chr11	51,797,959	51,798,981	LRR_and_NB-ARC_domains-containing_disease_resistance_protein
Phvul.011G202601	Chr11	51,841,857	51,842,933	LRR_and_NB-ARC_domains-containing_disease_resistance_protein
Phvul.011G202750	Chr11	51,843,212	51,844,162	LRR_and_NB-ARC_domains-containing_disease_resistance_protein
Phvul.011G202800	Chr11	51,844,829	51,845,587	Disease_resistance_protein_(TIR-NBS-LRR_class)_family
Phvul.011G202900	Chr11	51,848,083	51,852,564	LRR_and_NB-ARC_domains-containing_disease_resistance_protein
Phvul.011G202966	Chr11	51,865,432	51,869,290	NB-ARC_domain-containing_disease_resistance_protein
Phvul.011G203032	Chr11	51,888,739	51,892,610	LRR_and_NB-ARC_domains-containing_disease_resistance_protein
Phvul.011G203100	Chr11	51,893,582	51,897,126	LRR_and_NB-ARC_domains-containing_disease_resistance_protein
Phvul.L001657	scaffold_264	1,481	9,844	NB-ARC_domain-containing_disease_resistance_protein

Supplementary Table S9. Number of SNPs in disease gene regions in each of the 11 common bean chromosomes in the set of 40 common bean accessions.

Chromosome	Number of SNPs	Number of Disease Genes	Tagged Disease Genes
1	124	34	21
2	935	48	45
3	236	18	17
4	680	115	80
5	270	15	14
6	210	16	13
7	287	23	20
8	1,101	39	38
9	22	2	2
10	1,744	95	88
11	2,232	96	88
**Total**	**7,841**	**501**	**426**

Supplementary table S10. Number of SNPs effect predicted for common bean disease genes according to Sequence ontology classification.

Effect type	Number		Impact
upstream transcript variant	3,486	44.46%	MODIFIER
downstream transcript variant	1,705	21.74%	MODIFIER
missense variant	1,191	15.19%	MODERATE
synonymous variant	661	8.43%	LOW
intron variant	454	5.79%	MODIFIER
3 prime UTR variant	166	2.12%	MODIFIER
5 prime UTR variant	97	1.24%	MODIFIER
splice region variant	41	0.52%	MODERATE
stop gained	24	0.31%	HIGH
exonic splice region variant	13	0.17%	LOW
splice donor variant	2	0.03%	HIGH
splice acceptor variant	1	0.01%	HIGH
**Total**	**7,841**		

MODIFIER: with impact on no-coding regions, MODERATE: non-synonymous substitution; LOW: synonymous coding/start/stop; HIGH: non-synonymous affecting splice-sites, stop and start codons.

Supplementary Table S11. List of the 438 SNPs associated with R-genes genotyped in this study, position, functional annotation and genes related.

Chromosome	Position	Functional Annotation	Genes	Annotation
Chr01	4030355	TA=upstream_transcript_variant	Phvul.001G043000	disease_resistance_family_protein_/_LRR_family_protein
Chr01	4053677	TA=intergenic_variant		
Chr01	7509798	TA=intron_variant	Phvul.001G062600	LRR_and_NB-ARC_domains-containing_disease_resistance_protein
Chr01	16463762	TA=downstream_transcript_variant	Phvul.001G093900	disease_resistance_protein_(TIR-NBS-LRR_class),_putative
Chr01	35572627	TA=intron_variant	Phvul.001G128200	disease_resistance_protein_(TIR-NBS-LRR_class),_putative
Chr01	36668271	TA=upstream_transcript_variant	Phvul.001G132301	NB-ARC_domain-containing_disease_resistance_protein
Chr01	36689635	TA=intergenic_variant		
Chr01	36753732	TA=upstream_transcript_variant	Phvul.001G132748	NB-ARC_domain-containing_disease_resistance_protein
Chr01	36754228	TA=upstream_transcript_variant	Phvul.001G132748	NB-ARC_domain-containing_disease_resistance_protein
Chr01	36789155	TA=intergenic_variant		
Chr01	36846372	TA=downstream_transcript_variant	Phvul.001G132600	LRR_and_NB-ARC_domains-containing_disease_resistance_protein
Chr01	36955714	TA=intergenic_variant		
Chr01	37042988	TA=upstream_transcript_variant	Phvul.001G133400	NB-ARC_domain-containing_disease_resistance_protein
Chr01	37073004	TA=downstream_transcript_variant	Phvul.001G133601	NB-ARC_domain-containing_disease_resistance_protein
Chr01	37160940	TA=intergenic_variant		
Chr01	37167612	TA=intergenic_variant		
Chr01	37200149	TA=intergenic_variant		
Chr01	37231512	TA=intergenic_variant		
Chr01	37251889	TA=intergenic_variant		
Chr01	38971907	TA=intergenic_variant		
Chr01	38976255	TA=synonymous_variant	Phvul.001G145100	Disease_resistance-responsive_(dirigent-like_protein)_family_protein
Chr01	38986251	TA=upstream_transcript_variant	Phvul.001G145200	Disease_resistance-responsive_(dirigent-like_protein)_family_protein
Chr01	39008916	TA=downstream_transcript_variant	Phvul.001G145300	Disease_resistance-responsive_(dirigent-like_protein)_family_protein
Chr01	39114582	TA=upstream_transcript_variant	Phvul.001G145600	Disease_resistance-responsive_(dirigent-like_protein)_family_protein
Chr01	39197523	TA=intergenic_variant		
Chr01	39203768	TA=intergenic_variant		
Chr01	39231969	TA=upstream_transcript_variant	Phvul.001G145900	Disease_resistance-responsive_(dirigent-like_protein)_family_protein
Chr01	42991112	TA=intergenic_variant		
Chr02	2318358	TA=intron_variant	Phvul.002G021700	Disease_resistance_protein_(CC-NBS-LRR_class)_family
Chr02	11218256	TA=missense_variant	Phvul.002G074700	NB-ARC_domain-containing_disease_resistance_protein
Chr02	11365821	TA=intergenic_variant		
Chr02	11406341	TA=intergenic_variant		
Chr02	19956992	TA=intergenic_variant		
Chr02	24770488	TA=downstream_transcript_variant	Phvul.002G115700	Disease_resistance-responsive_(dirigent-like_protein)_family_protein
Chr02	27168910	TA=downstream_transcript_variant	Phvul.002G129200	NB-ARC_domain-containing_disease_resistance_protein
Chr02	27235726	TA=missense_variant	Phvul.002G129500	NB-ARC_domain-containing_disease_resistance_protein
Chr02	27258387	TA=synonymous_variant	Phvul.002G129566	LRR_and_NB-ARC_domains-containing_disease_resistance_protein
Chr02	27260505	TA=synonymous_variant	Phvul.002G129632	NB-ARC_domain-containing_disease_resistance_protein
Chr02	27271296	TA=synonymous_variant	Phvul.002G129700	LRR_and_NB-ARC_domains-containing_disease_resistance_protein
Chr02	27289999	TA=intergenic_variant		
Chr02	27299268	TA=synonymous_variant	Phvul.002G129900	NB-ARC_domain-containing_disease_resistance_protein

Chr02	27309776	TA=missense_variant	Phvul.002G130000	NB-ARC_domain-containing_disease_resistance_protein
Chr02	27319225	TA=synonymous_variant	Phvul.002G130100	NB-ARC_domain-containing_disease_resistance_protein
Chr02	27367135	TA=intergenic_variant		
Chr02	27399134	TA=missense_variant	Phvul.002G130500	NB-ARC_domain-containing_disease_resistance_protein
Chr02	27399203	TA=missense_variant	Phvul.002G130500	NB-ARC_domain-containing_disease_resistance_protein
Chr02	27418401	TA=missense_variant	Phvul.002G130600	NB-ARC_domain-containing_disease_resistance_protein
Chr02	27447210	TA=synonymous_variant	Phvul.002G130666	LRR_and_NB-ARC_domains-containing_disease_resistance_protein
Chr02	27492799	TA=missense_variant	Phvul.002G131000	LRR_and_NB-ARC_domains-containing_disease_resistance_protein
Chr02	27500702	TA=missense_variant	Phvul.002G131100	LRR_and_NB-ARC_domains-containing_disease_resistance_protein
Chr02	27531136	TA=intron_variant	Phvul.002G131200	LRR_and_NB-ARC_domains-containing_disease_resistance_protein
Chr02	27549452	TA=splice_region_variant	Phvul.002G131500	DZC_(Disease_resistance/zinc_finger/chromosome_condensation-like_region)_domain_containing_protein
Chr02	27603887	TA=intron_variant	Phvul.002G132000	ENHANCED_DISEASE_RESISTANCE_2
Chr02	27738169	TA=5_prime_UTR_variant	Phvul.002G133400	NB-ARC_domain-containing_disease_resistance_protein
Chr02	27751808	TA=synonymous_variant	Phvul.002G133600	NB-ARC_domain-containing_disease_resistance_protein
Chr02	27965786	TA=intron_variant	Phvul.002G135000	
Chr02	28035358	TA=downstream_transcript_variant	Phvul.002G135650	NB-ARC_domain-containing_disease_resistance_protein
Chr02	29469343	TA=missense_variant	Phvul.002G145600	Disease_resistance_protein_(TIR-NBS-LRR_class)_family
Chr02	29480770	TA=intergenic_variant		
Chr02	31286845	TA=missense_variant	Phvul.002G158300	Disease_resistance_protein_(TIR-NBS_class)
Chr02	32115878	TA=synonymous_variant	Phvul.002G166400	Disease_resistance_protein_(CC-NBS-LRR_class)_family
Chr02	32707617	TA=splice_region_variant	Phvul.002G171400	disease_resistance_protein_(TIR-NBS-LRR_class),_putative
Chr02	45976148	TA=intron_variant	Phvul.002G291100	Disease_resistance_protein_(TIR-NBS_class)
Chr02	46387877	TA=synonymous_variant	Phvul.002G294800	Disease_resistance-responsive_(dirigent-like_protein)_family_protein
Chr02	46395293	TA=3_prime_UTR_variant	Phvul.002G294900	Disease_resistance-responsive_(dirigent-like_protein)_family_protein
Chr02	48791926	TA=intron_variant	Phvul.002G323000	disease_resistance_protein_(TIR-NBS-LRR_class),_putative
Chr02	48795964	TA=intergenic_variant		
Chr02	48807198	TA=missense_variant	Phvul.002G323200	disease_resistance_protein_(TIR-NBS-LRR_class)
Chr02	48818650	TA=intron_variant	Phvul.002G323300	disease_resistance_protein_(TIR-NBS-LRR_class)
Chr02	48832900	TA=5_prime_UTR_variant	Phvul.002G323400	disease_resistance_protein_(TIR-NBS-LRR_class)
Chr02	48860028	TA=intergenic_variant		
Chr02	48876556	TA=5_prime_UTR_variant	Phvul.002G323708	disease_resistance_protein_(TIR-NBS-LRR_class)
Chr02	48893417	TA=missense_variant	Phvul.002G323712	disease_resistance_protein_(TIR-NBS-LRR_class)
Chr02	49029984	TA=intergenic_variant		
Chr02	49038484	TA=intergenic_variant		
Chr03	314418	TA=intron_variant	Phvul.003G002926	NB-ARC_domain-containing_disease_resistance_protein
Chr03	325646	TA=missense_variant	Phvul.003G002700	Disease_resistance_protein_(TIR-NBS-LRR_class)_family
Chr03	341522	TA=intron_variant	Phvul.003G002600	NB-ARC_domain-containing_disease_resistance_protein
Chr03	372019	TA=synonymous_variant	Phvul.003G002400	NB-ARC_domain-containing_disease_resistance_protein
Chr03	398184	TA=missense_variant	Phvul.003G002300	NB-ARC_domain-containing_disease_resistance_protein
Chr03	11171565	TA=intergenic_variant		
Chr03	29122121	TA=intron_variant	Phvul.003G114200	Disease_resistance-responsive_(dirigent-like_protein)_family_protein
Chr03	32438952	TA=5_prime_UTR_variant	Phvul.003G129700	Disease_resistance-responsive_(dirigent-like_protein)_family_protein
Chr03	48397662	TA=intergenic_variant		
Chr03	48409973	TA=downstream_transcript_variant	Phvul.003G247400	Disease_resistance_protein_(CC-NBS-LRR_class)_family

Chr03	48420452	TA=intron_variant	Phvul.003G247500	Disease_resistance_protein_(CC-NBS-LRR_class)_family
Chr03	48429695	TA=intergenic_variant		
Chr03	48451119	TA=3_prime_UTR_variant	Phvul.003G247601	Disease_resistance_protein_(CC-NBS-LRR_class)_family
Chr03	48459307	TA=3_prime_UTR_variant	Phvul.003G247651	Disease_resistance_protein_(CC-NBS-LRR_class)_family
Chr03	49126119	TA=intergenic_variant		
Chr03	50683076	TA=missense_variant	Phvul.003G269200	disease_resistance_protein_(TIR-NBS-LRR_class),_putative
Chr03	51679177	TA=upstream_transcript_variant	Phvul.003G279500	DZC_(Disease_resistance/zinc_finger/chromosome_condensation-like_region)_domain_containing_protein
Chr04	346467	TA=synonymous_variant	Phvul.004G005600	NB-ARC_domain-containing_disease_resistance_protein
Chr04	570116	TA=missense_variant	Phvul.004G007750	NB-ARC_domain-containing_disease_resistance_protein
Chr04	621035	TA=synonymous_variant	Phvul.004G007900	NB-ARC_domain-containing_disease_resistance_protein
Chr04	637922	TA=intron_variant	Phvul.004G008001	NB-ARC_domain-containing_disease_resistance_protein
Chr04	667839	TA=intergenic_variant		
Chr04	678055	TA=intron_variant	Phvul.004G008250	LRR_and_NB-ARC_domains-containing_disease_resistance_protein
Chr04	699987	TA=intergenic_variant		
Chr04	764719	TA=synonymous_variant	Phvul.004G008620	NB-ARC_domain-containing_disease_resistance_protein
Chr04	792971	TA=intergenic_variant		
Chr04	825660	TA=intron_variant	Phvul.004G008921	LRR_and_NB-ARC_domains-containing_disease_resistance_protein
Chr04	864676	TA=missense_variant	Phvul.004G009221	LRR_and_NB-ARC_domains-containing_disease_resistance_protein
Chr04	910070	TA=intron_variant	Phvul.004G009521	NB-ARC_domain-containing_disease_resistance_protein
Chr04	958785	TA=intergenic_variant		
Chr04	1091962	TA=intergenic_variant		
Chr04	1145772	TA=intergenic_variant		
Chr04	1176404	TA=intron_variant	Phvul.004G009500	NB-ARC_domain-containing_disease_resistance_protein
Chr04	1224034	TA=intergenic_variant		
Chr04	1431487	TA=synonymous_variant	Phvul.004G012801	NB-ARC_domain-containing_disease_resistance_protein
Chr04	1439070	TA=intron_variant	Phvul.004G012900	NB-ARC_domain-containing_disease_resistance_protein
Chr04	1447841	TA=intergenic_variant		
Chr04	1466386	TA=3_prime_UTR_variant	Phvul.004G013100	NB-ARC_domain-containing_disease_resistance_protein
Chr04	1485617	TA=intron_variant	Phvul.004G013200	NB-ARC_domain-containing_disease_resistance_protein
Chr04	1494283	TA=intergenic_variant		
Chr04	1511871	TA=intron_variant	Phvul.004G013350	NB-ARC_domain-containing_disease_resistance_protein
Chr04	1682193	TA=missense_variant	Phvul.004G015600	NB-ARC_domain-containing_disease_resistance_protein
Chr04	1687192	TA=5_prime_UTR_variant	Phvul.004G015666	LRR_and_NB-ARC_domains-containing_disease_resistance_protein
Chr04	1689436	TA=upstream_transcript_variant	Phvul.004G015732	NB-ARC_domain-containing_disease_resistance_protein
Chr04	1725233	TA=intron_variant	Phvul.004G015800	NB-ARC_domain-containing_disease_resistance_protein
Chr04	1728862	TA=intron_variant	Phvul.004G015800	NB-ARC_domain-containing_disease_resistance_protein
Chr04	1736585	TA=3_prime_UTR_variant	Phvul.004G016000	NB-ARC_domain-containing_disease_resistance_protein
Chr04	1807595	TA=intergenic_variant		
Chr04	3435441	TA=synonymous_variant	Phvul.004G028900	Disease_resistance_protein_(TIR-NBS-LRR_class)_family
Chr04	4229701	TA=missense_variant	Phvul.004G036200	NB-ARC_domain-containing_disease_resistance_protein
Chr04	4238578	TA=missense_variant	Phvul.004G036300	NB-ARC_domain-containing_disease_resistance_protein
Chr04	4260068	TA=upstream_transcript_variant	Phvul.004G036600	NB-ARC_domain-containing_disease_resistance_protein
Chr04	4270341	TA=synonymous_variant	Phvul.004G036700	NB-ARC_domain-containing_disease_resistance_protein
Chr04	4279048	TA=downstream_transcript_variant	Phvul.004G036800	NB-ARC_domain-containing_disease_resistance_protein

Chr04	4282801	TA=missense_variant	Phvul.004G036900	NB-ARC_domain-containing_disease_resistance_protein
Chr04	5248719	TA=upstream_transcript_variant	Phvul.004G044301	NB-ARC_domain-containing_disease_resistance_protein
Chr04	5305130	TA=intergenic_variant		
Chr04	5314976	TA=synonymous_variant	Phvul.004G044900	disease_resistance_family_protein_/_LRR_family_protein
Chr04	5569524	TA=3_prime_UTR_variant	Phvul.004G046400	disease_resistance_protein_(TIR-NBS-LRR_class),_putative
Chr04	5837372	TA=intron_variant	Phvul.004G048000	NB-ARC_domain-containing_disease_resistance_protein
Chr04	7038548	TA=synonymous_variant	Phvul.004G053000	NB-ARC_domain-containing_disease_resistance_protein
Chr04	8166747	TA=synonymous_variant	Phvul.004G058700	disease_resistance_protein_(TIR-NBS-LRR_class),_putative
Chr04	9776110	TA=intron_variant	Phvul.004G066125	NB-ARC_domain-containing_disease_resistance_protein
Chr04	13103570	TA=intron_variant	Phvul.004G076100	NB-ARC_domain-containing_disease_resistance_protein
Chr04	13106503	TA=intron_variant	Phvul.004G076100	NB-ARC_domain-containing_disease_resistance_protein
Chr04	15430426	TA=synonymous_variant	Phvul.004G095900	disease_resistance_family_protein_/_LRR_family_protein
Chr04	15700906	TA=missense_variant	Phvul.004G096200	disease_resistance_family_protein_/_LRR_family_protein
Chr04	15789448	TA=missense_variant	Phvul.004G096400	disease_resistance_family_protein_/_LRR_family_protein
Chr04	20307715	TA=missense_variant	Phvul.004G084600	disease_resistance_family_protein_/_LRR_family_protein
Chr04	27046340	TA=intron_variant	Phvul.004G094094	disease_resistance_family_protein_/_LRR_family_protein
Chr04	30787913	TA=synonymous_variant	Phvul.004G099400	disease_resistance_family_protein_/_LRR_family_protein
Chr04	30972009	TA=synonymous_variant	Phvul.004G099700	disease_resistance_family_protein_/_LRR_family_protein
Chr04	31165298	TA=synonymous_variant	Phvul.004G105400	disease_resistance_family_protein_/_LRR_family_protein
Chr04	31454093	TA=intron_variant	Phvul.004G105501	disease_resistance_family_protein_/_LRR_family_protein
Chr04	31518390	TA=synonymous_variant	Phvul.004G105600	disease_resistance_family_protein_/_LRR_family_protein
Chr04	31655325	TA=missense_variant	Phvul.004G104900	disease_resistance_family_protein_/_LRR_family_protein
Chr04	32434957	TA=intergenic_variant		
Chr04	33092231	TA=intron_variant	Phvul.004G103600	disease_resistance_family_protein_/_LRR_family_protein
Chr04	33442970	TA=intergenic_variant		
Chr04	33504886	TA=intron_variant	Phvul.004G103000	disease_resistance_family_protein_/_LRR_family_protein
Chr04	33659175	TA=intergenic_variant		
Chr04	33687768	TA=synonymous_variant	Phvul.004G100000	disease_resistance_family_protein_/_LRR_family_protein
Chr04	33701977	TA=intron_variant	Phvul.004G100014	disease_resistance_family_protein_/_LRR_family_protein
Chr04	33729952	TA=missense_variant	Phvul.004G100200	disease_resistance_family_protein_/_LRR_family_protein
Chr04	33781121	TA=missense_variant	Phvul.004G100300	disease_resistance_family_protein_/_LRR_family_protein
Chr04	39837450	TA=missense_variant	Phvul.004G115300	disease_resistance_family_protein_/_LRR_family_protein
Chr04	39932656	TA=missense_variant	Phvul.004G115600	disease_resistance_family_protein_/_LRR_family_protein
Chr04	39952613	TA=intergenic_variant		
Chr04	40076809	TA=synonymous_variant	Phvul.004G116600	disease_resistance_family_protein_/_LRR_family_protein
Chr04	43296310	TA=upstream_transcript_variant	Phvul.004G134300	disease_resistance_protein_(TIR-NBS-LRR_class),_putative
Chr04	43431429	TA=intron_variant	Phvul.004G135100	disease_resistance_protein_(TIR-NBS-LRR_class),_putative
Chr04	43644780	TA=upstream_transcript_variant	Phvul.004G136800	Disease_resistance_protein_(TIR-NBS-LRR_class)_family
Chr04	43699955	TA=synonymous_variant	Phvul.004G137300	Disease_resistance_protein_(TIR-NBS-LRR_class)_family
Chr04	43949849	TA=missense_variant	Phvul.004G139700	Disease_resistance_protein_(TIR-NBS-LRR_class)_family
Chr04	43970533	TA=missense_variant	Phvul.004G139800	disease_resistance_protein_(TIR-NBS-LRR_class),_putative
Chr04	43979145	TA=downstream_transcript_variant	Phvul.004G139900	disease_resistance_protein_(TIR-NBS-LRR_class),_putative
Chr04	43992584	TA=intergenic_variant		
Chr04	44023256	TA=upstream_transcript_variant	Phvul.004G140400	disease_resistance_protein_(TIR-NBS-LRR_class),_putative
Chr04	44033866	TA=intron_variant	Phvul.004G140500	disease_resistance_protein_(TIR-NBS-LRR_class),_putative

Chr04	44051133	TA=intergenic_variant		
Chr04	44055958	TA=synonymous_variant	Phvul.004G140800	disease_resistance_protein_(TIR-NBS-LRR_class),_putative
Chr05	589020	TA=synonymous_variant	Phvul.005G007200	disease_resistance_family_protein_/_LRR_family_protein
Chr05	1228395	TA=intron_variant	Phvul.005G014701	NB-ARC_domain-containing_disease_resistance_protein
Chr05	1228435	TA=intron_variant	Phvul.005G014701	NB-ARC_domain-containing_disease_resistance_protein
Chr05	1341808	TA=intron_variant	Phvul.005G014200	NB-ARC_domain-containing_disease_resistance_protein
Chr05	1444059	TA=3_prime_UTR_variant	Phvul.005G016500	disease_resistance_protein_(TIR-NBS-LRR_class),_putative
Chr05	2531512	TA=intron_variant	Phvul.005G027200	NB-ARC_domain-containing_disease_resistance_protein
Chr05	2822866	TA=splice_region_variant	Phvul.005G030500	disease_resistance_protein_(TIR-NBS-LRR_class),_putative
Chr05	2899662	TA=3_prime_UTR_variant	Phvul.005G031200	LRR_and_NB-ARC_domains-containing_disease_resistance_protein
Chr05	2984078	TA=intergenic_variant		
Chr05	3005116	TA=missense_variant	Phvul.005G032500	Disease_resistance-responsive_(dirigent-like_protein)_family_protein
Chr05	3015122	TA=3_prime_UTR_variant	Phvul.005G032700	Disease_resistance-responsive_(dirigent-like_protein)_family_protein
Chr05	9140277	TA=missense_variant	Phvul.005G062300	Disease_resistance_protein_(CC-NBS-LRR_class)_family
Chr05	27068009	TA=synonymous_variant	Phvul.005G087100	NB-ARC_domain-containing_disease_resistance_protein
Chr05	29069943	TA=missense_variant	Phvul.005G093400	disease_resistance_protein_(TIR-NBS-LRR_class)
Chr05	35311942	TA=missense_variant	Phvul.005G117900	NB-ARC_domain-containing_disease_resistance_protein
Chr06	440940	TA=missense_variant	Phvul.006G001500	disease_resistance_family_protein_/_LRR_family_protein
Chr06	4248983	TA=intergenic_variant		
Chr06	4348097	TA=stop_gained	Phvul.006G018911	LRR_and_NB-ARC_domains-containing_disease_resistance_protein
Chr06	4352637	TA=intergenic_variant		
Chr06	13540764	TA=missense_variant	Phvul.006G041400	NB-ARC_domain-containing_disease_resistance_protein
Chr06	13989201	TA=downstream_transcript_variant	Phvul.006G038100	NB-ARC_domain-containing_disease_resistance_protein
Chr06	14925480	TA=synonymous_variant	Phvul.006G045400	NB-ARC_domain-containing_disease_resistance_protein
Chr06	15626484	TA=missense_variant	Phvul.006G051500	NB-ARC_domain-containing_disease_resistance_protein
Chr06	15688177	TA=synonymous_variant	Phvul.006G052300	NB-ARC_domain-containing_disease_resistance_protein
Chr06	15696176	TA=intergenic_variant		
Chr06	15718279	TA=synonymous_variant	Phvul.006G052500	NB-ARC_domain-containing_disease_resistance_protein
Chr06	15744627	TA=missense_variant	Phvul.006G052600	NB-ARC_domain-containing_disease_resistance_protein
Chr06	16316012	TA=intergenic_variant		
Chr06	17835319	TA=missense_variant	Phvul.006G066800	NB-ARC_domain-containing_disease_resistance_protein
Chr06	28900119	TA=intron_variant	Phvul.006G188700	disease_resistance_protein_(TIR_class),_putative
Chr07	6500000	TA=missense_variant	Phvul.007G070400	disease_resistance_family_protein_/_LRR_family_protein
Chr07	7410402	TA=missense_variant	Phvul.007G077500	disease_resistance_family_protein_/_LRR_family_protein
Chr07	8715626	TA=missense_variant	Phvul.007G086300	LRR_and_NB-ARC_domains-containing_disease_resistance_protein
Chr07	8885168	TA=synonymous_variant	Phvul.007G087200	disease_resistance_family_protein_/_LRR_family_protein
Chr07	8895210	TA=upstream_transcript_variant	Phvul.007G087300	disease_resistance_family_protein_/_LRR_family_protein
Chr07	8902822	TA=synonymous_variant	Phvul.007G087500	disease_resistance_family_protein_/_LRR_family_protein
Chr07	8909624	TA=synonymous_variant	Phvul.007G087550	disease_resistance_family_protein_/_LRR_family_protein
Chr07	8964656	TA=5_prime_UTR_variant	Phvul.007G087800	disease_resistance_family_protein_/_LRR_family_protein
Chr07	17863572	TA=intergenic_variant		
Chr07	21182795	TA=intergenic_variant		
Chr07	27217417	TA=intron_variant	Phvul.007G160901	disease_resistance_protein_(TIR-NBS-LRR_class),_putative
Chr07	36203505	TA=upstream_transcript_variant	Phvul.007G238100	Disease_resistance-responsive_(dirigent-like_protein)_family_protein
Chr07	36204085	TA=upstream_transcript_variant	Phvul.007G238100	Disease_resistance-responsive_(dirigent-like_protein)_family_protein

Chr07	36979848	TA=intergenic_variant		
Chr07	37605928	TA=missense_variant	Phvul.007G254200	NB-ARC_domain-containing_disease_resistance_protein
Chr07	37610413	TA=intergenic_variant		
Chr07	37622116	TA=missense_variant	Phvul.007G254400	NB-ARC_domain-containing_disease_resistance_protein
Chr07	37628435	TA=upstream_transcript_variant	Phvul.007G254501	NB-ARC_domain-containing_disease_resistance_protein
Chr07	37635207	TA=upstream_transcript_variant	Phvul.007G254666	NB-ARC_domain-containing_disease_resistance_protein
Chr07	37653507	TA=synonymous_variant	Phvul.007G254900	NB-ARC_domain-containing_disease_resistance_protein
Chr07	38373528	TA=synonymous_variant	Phvul.007G261700	Disease_resistance-responsive_(dirigent-like_protein)_family_protein
Chr08	1208824	TA=synonymous_variant	Phvul.008G014700	NB-ARC_domain-containing_disease_resistance_protein
Chr08	1542524	TA=intron_variant	Phvul.008G018700	Disease_resistance-responsive_(dirigent-like_protein)_family_protein
Chr08	1741939	TA=intron_variant	Phvul.008G020700	LRR_and_NB-ARC_domains-containing_disease_resistance_protein
Chr08	1756823	TA=intron_variant	Phvul.008G020900	Disease_resistance_protein_(CC-NBS-LRR_class)_family
Chr08	2491136	TA=synonymous_variant	Phvul.008G031100	
Chr08	5561645	TA=intergenic_variant		
Chr08	6112209	TA=5_prime_UTR_variant	Phvul.008G066901	Disease_resistance_protein_(CC-NBS-LRR_class)_family
Chr08	6438399	TA=intron_variant	Phvul.008G070800	NB-ARC_domain-containing_disease_resistance_protein
Chr08	6478731	TA=intergenic_variant		
Chr08	6511131	TA=intron_variant	Phvul.008G071300	NB-ARC_domain-containing_disease_resistance_protein
Chr08	6556561	TA=intron_variant	Phvul.008G071500	NB-ARC_domain-containing_disease_resistance_protein
Chr08	6616722	TA=downstream_transcript_variant	Phvul.008G071966	LRR_and_NB-ARC_domains-containing_disease_resistance_protein
Chr08	6627931	TA=intergenic_variant		
Chr08	6639761	TA=missense_variant	Phvul.008G072100	NB-ARC_domain-containing_disease_resistance_protein
Chr08	6671198	TA=synonymous_variant	Phvul.008G072200	NB-ARC_domain-containing_disease_resistance_protein
Chr08	6700302	TA=synonymous_variant	Phvul.008G072300	Disease_resistance_protein_(CC-NBS-LRR_class)_family
Chr08	8123200	TA=missense_variant	Phvul.008G083300	Disease_resistance-responsive_(dirigent-like_protein)_family_protein
Chr08	9211444	TA=5_prime_UTR_variant	Phvul.008G091600	Disease_resistance-responsive_(dirigent-like_protein)_family_protein
Chr08	53811972	TA=intron_variant	Phvul.008G194828	Disease_resistance_protein_(TIR-NBS-LRR_class)_family
Chr08	53834894	TA=missense_variant	Phvul.008G194900	disease_resistance_protein_(TIR-NBS-LRR_class),_putative
Chr08	53846544	TA=downstream_transcript_variant	Phvul.008G195000	Disease_resistance_protein_(TIR-NBS-LRR_class)_family
Chr08	53879230	TA=intron_variant	Phvul.008G195100	disease_resistance_protein_(TIR-NBS-LRR_class),_putative
Chr08	53915832	TA=intron_variant	Phvul.008G195214	disease_resistance_protein_(TIR-NBS-LRR_class),_putative
Chr08	59301108	TA=intron_variant	Phvul.008G244500	NB-ARC_domain-containing_disease_resistance_protein
Chr08	59349718	TA=missense_variant	Phvul.008G244900	NB-ARC_domain-containing_disease_resistance_protein
Chr08	59380674	TA=downstream_transcript_variant	Phvul.008G245100	NB-ARC_domain-containing_disease_resistance_protein
Chr08	59404720	TA=intron_variant	Phvul.008G245200	NB-ARC_domain-containing_disease_resistance_protein
Chr08	59429518	TA=downstream_transcript_variant	Phvul.008G245400	Disease_resistance_protein_(CC-NBS-LRR_class)_family
Chr08	59550422	TA=synonymous_variant	Phvul.008G246500	NB-ARC_domain-containing_disease_resistance_protein
Chr08	59584744	TA=synonymous_variant	Phvul.008G247000	NB-ARC_domain-containing_disease_resistance_protein
Chr08	59607262	TA=intron_variant	Phvul.008G247200	NB-ARC_domain-containing_disease_resistance_protein
Chr08	59618720	TA=intron_variant	Phvul.008G247300	NB-ARC_domain-containing_disease_resistance_protein
Chr08	61146742	TA=missense_variant	Phvul.008G265900	disease_resistance_protein_(TIR-NBS-LRR_class),_putative
Chr08	61269299	TA=missense_variant	Phvul.008G267600	disease_resistance_protein_(TIR-NBS-LRR_class),_putative
Chr08	61284193	TA=missense_variant	Phvul.008G267700	disease_resistance_protein_(TIR-NBS-LRR_class),_putative
Chr08	62458514	TA=missense_variant	Phvul.008G284500	NB-ARC_domain-containing_disease_resistance_protein
Chr08	62515853	TA=missense_variant	Phvul.008G285300	NB-ARC_domain-containing_disease_resistance_protein

Chr09	35057775	TA=missense_variant	Phvul.009G233700	NB-ARC_domain-containing_disease_resistance_protein
Chr09	35589096	TA=synonymous_variant	Phvul.009G237600	disease_resistance_protein_(TIR-NBS-LRR_class),_putative
Chr10	1244580	TA=intron_variant	Phvul.010G008700	Disease_resistance_protein_(TIR-NBS-LRR_class)_family
Chr10	2692819	TA=missense_variant	Phvul.010G018400	Disease_resistance_protein_(TIR-NBS-LRR_class)_family
Chr10	3275813	TA=missense_variant	Phvul.010G023100	LRR_and_NB-ARC_domains-containing_disease_resistance_protein
Chr10	3287221	TA=missense_variant	Phvul.010G023200	LRR_and_NB-ARC_domains-containing_disease_resistance_protein
Chr10	3384111	TA=synonymous_variant	Phvul.010G024000	Disease_resistance_protein_(TIR-NBS-LRR_class)_family
Chr10	3389609	TA=3_prime_UTR_variant	Phvul.010G024100	Disease_resistance_protein_(TIR-NBS-LRR_class)_family
Chr10	3407446	TA=missense_variant	Phvul.010G024200	Disease_resistance_protein_(TIR-NBS-LRR_class)_family
Chr10	3412960	TA=intron_variant	Phvul.010G024250	Disease_resistance_protein_(TIR-NBS-LRR_class)_family
Chr10	3434599	TA=missense_variant	Phvul.010G024301	Disease_resistance_protein_(TIR-NBS-LRR_class)_family
Chr10	3444484	TA=intergenic_variant		
Chr10	3648910	TA=intron_variant	Phvul.010G025000	Disease_resistance_protein_(TIR-NBS-LRR_class)_family
Chr10	3667008	TA=intron_variant	Phvul.010G025100	NB-ARC_domain-containing_disease_resistance_protein
Chr10	3689342	TA=missense_variant	Phvul.010G025166	Disease_resistance_protein_(TIR-NBS-LRR_class)_family
Chr10	3692391	TA=intron_variant	Phvul.010G025232	Disease_resistance_protein_(TIR-NBS-LRR_class)_family
Chr10	3702591	TA=intron_variant	Phvul.010G025300	Disease_resistance_protein_(TIR-NBS-LRR_class)_family
Chr10	3705964	TA=missense_variant	Phvul.010G025400	Disease_resistance_protein_(TIR-NBS-LRR_class)_family
Chr10	3725448	TA=missense_variant	Phvul.010G025500	Disease_resistance_protein_(TIR-NBS-LRR_class)_family
Chr10	3752511	TA=synonymous_variant	Phvul.010G025700	Disease_resistance_protein_(TIR-NBS-LRR_class)_family
Chr10	3820739	TA=5_prime_UTR_variant	Phvul.010G026000	Disease_resistance_protein_(TIR-NBS-LRR_class)_family
Chr10	3838530	TA=3_prime_UTR_variant	Phvul.010G026100	Disease_resistance_protein_(TIR-NBS-LRR_class)_family
Chr10	3851549	TA=missense_variant	Phvul.010G026400	Disease_resistance_protein_(TIR-NBS-LRR_class)_family
Chr10	3871687	TA=missense_variant	Phvul.010G026601	Disease_resistance_protein_(TIR-NBS-LRR_class)_family
Chr10	3882911	TA=synonymous_variant	Phvul.010G026700	Disease_resistance_protein_(TIR-NBS-LRR_class)_family
Chr10	3897510	TA=missense_variant	Phvul.010G026900	Disease_resistance_protein_(TIR-NBS-LRR_class)_family
Chr10	3920433	TA=missense_variant	Phvul.010G027100	Disease_resistance_protein_(TIR-NBS-LRR_class)_family
Chr10	3961750	TA=missense_variant	Phvul.010G027166	Disease_resistance_protein_(TIR-NBS-LRR_class)_family
Chr10	3978120	TA=missense_variant	Phvul.010G027300	Disease_resistance_protein_(TIR-NBS-LRR_class)_family
Chr10	3989975	TA=3_prime_UTR_variant	Phvul.010G027450	Disease_resistance_protein_(TIR-NBS-LRR_class)_family
Chr10	3997339	TA=missense_variant	Phvul.010G027500	Disease_resistance_protein_(TIR-NBS-LRR_class)_family
Chr10	4028017	TA=missense_variant	Phvul.010G027700	Disease_resistance_protein_(TIR-NBS-LRR_class)_family
Chr10	4033919	TA=intron_variant	Phvul.010G027801	Disease_resistance_protein_(TIR-NBS-LRR_class)_family
Chr10	4042425	TA=missense_variant	Phvul.010G027900	Disease_resistance_protein_(TIR-NBS-LRR_class)_family
Chr10	4049862	TA=intron_variant	Phvul.010G028000	Disease_resistance_protein_(TIR-NBS-LRR_class)_family
Chr10	4064046	TA=synonymous_variant	Phvul.010G028101	Disease_resistance_protein_(TIR-NBS-LRR_class)_family
Chr10	4081261	TA=intron_variant	Phvul.010G028200	Disease_resistance_protein_(TIR-NBS-LRR_class)_family
Chr10	4109796	TA=missense_variant	Phvul.010G028400	Disease_resistance_protein_(TIR-NBS-LRR_class)_family
Chr10	4113581	TA=upstream_transcript_variant	Phvul.010G028500	Disease_resistance_protein_(TIR-NBS-LRR_class)_family
Chr10	4120232	TA=intron_variant	Phvul.010G028600	Disease_resistance_protein_(TIR-NBS-LRR_class)_family
Chr10	4145892	TA=5_prime_UTR_variant	Phvul.010G028700	Disease_resistance_protein_(TIR-NBS-LRR_class)_family
Chr10	4154727	TA=missense_variant	Phvul.010G028800	Disease_resistance_protein_(TIR-NBS-LRR_class)_family
Chr10	4206491	TA=5_prime_UTR_variant	Phvul.010G028900	Disease_resistance_protein_(TIR-NBS-LRR_class)_family
Chr10	4213916	TA=missense_variant	Phvul.010G029000	Disease_resistance_protein_(TIR-NBS-LRR_class)_family
Chr10	4247854	TA=intron_variant	Phvul.010G029100	Disease_resistance_protein_(TIR-NBS-LRR_class)_family

Chr10	4259635	TA=upstream_transcript_variant	Phvul.010G029100	Disease_resistance_protein_(TIR-NBS-LRR_class)_family
Chr10	4291091	TA=missense_variant	Phvul.010G029400	Disease_resistance_protein_(TIR-NBS-LRR_class)_family
Chr10	4330095	TA=5_prime_UTR_variant	Phvul.010G029600	Disease_resistance_protein_(TIR-NBS-LRR_class)_family
Chr10	4338775	TA=synonymous_variant	Phvul.010G029700	Disease_resistance_protein_(TIR-NBS-LRR_class)_family
Chr10	4341996	TA=missense_variant	Phvul.010G029800	Disease_resistance_protein_(TIR-NBS-LRR_class)_family
Chr10	8382016	TA=synonymous_variant	Phvul.010G054200	Disease_resistance_protein_(TIR-NBS-LRR_class)_family
Chr10	8400329	TA=intergenic_variant		
Chr10	8416250	TA=intergenic_variant		
Chr10	8428776	TA=synonymous_variant	Phvul.010G054500	Disease_resistance_protein_(TIR-NBS-LRR_class)_family
Chr10	8438156	TA=synonymous_variant	Phvul.010G054600	Disease_resistance_protein_(TIR-NBS-LRR_class)_family
Chr10	8451078	TA=synonymous_variant	Phvul.010G054700	Disease_resistance_protein_(TIR-NBS-LRR_class)_family
Chr10	8478511	TA=synonymous_variant	Phvul.010G054800	Disease_resistance_protein_(TIR-NBS-LRR_class)_family
Chr10	8514683	TA=intron_variant	Phvul.010G055100	Disease_resistance_protein_(TIR-NBS-LRR_class)_family
Chr10	8604009	TA=synonymous_variant	Phvul.010G055200	Disease_resistance_protein_(TIR-NBS-LRR_class)_family
Chr10	8668233	TA=intron_variant	Phvul.010G055300	Disease_resistance_protein_(TIR-NBS-LRR_class)_family
Chr10	9888387	TA=3_prime_UTR_variant	Phvul.010G061333	Disease_resistance_protein_(TIR-NBS-LRR_class)_family
Chr10	11339430	TA=intergenic_variant		
Chr10	11363943	TA=upstream_transcript_variant	Phvul.010G063100	LRR_and_NB-ARC_domains-containing_disease_resistance_protein
Chr10	11509117	TA=intergenic_variant		
Chr10	11552959	TA=intergenic_variant		
Chr10	11612859	TA=intergenic_variant		
Chr10	11794688	TA=synonymous_variant	Phvul.010G063700	LRR_and_NB-ARC_domains-containing_disease_resistance_protein
Chr10	11819477	TA=intergenic_variant		
Chr10	11868290	TA=intergenic_variant		
Chr10	12258863	TA=missense_variant	Phvul.010G064700	NB-ARC_domain-containing_disease_resistance_protein
Chr10	12321249	TA=intron_variant	Phvul.010G064800	disease_resistance_protein_(TIR-NBS-LRR_class),_putative
Chr10	34490361	TA=missense_variant	Phvul.010G091100	NB-ARC_domain-containing_disease_resistance_protein
Chr10	34505837	TA=missense_variant	Phvul.010G091200	NB-ARC_domain-containing_disease_resistance_protein
Chr10	34611416	TA=upstream_transcript_variant	Phvul.010G091400	
Chr10	34712749	TA=upstream_transcript_variant	Phvul.010G091650	NB-ARC_domain-containing_disease_resistance_protein
Chr10	34716466	TA=intron_variant	Phvul.010G091701	NB-ARC_domain-containing_disease_resistance_protein
Chr10	34745217	TA=missense_variant	Phvul.010G091800	NB-ARC_domain-containing_disease_resistance_protein
Chr10	37037272	TA=intron_variant	Phvul.010G101200	disease_resistance_protein_(TIR-NBS-LRR_class),_putative
Chr10	37479388	TA=intron_variant	Phvul.010G104300	NB-ARC_domain-containing_disease_resistance_protein
Chr10	37576602	TA=intron_variant	Phvul.010G104500	NB-ARC_domain-containing_disease_resistance_protein
Chr10	41251579	TA=synonymous_variant	Phvul.010G131200	Disease_resistance_protein_(TIR-NBS-LRR_class)_family
Chr10	41295052	TA=intergenic_variant		
Chr10	41303495	TA=missense_variant	Phvul.010G131600	Disease_resistance_protein_(TIR-NBS-LRR_class)_family
Chr10	41308942	TA=upstream_transcript_variant	Phvul.010G131650	Disease_resistance_protein_(TIR-NBS-LRR_class)_family
Chr10	41315654	TA=missense_variant	Phvul.010G131700	Disease_resistance_protein_(TIR-NBS-LRR_class)_family
Chr10	41350433	TA=synonymous_variant	Phvul.010G132200	Disease_resistance_protein_(TIR-NBS-LRR_class)_family
Chr10	41356748	TA=upstream_transcript_variant	Phvul.010G132433	Disease_resistance_protein_(TIR-NBS-LRR_class)_family
Chr10	41365080	TA=intron_variant	Phvul.010G132000	Disease_resistance_protein_(TIR-NBS-LRR_class)_family
Chr10	41491474	TA=intron_variant	Phvul.010G132800	DZC_(Disease_resistance/zinc_finger/chromosome_condensation-like_region)_domain_containing_protein

Chr10	41937396	TA=3_prime_UTR_variant	Phvul.010G136700	disease_resistance_protein_(TIR-NBS-LRR_class),_putative
Chr10	41941876	TA=intron_variant	Phvul.010G136800	disease_resistance_protein_(TIR-NBS-LRR_class),_putative
Chr11	710737	TA=intron_variant	Phvul.011G009400	LRR_and_NB-ARC_domains-containing_disease_resistance_protein
Chr11	1108303	TA=synonymous_variant	Phvul.011G014200	NB-ARC_domain-containing_disease_resistance_protein
Chr11	1130883	TA=synonymous_variant	Phvul.011G014301	NB-ARC_domain-containing_disease_resistance_protein
Chr11	1142445	TA=3_prime_UTR_variant	Phvul.011G014400	NB-ARC_domain-containing_disease_resistance_protein
Chr11	1147353	TA=intergenic_variant		
Chr11	1150684	TA=missense_variant	Phvul.011G014500	Disease_resistance_protein_(CC-NBS-LRR_class)_family
Chr11	2725179	TA=intron_variant	Phvul.011G030000	disease_resistance_protein_(TIR-NBS-LRR_class),_putative
Chr11	6084477	TA=downstream_transcript_variant	Phvul.011G068400	Disease_resistance-responsive_(dirigent-like_protein)_family_protein
Chr11	6973714	TA=exonic_splice_region_variant	Phvul.011G074800	LRR_and_NB-ARC_domains-containing_disease_resistance_protein
Chr11	11123438	TA=intergenic_variant		
Chr11	11130126	TA=intergenic_variant		
Chr11	11314167	TA=intergenic_variant		
Chr11	31534133	TA=synonymous_variant	Phvul.011G136130	Disease_resistance_protein_(TIR-NBS-LRR_class)_family
Chr11	35825197	TA=synonymous_variant	Phvul.011G140300	disease_resistance_protein_(TIR-NBS-LRR_class),_putative
Chr11	35920991	TA=missense_variant	Phvul.011G140400	disease_resistance_protein_(TIR-NBS-LRR_class),_putative
Chr11	42267619	TA=synonymous_variant	Phvul.011G149400	NB-ARC_domain-containing_disease_resistance_protein
Chr11	42858508	TA=intron_variant	Phvul.011G151300	NB-ARC_domain-containing_disease_resistance_protein
Chr11	44096817	TA=intergenic_variant		
Chr11	44127897	TA=downstream_transcript_variant	Phvul.011G154032	NB-ARC_domain-containing_disease_resistance_protein
Chr11	47003358	TA=synonymous_variant	Phvul.011G166100	NB-ARC_domain-containing_disease_resistance_protein
Chr11	48003329	TA=splice_region_variant	Phvul.011G172100	NB-ARC_domain-containing_disease_resistance_protein
Chr11	48353278	TA=intergenic_variant		
Chr11	49363761	TA=intron_variant	Phvul.011G181366	disease_resistance_protein_(TIR-NBS-LRR_class),_putative
Chr11	49366945	TA=splice_region_variant	Phvul.011G181432	Disease_resistance_protein_(TIR-NBS-LRR_class)
Chr11	49373951	TA=missense_variant	Phvul.011G181500	NB-ARC_domain-containing_disease_resistance_protein
Chr11	49377278	TA=synonymous_variant	Phvul.011G181600	NB-ARC_domain-containing_disease_resistance_protein
Chr11	49385829	TA=synonymous_variant	Phvul.011G181700	NB-ARC_domain-containing_disease_resistance_protein
Chr11	50496578	TA=intron_variant	Phvul.011G191400	NB-ARC_domain-containing_disease_resistance_protein
Chr11	50506238	TA=missense_variant	Phvul.011G191600	NB-ARC_domain-containing_disease_resistance_protein
Chr11	50551748	TA=synonymous_variant	Phvul.011G191800	NB-ARC_domain-containing_disease_resistance_protein
Chr11	50574471	TA=synonymous_variant	Phvul.011G192100	NB-ARC_domain-containing_disease_resistance_protein
Chr11	50581505	TA=missense_variant	Phvul.011G192200	LRR_and_NB-ARC_domains-containing_disease_resistance_protein
Chr11	50585700	TA=missense_variant	Phvul.011G192400	NB-ARC_domain-containing_disease_resistance_protein
Chr11	50594809	TA=3_prime_UTR_variant	Phvul.011G192500	NB-ARC_domain-containing_disease_resistance_protein
Chr11	50615556	TA=3_prime_UTR_variant	Phvul.011G192600	NB-ARC_domain-containing_disease_resistance_protein
Chr11	50657193	TA=missense_variant	Phvul.011G193001	LRR_and_NB-ARC_domains-containing_disease_resistance_protein
Chr11	50678303	TA=synonymous_variant	Phvul.011G193200	
Chr11	50692541	TA=missense_variant	Phvul.011G193500	NB-ARC_domain-containing_disease_resistance_protein
Chr11	50714298	TA=missense_variant	Phvul.011G193600	NB-ARC_domain-containing_disease_resistance_protein
Chr11	50732551	TA=synonymous_variant	Phvul.011G193800	LRR_and_NB-ARC_domains-containing_disease_resistance_protein
Chr11	50748778	TA=missense_variant	Phvul.011G193966	NB-ARC_domain-containing_disease_resistance_protein
Chr11	50801014	TA=synonymous_variant	Phvul.011G194800	LRR_and_NB-ARC_domains-containing_disease_resistance_protein
Chr11	50813527	TA=missense_variant	Phvul.011G194900	NB-ARC_domain-containing_disease_resistance_protein

Chr11	50826651	TA=synonymous_variant	Phvul.011G195000	LRR_and_NB-ARC_domains-containing_disease_resistance_protein
Chr11	50839883	TA=5_prime_UTR_variant	Phvul.011G195100	LRR_and_NB-ARC_domains-containing_disease_resistance_protein
Chr11	50844063	TA=synonymous_variant	Phvul.011G195200	NB-ARC_domain-containing_disease_resistance_protein
Chr11	50852380	TA=missense_variant	Phvul.011G195300	LRR_and_NB-ARC_domains-containing_disease_resistance_protein
Chr11	50866100	TA=missense_variant	Phvul.011G195400	NB-ARC_domain-containing_disease_resistance_protein
Chr11	50872658	TA=synonymous_variant	Phvul.011G195500	NB-ARC_domain-containing_disease_resistance_protein
Chr11	50893018	TA=missense_variant	Phvul.011G195600	NB-ARC_domain-containing_disease_resistance_protein
Chr11	50924375	TA=missense_variant	Phvul.011G195701	LRR_and_NB-ARC_domains-containing_disease_resistance_protein
Chr11	50925440	TA=missense_variant	Phvul.011G195751	NB-ARC_domain-containing_disease_resistance_protein
Chr11	50931846	TA=missense_variant	Phvul.011G195800	disease_resistance_protein_(TIR-NBS-LRR_class),_putative
Chr11	50938893	TA=downstream_transcript_variant	Phvul.011G195900	NB-ARC_domain-containing_disease_resistance_protein
Chr11	50976412	TA=intron_variant	Phvul.011G196000	NB-ARC_domain-containing_disease_resistance_protein
Chr11	50998362	TA=synonymous_variant	Phvul.011G196132	LRR_and_NB-ARC_domains-containing_disease_resistance_protein
Chr11	51169070	TA=missense_variant	Phvul.011G197400	NB-ARC_domain-containing_disease_resistance_protein
Chr11	51188163	TA=synonymous_variant	Phvul.011G197600	NB-ARC_domain-containing_disease_resistance_protein
Chr11	51210258	TA=synonymous_variant	Phvul.011G197800	LRR_and_NB-ARC_domains-containing_disease_resistance_protein
Chr11	51217170	TA=synonymous_variant	Phvul.011G197900	LRR_and_NB-ARC_domains-containing_disease_resistance_protein
Chr11	51229789	TA=synonymous_variant	Phvul.011G198000	NB-ARC_domain-containing_disease_resistance_protein
Chr11	51233615	TA=intergenic_variant		
Chr11	51263061	TA=missense_variant	Phvul.011G198300	NB-ARC_domain-containing_disease_resistance_protein
Chr11	51287357	TA=missense_variant	Phvul.011G198400	NB-ARC_domain-containing_disease_resistance_protein
Chr11	51384824	TA=missense_variant	Phvul.011G198800	LRR_and_NB-ARC_domains-containing_disease_resistance_protein
Chr11	51536530	TA=synonymous_variant	Phvul.011G200300	NB-ARC_domain-containing_disease_resistance_protein
Chr11	51570429	TA=missense_variant	Phvul.011G200800	LRR_and_NB-ARC_domains-containing_disease_resistance_protein
Chr11	51606148	TA=synonymous_variant	Phvul.011G200820	NB-ARC_domain-containing_disease_resistance_protein
Chr11	51610993	TA=intron_variant	Phvul.011G200840	LRR_and_NB-ARC_domains-containing_disease_resistance_protein
Chr11	51626184	TA=missense_variant	Phvul.011G200880	LRR_and_NB-ARC_domains-containing_disease_resistance_protein
Chr11	51641155	TA=intergenic_variant		
Chr11	51654005	TA=missense_variant	Phvul.011G201000	LRR_and_NB-ARC_domains-containing_disease_resistance_protein
Chr11	51659898	TA=missense_variant	Phvul.011G201101	NB-ARC_domain-containing_disease_resistance_protein
Chr11	51742627	TA=synonymous_variant	Phvul.011G201900	NB-ARC_domain-containing_disease_resistance_protein
Chr11	51755550	TA=synonymous_variant	Phvul.011G202100	LRR_and_NB-ARC_domains-containing_disease_resistance_protein
Chr11	51788948	TA=intergenic_variant		
Chr11	51796237	TA=missense_variant	Phvul.011G202366	NB-ARC_domain-containing_disease_resistance_protein
Chr11	51797615	TA=upstream_transcript_variant	Phvul.011G202432	LRR_and_NB-ARC_domains-containing_disease_resistance_protein
Chr11	51840935	TA=upstream_transcript_variant	Phvul.011G202601	LRR_and_NB-ARC_domains-containing_disease_resistance_protein
Chr11	51845228	TA=upstream_transcript_variant	Phvul.011G202700	
Chr11	51849863	TA=synonymous_variant	Phvul.011G202900	LRR_and_NB-ARC_domains-containing_disease_resistance_protein
Chr11	51869070	TA=synonymous_variant	Phvul.011G202966	NB-ARC_domain-containing_disease_resistance_protein
Chr11	51889800	TA=intron_variant	Phvul.011G203032	LRR_and_NB-ARC_domains-containing_disease_resistance_protein

Supplementary Table S12. Information about the 48 common bean accessions selected for SNP validation in R-genes.

	**Accession Name**	**Gene pool**
1	BGF0014179	Andean
2	BGF0014183	Andean
3	CF200012	Middle American
4	BRS Cometa	Middle American
5	BRS Esplendor	Middle American
6	BRSMG Majestoso	Middle American
7	BRS Notavel	Middle American
8	BRS FS307	Middle American
9	BRS FC409	Middle American
10	BRS FS212	Middle American
11	BRS FC416	Middle American
12	BRS FS318	Middle American
13	BRSMG Zape	Middle American
14	CF800113	Middle American
15	BRS FC401 RMD	Middle American
16	BRS Estilo	Middle American
17	BRS FC402	Middle American
18	BRS FC414	Middle American
19	BRS FC310	Middle American
20	BRS FC104	Middle American
21	BRS Esteio	Middle American
22	BRS FP403	Middle American
23	BRS Campeiro	Middle American
24	BRS FC415	Middle American
25	Perola	Middle American
26	BRS FP417	Middle American
27	BRSMG Uniao	Andean
28	BRS Pontal	Middle American
29	Jalo Precoce	Andean
30	BRS Radiante	Andean
31	BRS Artico	Andean
32	BRS Embaixador	Andean
33	BRS Executivo	Andean
34	BRS FS308	Andean
35	BRSMG Realce	Andean
36	BRS FS305	Andean
37	BRS FS313	Andean
38	BRS FS311	Andean
39	BRS FS319	Andean
40	BRSMG Uai	Middle American
41	BRSMG Marte	Middle American
42	BRS Pitanga	Middle American
43	BRS Agreste	Middle American
44	Ouro	Middle American
45	BRS FC406	Middle American
46	BRS Marfim	Middle American
47	BRS Supremo	Middle American
48	BRS Horizonte	Middle American

Supplementary Table S13. Polymorphic SNPs identified at the flanking regions of the QTL regions associated with fusarium wil (FW), anthracnose (ANT) and Cowpea mild mottle virus (CPMMV) in common bean.

CHROM	POS	REF	ALT	Rosinha G2	BRS Cometa	Functional annotation	Gene	Flanking (-)500bp	Flanking +500bp
Chr04	229,702	C	T	C|C	T|T	TA=intergenic_variant		TGTTTCATTATGTAAATCTCATAATTTGAGAGGATTTGAGATATTATCTTACCCATTTTGTATATATATTCAATGTATATATTTAATAAGAAGTAATAAGAATTATTCTCTTTTATGATTGACAAATCTTCATAAATTTTTAGGAGAGACGAAAATGTTGTAAAGATAAAGTGTTATTTTGTTGCACAAACAATATTTTACTTGCCGCAGATGAATTCCATACAAAATATTCTCGAAGTTCCGTATTGATCGTACATGGTCCATTGACAAGTATATCCCAAACACACTTATCATGATGACAATATCCATGTCGGTCGTGGACATATATATCAAAATAATGATTTTCTTCCAACCATGCAAAATGACAAAAGAACAAGATAGAAGGAAAATAATACTTAGGAAAAAACTTAAAACTAAATGTTTCACCAACATTCAATTGTTACACTCCAAAATCATTGTGCTTATCTTTGCATTAAACAGCTAAATATCTATTTATTAAG	ATTAGTTATTTCAACTGTCACTCTAGTGGAAAGAAGAAGAACACCACTCTCTCACCACACTTCAATTGCACACCAAAAAATATTATTAATAATAAACAAAATATTACTGTAATTTTGGACATTAAAACCATCATGATCTATAAATCTTGTAAAATAAGGTAAACGCACCTATATATACTAACTAATGTAAATATTTATGAATAATTTATTATTGTTTAAAATACTTTTATATTTGTTTAAATCTCTTATTTATTTAGATGAGATGAGTTATTTGTTTATTTAATGGAGTATCTCATGCTCGATTAATATACTAAATATTTTTTTAAATAATTATATTACTCAATTTAATTAAGGTGACATCTTCAACAATATTAAAATTATAAGCAATATACTAATTTAGATTTTTTTTTTTTTTAACTCTGTTACGATTAACAGCATTTGAAATTAATGTTAGACATTGATTTTAGGAATTGTTTTTTTGCATTAATTTTTCCGTACGT
Chr04	243,516	A	C	A/A	C/C	TA=upstream_transcript_variant	TGN=Phvul.004G003900	AAAAAATTAATAATATTAACAATGATAGTATTGATAATATTAATAACATATCAATATTAATATTAATATTAATAATAATATTAATAATAATAGTAATAATTATCATATTATTAATTTAATGATAATATTATTAATATTATTACTAATATAAACAATATTAATAGTATTAATTATAATAATAATAATTACAATATAAATAATATTAGGAGTAGCAATAATATTTTTAAAATACTAATAATGTTAATTATAATATTAAAAATACCAATAAGAATAATAATAATAATTTTAATAATATTAATAATAATATAAAGAACAATATTAATATAAAGAATACTAATAACATTATTATTGTCAATATTTTATTATAGTTATTAGAATATTATCATAATTTTGGAGATTGAATCCACCATTGTTGAATTCTGAGTAACTTCAAAGAATGCCAATTTTTTTTTATATTAACTCAGTTGTTCAAATAAATAAAATTCTCTTATATCATGTTC	AAAATATAATTAATTCCCACTAATTAAAAAGTTTCATCAACATTTAAAATGTAAAATATTTTCTAAACTATCCTAGCTTCTTTATAACAAAGAGATTTTGTAAATTAAACTCAAATAAAATTAAGAGCATTTAAAGATAGTCTAATTAAATATGATAAACTCGATAATTTATACTTAAGATCAACATCTAAAACATTTTTATGTTATATTTAAAACTGAAAAGAATATGTGTTTGACGATTGGAGTAATGTTTTTGCTTAATCTACAAAGTTTAATACAACATTTGATCTTTGAATCCTTTCCAAGAAATTAAATAATATAATTTATGATTAATATGTCATCTAATTATCAACCTAAAATATCAAAATGATAATAGTGTTAAATACTTATTTAGGTAGATATTAATAGCAAGGTTATTATTAATAATATTCTTTATATTAATATTATTCTTTATATTATTGTTAATATTATTAAAATTATCATTAATATTATTATTGGTA
Chr04	269,173	A	C	A/A	C/C	TA=5_prime_UTR_variant	TGN=Phvul.004G004800	ATAATCATATTTCAAAATATCTCGTAAATTTTATTTTATAAAGTTAAAATTATATAAAATTTATTATATGAATAAATTTGATAAAATTGAGATAAATTTAAAATCAATTTTTTAATAAATTTATATAAAAAAAACATTTAAGAAGAAAACAAAAGTAAACTAAAAAAACACTGTTTTTAGTTTGTGACAGTGACTGAACTCCTTCCGCGGTTTTACTTTACCAGCTGAATGGCACTCATTGAATGTTGAATACGCGTTGCAGAGATTAATAAAATATAGTAAAGACAATTTCTTTTATCTTCCACCACCTTCTTTTCTTCATTTCTCAATCTGGGTGGTTAACTTTCTCTCACTTTTCAACCTCTTTTTTGCATTCTCATAAAAAAATGAAAGCTTTTGTTTTTAGATATTGTTGTTTTTTCCATTGTGAATAGTTTTCTGCTGACAAGGCTGTTGTTTTTGGATCTTTGATTTGATTTCTTTCTTTTTTTAACACTGCT	AACTTCTGGGTTTGTTGTTGAGTGCCTCCTTACATGTGGGGTTTTATTCTCTGCCTCCCAAACAAAGGCAAGTGTTGATTTTTTCCTCTTTTTTAATGATCCATGTTCCTGATTTTGGGGATAGGAGTGTGTGTTGGGAAAGTCGCCTCTGGGGTGTGCTAAAGTCTTGCCTTTTTTCCTCTTTCATAAACTCCTAGGGGTTATAGCTTTCTTGCATGTTGCTATGCAACTGATTCATAGTTCATGCCAGTTAAGGGTCTTTTTCTTTTAGTAGGTTGGTGTGCTTCCACATGTGTTACTGTGTAATGCAGAGAATGGCATGCTAGATAGATTCACATTTTTTTTGAGGAAAAACAAGGAAAATGGGATCTGGCAACGAGGTTCACTCAGTTGAGGAGTTCAGTTTGGAATCCAAGTGGCTTGTAGATCCTAAACAACTGTTTGTTGGCCCCAAAATTGGAGAGGGTGCTCATGCCAAAGTATATGAAGGAAAGTAAGTC
Chr04	290,880	T	C	C/C	T/T	TA=upstream_transcript_variant	TGN=Phvul.004G005200	AGTTATGAAGGTACAAAAAGTAATTGCCTACAATAAGTGTTTCAAAATTAAGAAATTAATTTCTGCAACACCATAACTATTCATGCAACTCTGTTGCTGCAGCTAAAAAGACTAAAACACCCTTTTGGTTAACTATATAAAAGTAATCTAATATAAAAGTAATCTTTAGCTAATTCACGTGCCGAAACATAGAACACGTGAAATGTTAATTTTGTCTTTTATTATCACATCTTTTTTTTATATTCATACCTTCTGAATTATAGAGTGAAATTGAATTTTTTAGGTTCAAAAATTCATTCTAAACTTTAAAATTCAGAATACAAAATTGTGTTTTATATTCTATAATCTGAAAATAATTTTTGAACTCAAAAAATTCTTTTTCAATTCTAGTATTTGAATTTAAAACTCTAAAAATGAAAATACAATTTTATATTTTAAATTTTAGAGCTCAAAAAGTGTATGGATTATAAAATTTGAAAATGAAAAAAAAAAAATCCTACA	TTTTACTCAAGGGTAATTTTGGAATCTTAAAATTTAAGGTGGGGTAGGAAGAAACTATGGAGGTGGAGGAAGAAATTTGCATGGATTGATTTATTTTCCAGAAACGGCCCATAATGAAACCCAAACCAATAATAGACAAAAAAAAATACAAAATAAGAAATCTGCATCGTCATGTGTCTGTGGCTTTGATGTTGAATTGCGGTAAAAGAAATCACCAATTCTTAGCTTCGGTACATTTCTTCTCTCACTATCACTTTTAGGGTTCTCAATTCCCACAATTTGCAATCTCAATGAGCCAATTGTTTCCGATTGTGATAACTGTGAATTGAATTGGATTTGTTGCCTTTTAAATTTGTTTTTGTTCAAGGATTCTGCGCCATTTTGTGATCTATGAGGATGGATTTGATGTCTGGTTACAAGGTATGATTTGATCTTGAGTACTCTTTTGAATTTGCAAATTTTGTAGCTCGAGATAACGACAGCGTGCAACCCGAGCTAAT
Chr04	301,047	G	A	A/A	G/G	TA=3_prime_UTR_variant	TGN=Phvul.004G005300	CTAATATATTTAGATGAGAATTCTGTTGTACAATTTGTATGTTCTTAGGAGTTACTATGTATGCTAAAATTATTTTCTATATTCCAGGAAAGTTATGTTAGTGACACAAAGTTCAATATTGGACCAAGCACAAAAATAACTCAACTAGGATATCATAATCTTTTATAGCAACTTCCAAACCCTAAAATTAACTGTTTCCTGTGAGATTGTAGTTCATTTTTATAATTCCATCATATTACCTCCGAACTACTAGATCTTATTAGTGTTTACATGGAGAGATGAAGTAGTACAGTACAATAATATGTTCATAATCACAGGACATAAAATTTGTAGATTTTCCAAACAAAAATGAAAATGTTCCATCTTCCTTTTTCATAAAGTTCAGTGATGGCCGAGAAAGTCAGGAAGGTTGATCGTGAACTTCAGGGCATGGTAATGGCCTACATTGTATCTAGGAGTAGATTGTCTTGACAAGGATTCAAAAACAAAAAGTTGTTTCT	GCTGTCTTGTCATTGCTCAATAAAGACATGTTCGTAACATAATCTTGTTTCTAAGCAGAAAGTTTCATTGGAACTTCACAGACACATTGCAAAAATTGGTAAGCATTTCAACCCTGGATCCGATGAATAATATTGTAAGAATTGCCATGGCACCAAGCAACTGAATAAGAAGAGGCATCTCTCAATTATTGTAAAGTGGCAAAAATAGATGTCCATATTGCTGAAGCCTTTTTGCTGTGTTATCAGCATCACATTCATGCAGTCATTAGGATAGTGTAAGTCTATGAAAGCCACTATGTGGACAAAGTAGTGCATGTGCATGCACCCAACTAGGTAACTAAGGGCCACAAATCTCCACTAACTGCATTGATCAAGTCAATGAAAGCCATAAAGAACCTTGCGAGGATCCAATTGTCCACTGCAATAGCTTGATGAAACAGTAGAGAGTTCCTGATATCCCATCCACTCTTCTGAGGGCTTCAAGAGAACAGGGGTTTCAA
Chr04	304,686	G	T	G/G	T/T	TA=upstream_transcript_variant	TGN=Phvul.004G005300	ACAAAAAGACCCTTCAGAAAAATGGATGAGATTTGCAGTTTCCAAGGAGAAAAAAGCATGAGGAATAAGAAAGAAACAAGTACCTATTGAGTCCAATGAAAAAAGGAGTTAGCTTACTTTGTTGGCTATCTTTCACTATAATATTATTAAGTGTGTGTGTCAGTTTAGAGTTCCAAGAGACAGAGGCAACAACTCTGCGTTTCTTTAAGAGTCCATGTTAGATTTTCCATGGTATATATATAAGATTATCATGGTTATGAAACCGTGAAGGAGAAAAGAGTGTGGCTGGTTAACGTTGAAGTTGGAGAATATCCAACAGAAATCAACCGTTAGCATCGGATTCTCTGTTGCTTTTGTCTTTTTCAAAGTTAACTATCTGAAATGTTGTGTTCTGGTTGGTCCACCTGGGACACGTGACCAATATTTCTGCCATGTACTGTTAAAGTATAAAAATACTATACTATACGATCATATGAACGGTGGTGCAAATAGCATAAAGT	ACCAAAATCAAATTGATCAAATTGGTGCAAATATCATAATTGTTGCTCACATGTTTCTGTTCAGTGGGAGTTTACTGTTTTGAAGATTTTCATACACAAATAAATCTTAATAAGATATACAACTAAATTCACCAATATGAATGTGAAAAGATTTTGAGGACAATATTATTATTTTTTCTAACGTTTTTTAGTAAAAAAATGAGTTTCTTATAGTAATACACTATGATTAAAGGTTTTTTTTTTTTCTAGTGTGTTTAGAATAAGTTTAAATATGTTTTAGTATTTTAAATTTGGCTATTATTTATAGTTTTTTAAATTAAAAATGGGTTTTTTAGTTTCTGGGTTTTGTGAATATTTTTTTTTGTCTCTTTACGTTACAATTGTTGTTCACGTGGTTAATGATATTCGTATTAAAATGTAACTAAAATACTCTTAAGAATTTATTTTTTTTAATTTTGTACTAAAACATCCTTTTAATCATTCCAATTTAGGCCAAAATTT
Chr04	360,869	C	A	A/A	C/C	TA=intergenic_variant		GCTGAGACTTAAGCTCCAGATTCGTCTCATAAGATTGGCAATAAATTTTTTATTCATAACTGCCAGGTCTGAAAGGAAGGTTCGTTTGTGTGTGAAACTGTTTGATTCTATTCGCTGGTGAATACTCATCCGTTTGGTCCGAAATTTGTCGCGTTTTGTCCCAAATTCAGTGGAGATTCTTACGTGGCATTTCAGAAAACAACTATTTCGGGAGAATTTTTCAGAAATTTTGTGCAAAACCAAAGGATATCCTCTACCAAAACTTGTGAAATTTCGCCCAAATATTCTTCAATTTTATTCTGGGTCCGTATTGCGCTGAAAATATTCCCACAAGTATTTTCATATGTTGTTTGCACCCAGGTAAGGAGGCACGTCCATTAACAAATCACAGAAGGAAGATACGGGAAAGAAAAGTCAGGACGCTTTGTTTTCAGAAAACTAGTTTTATTCACACTCGGTCTGGGACTTACTACGCCTTACTCCTTCGGTATTTTGGTTTG	ATTTTTACCAGAGGTTCGTCACTTTGTCTATTGAGTTTTGGCTGAGACTTGAGCTCCAGATTCGTCCTCCAAGATATGCAATAAATTTTTTATTCACCACTGCTAGAGCTGAACAGAAGCTTCGTTTCTGTGTGAAACTGTTTGATTCTATTTCTGGGTGAATACTCATTCGTTTGACCTGAAATTGGTCACGTTTTGTCCCAAATTCAACGGATATTTTTACGTGGAATTTCCAGAAAAAACTATTTTGGGAGAATTTTTCAGAAATTTTGTGCAAACCAAAGGCTATCCTCTACCAAAACTGGTGAAATTTCGCCCAACTTTCTGCAATTTTATTTTGGGTCTGTATTGCGCTGAAAAAATTCAAACAAGTAATTTCATATGTTGTTTGCACCCAGGTAAGGAGGCACACCCATAAACAAATTACAGAAGGAAGATACGGGGAAGAAAAGCCAGGACGTTTTGTTTTCGGAAAACGAGTTTTGTTCACTCTCGGTTTG
Chr04	373,425	A	G	A/A	G/G	TA=synonymous_variant	TGN=Phvul.004G005800	AATTGCCTTTCCCTTGATTTTCCAGAAGTTGAAATTGAGAAGGCTTTTAATGAAGCGGAAGAGACCATTTTTCACAGACATGTAGTGCCAAAGTGTGTTTGGAAGCTGCAGAAATGGCTTCAGATTGGACAAGAGAAGAAGATGAGGGAAGCATGTAAAACACTTGATGAGTTCATATATGCATGCATAGAGTCCAAGAGAGAAGATCTGAGCAAGTACGATGAAAATGAAAAGGAAGAAAGTTCTCATGTTGACTTGTTGACAGTTTTGCTGAGAGAAGGGAAGGGAAAAGGAGAAGCACACGATGATATATTTCTAAGAGATGCTGTGTTCAATCTTTTTGTGGCTGGGAGAGATACCATAACTTCAGCTCTCACCTGGTTCTTTTGGCTTGTTGCTACAAACCCTTTGGTGGAGGCCAAGATTCTTGAAGAGATGAAGGAAAAGTTTGGAAGAAATGAAAAGAAAGCAGAGGTTTTAAGTGTGGAAGAAGTGAAAAA	TGGTTTATCTGCATGGTGCTATATGTGAATCACTGAGGCTGTTTCCTCCCATACCCTTTGAACGCAAGGAAGCACTTAAATCAGACGTGCTTCCTAGTGGTCATGGTGTTAATCCAAGCACAGTTATATTGTTTTGCTTGTATTCAATGGGAAGATTTGAGGAAATATGGGGAGAAGATTGCATGGAGTTCAAGCCAGAGAGATGGATTTCACAGAAAGGAGGTAATGTTTATGTACCATCTTACAAATTCATTGCTTTTAATGCAGGACCAAGGACTTGTTTGGGCAAAGATGTCTCCTTCATTCAGATGAAGATGGTGGCAACTGCAATTTTGCACAAGTATCGTCTCCAAGTGGTGGAAGGTTATGTTGCTAAGCCAAACCTTTCAATCGTTCTTCTGATGAAGGATGGGTTAAAGGTTAAGATAAAGAAAAGAGAAATTTAGTTTGATGTATGTGGGAATTCAAATCTCAAACAGAAAAAAGTGCAAAAGATATAA
Chr04	382,837	T	G	T/T	G/G	TA=intergenic_variant		GGGCAAAGATGTCTCCTTCATTCAAATTAAGATGGTGGCAACTGCAATTTTGCACAAGTATCGTCTCCAAGTGGTGGAAGGTTATGTTGCTAAGCCAAACCTTTCAATCGTTCTTCTGATTAAGGATGGGTTAAAGGTTACGATAAAGAAGAGAGAAATGTAATTTGATGTATGTGGGAATTCCGAAGTCAGAAACAGAAAGAAGTGTAAAAGATATAAAATAAAGTGTGATGTATGTGAAATGAAGCTAATAATGATGGTGTATAATATGCCTTTGGTTTGATGAATGCATTTGGCTTATTGCAGTAAGTTCCCAGGTTGTCGTTTTTTAACGACAGTTAAGTGCTAAACCGTCGGTGTTTGGATGCAGAACCCATTTTTAACGACGGTTCAGAAAATATCTGTCGTAATTTTCACATAGGACCATTTTAAGCGATGGTTAAAAAAGAAACTGTCTTATTTTGCAGGGAAATGTTGAGTTTGAGAAATAATGTCAAATG	AGATATTACATGAGGAAAAGTTAAATATAATTAACTTACATTGTCGTTAGATGTCAGTTCCAAAGGTGATATGTACCAAATAATATGGTTAATGTATTATTGCATGTATATTATCGTGTCACGCAAATGACTATTATTGCTGAACTGAATATCTTCAATGAGATGATTGTGTTGATCAATCCACATTATTATCCACTTTTGATGGCACCCTGTCCAATTTTTGTTTGTTCTGTCTCTCATATCTTCTTTGTACAATTGATACAAGTTTAATGGAATATCTTGTCAAAACCTAAATTGTCATATCACTCAATCAACTTGATGAAATTCAAAGGTGACAAAAAGTATCAAATGAACTACTTTCCTATTACCAGGATAAACCTCAACAAATTATTAAGGGAGCGATGTCGTCTTTGTTGTAAGGAGTCCACAAGAATTGCAAAAATTGGTATGAGACAATAAATGTTAAGATTTGATGTAGGTCTGTTAAACCAGTTTGGGCT

Chr04	422,733	A	G	G/G	A/A	TA=downstream_transcript_variant	TGN=Phvul.004G006200	CTTTACACTATTTAGAGGGTGTAATTGAAAGTGAAAAAGTTTTAAAAAAAAAAAGTAACAATTTTGTTTTCAAATATAATAAAAAGAAAAGTGATAAGAAAATCGGTGAAATATGACTGTATAAGCAAACTGCATGAAATATAATTGTATGGACCAACAAAAAATAGAAGACAACCTTCATAATCATAAACTAAAATTCAATAATGCTTTTATAAATGGCTTCATTATACTGAATTACTTTTTATAAAAGTTAAAAAATTCTAGAACTTCCAAATCTTTTTTTCTAAAACTTTGTTCTTATCTTCTAATGTGTTCTTACATTCCAATTTTTTTTCTAAGATTGTAGAGACAAAAAAAGAGAAAAAAGAGATATATGCATTCAACTGTTCCAATGTGTTCTTATTAAGTTGCCAATTTCGTTAGAGTGAGTTGTCCAAGGAGAACACAATATATAGACATATATCGAAACTATAAAACAAGTTATACATATAAGTGTGCGAT	GGCACATGATAAAACAATTTTCTTTGGACAAAAGTTGGTAATTAATGTCAAGATGCATATAGGATAAATCATTTGACACTGAGTCTCAATATTAAAATGAAACGATAATGCAAAATCCTCAATCATAACCAAGTTTAGAAGTTGCTTCCATGTGCAAACCAAACCAAACATAACTTGTGATAAAAACTACATCACATAGAGAAGTGTCTCCATACTAAAATAGAATAATGTCTGCATAAATTTTAAGGCAAGTTCATCAGCATAATCTCATTCTCAACTCAAGAACATCCTCATCCATCCCTGCAAAATTGAATGAAAATTAACTAGTGACTTTCAATGCTTTTGAAACAAATAAATTAAACATACATACTTAAAAGGTGGTTCAATCCTCATTTAAGTAAGATATACAAAAGCATTTTTCTCTATATTAACTAGATATACAAAAGCATTTTTCTCTATATTAACGATCCGTCTATTGTCTATCCCGACACTACCAATTG
Chr04	446,876	C	A	C/C	A/A	TA=upstream_transcript_variant	TGN=Phvul.004G006400	TTTGAAAATTCTATAAAATATAAATTATAAAAGTCTTTTAAAAAACCATTACATTGTAATAAAATATTTTAAAAACTAAATAACTTTTTTTCCGGAAACAATTCTTTTGGAATCCGAATGCAGTACATTCCATTAAATCTTCTTAAAAAATTATTTCTTATTTAATTTGATTGGAATCATTATTTTTCTTATAAATTATTTCTTATGTGATCTCATTTGATTATAAGAATTTATCTGATTGATCCAAATCATATACCATAATAATAATTTTATTATGAGAATTAGTCTGATAGATCCAAATCATATGCCATAATAATAATCATTTGATTATGATAATTAATCTGGATTGATAGAAAATAACAATTATTTGATTATGAGAATTAATCTGGATGGATCAAAACTATATATATATCATAATATGACAAGAAAAATTATACTTTTAAAGTAATTATATACAATTCTTAATATGATAATTTAAAAAAAATATATAATAAAAGTTA	TTTATAATTAAATAATATAAAAAGTTATTAAAATAATAATATTGAAAATTGTAAAATAATATAGAAAAAAAATCACAATCCAGAATACTTTTTTCAATCAGAAATATTCATCTTAAAATAGAAATAAGAAAGTTGGTGCATGTAGTTAAGTCTAAGATTCAAAATTTGATTATAAAAGCCATTTTTGATGGGATTTTTTGTAATTTTTTCTTAATAAATAGTGGTATCTTTTCATTTAATTGTTGTGATTTGAAAATCACTAGATATTTTTAAATTTTTTTCGTTTTCTAGTTTTGTACTTTGGTGATGAAGTTATGATTCTTTCTTGAGGTATTGGTGGCTTAGTGGTCAAAAGGAGTTTGGTGGCATGATTGAAGGAAAGAATAAGAAAGAAGAAACAATGTTGTGAATGTGATTCACAAGAGTGATTGATTAACATAGTATATGTTCTTTCCAGAGTCCTAGTCCTACCTATAACCAAACCTATGCCATTAAAGCAG
Chr04	456,076	A	G	A/A	G/G	TA=intron_variant	TGN=Phvul.004G006600	ACAGCACTTCCATGCCCTTCACCCTTGATGATCTCTGGTGCCAAGTACTCGTGTGTTCCAACAAAGGACATTGACCTTGCATTTGTTGGCTCGGCAATCAGCTCAGGAAGAGGGGTCACCTGGTTATGCATGTCATTCTTTGGTTTGGACTTTTTGTCTTTCTTGGATTTTCCGGACAAAAAGCGCGGCGTGAAGCACGACGGTTTAATGCAGTCCGGCTGCATCACACATGTTGGCTCAATGCAGGAAGGCTGAACGCAGTATCCTGAGCTTTTGGTCTGCAAGTTGTTTGATGACTTCACTAGAGTTGGACTGACACTGCACCTTAGAGAGAGATCAAAGTCTGAAAGCATTATGTGACCATCTTCTCTCACCAACACATTCTCAGGTTTGAGGTCTCTGTAGATGATCCCAAGCATGTGCAAGTACTCCAAAGAAAGGAGAACTTCTGCCACATAAAACCTATAAATCCAGAAAGTCTGTTATACAAGTGACAGTAC	TTACAAATTATTCAAACAAAAAAATTAAGATACATCTTTGTCAAACTTCAAGCCTCCTAGCCAGACAACTAGAAGATAATATACCAGCAATCTTAATCTATCTGTCATAAGTAGGGTTGGACTTGGTAACAAGGAGTGGTTTCCACCAAACAAGATATATATATAAAAAGGCCAAATTGATACTAGTTAGTTACTTATTTGCTGAAGAAAAGTAGTAAAAACTGATATTTTGAACAATATATGCCATAGGTCAAACACTTGTTAAAAAGTATTGAGGGTGGAAGAGAAGCTATTTCACCAGGCTCGGATTAAAAACAAGCATGGAAATGGCTCTTTGTAAGATCACTGAGCATAGCATTGATAATAGAAAGAATATAATTTCTATCTGCTGCTAATATAAACTTTTTCAAACCAGTCTAATCATGTATTGCCACTTTATTACAAGAGCTTCAAATGATCCTCTTTTAAGTTTTACTAATGTAGTAACACGTGATTTGAAA
Chr04	458,239	A	G	A|A	G|G	TA=upstream_transcript_variant	TGN=Phvul.004G006600	GCCTCTGGCTGAGAGTAGTTCTTTCCATGATTATCAACTACACGTTTCTTCTCAGATTCTAACACACCATTTGGCTCACAACCCTTGGAATCATCAAGTGGTGGCTGCTTGCTGGACAATTTTGTTGGCAACCCTTCTTGCTTCAAGTTTTTGCCCTCTTGATGGGAATGCTTTTGATTAATTACATGGCTGTTGTGATGGTGGATGGCCTTAGTTTGATGACCACCATCATAGTTTCTAGATGCTCTCAAAGGAGGCCGCGAAGGTAGGGGAGTCCCAGAAGGAAGAGGGTCATGATGATGTACCCCAGAAACTGAATTCTGAACCTCTGACAAGGAATCAACTCCATTAACAGAAGACTCCATGAAGGGTATCTGAACAGGCTTAAAAATCCAACTCAGCACACTGAATTACCAACATGTACTCATTCAAAGAGCTCCTAATAACAGAATCTCCTACAAAAATGTCTATAAAATGGACATTTAAGCAAAGAATCAGCA	TAAATAAGATAAAAGGCATCAATCAACATACATGACACATGTAATGAAATTAAAAATAAAATTAGAGAATCTGCCAGAGACCCATTTCAGACGCAATGAATCAACAGAAAACCGTCTAAATGAGTTAAATGAGCAGAATCAATTCAGAAAGCCCCAAACAAAAAAACTTCAAAATTCAGACACCTTACTAGTAACCCAAAAGAAAGAATCACAAACTGCAAACTTCTAAAGGACTCCTCATTAAGCCTCAAGAAAATTCAGTTAGTTAAATGTTCTGTTTTACGAGTTTCCCAAAAACCCCATGTTCCTTTTGTTTACACATGGCAACAAACAAGACCAAAACATGATGTGGGAACCAACCTCATCAAAGCCACCAATAATGCATTAGACTAAAACACCCCAGGTTGAAAAGTTTGATTCTTTAGAAGCCCCCTTGAGCTTTCTTTGATTCTACTGCAGTACAGTGATGAAAAACTCCCCCATAAAGAAAAAAAAAAACAA
Chr04	461,773	T	G	G|G	T|T	TA=intergenic_variant		CACTGCACACTGCTGCTGCACACTGTGGAGTGTAAAGCTACAACCTGTGTTTCATCACACGTCCATCACAGAAGTCTCAGCTCAAAAATCACACCATCAACTGTGAATAAATAGACATAACAAGTTACCATGGACTACTAGGTTGCATGATGTTGTTGATGTGATTTTTCAGGTTTAACTTCAAATTAAAAATTACCCTTTTTCATGCTTTTTGACCATATGGTTAAGTTAAAAGTTACCCTTTTTCATGCTTTTTGAATTATCGTTAAAAGATTTCTCATCACTAAAAACTTTTGTAACCATATTCTTAAAGATTTCTCATCGATTATATGTTAAAATATATAAATGACTGCAAATGTTATTTATAAACTGATTTTACAACATTAGATTTAAAATTCATTTCTTAATATAGACTCTAAATTCAAACGAGAAAAGTGATTAAATTTCTCTTACCCTTTAAAATTTTGGAGAATTTTCATTGGCCCTTTTGAAGTATGATA	CTGATAATAGAGTTTGAGATATACATGACTTTCTTAAGAATATCTTGGGCTGTTCACATTTATGGTAAATATCAGAGTTACATGTTAAGCTAAAACATTTTGTGATCATTGATGCAAAACTTGAAATACTGATTAAGTTAGAAAAGCTGCAAATGGCTTATGCATTCAATGATTATATCACACACAAGTTGAACAACTTGAAGCAAGAATGTTGCATAGAGTGATGGAAGAACACATCATAATTTCATCAGATGAACAATTGGGCTGAAGACAATCAAATCATGGCTGGTTAACCAAACACCTTCCTACAACTCCTCATCTCACATATGGCCCCATAGGCCATCCCTCCATGATTTTGCCAATGGAAATGAGAAACAATGGATCCAAAGGAAGCAATCACAACCATACTCATGAACTTATAAAACTAATATAAGTGTGCTAGAATTTGATCACTCATCAACTTTTGCTGTGTGTAGTTTGGACTCAAACACGTGCATTGT
Chr04	466,080	A	G	A/A	G/G	TA=intron_variant	TGN=Phvul.004G006700	ATGCACTTTCTTCTTTCTAGAGTATTCCAAGGTCTCAGCAAATAATTGATTTGGCCAACCAGTTGACCCATATTAATTTCCTATGTGGATTCTTATTAGAAAATTTAATTTAATACTCGAAGCAATAACTATTACTAAAACACATTATGACGTTATGGAACTATGGATCTGATAATTTGTGTTCAAAACATTGCAACTGGTGCATTGAACTCAATAGAAATATGAGAAATAATTGCTACTTGATTATTGACATAAATTAACAGTTAAAGTGACTGATAGTCCATGAATTATATTCTTTGAACAAGATAATCAGAGTCATTCATGAGCCTCCATCTTAAAGCTTAAAACCTTTAGAAGATATATTTTTGCAAAAGAAAATTCTAAAATATACATTTCATGGTGCCAACAAAATCAAGTTGCCAATATTTAACTGTTTTCAAACAAATTCAAAATAAAATTTCAGCCAAGAAATTTAAAAATTTATACAATTAACCCTCCGG	CTGGATATCAGTTACACAAGTCAAGAGTAGAACAATCTCATGCATGTACTCGTTTTTAATAAGAGATTCCTCACCTGAGCTCCAGCAAATTCAATTTTGTTCCCCATGGATAGAAGATCATCAACTGCTTCTTCAGAGTCAAATGTGATAAACCCAAAGCCACGAGATCTGTTGGTGGAGTGGTCCCGCATTATTTGGTGATCTTTCACTTCACCATAGCGAGTAAAGAAGTCTCTAAATTCATCTGCAGAAGTGCATCTTTGATTAAAAACGATACCAACCAAAGGAATTGTCGTTCACTGAAGCTATTCTGTGATTCAACAGTTACTTTAGAAATCAAAAACTGTTTTAGTGTCTAACAGACTGTTAAAAGTTAACTGTAACAGGTATTTAAAGGGACAAAAGGGATTCATAATGAAAATAGTCAATTTACTTACACAAAGTCACACAAATAAAACACATCATTCACATCAGGACAGATGCAAATTACATGTGGCCAA
Chr04	488,793	T	C	T/T	C/C	TA=intron_variant	TGN=Phvul.004G006800	CTCTTCATGAAAGCACTAACATTTTAACATTATTCAATAAATGAAAAAGGACAAATCAGTTCACTAGGCTTCCACCAAGTGGTGTGATGGAAAAGTAGATTTATGCATCCTTACCCTCACAAGAAAAGAGGGTGTCCATAGAATTTGAACCCATGAAATATTTGGTCACAAAAAAAGATAGTGGTTTATTGTATTCATGTGTTTTGTATGATGAAGGTAACTACTAGTATGAGAATTCTAGAAGGTGAGATTAAGAACTTTTTCATATGGATAGGATTATACCAGGACACAACCTTAAGTCCTTGTTTAATCTAGTCTTGGATGTACTTATTCGAGACATACAAAAGATTATCCTTGTTGAAAAACCACCTTAATTGCAGCCACCTCTAGCCTAGTGTAGTGCAGCCTCTCTCTACCCATATGCTTCAATCCACCATATTACAAAAAACAAAGGAAAACATAGAACAACTTATAAGGACCACAAATAATAGCCAAGCTCA	GGTCTTGATTCTTGAATGCAAGACAAGAAAACCAACCACAGGCATCTGAAATCAGCCTCTGCCTCCCAAAACCACCATTCCTCAACTCAAAAGTTAGCAACCGTAAGCACACATGAACAGTAATCACACATCAACATTCAGAACCATGGTTGTCAAACTTGCAAATCAACTTGTTGACTCGTCCGAGTTCACGTTTCCACCCACCCCAACCGATCATACACAAGCTTTAGAGTTGAGGTAGCAAAACAAAACAGAGGTAGGCCCGTTGTCATGTAATTAAGTCATTGTGATCATGTCCTTTGTCAGCTAGACAACATGTACCATGTTACTATATGACAAAGGAATTAGCATCAAAGGTTACAATACAATTTAAAGAGTGAAGTAATTGGCTTAGATAGATTAAATTATATGGACAGTGTGCAATGTACAAGACCAAATTAAGATTTAAAAGAGGAGATAGAGAAACTTGAACAATTGCTTGAGATGCAACTTTGAATCCA

CHROM	POS	REF	ALT	BRS Supremo	BRS Notável	Functional annotation	Gene	Flanking (-)500bp	Flanking +500bp
Chr07	28,263,327	T	C	C|C	T|T	TA=intergenic_variant		ACGTGCCTTGAAAAAAATTAACATTATAATAAAAAAAATAAAAAAATCTGCATTTGTTTTCAATGTAATCAACCATTTAATTATCAAAATTCGAGAGTGTATGTGAACTTGTGAGATCATGAACTCGTGAGAATTCTATAGTCCACTCGATTGGTAAACTCGTATATAAAAAATATTTTATTAATATGATTAATTAAAGTATAATTGCAAAAATATTCTTAAAATAAACAAGTTTATATAAATATTATAAAACATGAGTTCTTAGAGTTGAATTTGATTCACACTTACTACATGAATTTAAAAAATAAAATATTATGAAATTTATGCTTAATTGAATCTCTCACTATCTTCAATTAAAGAAAAATGTATGTAGCATAAGCAAAGAACAAAGTCACTTTTAAAAATCATTTAATTGATTATTTGTTTGAAGTATGTGTGTATTTTTTTTTTTGGTGTTTTCTGAAATGAGAGAGAACAAAAACATAAAAAGAGGTAACCAC	AATGGTGGTGGCGGTGCAAATAATCAAAATTGAGATCGGAGACCACAAATGCGACGAAAAATAGATACGAATGCAACCACAAACAAAAGTGACTACGAATAGATACTAATAACCACAAATATAGAGAGAGGCAACCACGAACAAGGACCGACGACCATGAACATGACCTCAATACCCTAATGCAAGCTCAAAATGAGTGTGTTTGAACCCTTATGTAAGCAATAAAATCATTTTAGATTTATAAAAAATACCTATGAACTCATCAACTCACTTACAAACTCGTGAGTTCAACCAAGTTCACCGAATTTGCTTGCAATTCTACCAAATTTGCTCCAAAACCGAATTTACTTCCAAGTCGAAAAGAGTGAGTGAAAACGTAAACTCAAATGATTTAAGACTTAACTCATGAATTTGATAACTATATATAAACAATTGTTTTAAGATTTGTTTTTTCAAGTGCAAAGAGTCCACATAAGAATAAATGGTTGATTTTAAGTGTT
Chr07	28,276,375	G	A	A|A	G|G	TA=downstream_transcript_variant	TGN=Phvul.007G167200	GAAAGCTCTACCAATTTCGATAGTGACTTTTATCCTAACCTTCTCCAGAAGAAGGGTCTGTTCCAATCAGATGCTGCCCTTCTAACACTGGATCAGTCAGCAGATATAGCTGGAGAGTTGGCGGATAAAGACAAGTTCTTCACAGAGTTCGCACAGTCAATGAAGAGAATGGGAAACATTGAGGTCCTCACTGACTCTGCTGGAGAAATTCGGAACAAGTGTTCCGTTGTCAACTCATAAACAAGTTCACTACTAATTAAGCTGTTTTGTAGTCTTAATTTCCTTACATTTATATAATCTATCAGTTGTTACTTAGATTTATTGGTAGTAATATATAATATTTTTAATACATTTTCATACTTATTGGTAGTATTAGATTACTCTCGGTATATGATTGTTTTTTTTTTAATTTTCACCATAATTTTGTATATTCCTACAATGTCAAATAGTTCGTATTTATTTTTTTCGACTGAACGATAACAATATTTTAATCAGAAGTG	TTTCTTGTAAAAATTAATTAATTATATTTTATTTTAAAATATTGTTAATCACAAAGGCACAATTATAATATTACAATTTCATATAATTTTTTTTTATCATTTACACTTTTCAAATCACTCATAAACTTCAATACTTTTTTATTCATTTTTCTTCACTTTCACTTAATTCAAACAACTTCATTTTCCATTATCTTTCTCTTCAAATTCATCTTTTTCCTCTCCCTCGAATCCACTCCTTAAAACAAAGGGTGATGTTGATGTAAGATGAAAAATAAACAGAGGAAAGTGCCTGTCTAAATTGTAGCCTCTTTACCTCAAGTATCTCAACAACATGAGATATTTGGAAATTCAGCTGTTTCAGGGGAGAGGGGGAAAAGGAATGCAAATGAAAGCATGAAAAAAATAGTTAGTTAAACAAAGAAGAGGTACCATAAAACGTCCATTAAAATAATATATCCTGAAGGGCCTTTCAAGATTCACTTTTTGCTCTGCTGAATGGA

Chr07	28,293,404	A	C	C/C	A/A	TA=intron_variant	TGN=Phvul.007G167400	AACATAAAATTAAATAGTAGCCTGCCTTAAGAGCAAGTAGTAAGGTAATGGTTTCCAAGCTGTGTTGTCCTCGATCAACGTAATCTCCCAAAAACAGGTAGTCAATATAGCTGAAACATTCCACTGATTAGCTTATTCCAAAATATTCGTACAAAAAGGATAGCATGGGTAAAATGCCTTTGAAAAAATCTCACTTGGGTATTGACTAGAAGAAATGTATTATTAACACAGAACAAAACCTATTTTTTAGGAACATATAACACGTATTAACTCCCAACCCAACCATCAGTTGGCACATTTTACTTCAAATCACAACAAATTGTAAGTAAATGACATGGTTTTTGGACTGAACAGATTTTAAACAGTATAGAGTTAATTAAATACAACAATCCATTGAGTAGAAGAGAAATCCACAAGCAAAGACAGACAGCCCAAAGTTCAAGGCTCCCACTTAGTGAGTGGGGCCAGGGCAAAATAAATGTCAGCAATCTTATCCCAA	AGCAGAGAAATAGTTACCAAGATTTGAAACTGTGACTTCCACATCACAAAAAGCAACCTTACCAACACACAAAGGTATGTTTTTAAGAGTTAATTTGTCTTAAACCGATTTCAAAAGAAGGAAGGAACCACCGTTACACGTGCAGAATAATATCCCTTAAGACCTCAACTGTAGCTAACATATAAACTTCAAAAAGTTTTTACACACACTAACAAATACTCTTAAAAAAGAATGTGGTGATGTGTTTGCTAAGGACAACCCATTATTTTAAAACTTTAAGAATATGGATGGTTGACCATAATAAGCTAGTTCATCATCAAAATGATGGGACTAAGTTCCAACGACTGCAAAGTAGTAAGATCAAGGATTAGATGATAGATATCACAGCAAGTGGGCCAGAGAAGAGTTGGTTTGTACTCTATCTATACTTTGCTATTTTGATACATGACCAATGTCAATCTGCCAAGTATATTAGTAAAAACTTACGTGATGTCTCCTGC
Chr07	28,298,620	A	G	G/G	A/A	TA=intron_variant	TGN=Phvul.007G167400	AAGATGAATTGACATCATTTTCGGAGTAGAGAACTCTTACACTAATTCTAAGGCAGTGATCTCGTTTTGATCCAACAACTACATCAAAAACTTTTAGAGGAATGTAGAATTAAATAGTTAATAGAAAATTTCTCTTTAGATTCTTTTAAAATTTCAGTCTCTATTTATTGTATGAGGACAAAATGATGATAAATAGAGAAAAGTAAGTTAAACATTCAACTTTAGAAGAAGAACAACAATATGAGAAAAGAAGTGTGAGCAATAGAGTAATATTTCCAAAGACAGTATACTTTCCCAAATCTAATTTCTTGTCAACCACTACTATCCATCTAGTTTCTCCTACTTTATAAACCACAATTAGCTTATGACCATGTTGGACTATGCGAGGTAAATGGCTTAAAACTTTTATTTTTGGTATTGTGTACGAAAGATAGCTTTTAAGTGGTTATAAATATAAAACTTAGTTATCAATCCTGGGTGGTGACAGAATAGTTGACGCC	AACCCCCATAGATGGATGGCCACTGCTAATTTATAAATTAAGTAACTTATATTAGATAAGCAAGAATGTTAATAATAAAAAAGTACTCATAATTGAAGGTATATTCATAACTAAAAATTAAAAGCCATAATTTTAAGGTAAAAACAAAGAAGTTAATAACAATTGTCTAGTAATAAAAAAATAATAACGAGAAAGGCCAGCATGAGGTTGCATATCTGAACAAAAGAATGAAATAAGGAATATAAACCAAAACATAAAATCTTTCATGTGAACATAACAGACCCCAAAGAATGTGAACAGAGTAGAGACCAGAGAATGGTATGCAAAAATTCTTTATAACAACGACAACAGAAGAGCATCAAGCATTTGAACAAAGATGTTAGAGAACGAGTTTGGAGAGCTTAGTAATAAAATCTCTTGTAATAATGAGAAGCTTTGTTTTAAAATACGTTGGAAGATTCTAAAATTTGTAGAAGCTTCTAGAATGTTCTAGAACAAAAA
Chr07	28,299,174	C	T	T/T	C/C	TA=intron_variant	TGN=Phvul.007G167400	TAGATAAGCAAGAATGTTAATAATAAAAAAGTACTCATAATTGAAGGTATATTCATAACTAAAAATTAAAAGCCATAATTTTAAGGTAAAAACAAAGAAGTTAATAACAATTGTCTAGTAATAAAAAATAATAACGAGAAAGGCCAGCATGAGGTTGCATATCTGAACAAAAGAATGAAATAAGGAATATAAACCAAAACATAAAATCTTTCATGTGAACATAACAGACCCCAAAGAATGTGAACAGAGTAGAGACCAGAGAATGGTATGCAAAAATTCTTTATAACAACGACAACAGAAGAGCATCAAGCATTTGAACAAAGATGTTAGAGAACGAGTTTGGAGAGCTTAGTAATAAAATCTCTTGTAATAATGAGAAGCTTTGTTTTAAAATACGTTGGAAGATTCTAAAATTTGTAGAAGCTTCTAGAATGTTCTAGAACAAAAAGTTTAAAAGTCATAAACTAATTCCAACTATTGGTAGTTAGAGTTGGTTCATG	CCTAACTACCTCATTATCACACGAGACAAGGAAGTTGCAACTTAGATTCCTTGCATGTTGGTTTTGTTTGGCCAACCCATGATTATGGTCATCTAATTGTTTGTTTTTGTTACCCTTTTTACTTCTTGAACACACCTTATCATCCTATCCTACAAACAGAAGGCTCTTCTCATGTATTTGAGATAAATGAGAATTAGAGTTTTTGTGAGATTTTCAAGTGTCTTGAATTTGGTACTCTTAGCTTAGCTTTGTATATTAAGGAGTTTCTTCAAGTGGCGAATCAATAATGTACTTATCTTTGAAGTCTTTCAAGGTGGTGTGTTCTTTTTCCATCTTCTACCTTGTCCATTTGATCTTCTTTTTACATTTGTTTCTTTGTCTTTCCCAAGCTTGGACAAGACACTCCATGGCATTTCTTGGTTGAGTTCTTGGTTCAAATCCTTCTTAGATTTTATAGATCATACAAGTCCTAGGGAGGACTACATCAGTTCCATGTGTGG
Chr07	28,299,236	G	A	A/A	G/G	TA=intron_variant	TGN=Phvul.007G167400	AAATTAAAAGCCATAATTTTAAGGTAAAAACAAAGAAGTTAATAACAATTGTCTAGTAATAAAAAATAATAACGAGAAAGGCCAGCATGAGGTTGCATATCTGAACAAAAGAATGAAATAAGGAATATAAACCAAAACATAAAATCTTTCATGTGAACATAACAGACCCCAAAGAATGTGAACAGAGTAGAGACCAGAGAATGGTATGCAAAAATTCTTTATAACAACGACAACAGAAGAGCATCAAGCATTTGAACAAAGATGTTAGAGAACGAGTTTGGAGAGCTTAGTAATAAAATCTCTTGTAATAATGAGAAGCTTTGTTTTAAAATACGTTGGAAGATTCTAAAATTTGTAGAAGCTTCTAGAATGTTCTAGAACAAAAAGTTTAAAAGTCATAAACTAATTCCAACTATTGGTAGTTAGAGTTGGTTCATGCACCTAACTACCTCATTATCACACGAGACAAGGAAGTTGCAACTTAGATTCCTTGCATGTTG	TTTGTTTGGCCAACCCATGATTATGGTCATCTAATTGTTTGTTTTTGTTACCCTTTTTACTTCTTGAACACACCTTATCATCCTATCCTACAAACAGAAGGCTCTTCTCATGTATTTGAGATAAATGAGAATTAGAGTTTTTGTGAGATTTTCAAGTGTCTTGAATTTGGTACTCTTAGCTTAGCTTTGTATATTAAGGAGTTTCTTCAAGTGGCGAATCAATAATGTACTTATCTTTGAAGTCTTTCAAGGTGGTGTGTTCTTTTTCCATCTTCTACCTTGTCCATTTGATCTTCTTTTTACATTTGTTTCTTTGTCTTTCCCAAGCTTGGACAAGACACTCCATGGCATTTCTTGGTTGAGTTCTTGGTTCAAATCCTTCTTAGATTTTATAGATCATACAAGTCCTAGGGAGGACTACATCAGTTCCATGTGTGGATTGGTTGGAAATTCTGCTAGAGTGCCATGAGCTAACCGTAAATGCAGCTGAGATCTCGAGT
Chr07	28,299,616	C	T	T/T	C/C	TA=intron_variant	TGN=Phvul.007G167400	CAAAAAGTTTAAAAGTCATAAACTAATTCCAACTATTGGTAGTTAGAGTTGGTTCATGCACCTAACTACCTCATTATCACACGAGACAAGGAAGTTGCAACTTAGATTCCTTGCATGTTGGTTTTGTTTGGCCAACCCATGATTATGGTCATCTAATTGTTTGTTTTTGTTACCCTTTTTACTTCTTGAACACACCTTATCATCCTATCCTACAAACAGAAGGCTCTTCTCATGTATTTGAGATAAATGAGAATTAGAGTTTTTGTGAGATTTTCAAGTGTCTTGAATTTGGTACTCTTAGCTTAGCTTTGTATATTAAGGAGTTTCTTCAAGTGGCGAATCAATAATGTACTTATCTTTGAAGTCTTTCAAGGTGGTGTGTTCTTTTTCCATCTTCTACCTTGTCCATTTGATCTTCTTTTTACATTTGTTTCTTTGTCTTTCCCAAGCTTGGACAAGACACTCCATGGCATTTCTTGGTTGAGTTCTTGGTTCAAATC	TCTTAGATTTTATAGATCATACAAGTCCTAGGGAGGACTACATCAGTTCCATGTGTGGATTGGTTGGAAATTCTGCTAGAGTGCCATGAGCTAACCGTAAATGCAGCTGAGATCTCGAGTTAGATGGTTTGCATCAAAGAGAGAAAAGACAATTGTTTTTTAGCCTTCAAAACGTGTGAGAGTGGGAAAATAGGGTGTGTTCTAATAAAATAAGGGGAAGAGAGAAATCCCAAGATATCCCCCCTCTTGCAAACAGCACCAAACAACAATGTTTGAGGTCACTAAAGGGGGGATTTAGTCTCTCATGCAATAGCAATTGCAAGGCTTCAGTATCAACCATGATTCACGGAACTCCTTGCACAACAGCAAGTTGTATTGTAATGCCATGGGTTCCACTGGGCGACCAATAACCATAATGCTCCACAAAGCCGCTATAGCACACAATTACTACTATTGTATAAAGTGAGACCCCACAGTAAAACTTGGAGCTTTTTAATCAT
Chr07	28,302,046	A	G	G/G	A/A	TA=synonymous_variant	TGN=Phvul.007G167400	GTCTATGCCTCATATCTTGTTTTGTTTCGAACAACTAACATATTCACCACAGTTTTATTACAAAAATTTGAAAAAGGGCAACCCAGAATTGAATTTGTCTCACCATCATTTCCACTGACAGTTACTAGGTATCTCTGAGCAACCAAGTCCATTACATGGCCATAGCGAGGTCCAGGTCCTTGCCCTTGTACAACAACCCTACAATTAAATCAATTGCAACATTGAATAATTGCGTGATTTTGTCAAAAGTCTCGACGTAGGAAAAAAGAAAACATCTATTTACAACCAACCTCACCTGTGCCATTTGTATTTATCGTTGGTCAAGTCAAGCACATAGAGATCGTCAGTAGAATGCCCGGCTGGTCCTATCCCACCCTACCCAAACAGAACCAGTCAAACAAATGTTAAGATGGCTAGACTAGTCAAGAAGATGGGTCCCAATTCCCACCAGTAATCTCAATTCTGACAATCACCTGAAAAACAACCATGGTACCAACTGC	CAGCTGCATGAGCAGCCCGAGGAGAAGGCGGCTCTCCAGCCGGTTTAATACTAAACACTCAGCAGAAAAATCATTACTTTAGTACCAATTCATCAATCATTAATCTTCTTAAAAGAAATACAAAAAAAACTGAAAACACACACAAATAAGCTAAATTCAGAAAAATTCAGAATTGTGAAATCGGGGAATAAACTCACCTAATCCATTTCCTAGTTTGCACGTCATAGGAATGAACAGTATTTGTGACTCCAGCTAACCCTGCAAAGTTATTAAAGCAGCGAAATTCAGGCCACCAAAATCGGAAAAGAAAGCAAATTGAGTGTTTGTGAAGGAATTTACTAATGCCGGGAGCGGATGAAGATCCTCCTTCAATGGCCGTTGCGCCGCCAAACAGGATCAAGCGAGGACCCTGCGTCTTGGTGGCAGCAACTGCAGTGAGGGTGTGCCCACAGCGAGGACCAGGCCCGTCCTCATCGGTGTCCCAGAAAGTTTCCAAATTT
Chr07	28,304,919	G	A	A/A	G/G	TA=intergenic_variant		CTATAAAACAAGTAAATTATAATTTGTTGTCAAAATTTAAAATTTTAATTCATCTTTAAAAAAACAATCAAATCAGTTTTTTTTCAAAAATAATATTTGCATAGTATGCGTTCCTTTAAGTATGAAGTTACTATGACCATTTAAATCATTAATGATTTTTTTAAAATAATTTTTTTTAATCTAGTATATCCTTCATCCACATTCAAAATTATAATATTTTGTAAAGAGGATTTTGCCTCTTTTAACATAGGCTTCTTTATTAATATATTTAAAGAGCTAGACCCTTCTTCACCAATCTTGTTAAACCTACCGTTATATATTTTAGGGTTTTAAAATATTGGTTAATATTATGTACCAAACTTTATAACATAATACCCTATAGGAACTTTCTACTAAGTATAATATTAATCCTATGATTAGATTTAAATTTTCATAAAAAATGGGAAATTTATAATATTTCCAAGTTAATTGATATTTATCACACTTGATCTTATTATTTA	ATATAGATAAATATTGAATATTATATTTTGTATTTATAATTTCTAAATCAAGTAGTAAGGTGGTTTGGATTGGATTTCCAAATCCAAATATTTGGATAAGTTTTAAATCCAAATCCATATTTTTTAAAAAAAGAAATTGGATTTTTATAAATCCAAATCCAAATATTTGGATTGAATTTTAAATCCAAATCCATATCTTTTATAAAAATAATTTGAATTTTTATAAATTTAAATCCAATCTAAATCCATATTTTTATAAAAAAAATAGATTTTAAATTTAAATCCACATTTTCTAAAACAACTAACATAATATTAATATAATAATATATAAAAGGAATTTAATATTATATAAATATTATTGGACTTTGTTGCCCAAAATTCTTCTTGCACTGATTCTTATTGTTATTATCATGTAATTTACAATAAATTTCTAAGTTTAGCTTAGTGTTTTAAACTTTTTGAAACGTAAATAATTTGAAGATAATCAAATAAAGTAAGAG
Chr07	28,330,564	G	A	A/A	G/G	TA=intergenic_variant		GTTTTTTATTTTCGGATTAAATTGAATCTTGTATACTTGGGATTGGGAAATTCAGCCACTGGAACTGGTAAAGATACATCAGTCACGTTATCTTTTCTGCAGCAAATGATTATATTTGGTCATCTCATCCCTTTATAACAGGAGACAATCGTATCCATGGCATACATGTCCGAGTCATCTACTACCATGGCACGCAGCATATTAAGCAAATAGTCTACTGAATTACTGATATTCACCACTAATATCCTTTGTTGTATTGGCATAAGTGTTTTTAGAGTTTGAACTGGAATAAATGGCGACTTTTTTGGCTTTCAGGCAAAGGCTGGATAGCAGATACAACTTGTAGATACATTATGAAGACCTCGTGGATATGATTATTTCCCATAAAGCTGCTGAAATAAAGTACCGGATAACTTGGGGACAATGCCAAGCGGTATGTGAATGCTAGCTAGACTATTTTCAGACCATTTCAGACTATGTAGAGCATGGCCAAAGCTCGG	ACTGGATTATAACAAATTAGGTACCTATATGCCATTGCTATCTTTTGAAGAACCATATCATGCATCAACATGACTAAGTAACCAATCCAATTACAATTTTCTCCGTTATTCCTGGACAGGATTTGAAATTAGCAACTGGATGTTGTTTATATGCAAGTGATCGGTGTGGAATTTTTCTATTCAATAGATGATTTTTATAGTTCTTAAGTGTTAAACCGTTTACAAATGACTGCACTTCATTGTGTTTAGGTGCCAAACCGTTTCAGTATGTTACATTATGTGTTCATGCTTTTCTCCTGCCAAACTTGAACTATAGCATTTTGTTCTCTCTCTATATGCGTGTGTATATATATACCTATCCAAATCAATGTGTCAAGCTGCAATTTGCAGATCCCAATAGCTAGATTGAGATGAATTTATAGTTATGTTGCATTTAAATGAAATGGAAACAGTATGAATCTATATGAAGAATAAATGAGATGTGGTAATTTTATACTTAG
Chr07	28,336,242	C	T	T/T	C/C	TA=intergenic_variant		ACTAGAATTATTTCTCCTTTTGACCACAACATAAAAAAAAGTCGTATTTCGATCTCCATCTTTGAACCATAACATCTTAGCCATTTCTTTCCAAAACTTGACAGTGAAGAGCATGATCAAACTCCTCCTTAGCAATCTTTGCTTGACAGAGAAGCCCATCAATATCAGACAAACTAGCAGTCTCTAAGTTTTGTTGAATACCAAGGAGTAGACCCTGCTTTAGAAGATATGCATTGTGAACATTACCAAAAGAATTTTTATTTCAGTCTCGGAGTTCAATTTTCAACCTTTTTAACTTATGTTGCAAAATAAACATAGGACAACCAACAACCTTATTAGCCCAAAAAATCATAAATAAGCTTCAGACATAAAGTGTCCTGAATCCTCATAAAAAAGAAACGAAAATTGCTAACCTTCCTTAAATAATTACTAGCAAGGCTAGCAAAAATAGGGGAATGATCAGAACAATTTTTAACAAGAACCTGATAAGTACAAAAATC	ATTCATCCATACACACTCCATTACATAAAGCCCTATCCAGTCTTCTATGAATTCTATGCAACCCTCTCCTTCCATTACACCAAGTATAACACGATCCAGTAAAAGACATACAAGAAAGATCTTTAGTATTAATCCAATCAAGGAATTAATTACAAGAAACCTTATTAGGAGCCACCCCCTTTACAATCATCCGCCAAGAGCTAAACATTAAAATCTCCCATAATACACCAAGGTCCTGTGTAAGAACTAAGATCTCTCCACAAAAACCGTCTAACCAAGTATGTGTTAACACCATAAAAACCTGCAAAACTAAAACATTTATCATGCAGAAGACAAGTTACAGAAATAAACTGATCAGTGACCAAGAAATTTTGGACAAGATTAAAAACTGACAAATACCAAAGATTAGCAGCTTTATTAACAATAGGAGTCGTAGCCACTAAATGCATATTAATAGATTTGAAAAGGACGTCGACAACATCAATGTACGACATCATAAG
Chr07	28,342,987	T	G	G/G	T/T	TA=synonymous_variant	TGN=Phvul.007G167600	GAGTTTGATTTGGTGGCGCAAGTTGAAAATTCCTCTTTTCCTGAAGATTTGTAAAGCTCGTACCCTTTTGTAATCGTTGGTTCTGGTCTAATTTGTCAGCTTTTCCTTTCGATATTTGCTTGCTTTTCTCCTATTGATCTGCTAATAGTGTTATGATGCTTTCTGCTTAGTGCCCCTGTTGTCTAATTAGAGGGGAGGACCTTTCACCTTAGTGACCTAGTTATCTATCTGAATACAGGAATCAATCAACGGTCTTTTTCTAATTGCCACTACAGTGTTAGTTCATATAGATAAGGTTCTAACTTGTAATAATATGTTCAATTTTTCTTGTAGAGTGTTGTTCCACTCAATACTTGGGTGCTTATCTCCAATTTCAAGCTTGCTTACAACCTACTAAGACGTGCTGATGGAACGTTCAATCGAGAATTGGCGGAATTCCTCGACCGCAAGGTCCCGGCTAACGCAATTCCTGTTGATGGGGTATTCTCTTTTGATCATGT	ATCGAAACACCGGCCTATTCAATAGGGTGTATCAAGTAGCTCCTGAAAAGGAGGCTAGGTGCAACATAATAGAGCTGGAGAAGCCCCTGAGCACTACAGAGATCGTTCCTGTTATAATTTTCTTCCATGGTGGAAGTTTCTCTCATTCTTCCGCTAACAGTGCTATCTATGACACTTTCTGCCGCCGCCTTGTGAGCCTTTGCAAGGCTGTGGTGGTTTCTGTAAACTACAGGCGATCGCCGGAGCACCGTTATCCATGTGCCTATGATGATGGCTGGGCAGCCCTGAAGTGGGTTAAGTCTAGAAAGTGGCTTCAGTGTGGAAAGGATTCCAAGGTTCATGTGTACTTGGCAGGCGATAGTTCTGGTGGTAATATTGCTCATCATGTGGCTGTGAGGGCTGCTGAAGAAGATATTGAAGTGCTTGGCAATATTCTTCTTCACCCACTGTTTGGTGGGGAGAAGCGAACCGAGTCAGAGAAGAAACTGGATGGGAAGTAC

Chr07	28,367,607	C	T	T/T	C/C	TA=intergenic_variant		TATCCTTGAAGCAATTACAAACCAGTCGCAAAATCAATCTCCTTTACAACGACTCTAGGTTTATGCAACAAAGTGGGTGAGACTATTTTTAACCAACACACTATATTCATAAAGCCCTAGCTAAAGTAATAAAAGTTTAAAGGAAAATAACCTAACGATTAAAAACATCTTAAGTTTAAATGTTTTACAGACTATTAGGAACCTACATTTAAAACTTGTATGAATTGGGACTCTAAGCATGCACCAATCAAATGTTCACCGTGGTTTGTTACACTCTAAAATTATATTTCTTATGTATATAGATTTATACATATGTAAGTGTACCTAAGGGTTTTCCTATAGCATCTAGTGGGTTAGAATGTTAATTGACATGCATCTAGTATAAGTTGTGTTTCAGTCAAGTTATAATTGGTCAAACATCTTCATATGAGAGAAGGCAAGTGAGAGGTTATTTTAAGTTTTATCACCTTGCACAAAGAACTTGTGTAACATTATAATCT	CCTTCGTGTATTTCAAATATAACATATCATAGAAAATTGTTACCCAATTTTAATTTTAAGTATTTTCTTTGTAATTATTTGGAAATTCTTTTCTCGGATGGTTAGTAATTCTTTAAAACTATTGAAATAAATTGATTTTTAAGTTGTGATGAACTTCATTAATTGATACAATTCTCGAAATAAGTAATTACTGTGACTATATATCACTAGATAAGAAAAGATTTAAAAAATAAAAACACCCATATGTTTTATCAACCTATCAAAATTTCTTTTTTATTAGCAAAATCGATCTACCATCTTTTCTCTTTGAATGTATTGCCATTTTTGTTACAATTTATTTGGAATATAATTTGTTAACTCAATACGAATTGATAAATTTGTTAGGTTTGATTGATAATAAATTTCTAATTGTGTCCCTTTCATAAACCAATCTACATTTCCTTTATTTTAGTGTTTGTTTGATACTATTCTTCAATTCTATTATTTGGATTTTCATTTCA
Chr07	28,373,221	A	C	C|C	A|A	TA=upstream_transcript_variant	TGN=Phvul.007G167701	TTAGTTGTAAAAAACTGTTCTCAATAGATGTTACATATATCGTTATTTAGTATTGTAATAAGTTGAAATTATTAATTTTTTTTTGGAAGGGATCATAATGGATTATGTCCCATAATCTAATTGATTAGGATATATAAGTAATTGATTAAAGGTATCCCTATAATTATAATAATCAGTTAAGATTGTTAACTTAATCATTTAGGCTTAGGCTGAGGGTTAGATCATGCTCCCTTCCATACATAATTGATTTTAAATATGATTTATTTTTTCTTTAATTACAAATTTCAAACACAAATATACTAATATATGTATCAATCTTAGTATACAAATATTCATAAATCCTTCACCATTTCAAGATAAGAGTATTTAGATAAATTCATAAATTATACAATATTTTTTTTACATTTTTCAATCACATCAATCACATATTCTACAATTTTTCTAGTCAAATTTATTCACTTTTCATTCATTTTTCTTATATCCAAAGAAGATAATTTTTC	ACTTATTAAATCCTTTCTTTTCACTCTCTTTCCACTCGCACCACGTTAAAACACGTTGGGATGTTACCATCCACAAATGTTTAACAAAGTAACATGCATTTAATAGTTTTTCTTTTCTCAAACATAAGCTATTGTTTACCACTGAGTTTAACTTTTATATAATGTTAGTTTAGGATTTTATTTGTTGTCTATTACCCAAAAGTTATTTATAAAACAATTCTTACAAATATTAATCCTTTTTATTTACATACTAATCATAATTACATAACTTTTTTCGTAAATATTACTTGTTTATCCATCAAAACTAAATTTGTTGTAACTCACTGAAATTATTACAAATGATATTTTTTGTTAAAAATGTAATATAACTGTGAATTCAATACTTAAAATCGAAATGACTAATTTATGCGTTCGTAATAATTTACTGAACATATTTAAATTAAGTTTCGAACAGGATATTACACCCTTTTCCCTGCAACAAAAATTTATTTAAATTAAAT
Chr07	28,377,571	A	G	G/G	A/A	TA=intron_variant	TGN=Phvul.007G167701	GGTTAAATAAATATTGGACCTGAAAGTTGTTATTAGTTAAGTGCGGTGGTTCTTCTCTTTGACCATAGTCACTCATTTCAGAAATTCCATGAATAATCATTAGAGTTGTTTTAGTTTCTCTTTGATTGTTGGTGAAGAAAAAAATTCTAATATACAAGTTTGGTTAGGTCTGGGATCGGCGTTGCTTGATTGCTAAAGGCAAGCCAGCAGGGGTATTGATGGGACACCTTGAGGGCATTACATTTATTGATACTCGTGGAGATGGGCGTTATTTCATTTCAAATGGTAAAGATCAAACCATCAAGCTCTGGGACATACGCAAAATGTCATCCAATGTTACCTGGTATGCTCCCCACCCACCGACCCACCTCTAATATTCTGATGCGCACATTTCTTCTTAACGTGTGTGGAATAAAAAGCACGAGTTAAGGTCATTATAGGATAATATTAGCTGATGAGTTTTGCAATGGTACCCAAATTATCGTAAGGTATCATGTTGGA	TTGAATTTATATTAATTCTTAATCTATAGGCTAATTATGATATATTATCTGATTATGAGACTGATTTTTTTTTTTGAATTATCTATGAAGCGTTATCACTACATTCTTTTCAATGGAAAATACTGAAAGTTTTTTTATAATTTTGTTACAGAATGATGTGTCATGTTGGACTCATGCTTCTGTCTCTATTTGTGTTTGCATGCCTGTGTGGGTAATTCAAGCTCCGATAATGATTTTCAGAATTCAGTCTTGCATGTGATATTTATAGATTTTTAATCTAGCTAATGAAGTCTGTTCCTCTCCGAGATTATGTTGTTTCTTGATGAAATTGTTGTCTATTCATTGACATACTATAACAGAACTTTCTCTCTATTCCAGTAATCCTGGGTATAGGAGTTACGAATGGGATTACAGGTGGATGGATTATCCACCCCAAGCAAAAGACTTGATTCACCCTTGTGATCAATCAGTGGCTACTTATAGAGGTCATTCGGTCTTGC
Chr07	28,411,479	G	A	A|A	G|G	TA=intergenic_variant		TTTGAACAAATTGGTTTGATGGACATTAATTGTCTAAAAACTCCATGTGGATGGCTATAATATGGGGTATCTAGAATTAGAGAAATTGTATTATTTTAAAGAATGCACTGGTTGATGCTATTAAAAATTTTACTATGGTTCAAATGAAGGTTTGGGCATGGATTAACTATAAGTTTTTTAAATCAAATTTTTCTTATTCTGATTAGTGTCCTAATTTATATGTGTGTTTAAACTTTATTGATTGAATGACCTTTATATGATTTTCATGTATCATGTACAAGAAATTCTTATTCAAGGGCATAGCTAAAGCAAACATGATAGAAGAGGTCGTGAAATCCTTGAATGGTATGCAATGTTTTGAAAGATAAGATAAATTGGTTGTATGTATTTGTATCCTTGAATAGTTTTATGCAACTTTTTGTTGTTGGGTTGAAAGAAAATGGTTCAATGAAGATTTTTTTTAATATGTTAAGATAATTTTATGCTTGACGGTATTTTGG	TGTTTTATTTTTTATTTAATTATCCGAAGGTAAGCTTTAGATCTTATTATTGATATAGATTTATGTTTTTTATCAATTTTTGTTTAGGGATGAATTTTTTTCTGAGTTAGAATATAGCTAATCCTCCCATTTATGTAATTCTGTATTGTGATAAAAAAAAATATTATAATTCGTTAAGTTCTGTATTGGTTTTGCCCTTGAAACCTAATTATCCTAAAAAAACTACCTCTATTCAGAATAACTTGAAAAAAAATATTATTTTCTAACGGACCGCTTAAACCAACAAAGTTGAAGTGATTAATTATGTTCTTTCTTAAAAAAAATTCATTCTCGGATTAAAATTTCGATTCCAAAATTATGAAATTTAAAATATATTTTTTGGGCTACTTTTAAAATTCCATATCCTTTTCAAAAGTTATAATAAAAAACTTTTTATATATATCTTTTTCTCCTAACGAAACATAATTTAAAAACAAAATCATCAAACTCTAAAATAAATA
Chr07	28,436,926	A	G	A/A	G/G	TA=upstream_transcript_variant	TGN=Phvul.007G168200	TTTGAATAGGAAAAAGTTAATCTGAATGTATAGATTGAAATTGGGAAGTTCACAGCTTTGTCATAACTTATAAGGCAGGTATGGGTTTCATATTTTTATAGTTTTTCTTGTTTGACTGAGACAGAGAAGTAAGGGAATTAAGGGAATGAAAGGAATATTAATGGTAATAATGAGAGTAAATTAAATGAAGAAAGAAGAGAAGGTGGAGTGAGATTCTTCTTTGGAAACAGGAAACCCGGCGTACCCATCTCCAATGAACCTAACTACCATTACCATTTTACAGAATAATCTCAAGAGTTAAAAGTGTGAAGTTGTGACTGTTGACTCTGACTCAGACTCATTGTGCAGCTGTGAGAACTTCTCTTGTGTGATCAAAAAAATCAAAAACAATGGAATGTGAGCTGGTCTGGGACCCCATTTCATGAACTTAGGAAGTTGGAATGGACTCAAACACCTTGTCAAAGACCAAATAAAGCTGGAATACCTCACCTTTCTCCACT	TTTTAGCTTCCACCATTCACGCCAAAATCAAAATATTTTTATTTTTATTTTTGGTCACAAACTTACAGAAGATTCAAATCGAAGTTCATACTGAACTAATAGACTTGTATGTGCCTAAGTATATTATGCATAGAAAACAAAATAATAAACACAGGAAGGTCTGTTTCACAATGTACCCTACACGGTTACAGTGACAAAGGAATGTATTGTTTGCCATATAATGTAACACTAATCATCACGTATAGTCACTTTCTAAAGGAAAAGAAGAAGCAGAACAAGAATTCTGAAAGAGAAAGGTGGAGTAAAACTACCAAAATTCTTCTTCAAATATTGAAATACTTTAATAACTTGTAAAACAGGTTGACTTAGATACTAGGATAAATTGAAGTAGATTTTACAGTTTTTTATTTAAGTGGTGGGATTTGTACACTTGTTTTCCCATTCATAGAGAGGGCTATGCCAATGTGAGCTTGCATAATAGTGTAACAGTGATTCTATAA
Chr07	28,442,190	C	T	C/C	T/T	TA=intron_variant	TGN=Phvul.007G168300	CTCTCTTTTGCCAATCTACCCTGTAACATTTCCACCGATTGTGGTATAGCAATAGAATAATAAGCTGTTTGAGTACCCATTTTAAACAAAGGGGACACGGATATAAAAGAAAAACTATTATTTAAAAGGCACTTGTATATATATGTAGACGAATACAAAGGAGGTGTATTATTGAGAAGCCTATTTCTATACAAGAGGAGCAAATCTTAGCTGCAAAACTAAATATTGACGTCCAAGGGATTATTAAAAGAGACTTGGTCACACAACACAAGACCATTAAGTCACATAACTTCGTCTTTTATCTTGACTATTATTGGTAAGATTTAATGATTAATATTTGTCTCTTACTCTTACATAAAAACTAACCCTCTCATTCTACAAAATCTACTCTGTGATACCCAACAAACAAAAAAACTTGCAAGGAAGACTTGTGCGATAGATTATTGAATGCGCACTGTTACTATACTTCTGTTTAATCTGATTCTAGACTTAGCACCTCT	CATGTTGCAGTACATGGCTATAATTGTCAACCACGTATACGTAGACTAAAAAATGTATATATATTTATTTCAAAGTATCCATGAGTGTTCAAGGTGCCTAAACATTTCCAACATAGGAGCATAAGGTTTGGAGTAGGACTGTCAAAGTAGAGAGGCAGACACTCAAGATAGACGTTGACAATACATACAAGTAAACTCCACAGATTTTTTCAGACATATCTTGATATTTGGAACTAAGTAGTTTCGTATTTATCACATACCTCAATCTTGGCTTGAAGATTTTTCTCTTTGTGTAGCTCCTGTTGCAGATTAAATAATTTGTGCTTCTCTTTCTCAAGGTCATCTCGAAGCAAACTGTTCTGGCCTTCCCATGACTGGGCCTGTTTAAGAGCCGTCTGCTCACTCTCCAGTGCTTGTTGATGGCTTGCCATTGACTCTACAACCCACAACTTAGCAGCATCCAACTCCTTTTTTAGCAAGGAGTTTTCTGCCTCTAGCAT
Chr07	28,445,243	T	C	T/T	C/C	TA=upstream_transcript_variant	TGN=Phvul.007G168300	TGTGGGCAGGGCTTATATAGTTCAAGCAGAACAGGGAATGGAAGCCAAGTGCACCCACATACCAATCAAACACTGACAACCCATAACACAAACAATGAAAACGTGACGGATGCCTTCCAACAAGGCACACACAACAGGGTTATTGAAAACAGAAACCAAAAAAAAAATTCAGAAAATCAAGAAACTTTAACTTGGAATCAGCAAAGAAAGCAAATGTACCTGACGCATGCATTGTTTCCATAGGAAACGTTACGGAAGAGGATATTAATTTAGGTTGGCGATTATGGCGTCAAGATTTTTGCTTAATACGCAGTGTATGTGTGCAGCCACAGTTCTCACTCACAAGTTTCTTTGGGTTCTTCTGGGTTTTGGAATGATCCTCTTTCATACATAATACTGCGGCTATTCGTTTAGGCGCTAACAACTACTCTACTTGCGGTTAAACCTTCTTCTCTTTTTATACCCAACCTAATACCCTCATCTACTGCATCTTTTCATTT	AAATTCAATTTTTTTTTGCATTAAACACCAAATTTCAAAGTTTTGAAGAAAAAAAAAAACAAATGTTAGGGATACATAGACTACATTTTTCAATGAATCTACAGTATATTTCAATTCAAAGTAAATAGCTAAGAAATTAAAATATTGAGGTTTATTTAAAAGAAATGTTCTTTTTCAACGAATATTTTTTTATGACAAATTAATGTTTATTTTTGGATTTTATTTTAATAATTAATAACTGCGAGGATTAAATTAAATGATTTTTTTAAAGATAATGTTTAGTTCATTAAATAATTTAATATTTTCTTAACTTTGTTTTGCTGTAATTAATTTCTGCTCGTTTCTTTACTTAAAAGAATAAATTTAGCTCTGACTAATTCAACATGAAAATTAAATGACCTTATTTACCGAGGAACACTCACACAATTTCTTCGTTTATAATAATACAATTTGAGATTTTTATTTTTTTTTAAATATTATTAAATATCCATCATGTGTAA
**CHROM**	**POS**	**REF**	**ALT**	**Sel 1308**	**BRS Cometa**	**Functional annotation**	**Gene**	**Flanking (-)500bp**	**Flanking +500bp**
Chr08	2,135,846	T	C	C|C	T|T	TA=intergenic_variant		TTTGAACAGTGTCTTCATCCAAAATGTTAAAATGGTTACATAAAAAAACTGTCAAAATGGTTGTTGTCATTTTGAATAATATTATTAAATTATATTAGTTCTTTATTATGTTATTTTCTTTTAATTGTTATTTTTTAATAACAACATTAAAACATGGTAAATCATTATTACAACCAGGTTACTTGTAGTATTCTGTTCAATCAAAACAAAGAGTAAAAGAGATAAAAGAAAATATATCAATTGGTAAATTTTAGAACAGTTATCTTTTGTAGGAGTTAAATGGTGAAAAATAAATATGTAAAACTGTTTGGTTTGATGAACAAAGTGACAGTGACAAAGGAGATGAAGTAGTATGAAATTTACACCTCATAAATTTTCAAACCAGATTACATTTAAAAAATACCTTGTAATGACTGATTTTGTTGTCTTTTTTACATTTTTTTATCGTCAAAATTACAGTTTTGTATTTATTTAACAAAATTATTGGGTTTCAGCATGTT	AATAAAATCTGCAAAATTGGGTCTCATATTTTTGTTTTTTATTTTGAAAACCTATATGCATAAATTAATACATGTGCAGACATAAAATATGTTTTGTAACATCCTTTAAAACTTTGCATTTGTTAGTTTATATTGAATTGTCCTTTTAACACAATTAACCTGTTTTTTCCCCTTCTCATGGTATTTAGTAAGAAATTGAATATAAGCTAGTTCTAAAGCTTTAAGTTACTAAAGTATAAGTTTTATTTTGTTCTATTAAATTAATTTCAGAATTTCAGAACCTCCTGTTATAATATTTAGAAAATTAGTAAAAAATAATAATCTTTATATCGTCATTTTTTAATTTGTGAAAGCACGTATTTCAAAAACTATATTCCTTATAATTAGGTCCCATCTATGTTAAAATGTAAAGGTTATAACTTCTTTTTATTGCAAGTTACAACAATCAAATACGAACAGAATTTTCAGCAATCATATTGGTGAAATAATTTGAATCATTA
Chr08	2,170,050	G	A	A|A	G|G	TA=intergenic_variant		TCAGTGCGGTTATAATGAAAGTATCTACTTGTGAAACTAAAATATCACTTTTATGTTGCCCTTATTAGTATAAACTATATATATTAAATAATGTTAAAAGCTTGAAACTGATTTCATGAAACTGGGAAATTTTCATTTTAAAACCAATTTCAATGATTGTTTGAAGATAGTAGATCTTCACAGGTGGTTAGACCTTCTATGAGGATTTGCCCTATGTTTTTCTGTATATCTTTTCCAACAAGTCGTTACACAGACCGTCAATCCATATACTACCAAGTTTTGAGAAAATTGACACAAATGTACATACGAATAATAAAATACTTACAATTCACTTATTGTCTCTTTCTATGAAAGTCTGAATAATTTGAACCTCAAAACAAACCTTTTTAACTCTAAAAGATTGCTCAAATCAAATGTCTTCATCATTTCTTGAGTCAACCTTAAATAGAAACGATTTATCCAACAGACTCTTTCGAAAAATTGTCGGGGTCTGAATAAAA	AGATATTTTAATGTATAATATAATTAAAATATATATTATAATTGACCTAAAACAAGAAATCACAACTGTTTGAATCTTCGAACCATTTACACATAGTTGTCATTGCTAATCAATTATCACTACTTTAGTAATCAAGTATATAGGGTGTGGTTATTATTATCGGTGGTGTGCTTAGTTCTTATCGGGTGTGATGTGTTAATAATTGTTAAAATTTGTTTACCGTCAATAATATTATTTAAATTCAACACAAAATAATTTAATAATTATATTTCATAAAATTTTGAATATTTAATTTATTTTTTTAATATCACCATTTAAAATGTTTATATTGATAATCTTCCACTTCAAAATGTTTACAAGTTATGTAAATTTTGTTTTCACAATGTTAACTAACCGTATTTTAAAAAAAAAATATTAACTCAACACTATTTCTTCATCTTCCTCTCTGATTTTCATATATTCATTTAAAACTCAGTTAAAAATATATTTAAGTCTTAAAA

Chr08	2,174,575	G	A	A/A	G/G	TA=intron_variant	TGN=Phvul.008G026500	ACAGCGCGATGGACTAGTGTCAAGACGTGAGAAGGGTTCAAGTCATTTGAATGAAGAGTC<br>AGAACCTCTAAGAGGTGATGGAGATGCTGACTCAGAATCTTTAAATTTGGGTGGCAAATG<br>GAATGACACCAGTGAAGATGGATCAAAGGAATACCGTAAGTCCTCTGTGAGACTTTCATC<br>AGCAAAACTTACAACTGGAGCTGGGGTTGTGTATTCATCATCAGAAGAGGAGGATGTCTG<br>TCCGACTTGTCTAGAAGGTAAGCTCTTTTTAACTCAAATTTTAATCGATAGATCACGTGATG<br>CTGCAGCTCAATTTTGCATGAAAATATTGGTTTGTAAGGAAGTTATCAATCCTGGATAGTA<br>GTCAGAATGGTCAATTTCAGAACTGCTATAGCAGGATTGGCTCTTCTATTGAAGATTATAG<br>ATACAAGTTAAATATTTTATACTACTCATTATGAAATGCGAAAAAACAATTTATTCTACTCAT<br>GACATGATCGTAT	TGGGTTAAATTCACTAGTCAGTAACCACTATGTCATATCAGAAGTTTTTGCTTCTTGACTTTTC<br>TTCTGTGTTCTGCTCTATAAATAGTATAAACTTATTTATAGCTTTTAATTGCAAATTAACAGTAA<br>TTCTTAAAAGTAAGGAAACAATATTAATAGATAATACTATTCCTTATAATTAATCACCCCCATAT<br>ACTAGGAAAAAGAGTTGGCTGAATGCATGAGACTCACATTTAGAGGGTTTTCAAAGGGTAGAT<br>GAACCTAACTCATCTGCATGCTATTCCCATAATTGGAGTTTGTGATGTCCACAACTCCGTGTC<br>ACAAGGCAGCAAGCTTACTGCTACATCAAGGATCACCATGAGAACTTGCAACGTCTTTGAAAA<br>AGTAAAGAACACATCACGTGAGACAATCTCCCTTAGCACATGACAGAAAGAATTTCAAAGAAC<br>TGCATGGAGATGCTGATTAGAATTAAGGATAGCGACCTTTCTGAATTTGTGAGG
Chr08	2,279,702	A	C	C/C	A/A	TA=intergenic_variant		AATAAAAGTTTCACTTCCCTATTCCCACAGAAAAGTAGTTATTTTCTGTCTGATTTGCAAAC<br>TCTACTTTGCAACATGTTCATCTAGTTTCTTCAAGCAATATCTGTCTCAACTATTACTGAAC<br>GGAACTTTGTTTAGTTAGCTTTAATTGTATACTTTAGTTAGCCTTAATAACAAAGAAGAAAG<br>CATATTGTGACATGTTTTTCCAAATCTGCACATCAAAACATATTTCATGATATGCATTTGATT<br>TTTGGTTCACCAGTATGCCAAGATTTCACCTTATACTAGTTCGGTCTGAAAATTAAAAAGCC<br>TGAACAATAATTTTTCTGTCAAGATCCAATTTAGCCTTTGATTTAGTCACATTCACAGAAAT<br>AGGATGATTTGAATAAAAAGAAAACATGAGATGATTGCAGAAATGGACTCAGGGAGTTAAG<br>TCAAAGAGAGTTCTAGGCGATCCCAAGTAAGTTAACGCCCACGACACTTTGCAGGGACAA<br>CAAGCC	AAAAAATGTAATTAAACCAAAACGAGTGACTACAAAAGGCAAGCGAGTTAACCAAATCTAAAC<br>AAACACTTGTGAACAAAAGTTGTTATTAATGTTTAATATAAATTTGCTTATTGAAGGTTTATTAA<br>GAGTATTTAGTAGTGTAAACATTTGACTTTTGTATTTGTAACCATTCTAATAATTATTTTTACTCT<br>ATAAAATATTTTTGAACTTGGCATCAAAAGGCTGCAAAACTTACGTTCAAAATCACATTTTCAC<br>TTTGATACTTTAACATCTCCAACATTTGTTATTATATCATTCAACTTATCAGTATTCAAATTTATA<br>ATATACAGTCAGCTTTATCCTCTCAAATTTTGTATATATTTTTGCGCATCAGTGTGATCAGCGA<br>TCATTTGTGTAATATTATAAAAACAACAAACAGGAAAAATATTATAAGCCCATAACACCAACTA<br>CAACCATTAATTAAAAAGATGGATTGAAAGTTGTAACGTACACGTTTA
Chr08	2,292,319	T	C	C/C	T/T	TA=upstream_transcript_variant	TGN=Phvul.008G028500	TGAAGAAGTGGAACTGGAAATCATCACAGAAGACAGTGATAGTGAAGAAATGTAACGAAT<br>TCACACCTAATTTAAGCTGAAATTAAGTTTAGTGTTTTTGAATCAAAACAGTGACATCTGCA<br>GAGATCAAGTTTAATTGAGATACCTGTACCTTTTATTGTGATCTCTGTTTCTAACATTATCAT<br>CTTACCGGAAGCTGTCATGGCAATAATCTGGTACTAGTATAAATTTGAAAGAATTGCCAAC<br>AAAATCAATAATGATAGCACTCCCTTGCTTCAGTCTGTAATTTTTGCTCTTCTTTTTCTAGG<br>AACCCTCACCACATAACCAGTCACTGATATATAAAGCAAAATCATACAGATTGCAGCAGAG<br>TGTAGGGAACTCTCTATAGATACCATGATAAATTGCTTTTAAGTACGGATCGGATAAGCCA<br>CCAACAGTATGTCAAGTAGAGGTGCAACTTGACCAATTGTGATTATACTTTATTGTCCATAA<br>CCGTTACC	AAAAAGTGTACAACTGGGTACAATGAAGGGGTAAAAACGGCAATCAAAGAAATAGTATTTTGA<br>CTGATACCTTTAATTTTTCCCTTGCCATTATTAGAGTAGATGAATCTACCAAACCATATCTAGG<br>ACCATATTGGATGAATTTCTGTATAAATATTTCTAGAAAAAAAAAATCTTTGAACTAGCAGTAGT<br>TTTTTCACATCTTGACTAAGTCAGAGTTTTATATGCAGGCCTTGGATCTTATTTGGGGAAGATT<br>CCAACCCAACCTTAATATGCTTCACAAGCCAAACAATTGAGGAGCAACATAGTTCAATGTGTA<br>TGGTAAGTGACAAGTGAGATCTACTTAAGCAAGCAAGGTGTCACAAATTGGAACTATGTTTCT<br>GAATTGTTTGGGCATGTGTTGTTCGAAGCCCACAACAAATACAACTTCATCTCAGAGACAGTT<br>TCCAACGTTGATAGAAGAGCTGTGCCATCAATTTTCTCTCACCGATCTTAGGAAA
Chr08	2,299,493	A	G	G|G	A|A	TA=upstream_transcript_variant	TGN=Phvul.008G028600	TCAGTAGTACCTAAATTTCTTTTTATTGAGAAACTTCATCTTACTAATAAAAATGCATGTATT<br>ATTTGATTTCTCTTTCCATTATTATAACTAACCAAATCTTTGTTTTTGTTTTATGTTTTAATGA<br>AAAAACTAGTTTTTTTTTTTTTACAAATTTGTCCTTCATGTCTACCTTTACTTTGTTTTTCCAT<br>CATCTTTCCGTCTAAAAAACCCATCTTTTAAGCAAAAGAAACCCTAAAATCAGAGAGAGAA<br>AAATCCCAAAGTCGACCCCGAAAGCACTGCAGTAATCGAAATTGCATGAGAGGGTTGAAG<br>ATAAGAAGCTCAACACCTGACCTTACTGAAGGAAATTTAAAAAATATTTTATAAATTTTATTA<br>ATAGTAAAAATATTTTAAATAAAAAATAATTTTTACTCTCTAATGAATAATTATTTTTTACTAT<br>TTAAAGATTCAACAGTAAATAATTGAGAAAGATTTTACTTACAGAATTCAGTAGAAACACA	ATCATTAATTAAAAACTGGAGGAACCTACTAAGAAGTTGTCTATGTCCACTTCGTAGTCCATAT<br>TGAATGCACAAAAGTCCTCCTAATTCACCGTTTGAAATATGTATGTGTGTGCCAATGCATGTA<br>GGGACAAGGAACCAATTTCGTGCAAGAAAAGGTGATACAAATTTTGATATGCTATTTCTTAGG<br>TGATGATATTTTGACACTTTACATCCACAACAAAATATTAATGTTGGTTAATGCTGGTGAACAT<br>TTTGCAGTTTTGGTAGATGCATGTTTTGTGTGGTTTGTTGATCTGCTCTTGGAGCTTAAAAGAT<br>GAGGATGTGTGGAGATTTTAGTAGCTTTTGAAAGTAGATTGGTAGTAGAGGTCGTGCTTCACT<br>TTGACAATAAAATGTAGTGCACAAGGTTGTAGTTTTGGTATTGTTGCAATGGGTAGGGAGTTA<br>GCTAGACTTCAAGATTTTAGATTGTTAAAATTCCAGTGGCTAGATAGCCATCAAGT
Chr08	2,311,064	G	A	A/A	G/G	TA=intergenic_variant		TGGGACATTATATTTCATTGTAACAAAACAAGTGATTGATTATGGTAAACTTGTGAGTTATT<br>TTCAGACTGTTGCAATTAAGCATTTGGCAGAAATTTAGATGGCAAAAGTTTAGCAGCAATA<br>TGGTGGTGTTATGCAAAACATGAAAACTATATAGAAAATGCAAATTATATAAAGATGTTACT<br>TTGGTCAAAACAATATGAATACGTTTTCTCCAATTAAAATTCACTTCATATTTTTCAATTGTT<br>TAAGGAAAAGGATCACAAATTACGTATCAATTCATATAAGTTTCATGCAGATCTTTCATACT<br>CAGTTCTCAAGAGCTGTCAACAGTTGTGTACATGTAACAAGCAGGGTGTCATAGATTAGAT<br>AGATTTTGATTTCAATTACAATAGATGGATTATAAATATTTTTCATTTTATTTATAATTTTATA<br>AATTAATATCAAATTCATTCATTTAAACTATATATTGTAATTTATTTTTAAAAAAATTACTTTAT	AAAAGAAGTTCAATGCTAATACACATTACAATATAAGGATCTCTCCCTTCAAAATTTATGCTCT<br>ACCACTGAATTTTGGATTAATTGTTTCAAAGTATTACAAATATATGTTAGATATAATTATCATTC<br>AATAAATTAATAGTGATACTATTAAACTAATCCATTAACTTTTGCTATAATAACTCAATGGTTAT<br>TGCTTGCATTTATGAATTGTTTGACTCCGCTTTTTTTTTATCAATATATCAACTTTTAGTGTTATT<br>TCTTTTATAATTTTAATTTTGACAAGTTAAGATGAGTAAAAAATATTATGAATATATAAAAACCAA<br>AAAATAACATATAACACAATTTAATACACATACAACAAAAGACAAATGAAATGAAATAATAATT<br>TCAATCTATTTATAAAAGTTATATGAAATAACTTACAAATAAAAATAAAACTATATAGATTTTTTT<br>TAACTCTAGACTTTGATTCGTGGCACTTTAAATAAGGTCCAAA
Chr08	2,326,201	T	G	G/G	T/T	TA=upstream_transcript_variant	TGN=Phvul.008G028800	CATGATTTGTTGCATATTTTAGCCTTTTTTTTCAGTGAAAGCTGTTTCATCCGCATCCCCTG<br>CATGACACTGTGATTTGTCACAGTAATTAAGTTTATGGCATTACTAGGCCTAGCATCTTGC<br>ATTTTCATGAAGAATAGTTTGAGGCTGTTCATTCATGAAGTGGTTTAAGAGATGTCAATGAA<br>CGTTGTTTCAGAGTAAAATAAATGAACAACTACTCTCTTCTGTTGTAGTTCTGGGAGTGAA<br>GAGAAGAGGAAAGTGGGGTAGGGTCATTAGGCATAGACGTGAAGATCCAGGAAAAAGAT<br>AAACTCTCACCCCCCCTCACGAATGGCACTCACATCATCAATGTGTGAAATGAAAAAAAGA<br>GAAATTGGATATGCACAACAAATTTTTTTTAGAATAGTTGTTTTTGTAGAACAAAGAGAGAT<br>ATGTAATTGATGTGATGGAGATATGAAAAAAAGAAGATTGTGGAGATTGTTAAAAAAAGTG<br>TAATAGGAATA	ACGAAAACAATCACACCTGTTGGACAAAACATGAATCTTAGTAATATTTTTCCTCAAACTAGTT<br>AAACTGGGTGTGAGGTAAATACTATTCATTCTTGTACTTTTGGTCTTTACCTAATTGGTTGTGA<br>TTATAACTTTTTTTCTTCTCTTTTGTGAATGGCAGGTATGGCAGTTTTTTCAAATCTCACATTCT<br>GGGGTGTCCTACCATTGTATCCATGGATCCGGAGCTGAATAGATTCATACTGATGAATGAAG<br>CAAAAGGGCTTGTTCCGGGGTACCCACAATCCATGTTAGATATCTTAGGCACACGCAATATTG<br>CAGCTGTTCATGGCTCCACTCACAAGTACATGAGAGGGGCACTGCTTTCAATTATTAGCCCC<br>ACCTTGATCAGGGACCAGCTTTTACCAAAAATTGATGAGTTCATGAGAACCCACTTGATGGAT<br>TGGGATAACAAAGTCATAAACATCCAGGAGAAAACTAAAGAGGTTTGGATCACCATC
Chr08	2,332,769	A	G	G/G	A/A	TA=upstream_transcript_variant	TGN=Phvul.008G028900	GAAATATCTATTTGAGTCACTGAAAGAAAAAGCCAAATATGAGTGTTGGATTTGGGTGACT<br>TTTCTTCATAGAAGAATTCCATAAAAAAGCACAAAGCAATTCATGCATCTCATAAATAAATA<br>AAAATATAGTTAATAATTGGAGAATTGTGTAATCCAATTTACTTTTTTTTTTAAAAATTAGTTT<br>GTTCTTGGTAAGAATATATTTCAGTTTCACACAAATAATTTGCGTTATTGGTCAAATATAATT<br>GACGTAATCAGATATTTGTCTTCAACATACTCGAGAAAAAAAAAACAATAAAACATCATTAA<br>CAAAATCAGAGATTCAGATAATAAAAAAATTAAACCTTTTGAATTTATTTCTTGATTAGGATT<br>ATTCTGAACTTCAGAATGTAAATGTGAATAAAATTCTAGATTTAGTTATGAATGTAAGGCAA<br>ATACACATCAACAACGTTTGAAGTTGAAGGAGTCCTCTAACCTTAAACATCAGACACGTGT<br>CA	GCAAGCTCCCCAACTCGTGCCGAAAACAGCGGACTCGCACGTGCCACTCGTCTATATCATAG<br>TCCTCTACAAACTCCCTCTTCTCCCCTGCATCTTCAAACAAACACACACAACACTGTGTCTGT<br>GTAAAACTCACACCACCTTGTGTTCGGTTTCCAATTCAGTGCCATGGCAGCACCAACACCCC<br>CTTTGAAAGATGAGCTCGACATCGTGATCCCCACCATCAGGAACCTCGATTTCTTGGAGATGT<br>GGAGGCCATTCTTTCAGCCTTACCATCTCATAATTGTGCAAGATGGTGACCCTTCAAAGACCA<br>TTAAGGTCCCTGAAGGCTTTGACTATGAGCTCTACAACCGCAATGACATCAATAGGATTCTGG<br>GTCCCAAGGCCAGTTGCATCTCATTCAAGGACTCTGCATGCCGTTGCTTTGGCTACATGGTG<br>TCCAAGAAGAAGTACATCTACACCATTGATGATGACTGCTTTGTGAGTTTTTTCTCTCTCAA
Chr08	2,352,136	G	A	A|A	G|G	TA=missense_variant	TGN=Phvul.008G029200	CAGAATATGAGGCTGATAGGAGGCAGGATGAAAATAGAGATCATATTGAGTGGACAACTG<br>TGAAAATGCAATTAAAATCCTATAACTTACTCAGTGCTTGGGTCGTTCTCCTTTTGTCTGGA<br>GTAATGAACTTAGTGGGTCTGACAATCCTAGCTTTTCCACAAGTATAACCTGGTCCAGGTG<br>TTTTCAAGGTGAAGTTTGTTGGGAGTTTGACAGTTCTCTTTGAGTTTCCAGCATCACCAAC<br>ACTGACTTGAAAAGATGCAGCTGCTCTGCTTGGATCCTGCACCCATGAACTGAGCACCCC<br>ACCTTTGCAACAATTTGCAATTTGTTGATTGTAAGGTGTCCCAGGAAGCAAATCTACCACT<br>GTTGGATTTTTCTTACAAGAATGTGGAATATTTCCCTTAAATTTTGAGCAGTCTCCTTGTTC<br>AGTGGTCTGCCCTCCCATCATGCTCCATATTACCTCCCTTTTTGCCCATGTCCATCCTAGT<br>GACCAGCCAGGT	TGTAATGTGACGATATCTTTGGAAGTTGTTCATTGTAACAACAGCCTGTACATTCAATTCTTTC<br>TTAGATTTCTGAATAACTAACCCGACAAAACTAAGCCAAAACAAATCATTTACAAAAGATGCAA<br>CCAAGCAGTGGAAAGAAAAGCAAAAAAAGACATTTCAACAGCATGCATAATGTACAACATATG<br>TTCTCATATTAATGGGTGATGAATGTACTTACTACATATCCATCAGGGGTCCAGGATAAAACAT<br>CCCATTTGATTGTGATATTCCCATTTGGGTCAAGCGGATCATAAGCATCTGAAAGATAAACAA<br>CCAACATCAAGTGGAAAGTGATTAAAAGGAAAAGAAAGTAAGCAAATAAATGGTGAACCTCTA<br>AATATTCCAATCATTTACTTGCTTATGTAGTCATAAAAAAGGAAAGCAAGAAGCAAGCACACAT<br>TTTCTTTATGGGTGAAGATATAGTTCTTCATCTTTGCATATTTATTTCTTGTAGA
Chr08	2,412,539	T	G	G/G	T/T	TA=missense_variant	TGN=Phvul.008G030000	AAAACGTGTTGCACAATAGTTCATATCAGACTTCTTCCCTATCCCTTCATTCATCTGTCTTA<br>TATGAAATGGTCACTTACGACGGCGTTTAGAAATCATTTTAATCCTTTTTGTTTGTTTCTTC<br>CACTGTCAACCCCACTCTGCGCTGTATATGTACCTGATTTAGCTTGAAAAACAAACTTTGG<br>ATACAAAACACTACATTAAAAAATAGATGTAATATTATGAGAAAGAAATGTGTAGGGCCAAA<br>ACATGGAAGCTCTGAGAAACTCAAAGGAAATAGGCAATGAAAGAAGACGGAGTAGCCAAA<br>TGTGAATTATATATTAGAAACTAGAGACAATCATAGGAGCAAAGAAATTAGGAACAAGAAT<br>GTTTCTCAAATATTGGGTCTTCTGCCATTCTAAGCGCACAAGTTCACGTCAGTATACAACA<br>AGAATAGAAGAGTTGTGCCATCACTTCTCTCTTGCCGATATTAGGAAATCAACCAACAACT<br>TTGATCATAA	GGCTCATTAACTTTGAAGGATTTGGTATCGAAGTATATAAAGGCTGTCTTCAGCATGATGGTT<br>CTGATTATACAGTCACTATAAAGCGATTTAATGACGATGTGGATAAAAGAGAGAAGCAATGGA<br>ACCTGTGTAAGTTTAAGAATGAAATTGAGTTGCTCTGCCAGCTTCGTCACCCTAATATTATCTC<br>TCTTACTGGATTCTGCAACCACAAGAAAGAGAAGATTGTTGTGTTGGAGTACATGCCCAATGG<br>ATCTCTGTATGATCACCTACAACGTGGGGAACTGTCATGGAAGAAGAGGCTAGAGATATGCA<br>TAGGAGCAGCAAGGGGATTGCACTACCTTCATGCTGGAGCAAAGCGCACCATCATTCATCGT<br>GATATCAAACCTCAGAACATTCTTTTGGATGCAAACATGGAGCCAAAGCTTTCAAATTTTGGG<br>CTTAGCTTACAGGGACAACGTTTTATGTCAAAGCCAAAGCCAATTAAAGTAGATACTATT
Chr08	2,421,572	A	G	G/G	A/A	TA=synonymous_variant	TGN=Phvul.008G030200	CTCTTCACGTCACGGTGAATGATCATTTGTTTCGCACCTGTATGCAGATAATGCAGTCCTC<br>GTGCGGCGCCGATGCATATCTGAAGCCTTTGATTCCACGAGAGCGGAGGGTTATCGGTT<br>CCGTAGAGATGATCACGGAGGGTTCCACGATCCATTAAGTCGTAAACGAGTATCATCTCG<br>TTGCTCTCGTAGCAGTAACCGATGAGGGAAACAAGATGGACATGACGAAGTTTAGAGAGC<br>ATTTCGATCTCGTTCATGAACTCGTTCAGACCTTGCTGAGAACCCGGTTTGAGCCTTTTGA<br>TAGCGACAGGTGTTGAGCCGTCGTCGATGTAGCCTTTGTACACGTCGCCGAAGCCTCCC<br>GCTCCAATGACGAAGAGTTTGTCGAAGTTATTTGTCGCGGCCCTGATTTCTGCGATTGAG<br>AAATGACGGCAGAGGTTGGTTGGCAGCGATGATGATGAGCCTCCCCCGCGAGAAGTTCC<br>ATCTTTTTGGTTTTTGTAAAC	CAACGTTCTGCTTGCATTTGGTGATGAAAAAAACGACTATAAAGGAGAGTAAAACGACACCAA<br>AAACTGCACCTGCGATGGCTGCAATTAGGGGTCTGGTGGTGCCGCTACTTTTCTGCTCTGGT<br>ATTTGCTTATCGAGGGTTTTTGGAAGAGGATCTGGGTTGGGTTCAGCGAGACTACCTGTTGT<br>GTCGTTGATTTTGAAGAGTTCAATTGCATTAAGTTGTGCATCCTGGATGAAGCTTTTAGGATG<br>AGGATGCATTTCCAGTGAAAGATATACCTTTTCCCTATTCCCTGGAATGTAAACTGCGTAGTC<br>TTTAACCACTGGTACACCCTTCTGGTTGTCGCTCCACATGAGAATGTCTGCACCTCCTTCAGC<br>CATCAGTTTCTGAATGAAGATGTAGAATATTTGGTTTCCAGGGTCTTGAACCTCCGGGTTGAG<br>CTGACAAAAGTGTAACCTTAGCAAGTAGAAGAAGCCAGAATAAACCGGAAACTGCCATGTC

Chr08	2,432,797	T	C	C|C	T|T	TA=intergenic_variant		AAAGATGTATTTTTTAATTATAAAAACTAAAATAAAAATTGTATTTTGGAATATATAATTCAAAATATAAAATAGATATTCTGAAATTTATACTGAAAATAACATAATATATATTCTAAAATACATTTTTCTATTACAGAATGTATTTTAAAATGTATATTTGGAATAAATATTTATCTTCTGAAATAGAAATATAATGTAAATATGTATTTTGAAATGTATAACTTAAATTTCAAAATACATATTTTGTATTTTGAATTATTTTTTTACATTAATACATAAATTTAACTTTTTAATAAACAACAACAATCACGCCACCAAACATCACACATCCACTAAGCACCCACAGCCATCACTAACACAATCTTCACATTATATCCAAACACTCCAACAAAAACACCCTAATTTCCCACAACTGACAATTTATTTTGAAAAGAATAAACTTGGAATTTCCAACTTTTTAGGAGTGCAGCTTCAAAATATGGAGGTGCAAAATACAA	GCAAAAGAGGGCCAATATCAGCCCGGCCCATACAACATAAAATAGACCGCAAAATCAGCCCATAATTAACCCAACTATGGGTAAATGAACAAAGCTAACATTTAAAGGTAGAATGAGTAGAGATGAGAGCTGTTTACACCGAGGTAAATGAGTAAACTAATAATGAAACAGTTAAAAAAGAATATCTCCCTTTATAACAATGAAACACAGAAGTAGGAATTTTGCCTTGAAGGAGGAAACCTCCTTAACCACTCCAACCTTATTAGGATTTCTTATTGAGGTTTTTGCTTTCTTTAAATTTTAAACTTGTAGAGTTAGGGTTATGATTGGAAAGTACTATTAACACATCTGTCTTTTTATCCCTCTTGTAGTAAGATAAATAGTTAAACATTTTAATAATTTTTTAATTTTTTTATCTATTTTAATAATTAATTTATAAAATTATTCTAGTAAAAAATCGACTGAGACACCTATACAGAAAATTGTGATTAAAATATGAT
Chr08	2,488,852	A	G	G/G	A/A	TA=upstream_transcript_variant	TGN=Phvul.008G031000	AAGAACGTGATGTTACGTATATTAGATGTAGTTAACTTACTTAAGATAGACAACCGATTAATTTTTTCACCGCTATTTTACAACCTATTTTATTAATTTTATAATCAATAATTTAGTTTATAATTGGATTAAATGACTTAATTTAATATTTTAATATTATTTATGATTGTTAAAAGGTTGCACATTGGAAGAAAATGAAATGTCATAATAACTTTTTTCTTATACTAAAGACTAAAAAATTATGTAAGATAACAAAATTAAGAAGAGAGAATATATAGAAAATGTGGTTATTTTGAATGGAGAAAAAATACTGAAAAAAGAAAAGTAAAAATTTGTCAATAATTTAATTATATACTAAAATTATATATATATGATTTGATAATTTAAATAATTATCAAAAGTGTTATTTTCATTAAACATTAAATATGAGTAAATATAAATTAATATTTAAATACTTATCTTGCTAAAAAATTATTTCACTATCTACTTAAAAAATAATC	AAATTATTTTATATCCATTATAATCTTTAAAGTTTCACCTTATTAATCCATATAGTTATAGTGATATCACTTTACTATTAAAACAATAACGAATTTTAAAAAAATATAATATTTAAAAAAATTAGTGATCATTCTACATAAAAAAGATTAATTTCAATTATTTTTATATTATAAATATATAGATGGTTTAAAGTTTTTTAAAAAATAAATAATTTTATATTAAAAAAACAGTAAGAAACACATGTTTAATCTTATTATATTTATATTTTTAGCTTTAGCAACACACATACAAAACAATCCATTGGCATTTGAGTGGTCATATATCAATGAATGTTGGAGAATGTTTATCTCATTGCTCATTATGTGAACGACGTTAACATATAATATTGTTAATAACAATGCAAAAAAAAAAAATACTGTGATGAAAAAATGGGAATGGCAAGAGAGAAATTGGACAAATGTTTGGATCTTAGCATCTTTCCACCATTTTTTCTTCAAAA
Chr08	2,492,841	G	A	A/A	G/G	TA=intron_variant	TGN=Phvul.008G031200 *	TCTGGGCATATTTTGCAAGGAATAGAATAGAATACGGTGGCAGCTGAGATTGCTTAGTGTAAGTCAATAAGACAATGACTAAGGTCTAAAGCTTTAGCAATTTATAAAAAAAAAATTCTATATAAGATACTTTGACATGAGACATTCATTCCCTTCCTCATTTAGCCTGTCTTTTTCCTTTTCTATTATATTACAACCTTTACTTCTTTGACCACAAAACAAAAGGGTATCCTCTGTTTTCTTTTGTTTCTCATACTGAGTCCAAGTGAGCAAATTCATGCTATCAAGCAAATGTTTTAAGGAAAAGAAAAGTCAATTATCATAGAACTTTGTCATTGCTTTTTCCCTTGAAGAGTTACAATCAGCAAGAAGGAACTTCAAGCAAAGTTGAGTAATTGGATTAAACATCTATAAGAAAGTGGTAATATCATCCCGGTTGGTGGATGCTGTCTAATGGTGGCATAAACATTCTTTTTATGGTTTTTCCCAGAACAGCATTAG	TGGTGCTTTCACAACTCCACCGCCCCAATCTTGTGCCTTTGATTGGATTTCGCAATGAAGGAAACAAGTTGGTTCTCGATTATGAGTACATGACTGCTGGTTCCCTTCATGATTACATTTATCACTAAAGTACTAACAAAGTTGGTTCACTTCTGTGTAAGAGAGGCTTCACATTTACATATACATGGGACCCGGATTGCACTATTTTCTTCACACAAGAGTCAAGTATGTTCATGACATTAAAACCCAAGACATTCTTTTATTGGACAAAATTTTCTAACCTCCGGTAGTATATATTTCTTCTCTTAAAATAACAACACTCATTTTTCTATGATTATTTTTTTTCCTAATTTAAATTTTAATGAAAAAAGGAAAAAAAAAATGTTACTGAGTGAAGAAAGTTATATGAAATGAAGTATGTAATACATTCTGAAAACATAGAAATCAGTGTTTACATTTACAAGTTTGTGATAATAAAACAACCAAGCTTTTTCACTAAA
Chr08	2,494,274	G	A	A/A	G/G	TA=missense_variant	TGN=Phvul.008G031200 *	CTTCAACGCGGTATTTAAACTTTTCAACTTACATCCTTAGTTCAAAAATACAATAGATGGAACATGCTGGACTTTGTGGTAATCCTCTCCAGCAGTTCCAAGCCTGGTCTTGAATGATTTAGGCATCCACCTGATTTCTAGCTCCCGAAGACTAGTAACATATTTTAGCCCATCAGGAATCGTCTTTAAATTGTTGCAATCAGATATGCTCAATCGGTAAAGACTAGGCATGGCTTCATTCTCTATTGTCCACTCATCTAGATTAGGCAAGCCACGAAGTATAATAACCTTTAGCTGAGGAAAACCATTTCGTGTGCATGCCATTTTCTTTCCAACAAACATATCCCACCCATTTAGAAACTTCAAGTTAGGAAGCTTCTCTAATGTTACCATTGGGTCTTCCACAAGTCTGCAACCCCATAGAGTCAACTTTGAAAGCTGTGGAGGAAACAGTGAGGCATCTGGCAGCCTTTCCATCCGCCCTTCAACCTGCAGTTTAC	AGGGAAGGACAGCCGAGCACCAATTTTTCAACATCTACTACATTTTCAGGGAAGGAAAGCATGTCGGTCCTTAAAGACAAGGATTGAAGGCAGTCCAACCTCTTATTAGGGGGACTGAAACTTTCACTAAACTTTTGAAAATGTCTTGGGTCATTCAGTACTAACTTTCTGAGCTTTTTTAACTTCAACAGATCTTTTACATCACATTTGCTGGCAGGAAAGCTGACTAGTGTCTGTAAATTAGTCAAATTTTCCAATTGCAGGTTGTTAGCAATATTCCCACACCAATTAGGAAGATATAAATGCCTCAACCGTTTCAACTTACAAATTACATTTGGAATTTCCACTGTTGAATCCCAGCTCACTTTATTGACTGTTTGCAAATTTAGGAATTGCAGATTCTCCAAATTGCCCAAGGAAGATGGCAGTACTTGTATACGAGTCCTTTTTAGACTCAGAAACTTCAACCATAAAAGATTTCCCACTTCTTTAGGCAATGA

CHROM	POS	REF	ALT	BRS Sublime	CNFC 16207	Functional annotation	Gene	Flanking (-)500bp	Flanking +500bp
Chr08	62,080,599	T	C	T/T	C/C	TA=intergenic_variant		AAAGATTTTCAGATGATCAAAATGATTAGTGGTTCACTATTACTATTATAAGAAAATTGCAGATATCAAAACATTTATTTTCTTGAATCATGGTAGCAGTATTAAGAAGAGAATACACTTGTGAGAGAGAGAGAAACCAACCAAAACCCCATCATGTGGACCTGTCTTCCTGTTTTGGTGTTTGAAATTAATGTGAGGGACCATGGAGCGTGTCTCTGTGATGTATGCTCTGAAGGGTTTGGTTGTTGTTCTCACTTGTGACTTCTGAGCAGAGCAGAGAGGATAATAGTTTCCATGACACGTTGCAGACATGCACAACAATTGCTAATGCTCATCTCTGTCATAGCCTCTGCATGGCATAGCACAGTTCAGAGAAACCAAATGTACTCACGCAGTCACAGCTTTCTCCTTATCTTCATTTTCCATTAAACAACTCATCACAACCATTCATATCATTTCTCCAAACAAGGTGTTAACATGTTATTCACCCTACAAAACTT	TTTTCTTCTAAAAAATTACCATTTTATTAATATATTTAGAAACTGTACATGATTGTTATTGTTAAGTTGCATAATGATATTGAAAACTTTATAAAAAATATATATAAATATCAGTTTGGGATCTTGAAATCTGCATTACTGTAAAAGCTAAAAAAGGGGAAGACAAAAACGAAAGTAAGGACTCTGCCAAACGATACCCCGTGGGTGAGAAAAGCACGTTCTGCCCAAGTAAAGTGTTGGGTTACCAAGGAATCAAGAAAAACAAGAATAAGGGGAAAGAAAACACTTCACTGATAAAAGAATATCTATATAAAAAGCTATTTTTAAAAAAGATCACAAAACCTCGTTCAAAACAAGAAACAACGTTACAATTATAACAAATACGAACTTCACTGCTTAACTCCTTTCAACACTACTAGACGGTCTTTACTACACATTTCATCTCCTTTTTCACATAAGATAGACGGTCTAATAATAAACTCTTTTACAACATATCATCA
Chr08	62,083,555	G	A	G/G	A/A	TA=intron_variant	TGN=Phvul.008G279400	TATACTGAATAATTTGTGATAACAATGACAATGAGGAAACATAATCTAATACAATCACCTTGGATAGAACACCAAGTCCAGAACATGGAGCGTCCAACAAAACTTTGTCACTCTTGAGTTGTCCATTATCCTGGGAAATCCAAAAGAAAACTAATAAGAAATAATTAAATAACAATTTCTGAGTTAACACTCATAGGGACAACATAACATACAGTCAATGTACGAAGATCAGCATGAATGGCGGTAATGACACCATCCACTTGGTGCAACTTTGCTGTCTCTTTAAGAATTCTCAACCTACCACTATTTACATCAACTGCAAATACCTTACCTGAAAACACAAGGTTTCCTTTTATTCTGCTGAATGGAAAACTAAAAAAGCAGGGAACTAGTATATCAACATTGTAGAGACAAACAAAGTCTCGTAGCCAAAATGAATGAAGATCCTCGGACGAAAAACAAAGTCGAGAAATGATGTATTAGAGTGTCTGAAGAAATTG	ATACAATATGATTTAATAAAGTATCTTAGACGAATTAGTACCTTGTCCACTTAAAAGAGATGCCATGTAGAGGGTCTTTCCACCAGGAGCAGCACAACCATCAACGATAGTTTCACCAGGTTGAGGATCCACAACAGAAACTACTAGACCTGCACTTTCATCCTGAACTGAACATAAACCCTTCTTGAGGAGACCAGCATGAATGACAATCTGAAATATGAGCCTACAAATAGTCAACTTAAACGAGAAAATCATGCTTACTTCATAACAGATAATAACAGCAAAAAAGAACATAACTTCAATGCCTCCCATGTGAAAAATATAAAAGAAATGAAAGTAAAAGATAACCCACAACAAACAAGTAGTCAGCTAAAGCACAATTACACACTATTGAACCTATTAGCTATTATTACATAAGGTAGAAAATATGTATATAATTCAATCCCAAACGGCTTTTCTCCTCTAGTCAGTGTTCATTATACTTGTTAGAAAATTCATAC
Chr08	62,085,019	G	T	G/G	T/T	TA=intron_variant	TGN=Phvul.008G279400	TATTCACTAGCCTTAAGCTGAAACTGGGCTCACTGTTATTCCATATCATCAATTTAATGGCTTCTTCCTGGCCGAGATACTTTGTCCATCTCCTTACCATCCACTGCAAAAGAAGAAATAAGAAAAAATTTCAATTAAATTTTACTTATCTTATTTTGGTGATAGCAAGTTTAGTAAATTGCATAATTCCTTCTCTTAACCTTTACATTGTTAGTTTGATTAAAAAAATACTAAATTATTCATTATATAGTGGTTCTCTGCCTGCCCTTGTAGGATCTCAAACATGCCATTGGCACCTTGGGTAGTAGTATAGGAGTAATTTATCCCTACTATAGCTTTGATTCATAGAAAAAGGAAACTAAAATTGTTATGAGCCAAAAAAAGGTAAAAGGAATTGTTCATTGTTAAATGAATTCAATCAATAAATTTTTCTCCTCATTTCCATTTTGGGTTTGTAACATTAATCAAAGTGAGAAACATTAGATTTTATTATTGCACTG	TCTGAATTCTGAAGTACAAAATCCACCTCCAAAATAAATAAAAAAATTCAGGCTTTCAACGTTATCCTGTTAATTATGTTTTGATAACAACCACAAAATATTAAGAAAACACCAATAGCATAGCATTTTAGGTATGCTAGAGCTTGGTAGCAAATGAGTACAAATTAAGTATTGATCATATAGTTCTAATTCTTCTTCGTTGAATTTTTTGTTTACAGCCATTTTGAATATCTTAAGCTTATATCATGCTCATACTTTAATGTTATTGCTGAGCACAAAATAAAAGTTTTTATGCTTATCGGCCTCTGATGCAGTGATTCCTAATTCTGAATTGGAATAATGAATAGTGGTATAGAAAAAGTGAAAGCAGTAAGTCACTCACAACTGGATGTGAGTGCAGAGTTGCCAAAGCACGTGCTTGAGCACGGTCATCACCCTCCACTTTGGGCAAAGGAAGAGTACCGTTCTCCTGCAAACCAAACATCAATGGATTTGATAAA
Chr08	62,096,576	C	T	T|T	C|C	TA=intron_variant	TGN=Phvul.008G279500	TCCGTTTAACGCGTTACATCTATAAACCAAATAACGCTCCCTCTCATTCGAAGACACCATCTTTCATTCTAGATTTTCTCCTCTTTTTCCTCGTTCCTTTCTTTTCTCCGGAAAATCGATCATCATCGTATTCATGTCGGTATGTGTTATTTTCCTTTCAATGTCCATCAATTGCATTTCATCATCTGTTCCTTCTGATTATAATTGTCTGAATTTTAGGATGAACACAATTTGAATGAGAATTTCTCTATAATTGTCTGAGTTTTTGTTTGTGCAGGACGAAGGTCGACCATTACCAAAGTTTGGTGAATGGGACGTTAACGATCCTTCTTCTGCTGAAAGCTACACAGTCATTTTCAACAAAGCAAGAGATGATAAAAAGACAGGTGGCAAACCAGATTCACCTTCTAAGAGAAATCATCGTACCAGACATCAGTTAGATCCTGCTAGAACACAACAACAAGTAAGATTTTCTTCTTCTATGTTTAAACAAACCTATT	AAAACTGAGAAAAAATTACTTTTTCCACACAAAAAAACTGAAAAACAACACAAAAGTTGAAATCACTATTAATATTCACCCGACATAAATGTGGGGACATGTATTTTTTTAATTAGTTTAAAAATCTTGATCATTATAAAAAGTTAAAACAAATATTTTTATAATATGTTTAGGAAATTTCTTCCTGCACCCTTACATTTATTAGAATACCGAAATTACCTTTCACAATTTTAGGTGATTCTGGAATAAGGATTCTATAAGCAAAAGCAAAGAGTGTTTCCATAATAAGAAATCCGGATAACAATAAATCATTCCAAAATATCCTTTTCTAGAACATAATTTTTTATTTATTTCTGGATTGATAAATCTGTAATTAATAAAATATGTTATGAAAATAGAAACAGAAAAATATTTTTAAAAGGAGTAAAACAATCTCTTTTTTTTTTAAATAATGGGTTGCAGGAAGACATTTGTTAGGATGGAGGAAGAAACAACCATATG
Chr08	62,099,963	C	T	C/C	T/T	TA=intron_variant	TGN=Phvul.008G279600	ATACAATTAGTAAATTAAAAATATAAGTTCGAAGTATCAAACTATTTTTGAAGAAATAGACTACTCAACTTAAGATTTATTTAATGTATTTAATTAGATTTAAGGATGCACACAATTGCTAATATGGGACGAAATTCCATGCCAAAGTAGGAATTCAAATTTTAAATTAACATAAGAGAAAAAATTGATGAAAATAATTATTTGAGAAAAAATTAATAAATTAAAATAAAAACTAGCCTATTAAAATCCAATTAAACGAAAAAGGCCTCTAAAACCCCACTAATTATATATCAACTTGACTGGAGAGATTTGGTTTATCATTGAGTATAAAATCAAACAATAAAAGAAGACAACTTTCTATGATTCAACTTTTACCTAATTATAAATTTTAAGTAATTTACAATATGAATTTGTTGATCAGGAGTTTGAGAGAGAGAAAAATGTTGCAAAAGTTCTGATTGTTTTGTGAGAAAAATGATAGTTTGACAATCCTGTACAACT	TAATGTGATGAGTTTAGTAATAAATATGTGTAATAAAGAATACATTTGTAATTAAATGATATGAAAAATATTTAAATAATAATATTGAGAGTTGTAATGGTTGTATACAAAATCGGAGTTGCGAAAGTGATATTACCTTTGGAAAGAAGGAAACGCCCTTAAGAAGATAAAGTGAAGGAATGATGGAGTAATATAGTACTGACAAGGAAGTGACTGCAAAGAAGTTGTAGTGCCAATATCCCAATAATAAGCCTAAGTTGATCTTTTTATCACCATAGCATTTAGGTCTAAACTCAGAAAGCAAAAGTTGCAATTGACCTTCACCCCATCTCTTCATTTGAACTAGTGTGCGAAGTAAGTTAGTTGGAGCAATACCAGAAAAAGCATTTATTGATGGATTGTAGTATACTGATTTCCATCCCTGTGACTTTATCTGCAACCCTGTTATCACATCCTCCACAAGACATCCATATATTGAACCAATCTACAATAAAATAAAT

Chr08	62,102,713	T	C	T/T	C/C	TA=synonymous_variant	TGN=Phvul.008G279600	AGAGATACACGCTCAACTTCTCAGCTGGATAGTCATAAGCCATCACAGATAACACTGTATTGATCACCATCATCGGTGGCTCCACCACTGCATCTGCCGTGAACACAAAGATGTCCACTTCCGGGAACCTACTCTCATATCTGCCAAATTGAAACACACACATAATCACAAATTAGTACACTACAAAAAAATCAGATTTATAGACAGATTTTTATCCACAGAGTCCATAGAAACATCTACGTAAAGGTGACAAAGATGAACCTTGGAGAGAGTCTGTTGGGGAAGGTTGTTCTGTAGACCATGTTCCATCGAAGGGCATGATAAAAAACCCAATAGATGCCACCCCACACTTCTGCAACAAACAAACCAAGCCAAGCCCATTGGCCAGCTTCTCCTTTTGCAGGTATGTGAGTGAGTCTGAATGCCCAGACAAAGCATATGGCAACAAACAAAGAGACAGCATAGAGCCTCCGAATGTGCCTTGCTTTGTCTTTCCTTGT	CAAACAAAGAGTTCTCTTCACCACTCCCCATTCTGCCTTCTAACTCACTCCTCTCTCTTCTTGCTCTCTGCATCCTCAGTTCACTTTCTTATATAGCTACAAAAAATGATTATGATATTCCACGAGTTTCAATATAATATTCTGTTAACTTGAATGTTTTTTGAAAATATAAAATTGGCTCTGATCGTTCGTTAGCAAGGCATGAGTAGATAGGGAAGTGATAATTTTGGAATGGTAGGTAACGATCCATGAACATCACACGCCATTTTGGAAGGGTAACTGGTCCATGAGCTACTCATGTCATTTTGGAATCATAGATAACCCAGAAATTTTATACTATTATTTTAATATTTTTTCATATCATTTAATTATAAATTTATCCTTTATTATATATATTTATTTCTAAATTCATATATAACTCTTAATTTTCAAACCATCATTTTCCATCCACAAATTACCACTAGTATATGCTACACTGACTTTACCACCAAGACTTCAAA
Chr08	62,115,551	T	C	C/C	T/T	TA=missense_variant	TGN=Phvul.008G279700	AATATAGTACTGACAATGAAGTGATGGCAAAGAAGTTGTAGTGTCCATATCCCAATAGTAAGCCTAGGTTGATCTTTTCATCACCATACCATTTAGGTCTGAACTCAGAAAGTAAAAGTTGCACTTGACCTTCACCCCATCTCTTCATTTGAACTAGTGTGTGAAGTAAGTTCGTTGGAGCAATGCCAGAAAAAGCACTTATTGATGGGTTGTAGTATACTGATTTCCATCCATGTGACTGTATTTGCAACCCTGTTATCACATCCTCCACAAGACATCCATATACTGAACCAATCTACAAAAAAATAATCATTTATTGTACTTTAATAAATGCTCAATATTATTGACTATGATTATGAATGTGACTATGAAACTGTAAAAGAGATTAGTAAACCACCTTTTTCCCCCATAAGGTATTTTTCTCATAACTGCAATTTGCTAAAGCCTTTGATTCTTCTTCTAGTTCATGCAAGTTTACTTGAGTCACCTCATTCTCATTC	ATCATTCCAATCATTTTTGTATTCACGACTAAACTTCTTCCTATAGAAAGCATCTCTTCTAAGATAGCAACAAGTTCCAATAAAAATAGGGCCACCAAAACCATCAGCACCATGGAGTTCTACCTTCAAAGTGATCAATAGAGTGTGAAATTATTTTTAGATAAACATATCAATATTTATCATAATAGCAATGTTATCTATGTCTAAAGTAAGAAAATTACTACAAATATATTTTCACAAAAATTATGTACATTTAAGAAAGACACGTGTCATCTTAGAGATAAAAAAAATTAATATAGAAATATGGACAGGTAGATGAAAAAATCTATTACATCAATAATTTAACTAACTCACCTCCACAATTTCTGATAGAGAATTGCCGTAAAGATCATTTTTTCCAATGTTATCAAAAGATTGTGGAAATTGCACATAAGCAATTTCTTGGCCTTTATCTTCATCCATGAAAAAACATAGAGTATCTCTCACTGATTGTGAATGGG
Chr08	62,121,699	G	A	G/G	A/A	TA=missense_variant	TGN=Phvul.008G279750	CAACAAGTTATTACTTAATATGGTAAGAAACATGATACAATAATGATAATAATAATTAATTAAAATAACAAAAAGAGTTATGTAATTATATACCAATAATTATATTTTTAAATAATAATCCAAGTTTGAGTCTTGAAGTCTTATGTTTAGTCCTTTTGTATTGTTTTATAAATACTATCTCGATAATATAAATATATTTAGGAAGCTTTCTCTCTTGTATAATTAACCTCTAGCATAACTTAGAATGAAGGTTTAATTAATAACAAAAGTTATAATAATGTCATGTATTGAGAACATTTCTTTCCATTTCAAATTATTTGTATAACAATAAGTACATCTTTTTTCAATGGAAATACAAACAAATTCATATAAAATAATGCAAGCAAAATGTCTTTACCAATAGAGGATAAAACAAAAAAAAACTGCATTAATATAGTGAATTGAATAGTTTGAAGAACACACATGCAGTAAAAGCCAATGTAGTAGACTTGATAGAAACA	AGTTGGCATTCTACCCTTATCCTTCCTCACAAAAATGGCTTGGTATAAGGGAATATTGATGACAACTAAAACTCCACACAAAAGAACTTGCAATGCCATTTTCTCAACACCAAAGGAACCTTTGCCAAGGACCAAATCCTTTAAAGTTAGAAGCAAGCAAAACAAATTGAGTAATGCAAGTGTTGCTAGAACAGTGAAATAAGGAGAAGATGTTCCAAACTCCATGACCTCTTTCTTGTAACGTTGAGAAACATCATCATCAATGACCTTTGATGTGAGTAAAAATGATGAATTTGGACGTCCAAAGAATCCTAGGATGCCGTCTATTAAGGAAAAAAGAAAGGAACTTGTTTTCACGTACAACCACATTCTTGTACCATTCCACCAACCTTGGATTGATCCTCCCATACTCAAACCCTCAATCAAACTATAAGCAATTTCTCCAACAATTACACATGTGAAAGGTATACACCATAAGCTAGAAATCTGTTGAACCAATA
Chr08	62,164,249	T	C	T/T	C/C	TA=intergenic_variant		TTATGTTTAACATTTAAGAATTTGTTTAATATTTTATAGTTTCAAAGAAAGAAAGACTGTTATAACATTTTTATAATCTTAGTGATTAAAACAATATTATTTTTGTAATCTCGAATCTTTGAAATTATAATTCCTTAAAAAAAAACTAAATGAGGGATGAAAAAGTAATTATAGAAATGTTTAATATGAAGTAAAATAACAAAAAATAAGCACAATCTCTTCAAGAAGAGACTAAAAAAGTGTAAAACAAAAGTAACCAGAAAAGATTGAAAAGAAATTACTTCGAAAGCTATCACATTTTATTTCCCAAAAACGATTGATCACTTCATCATTCTCTGGTCTGTAATCACACGTAAGAAAGAGATGTAAAAAATATTATTTTAATGAGCATAATTTAGTATAAAATAAACCATCTATTTGGATAAATATAAAATTGTACAATTTTTTATATTAAAACAATTAATTTTTATTTTGTAAAGTTTGATATAATTTTTATATAA	AAAAGTTGATTTTACTTTTAAAGACCCTTAAAAATTGTTTCCTCTTTATTACCGTTTAAGGCACGGGGTATTTATCTAAATAATGAAAAAATAGTATTTTCTTTTATTACACTTTGTCCAACACAGACTGCAAGTGATAACTAATATGAATGATAAGTTGTTTCAGAGTAGAAAATAAGTTATCCTTGGGGATCGAGTATGTAAATTATCAAATTCGAGATATTTTATGGAGTTCTGTTATAATTTAAAAAACAAGGGAACATTTAAATTGGTTTTAAAATATTAGTAAGTTTACTCAAAGTTAAAGTATATGTGTTCTTGTAACTTCTCTCTCTGATAAGGTATTTTATTCAATAATTTTTTTTATTTCAAATGTTCTTTTGAGATGTTCGAGAGGATGAAGTACTTATAAAAATATTTCGACGTTCAGGTTACATGAGGTTAATTTAAGTGATCTAGTTCAAAATTCAAAATAACAATATTTTTCATCACAATAGTC
Chr08	62,165,168	A	G	A|A	G|G	TA=intergenic_variant		ATTTCGACGTTCAGGTTACATGAGGTTAATTTAAGTGATCTAGTTCAAAATTCAAAATAACAATATTTTTCATCACAATAGTCCTTTTTTATTTATACTATTCTAGTGGGTTTTATCTCGTTACAGGGATTAAGACAAATTCAATAAACATCTAATTACACTTTATAACCTCTAATCAAGATTAACACTCTTGACCTGTAATGTGACGTGGACACTACTTTTCACCGAGAGATTATTAGGTCATATGGGTAACCGACTACTCAGACTTCCGATCTTCACTTAGTACAAACTTTAATGACAAACACTTTAAAAAATTTCACTAGGACTTTATTGCCATGGAGAGTCTTGTGTTTTTCAGTTGTCTCCAACTTATTCAATCGGTACTGATTAACGACCAGACACATGACTCACACCAATTTTTTACCACACTGATTTTAACTAGACAGAGAATTTGTTAATGAGAAACACTTTAAAGAATTTCAGTAGGACTTTATCGTCAT	AAGAACCATGTGTTCTCCATTTGTCTCCAATTTACTACCAGATATCTAAGTTTCTTCGATCCCTGATATAACAATACGTTAGTTATCCTATCTCAAATATTCATAAAAAATCTTATATATAAATATTTAATAATTTTTTTACATAAAAAATTGAATTAAAAACTACTTGTGCAGTGCACTTAACTTTCAAATAATAGTTTAGATATTTCCTAATAAACCAAACAAATCCGTGGAAAGTTGACTCTTTGATGTGTTAACTCGGAAAGGAAAAAAATTTGTGGGACAAAGCAATTAATAACTTTAAAGTAATGTTTCCAATTATTATAAGGAATTGCTTTTGTAGGGCATTCTCCACATTTATGGCAGTTGACTAAATGAAATTAAAGTTTGTATCAAAAGTGTAAAAGGGTGCTGCCAAAATGCTGACTTTTTGTTCCATTTTAAGTTCAACATTTCTCATATCTTAGAAGTGTATGAATTTTTTTTTATATTTGTATAAA
Chr08	62,178,784	T	G	G/G	T/T	TA=intergenic_variant		ATAACAGAAAGCAATGAAACACATTTTGTATTATCTTATTGGCACAATGTGAAATAGAATGTTTCTTCAACCAATGTTTTTCGCAATCTTTAAATTTGAGTTATCATTATATTATATCGTATCTTATTTAATTCTAATACTATTTCACAGAAATCCACAAATTCATGATTTGGGACAAATAAAAACAATACACCTTATTTAATTTTTGATTTGGTCAAACACTTTATCTAGATTTTAATATTTGGACGTTGTTACGAGGAGATATCAGTATGCATAATATATTATTTTATTAAGGTGATAAGTTAGTAAACAATATTTTATAATCACAAGCATTTGATAAAGCTATTTTATGGGTTAATCAATAAATATTAAACTAGGTAATTTATTGGAGATCTCACGATTAAAAAAATCATATTTTATATTAAATTATACAAGTGGGTATAAATCTGAATTATTAACAACATGATTTAGTTAATCTTAATAATTTTTTAAAAATTATT	ATAGATAAAAAGAAGAAGAGGAAGTTTAAAATATTATAAAATTATTTTATAAAATTTTATATTTTTTGTAAAATAAGTGGAGGAATAATTGATGTGTATAAAAATGGAAGAAAAAGTAATGGAGTTTCAGAGGTTTGTTGGTATTCTCTGTTTTAGTTTTGATGAGAGATGAGTGCATGTTCAGTTATTGCTTTTAACTTATTCTTAAAATATGTCACTGTCAATAATTGTTTCTATTGCATCTTCTGTGAGGATCCAATATGCAATACCCTCATATTTATTATATTTCATTCAAAAAATTAATCTCACTTATATCTTTTTTTTCTTGATTAATCTTAGCTTCACATTTTTTTCTCAAAACCATGTTGAGATTTTTATAATTAATAATTATATCTTTTGTGTGTGTTTGAAATTTTGTGTTTCGTTGTAGTTTTAACAATTTTTTCAGATCTATGATTAGAGGAATGATGTTTGGTCATTGTAATATTTTTTTCATTTAA
Chr08	62,183,317	G	A	A/A	G/G	TA=synonymous_variant	TGN=Phvul.008G280600	AAATCTTTTATCTGTTTATCCAGTAAATTGAAGTGTATTCATTTTCTTGATTTTTGTGAGCTGCTGGGCAGAAGTTCTCAATGTGTCATGAGTTGCCTTTCTGTCATCTTATGAGCACTGTGATTGGCAAACTTCATGTTGAGATTCTTATAGGTCGGTTGCTATGGGTTTGAATGTCTGAGTAGGTCTGGTTACTTTGGGTTTGTGTTTCCTGCCTGTTATGATGTGTGGTATCATGCAATAATATTATTTGCTGAGTAAAATGACTTGGGTCTTGTTTTGCAGTTGCTTATGAAAATGTTTCTTTTTATCTATGTGTTCTGTAATTCTTTATATCGTGTTATAAGTAAACGTGTATTGCCCCATGTTTCTCTCCTTAATGATTTTTATGGGCCTGAGTGATTTGAGTTAAATTTATTGTTTCAGGGATTGAGCAGCAGGGTTTCTGATGAGCCTAGGATAAAGCAAGGTGGACAAGGGAGGTGGGGAAGGGGTTCTTC	GAGGATATTCCCCTAACTTTCCTGGTACAATCTCCCCACCTTTTTAGTGACTTTGGGCTTTATCATTAGTTTTGTTTTGTCTCTTCAATAATGGATATTCATTTCCACATATGGTCACCTGGAAGCTATTGTTCTCCAAAGGAAAATTCTTCTTGAAGACAGTCATTTTTTCGAGATATTAGTACTATGTGGTGTTTCCCTCTCAGTAACTTGTTACTGAGACATTTTTATCATAATATTAAGAATTATGCTTTTATTTTGCTGCTTGTTAAGCTCTATCACGCCCCTTTTGAATCTAGCACGGCACTATAAGTATCTTTCAATAGCCATCATTTATATTTTTTCCATGACTTTTTATTTTTTAGAACAATGAACCCACTTTAGTGCAGAGACCTGTCATATGAATCTGCAAATTTCTTGCTACTTTATTAGTTTGTCAGTTTCACCATCTTGCAAATGTCTGTTGTTAGATGGTGGTGGTGGGAGGAACTTAGCTAATC
Chr08	62,185,847	G	T	T/T	G/G	TA=intron_variant	TGN=Phvul.008G280600	AAAAAATCAAGAACTGTGTATTTTTTATTTTTATAATTTTTCTCAGTACTTTTCAATATATCTCTAATTATATCAACTTATAATGTCTTTTGGTGGGCTCACACATGTTTCTGAAACAGCTAATATCACGGAGGTTTCAACTTCAGAAGTTTCTGTGCAATCTTCAGCAGAGTTAAGGCAACATGTTACATTTCCGAACCATTTCCAAGTACCAGAGGCTTTAAAAAGTGGTCTGACATTTGGAAGCTTTGATACTTTTGGCCCAACTGAAAAATCTAACAGTGGAACTGATGGGGACAATAACGTTTCTTGTGCTCTTGAACCTTCACCAGTGAGTGATGAAGCGGCAACTTCAAGGTCTGTAATTCATATGCTTCATATGTGTTTTTTCTTTGCATATCAAATCACAGATCCCTTCGTCCTGTGTGTGCATACACATGCTTATGTTTTCTTTCATACCCTTCTAAATGATGTATATGGTTGGATTATCTTAGCTTTGT	TTTAATATCAAACTTCTTCACCAGCAGAATAACCGTAACCTGCCTGAGAGTTCGTTCTAGAAAGTTTTTCTCTGGAGATAATATTAATACCTTTTTCAAATCCCTTTGGCAATCATAAGGCTGGGTAATTATTTCGTGAATAATTTTGGCTGAATTGACTGATTATTCTTCCTGTTACCAAAGGTATTATTTGTGTCATGGTGTGTTCATGTATTTTTTTTTTAAATGGTATTTCTTGTCTGGATGTAGCAACCAAAATGCATCATTAACTTCACAAGGGGATCATCTTGATTATCCACATTCTTCAACTTATTTGATTGAAAAGACACCAGATACAGAAGGCAATTCTATAAATGGTACTGACACAAAGGTTGATCAGCCAAAGAAGGAGGTTCTGTTGGCCCCTGAGGTCCATCAACTTCCAACTGTTCCAACACCTCAAAACTATGGTTTGAATTTCATGTCCACCATGTTAGGGACTCAGCAGGTTCAATTTGAGG
Chr08	62,188,506	C	T	T/T	C/C	TA=intron_variant	TGN=Phvul.008G280600	GCCACTATTACCCTCCAATTTATGTGTCTCCCATACACCAATTTTTAGGCCACAATGGTTTCCCTCAACAGCCATCAGCTGGCAACATGTATCTACCAGCAGCAGCTGGCATCAAATTCCCACTCCCTCAATACAAGGCAGGAACAAACACAGGAAATGCAGCTCATATTGGAATTCCTTCTGGGTCATTTATCACTCCACCTGTAGGTTATGCGCCTGGTCAAACAGTTAGTACCGGAAGCTCCACTGGAAATGAAGATCTTTCTGTGTCGCAGTTAAAGGAAAATCAGATTTACACAACAGGACAGCTGGTTGGTTTATTTCCTTATTAATGATCTTTTATTTCACCCATGCTTATTTCTTGATAAATCAGGAATTTTTGTTTCATTTTCTATTCCCTTCCTTTCTACAGTATCAATCTTATTTTGTTAGGTTGGTTTCATCGATGTATGTCCTTTTTATTGTGCTCCCATGAAACAAAATTCTTGGATTTCTGTAAT	CTAAACTTGTATGGTTTTATACTTTTTTGTTGCAGAGTGAAGGCTCTGCAGTGTGGATACCTGCTCCAGGCCAAGACATATCCAGTTTGCAAGTTAATTCTCTGTATAACCTTACCCCTCAGGGGCAACATCTCACCTTTCCCCCATCACAGGCTGCTCATGGAGCATTTGCTGGTATCTATCAAGCAGGGCAGACTGTAGCTTCACCTTCAACACTTTTGCAACAGTCTCAAGCTGTGGCTGGGCCTGTTGAAACTGTAGGGCCCCCTTCAGGATCTTACCAGCAACCTCAGCCTGCGCAGATCAATTGGAACTCTAATTTTTAGGCTGGAAAACATTTTCTTGGTGCCATAATGTTGATGTTCACACATGATGTGCTCAGATCCAGGGAAGTATGAATGAACACTGGCCAGATCTGAAGTGTAAATAAAAATGCGGCCAAAGAATGAGAAAGGAAATTAGTAACTGTTTTTGGTGGAGATTTTGTACTAACGATTTTG
Chr08	62,194,814	G	T	T/T	G/G	TA=5_prime_UTR_variant	TGN=Phvul.008G280700	AAACTTATAGTCTTCCACCGCCAAAACCGGAGTATCCAATGCTTTTTAAGCGATAAAATATCATTTCACACAAAAAGGGCTGAGGAAAATAAGATCATGCAAGATGAACATAGTTGAGATCTATCCATTACATAACAAATTTCAGAAAACCAATCCCAGAAAAAACAAACACACATGAATGAACATAGGTAAAAGCGAAAGTATGATATGTATATATATACATTACTGAAATTAGATAATGCAATAAAACTAAGATGGTATGAAATCAGCTAAGAAAATCATAAAAGACCTGCATCACCAGCATCACTGTCTTTCCTTTTAATAAACCTTCGTCCTCCTCTCCACAGTGAATCCAAAAGCCCCATTACTAAGTGGCCTATTCATGGATTTGGATCACATATGCAATGAAAATGCAAGGCAATCTTTGCAAAACACATGCATTTCTATGATTATCTATACCAATCAGATGCAGAAGCCAAAACAGAAAGGCAGAGAGAAAA	GTGTTGATATAAATACGAATTAATAGGCCATGTTGCTTAAAATACAATCAACGCAAAAGGGTGGGGAAACAGAAAATTTACATTGTGAAAGGAGGGGTTTTTGAAATGCATGAAAAGAAAAAGACAAAAGAGAAAGTGAAGGGAATTTACTTTCTTCTCCAAAGGAATATTGCATCTGATGAGAGTGCCAACAAAAGGGCACCACGTGTTTGTGAAATGAACCTAGTAGAAAGTGTAGGTGAGGGTAGTAGCAACACCTGTCTACCTCGTACACTAGGAATTGATGTAACTGTTGATTTACGGGGAAGTTAATGTGACATCATGAAATCAAATATTGTTATATATATCATTATGAAATAGTCAAACACTTGTTGCAACCACCACCATGCACTTTTTTTGTTTTTAGGTATCATTGCCTAATCTAATGCCTTCAACACTACAACCTCTTCTTTTCTGCTTTCATATCAGCTTAACTTGATGCAGAAGTTTCATCTCACTCA

Chr08	62,199,068	G	A	G/G	A/A	TA=intron_variant	TGN=Phvul.008G280800	CACAGTATCTAAACTATACTATTACAATACTTGACTTCAACAAGTTCTCATTAGCTTCCAAGATAATGGAGAATGAAAGAGAAGAAGAGATTGAGGACATGTCAATGTCACCCCCATCTCTTGGCTCCATGCAATTTGCAGGGAGCAATGGCTTTGGTCACAGCATGGAGTTCATGTCTCAGGCTTATCTTCCGAATATGTACACAGAGATTGACATCAAAATTGAGGATTCCAATTTCAACCAAGATCCCCCCTTCCCCATTTATCTCAAGGTAGAGAAAAATGGTTAAACTCTGCAACTTAATCAGGATTTTCTTTCCCCCTACATCACCACAGATTATTGCATGGTTAAAAACTATGTATAAATAATTTTGGTCAAATTCTATGTGACATGTAACTTACTTTTTCAAATTATGAAAGAGTTAATAGAACTCAGCTAGACAGCTACAAAAATTGGCTGGAATTGTAGATCATATGATAATTTATTCTATTGCATTAGA	AATGTTTTTGTTTTTATCAGAATCTTAGTTCTTCCTGCAATGCACTTAGAGGGTCTTGATGATTTTCCACTTACTGATTTCAATCTGTCAGAATATCATCATTAACAGTGTTTGCTTCTTGATTGTATAGTTGTTATGATTGTTGTTATTTGCCATAAACATTCTCAATAATATGCAGTTTGAAGATGTAGAGTATAAGGTGAGAGGTAGCCAAGTGGCTTCCACTAATCCTGTGAAGTCAGTGATGTCAAAAGTTGCCACACAACACACCATGGAAGAAGATAGATACAAGAAAATTTTGAAGGGTATGACAGGAAGCATTGGCCCTGGTGAAATTCTTGCTTTGATGGGTCCTTCTGGTAGTGGAAAGACAACCTTGTTGAGAGTTGTAGGGGGAAGATTGGTAGACAATGTAAAGGGAAAGATAACTTACAATGATGTTCGATTTAATCCTGCTGTCAAAAGGAGGTTAGAAAACTTCATAGGCATCGGCATCAGCA
Chr08	62,200,815	T	C	T/T	C/C	TA=synonymous_variant	TGN=Phvul.008G280800	AGGTGGAGGATATCTCAAAAGCATCTCTGGAGGAGAAAGGAAGAGAACCAGCATAGGCTATGAAATCCTTGTTGATCCTTCACTGCTTCTACTTGATGAACCAACTTCAGGCCTTGATTCCACAGCAGCTAACAGACTCCTTGTCACTCTTCAAGGACTTGCCAAGGTATCTGTAACCATATTTAATTCTCATTTTGATAACCCCCCAAGAATTTGGAATATATGCTGAAAATACTTTGGATTTGCCTAAAGGGTGGAAGAACCATAATCACAACAATTCACCAGCCATCCAGCAGAATCTTTCACATGTTTGACAAACTTCTTCTTATATCAGAAGGATACCCCATTTACTATGGGAAGGCGAAGGACGCAATGCAGTACTTTTCATCGTTGAGGTTCACCCCAGAAATACCAATGAATCCTGCAGAATTCATGCTTGACTTGGCAACTGGACAAGTGAATAACATAAGCTTTCCACAAGATGTTTTCAAAGAACAAGA	CTGCTGACAGTTCAAAATCTGTTATTGAAGTAAGACTCTGCATTATATATCTTTTATGCATGTTCAATCTTATTATACACCACCTAATAACCCTTCCCTTGATCTGTTAGTATCTACAACTCAAGTATAAAGAAACACTGGAACCAAAAGAAAAAGATGAGAATCATGGAGCAGCAAACACACCAGAACATCTTCAGCTAGCCATTCAGGTTAAAAAGGACTGGACAGTGAGTTGGTTGGACCAATTCTTCATTCTTTACAAGAGAACATTCAGAGCAAGATGCAAGGACTATTTTGATAAATTAAGATTAGTTCAAGCACTTGGCATTGCTCTTCTGTTGGGCCTACTTTGGTGGAAATCTTCAACCAATACAGAGGCTCAAGTTAGAGATCAGGTAGTTTCAAAACCCTTACACAAAAAGACCTATCTGAGACTCTCTTAGACTAACATTATAATGGACCACTATCTTTTCTGCAGGTTGGTTTAATGTTCTATATCT
Chr08	62,222,396	G	A	G|G	A|A	TA=intergenic_variant		ATAGTAAATATTTATATATATTATTAGTTTTCATTACATTACAATCTTTTAAAAAAAAACTAAATTTATATAATAGATATATCATATTTTTAAACTAAACCATGTATGTTCCACTGATCAAAATACTAGTATATATATATTATGAATTTTTAAAAAATACATCTTTTGTTTTGATTTTATATTTTTCTGCCCTTCTAATTATCATTTTTTCTTTCTACCTGACATTTTGCAAATCAATTAGTCAATATATTATATTAGAAAAGGTTTTTGATGTAGGTTTCAAATATATTTTCGTTTATATGGAAAGATAGATGGTAAATATTATGAGATGAAATTTCTTACCATTTTAAGTTTGGAATTAAAGTTAAGCAATATAAAAGGAGTTTTATGAGACACAAGAATATGTCAAGTGACAAATTAAGAATATGTCAAGTAACAGTTTATGATAAATTTGATTTTGATTCACATTTTATATGTAATTGTTCTTGGATAATGAAAGG	GGTCATTCTCACTAAAAATAAAGAACAAATATTCTTTTGAGTTAAAGAGTAAATCTATGCATTTAAGTCACTTTATGAGATATGTTAATGGGGATTGACATTCTCTTGGTTTTATTGTTTTAATGATTAGTGATTTTGTTTCATATTTAAATTCCCAACTTTGAGAACTCACAAAACTACAAAACTTAATTGATCCATTTAGGATTATGAAGATACCAAATAGTAATGCTCTTTTTCATATTTTTTTAGTATGGTTTTAAAGAAATGTGTATTTCTCTTCATGCACAATTTTAAAATCATGGAGAATCAAGGACAATATATAACATCCCCAAAAGTTATTACTTATAAAATAAATGGCATCAAATGAAAAAAAAAAAAACACTGACTTGGATTTATGTATCCAGAACATTAGATATATGGGCCGAAAATCCCTAATCCCAAATCTATCATCTACCAAATAAGACTTTTTCTTCCTGCATCCCCTTTTTCTTGTTGCACTCC
Chr08	62,233,744	T	C	C/C	T/T	TA=intergenic_variant		AATTTCCAGAAAATGGGGCACGGGGAGCCATTCTGTTTCTGTTCTGTGCTTACATTTGCTTCTATTATTGCAAGCTAAAGAGCTGGCTAAGGCAAACCAAACATAATAAACAAACACCAAGGAAAAAGGCTTAATTGAAGTGCATTAAATTCTCAAAAGAGGTAACATTATTGTTCATAATTATCTTACTCAGATAAAACTGTATAGAAGAATACTGAAAACGTAATAGATAGTAACTGTAATAATGGATAGTCGAGAAGGTCGAATTAGTCTCTTAATCCAATTACAATTTTTAAATTTACAACTAACTGTAATTAAGTTAGTTTAATGGTAAATCATGATTACAACATGATTGAATCCTCTTTTAAACTTTGAGCAGGTCCATGACAACTGAGGCCCATTCGAAAGATATAAATATCAGAGAAGATCCAACACCAGAGATCATTTTTTACTCTCAACTCACTGTATATTGTACTCGAACTAACTCGATCGTCAGAGTG	TTTGCAGATTCTCCACTTATCTAGATAAGAAAAAGAACTAATCAAAGACCACCGTATCTGAAGACCGTGAAGACCGAAAACGAAGACAAAAAAACCACTTTAAGAACAATCCGACCTTCTAAGAACAACTCGACCTTTTAAGAACAGTCACTAGTATTGTAAATGGCTTATTTTTTTTATAACCAAACAAAGACAGAGAGAGGGTACAAAGTACTCCAACCCTTCTTGTGTATACACAAAGACATGTCCAATGCTTTTATTCTCTCACCAACAAATTTTACCAAGCATCATATCCTGCTACTTACAATTCATATTGTCCACTCAGAGAATGAAAAGAAAGTTCCTCACCAACAAGAGAACCAAGTACAAGATCTGTACTTAGATAACTCAAATACACATTCAATGTCAGGATTCTCATTTTTCAAAACTTATTATTTTAATTATGTGAGTGGAATTACCCTGCTCTAATGGCCATATTCTTGAGCACAGACATATCAAAT
Chr08	62,239,301	C	T	T/T	C/C	TA=5_prime_UTR_variant	TGN=Phvul.008G281500	ATCTATATGTATAGAACACCAATCTATATGTATAGAGTAACATGTGTTGAAAAAAGTCATATCAGTGTATGAATTTCATTATTTCAATATATTAGTCTTTAATTTTGTTCAAGAATACACCTACTTTCCAAAAACAAATGAATAAAAAGAGAAGTTTGGCTGAATAAAAACCAACAACCATCATCATGGGTATGGACAGGTCCTTGTCATTGAACAGGAACCTATTACACTAATAAAAGAGTGAGGAAGCTGGCTCTTGTGTTCAGAGCTTCTTCTCTTCCTCTATAAACAATTCATAGGATTTCCCCCTCTACAATCCCAAAACAGATCACCACTTCTCTGCTCTAAGCAATGTCTGTAAGCTTAAGGAATCACTCACTTCCTCCCAAAAGCTTCTCCCTCACCTTGCTTCTTTTGTTGAACTCTCTTTTGTTTTCTTCTTGTTACTCCCTGCTTAATGAGCAAGGCCAAGCTCTTTTAGCATGGAAAAACAGTTTAAA	GTACTGCAGATGCATTAGCATCTTGGAATCCTTCCAGCCCAAGCCCATGCAATTGGTTTGGGGTGAGCTGCAACTTACATGGAGAGGTGGTTGAGATAAACTTGAAGTCAGTGAACTTGCAAGGCTCATTACCTTCAAATTTTCAAGCTCTAAGATCCTTGAAGATTCTTGCCCTCTCAACAGCCAACATCACAGGCAGGATTCCAAAGGAGATTGGAGACTGTAAAGAACTCACTTTCATTGATCTCAGTGGCAATTCTCTCTTTGGTGAAATCCCAGAAGAAATTTGCAGGCTGAGCAAACTGCAGACTTTAGCTCTGCATGCCAACTTTCTTGAAGGCAACATTCCATCCATCATTGGAAATTTATCAAGTCTGGTGAACTTGACACTCTATGACAACAAATTCAGTGGCGAAATTCCAAAGAGTATTGGCTCATTAACTGAGCTCCAAGTGTTGAGAGTAGGAGGGAACACAAACATTAAGGGTGAGGTGCCATGG
Chr08	62,239,532	A	G	G/G	A/A	TA=5_prime_UTR_variant	TGN=Phvul.008G281500	AATAAAAGAGTGAGGAAGCTGGCTCTTGTGTTCAGAGCTTCTTCTCTTCCTCTATAAACAATTCATAGGATTTCCCCCTCTACAATCCCAAAACAGATCACCACTTCTCTGCTCTAAGCAATGTCTGTAAGCTTAAGGAATCACTCACTTCCTCCCAAAAGCTTCTCCCTCACCTTGCTTCTTTTGTTGAACTCTCTTTTGTTTTCTTCTTGTTACTCCCTGCTTAATGAGCAAGGCCAAGCTCTTTTAGCATGGAAAAACAGTTTAAACAGTACTGCAGATGCATTAGCATCTTGGAATCCTTCCAGCCCAAGCCCATGCAATTGGTTTGGGGTGAGCTGCAACTTACATGGAGAGGTGGTTGAGATAAACTTGAAGTCAGTGAACTTGCAAGGCTCATTACCTTCAAATTTTCAAGCTCTAAGATCCTTGAAGATTCTTGCCCTCTCAACAGCCAACATCACAGGCAGGATTCCAAAGGAGATTGGAGACTGTAAAGA	TCACTTTCATTGATCTCAGTGGCAATTCTCTCTTTGGTGAAATCCCAGAAGAAATTTGCAGGCTGAGCAAACTGCAGACTTTAGCTCTGCATGCCAACTTTCTTGAAGGCAACATTCCATCCATCATTGGAAATTTATCAAGTCTGGTGAACTTGACACTCTATGACAACAAATTCAGTGGCGAAATTCCAAAGAGTATTGGCTCATTAACTGAGCTCCAAGTGTTGAGAGTAGGAGGGAACACAAACATTAAGGGTGAGGTGCCATGGGAAATTGGTAACTGCACCAACTTGGTGGTGTTAGGCCTTGCAGAAACCAGCATTTCTGGGAATCTTCCTTCTTCAATAGGGATGCTGAAAAGAATTCAGACCATAGCCATTTACACAACTCTGCTTTCAGGCTCTATACCAGAAGAGATAGGGAAGTGCAGTGAGTTGCAGAACCTGTACTTGTATCAGAACTCAATCTCTGGTTCAATCCCAAGTCAAATTGGAGAGCTC
Chr08	62,241,339	T	C	C/C	T/T	TA=synonymous_variant	TGN=Phvul.008G281500	ACTTGGGAAAGAATCAACTCAGAGGAAGCATCCCTGCTGAGATCCTATCTTGCTCCAAGTTACAACTTCTGGACTTGGGAAACAATAGTTTCTCTGGAGAAATTCCCAAGGAAGTTGCTCAAATTCCTTCACTAGAAATCGTTCTCAATCTGAGCTGTAACCAGTTTTCTGGTGAAATTCCACCCCAGTTCTCTGGTCTTAGGAAACTTGAAGTGCTTGACCTCTCCCACAACAAGCTCTCAGGGAAGTTGGATGCTCTATCTGACCTTCAAAACCTTGTTTTGTTAAATGTTTCCTTCAATGATTTCACTGGAGAATTGGCTAATACCCCATTCTTCAGGAAGCTCCCTCTCAGTGATCTCACAGGAAATGATGGCCTCTACATTGTTGGTGTTGCAACTCCAAAGGAAGCCAAGGGTCATGCCAGGTTGTTCATGAAGATTACATTGTCCATTCTCTTAAGCATCAGTGCAGCACTAGTACTGCTCACAGTCCTTGTC	AATTCGTGCTCATGTTTCTAACAAAGTACTCATGAGAAACAACAATTGGGTGTTAACTTTGTATCAGAAGTTTGAATTGTCTGTTGATGATATTGTTCGGAATTTGACTTCATCCAATGTGATTGGCACTGGAAGCTCTGGAGTTGTGTACAAGGTTACAGTTCCAAATGGACAAACAGTAGCAGTTAAAAAGATGTGGTCATCGGCAGAGTCAGGAGCATTTACTTCTGAAATTCAAACACTAAGTTCAATCAGACACAAGAACATCATCAAGCTTCTGGGTTGGGGGTCCAGCAAGAACATGAAGCTGCTGTTCTATGAGTACCTCCCTAATGGAAGTCTGAGCTCATTACTTCATGGTTTAGGAAAAGGAAAAGCAGAATGGGAGACCAGATATGATGTTATGGTAGGAGTGGCTCATGCACTTGCATATTTGCACCATGATTGCGAGCCTTCCATATTGCATGGAGATGTTAAAGCCATGAACGTGTTGTTAGGTC
Chr08	62,249,858	A	G	A/A	G/G	TA=missense_variant	TGN=Phvul.008G281600	CTAACATCCTCTGACTTATAACTGAACCACTCGACTGGAGCCACCATTCTGTAACAACCACTACACTTCCTGACTCATAACCAATTGGCACCATGTACCACTAGCTCCTTGGCGTGTCATCTTCACACACCTGCACCAAATTGATGCATGAAGCTTTGTCAACTCGAGCAACCAACTAACTCACATATACACTAAGTCTCTCAAAGGGTCTCCTGTCGTGTTGATAAAATTTATAACAAACTTGACTAGTATACTAGCATGGCAAAAATTGTTTTTTCTTGCACTTAAAAATGAATAAGCATTATGCTCGCCCATACTTGGTTCAGAAACTCCTCAATTTGTTTGACTATATAGTTCATACATAGCTAGTCAAGCATTGAATAAATTAAATTTTCAAGAAAATCAAAAGGGGAACAACAAACCTAGCTGCAAGAAATTTAACAGCAGATTCCTCAACATCACTAATAAATTTGTCAATTTGTATCAAATCCTGGTCACCC	GTCTCCTCCTTGATTGATTGTCAACTCTGCTACCTCTTCAATTTGATCGAAAGAACCTAAAATCCACAAATTACAACGCTCAGAATCCTCCATTATAACAAATTGATATAAAAATGCCACAAGACGAGAGTTAAAACAGGTTAAAGCAAGATATCAAATGCAATTCCTATAGTGACAAAAAACAAATAAATTGTGAATCTAGAAGCTTTATAAACCACCCGCAATGTTAACAGTATAATATAAGAAGATCTTATTAGACCCAGGAGAAAATTAGCTTTAGTTAACCATGAGGACCAACACTGCTTCTCCTTCTTTTAAATTAATAATCATCATTTTCTGTAAGCAGCTTACTTGTTCAATTTATCAATTACGAAATCACAAAGTAGCCTCAGAAGCGTGAACAACCTCTTTCATTGTTTGTTTTCATAACAAGCTGAGATTAATCAGTACCACAATATAATAAATTGTATCACCACACCTAAAACTATGTCACCAATCCC
Chr08	62,255,442	T	C	T/T	C/C	TA=intron_variant	TGN=Phvul.008G281800	TCCACCTTGTCTTACCACACCAAGAGCAGCTTGCTTGAGTTGTTCCTCTTCATGTTCTTGATTAACTTTTCGGGAGTTTATCTTCTGGAGGTTGTTGAAGAGAGTTTCATTAGTGCGATCTTGCTTCACTGACTCCTGTGAATGCAGATTCACCCTTGAGTTATTGTCTATAGAGGAGGGCATTGGCATTGTGACAAGAGATTTATTTACTAGTACTCTAGATGGAAGAAGATGCTTTAATATTGGTTGATTTTCTGCTGTGTCACTTCGGACCTTGCTTTTAATGACTGACCCTCTACCAGAGGATATTGATCTCCTCCTTACTGGAGGAGATGGTGATCTACTTTTCCCTGAGCTCACTAATTTTTCTTCTGCCAGAATGGACATTTTTGGCATTGACTCCTTTTCTGTATGAGTTGAGGGAAATCTTGACCTTCTTTGCTTACCCGAAGAACAAGTTTTAGCCTGAAATTATAATTTCAGGTTTAGAGACTACAAAT	CACTTTAGTGTAACACAATTTTCTAATATATGTTCACACAAATTTATATTAAAGTTTTTGTCAGCATGCAATAACCTCAGAACTTCTATCGTCCATGTGTCTTTGATAGTTTTCAGGCTTCATGCTGTCACTGGTACCATTTTTGGGCAAACGGAAAGGTGAAACAGCTCTTGGTGCTTTCTGAGATTCTGTAGGATTTCGTGCATTCCCAGATTTCCATTGCTGTAGTTCAGTTTCCTTCCTCTCCAATGCTGATTTCATATTTGATATCTATAAAATGTTCAAACTATTAAAACATTGGCCAAGAAAAACTGAAAACCGCTCAAGTTATGCAGAAAACCAAACCTCTTCCTTTAGCTCCCGAATTTCTCCAGTTTCTTTATTAGATTGAGCAGCCCCAAGTTCAATGGTCGCAACCCTCTCTGCAAACTTAAGTGTGCTAATTGTCTCTCCAAGAGCATTAAGCTCAGGATTTATATGAACAAACATCAAAGTTTTTG
Chr08	62,259,923	A	C	A/A	C/C	TA=intron_variant	TGN=Phvul.008G281800	AACACTTTCAGTGCCGAGTATGTTGGAAGGAGACCATTTGGGGAAGGAAGTAATCCTGACAGTTCCACCATATCTCCACACACCAACTCCACCCGACAGCTTCCATTCATAATACCCTTTCAAACACAGAATGCAGTCCACAACTTTGCTCGACGAACCCCCCTACAAAAACACATTAAACCCTTAAAAAGATGCTCATGTTTTTACATAACAGAATAAATAAACAATTTGTAACTCTACGAATCCAAACGGGCATTACTAAAATTGAAATAAAGAAGACAGGTTCCATTTTTATTCATAAAAGAATTATGTTTCTGTCTATCATATATGGCCTGTTTGGAGGAAATTTCTCCATAAGATCTTCTTGGAGAGAAAAATAAAATCAGCTTTTCTATAAAATGAAATTAGATTATGCATGAATAAAAAATCAGATTTGGAGAAGTTAATTGTACAAACTACCTTATGGAAAAGTTAATCTCAACTTATGGAGGAACTGATTC	TTTTTTTCTTCTAAAAGTGTTTATGGAAAAGTTTCTCCAAACAAGCCCAGTAGCTCTATTGAATTTTGATAACTTCACTGACTTAATACTTCACAAATACTCATAAGCGAGGTACCCAAGAGTAAAAGATATGAATTGCTGAAGCATATCAAAGTAATAGTATTTTGTGACCCAACCCCATAAAAACAGTACAGAAAAGGTAGCATCTTTAAACGTGAAAGGGGTGAGGGTCATGATCATGGTCACCTTTTCCAAATCAGAAGCTTCGAATGTCAAAAGCTTCATGTTGGTGACGGCCTCCAGAAAGTTTCGCATGTTCTCGAAATACTGAATTGCGGACTGGGCGGGTCCCTCGGAAGACGGAAGGGCGAGGTTGTCGACCACCGCATTGTCGACAACCTTGACGACGGCGCCGGGGTTGACTCTGTTGAGGACGTTGCAGAGAATGAGACCGTTCCTCAGTGCGAGGCAGAATTCCTCTTCGGAGGGTTCTTTGGAGA

Chr08	62,272,188	G	A	A|A	G|G	TA=intron_variant	TGN=Phvul.008G281900	TCATAAAATCATAATTTTGTCTATTTGTGTAATGGAAGTCAATGTTGGTACTTGTTACATTAACTTTGTCTTGTTTGTTGGGCCTCATCTAATGTACACTATGGTACTAAGTATACCAAGGATTTGGTTGCTATGAACTACCCCATATGTGAATTGTGCTGTTCTGTTAAGGCAGAGGAATATCACCAGTTTTCTTTTTCCTCCCGCATTAGCAAATGATTCCAGTGAGTTGGTGTTGTTAAGTGAAGAAGAACATATTACCTTTCAAGACATCGTACTTCAATGCAATTTTGTTTTAGATTAGATCAACGAAATTTCTTGAAACAGAAGTTTATCTGATTCTGAAAGATTAACTACCACCAACTCAGAATTCATATTTTAATCGTATTTATTTTTCCTCTCTAGGAAGTAAAATTATCCAAAATGAACATACTTCAGGTTCTTGCATCACTTCACCTGATTATTCCTGTTACTTTGTCATTCTATAGTTTTGAAATTGT	TTACCTCAGCACCTAACACATAAATTAGAGCTTCCGGCAAACTTGAGTTTTTCTTTGTTTCAAAGTTAGAAGCAGGAGCTTTGAAAATAAGATCTCAACAACTATGGATGTCTTATTTTCACATCTTAACAATTTTTATTAGTTATCTATGATTCTAAATCCCTTTCTATTTACTTTTTCGTTTGTGTATGTGACAGGTGTTGCTGGTGGAGCAGCATCTGGTAAGACATCAGTTTGCGATATGATTATTCAGCAGCTTCATGACCAACGAGTTGTACTTGTTAATCAGGTATTTATATCCTACAAATTATATTTGAACTCCCTTGGTATTTTGTTATTGTCCCTGTGATATCCCAAAAATCTTTTGAATTGATTGTTTTCACCTCTATTTCTTTTTTCATTCATATTCAGCATTTTGTTGTTCTCATCATCTCTTTTACCATGAATTTGTATGTTTTCCTCCCTGAATTTCCGTAAAAATATCAATGATTTTCTCACCA
Chr08	62,275,909	T	C	C/C	T/T	TA=intron_variant	TGN=Phvul.008G281900	GTATGCATACCCTGATTCGTGATTCTCAAACAACGAAACATGACTTTATATTTTATTCTGACCGTTTGATTCGTTTGGTATGTAAACGGGGTTCATTTGTTGCTCTTAATTTTAGTGGTGACATGACATTTTCTTTAAGCATTAATGGTGAATTTTTTCTCACTTGAACAGGTTGTAGAACATGGCCTAGGGCATCTTCCATTTACGGAAAAGCAAGTATTCACTCCAACTGGTAAGCTGTTCTTTTCAATGATGATTTTCTTGGCTTGTATTCGTAGTTGACATTCTCCTTCGAACATTCAAAAGTAGAACTAGTTTGTTTCGTGTCTTATTTTCTTATGGTTACATTGATACTTGTCTTTTAATGCAGGGTCGGTTTACAGTGGCGTGGATTTCTGTAAGAGGTTGTGTGGTGTCTCCATCATCCGGAGGTATCGTTTTAGTGCAGATTTTTTTCTATGATTATCTTTGTCATCCTTGCTATAAAGTATTGACTAAAA	TTTTCTTTCCTATTACTTGGTTTGATGTGTTCCTATGATGATAATGCAATTTTATATAAACAAGAACAAATTGCTTAGAGAAAAAAACTGTCCTTAAATTTGATTTACAATCTAATCTCAGTGGCGAGAGTATGGAAAATGCTTTACGAGCATGCTGCAAAGGCATCAAGATTGGGAAAATTCTTATTCACAGAGAAGGTGACAATGGTCAACAGGTTTGTCATCTGTGTGCTTGGTTATGTATGCTTTTGTTTCATTTCAGGGTCACTAACCTATCCTTTGATCATTGAGCAGCTAATATATGAGAAGTTGCCTAAGGATATCTCAGATAGACATGTATTGCTGTTGGATCCTATCTTAGGCACAGGTTTGCATTTCTCATCATCTTTTCTTTTATGCTAACTGTTTGTTACGTGACTTATTAAGTTAAATACTTAGTTTAATGATTTTTTATTTTGAATAACATTTCATCTTTAGGTAATTCGGCTGTGCAAGCAATT
Chr08	62,314,307	C	A	C|C	A|A	TA=intron_variant	TGN=Phvul.008G282700	CAGAAAGTGGTTGGAATGTGGCTCTTTGGCTCTGCAGCATGGGTGTTCAGCATGGTGGTGCTTGGGGGTTTAACCAGACTCACCCGATCTGGTCTTTCAATGACTGATTGGAAATTCACTGGCACTCTTCCTCCTTTGTCAGACGAGGAATGGTTGAAAGAGTTTGACAAGTATAAGGAGTCACCTGAGTACAAACGGTAAACCGCAATCACTTGCTCCTCCTGAGTGAGCATATACTGTTGTAGAGTTTATAATTCTACACGTTTAGTTTTTCCATCAACTTATGAAATGCATCAAGGGACTTGTTTTTTCTGGGTGAGAAGACACACAAAGTCATCAATTCATGTAAATAAGTCCTTTTCAAGTCGGCTGACTAATTGTTAAAGCTTATAAAATCAAATCATAAAAATTCAATGATTAAAATTCTAGGAATAATGTTTCATACAACTGTGCTCAAACTTCATCATAAAATTAACGTGGTTAAATAATAGTTCAAACTT	CACTGCAAGAAAAAAATGAGGAGCATGATTGTGTGATTCAGAGTATGGGGAGGATAATTAATATGCAGTGATGGAGAATAGTTGACTGCATTGGAACTGCGCCAATGGACAGGCTTGTTTTACCTTTCAAGTTCTTATTAGAATCTACAATTTTTTAAGTATTGATTGTGTATTTATTACAAGAAAAACAAGGTATTGATTAAGTGGAAGTGATTTGATTCTCAGTCTGATAGAGCTTAGTAGTTGTTATGAATGTCATGATGATAATTAGTTTTTCCCATGTTGCAGTGTTAACAGAGGCATGCAGATTGAAGAATTCAAATTCATCTATTGGATGGAATATGCACATCGTATGTGGGGAAGGGCGTTAGGAGTTATGTTTGCTTTGCCATATTCATATTTTCTTCATAAAGGGTATATTACCTTGAGATTAGGACTTAGACTCTCTGCTCTTTTTGCCCTGGGTGCTGGTCAGGGTCTCATTGGTTGGTGGATGGTCAA
Chr08	62,321,296	T	C	C/C	T/T	TA=intron_variant	TGN=Phvul.008G282800	TCTAAATTACGGACTCTTTTTGTTTTGTAAAAATTTGCCTTTTTGAATTTTCATTTTAAAAGATGTTTGTAAAATTAGAAATATTATATGATACAAAAAGTACTCTGTAAAACTTTATTTGATATAAAAAAAATGGCACCCTATGATTGGATTGGATTGCAAAGTGATTTGAATGACAAAGTACATTTTAATCTTAATTTGAAAATAAATAAATAGAACAAAAGTGTATATAATGAGAGAAAAATATGAGAAAAAGAAAAATCATTATTGCATAACGTAGTGTCACATAATAACAGTGACTATATATAGAAGACAAAATTGAAAAATCTGTAACCACGAAATCCAAAAGCACAAGCAAGATATGAATATTTAATTGATATTTCAACGAAATTTAGATAACTCAACAATAGACTTGTATTTGGTAAAATCCTCTTAAACTTTTTTTTTTTTTTTTCATTCAACTCTTATAGTTGTTGTTCGAAATATTGGGTACTACTTTG	ACCCTTAACGGGCTGGGTTAGCTATCAACGGGAACTACGATAATTGGGGCATTCCTTTTTGCACCCCTACAATTTCTTTCTATGTCCTACTAAATATTAAATTTTTTACTTTGTCATGAAAAATTGTTTTTCCGTTTGTATAATTTGAAAGTTACTTTTGTATGAAAAAAAATTTAGATTACATAATCTAAAATCCCATTTTTATATGATTTTTTTTTTTAAATTGTACAATTTAAATGTCATGATGAATTTGGGGTGTATTTGATTTCTATTCTTTATTTTTTTTTAATTTTCAAAATACAATTAAAACCCAGTGGGTAGAGAATAGTGTAATACACAGTAGAATATGGAATTGAGGAAAAATAAAATAAACAAGATAAAAACAGATGAGAAAACATATTTTTAAGTTGTTTCTGTTTTTAGGCTTTTAAAATACTTGTTCAATAAATGTTTTAAAAAAATAATTTTTAAATTAGTATGTAATTAACTAGTAATTATTTAA
Chr08	62,327,137	T	C	C/C	T/T	TA=downstream_transcript_variant	TGN=Phvul.008G282800	GGCTTTGTGAATAATTTCTACCAACTGAGAGTGCCTTTGTTGCCAGATTTTCAGCTAAACTTGTTGAGGATATGTTTTAGAACAGCCTATGTGGTTTCCACAACTGGACTTGCGATCCTATTTCCTTACTTTAATCAAGTTCTGGGAGTGCTAGGAGCCTTAGGATTTTGGCCATTGGCTATATATTTCCCAGTAGAGATGTATTTTGTGCAGAGGAAAATTGAAGCTTGGTCTAGAAAATGGATTGTCTTAAGAACCTTCAGCTTTATTTGCTTTCTTGTGTCACTACTAGGTCTCATTGGATCACTTGAAGGAATCATAAGCGAGAAACTAAGCTAATGAAGGGTGCTTCGTTTGTTTGTGTTCATTGTCTTGTTGGCATCTATTTCATGTATTTCAGTTTCACGTTAATTCCATTGTTTGTGAAGTTGGTCTTAGACATGGAAAAAGTCCTCTTTTGTTTGGTGAAGAATTCCCTTTAAACAAAGTGTAATATGGAT	AGACTTTTTTTCATTATGCATCCCTGTAAAATTTAATTTTATCTTTTTTAAAATTGTATTCCGAATTTTATAGATCAATAATCACTCGAAGTTTTTTTTTCAAAATACAAGGAAAAATACAACCCTCAACAATAGAGAATTTTAAAACAATATATATTTTCTCGAGTTATTAGATGATATATAATCAATTAGCCATCTTTACCAATTGCTTTGTAACAAAAACTGTAGCCAAACATTAGAATTTGATGACAAACTAGTACTAACGTGAGCCAAGAGTTACAAAAGTACAAAATCCAATAATAAAAATAACTTGTTCAAGCCACAACACAAGCAATACCAAGCTAGTAGTGATGGTAAATTAACAGCTTGGGTGGAAATATTTTCCGTCATGGTCAGTTTAAGGAATAACTTAAGCATTACTAAAAACATCAATATGGGCAAGTTCATTGCTGCTGTGACCCCGATGTTGATGTTCCAGATTCAGTTGGGGCATGTGCAGC
Chr08	62,335,359	T	C	T/T	C/C	TA=intergenic_variant		CTTTCTGCAATTTTATTCTGTGTTGAAAAATTCACACAAGTACTTTCATGTGTTGTTTGCACCCTGCGAAGGACGCACATCCATAAACAAATTACAAAAAGGAGATATAGGGAAGAAAAGCCAGGACATTTTGTTTTCGTAAAACCGGTTTTGTTCACTCTCGGTCTGGGTCTTACTACGCCCTATTCCTTGAGTCTTTTGGTCTGAAAATTTTACCACACATTCGTCATTGTGTGTATTGAGTTTTGGCTGAGATTTGAGCTCCAGATACGCCCCACAAGATTTTCAATAAATTTTTTATTCATCACTGCCAGGGTTGAAATAAAGGTTCGTTTGTGTGTGAAACTATTTGATTTTTTTCCTAGGTGAATTCTCATCCGTTTGTCGTGAAATTAGTCGCGTTTTGTCCCAAATTCAGTCCAGATTCTTATGTGGAATTTCAAGAAAAAATTATTCTAGGACAATTATTCTGAAATTTTGTGAAAAACCAAGGCTATCCT	AGAAAATTTGTGAAATTTCGCCATACTTTCTGCAATTTTATTATGTGTTGAAAAATTGCGTTAAAAAATTCACACAAGTACTTTCATGTGCTGTTTGCACCCTGCGAAGGAGGCAGGTCCATAAACAAATCACAAAAAGGAGATATAGGGAAGAAAAGCCAGGACATTTTGTTTTCGGAAAACCGGTTTTGTTCATTCTCTGTCTGGGTCTTACTACACCATATTCCTTGGGTCTTTTGATCTGAAAATTTTACCAGACATTCTTCATTTTGTGTATTGAGTTTTGGCTGAGATTTGAGCTCCAGATACGCCCCACAAGATTTTCAATAAATTTGTTATTCATCAGTGTCAGGGTTGAAAGGAAGGTTCGTTTGTGTGTGAAACTGTTTCATTTTTTTCTAGGTGAATACTCATCCGATTGACGTGAAATTTGTGAAAAACCAAGGATATCCTTTAGCAAAATTTTTGAAATTTCGCCATACTTTCTGCAATTTTATTCT
Chr08	62,341,545	A	C	C/C	A/A	TA=intron_variant	TGN=Phvul.008G283000	CAAGCTAAAGCTGATTCATTTACCTTTTAGTGGTGTTGAGCTCTGTTCGTCTATGCATGTGGTTAGGAGTTTGAATCCTAACTCTTGTGCGTGGAAAAAATCTAGTTGAGAACTCACTTTTGAGTGTTTCCAAATCTTCCAAAAGAACTTAGTCTCGATGAACAACCAAGGATATTCCTTACAGTAATGCATTACAACAACAAAATAACATCTTGGTACCACATTTTTACTGGAAAGAATGCTATTTATATGATAAGATCATCTTTGGGTGTTTACAGGAATAAGCAATGGACGGCTCAGTGGCCTTCTTGTGAATATAAGTTTGTATGGTTTTGCAATTGCCTTCGTCATTACAACAGCTATCAGCTTAAGGTGTGTTGATGACTTATCTTACTGGTTTCTCTCATGTTATGACATTTTCTTTTTTTTTTTTGCTCTTCTTTCCCTCTGTTTATCTATGGATGCTTTGCGAAACAAGATTTTTTCACTGGTACTCCACT	GGAAACATGAGTCTGTCCCCAGAATAACACCATATGATTGCCAATATTAATTTAGAGCTATTTTTGTATTTGTGGCTGCAGAACAATCCAACATTCATTTGTTACCATAACAAAGGGAGTGAAGCATCATGTGAATCTGTAGATGCATACTACATGCTGCTTTTTGGCGCTGTCCAAATTATTCTCTCACAGATACCCAACTTCCATAACATTGAATGGTTGTCGGTTGTTGCTGCAATTATGTCCTTTACTTATTCTTTCATAGGAATGGGACTTTCTATTGCAAAAATCATAGGTATGATAGGAGCCTTGGCTAAGGCCCACCAAAAGTATTAGTTTTGATCAGATAAATGTGTATAATTTTATGACAAAACTTTGCTCAGAGAAAGGACATGCTGAGGGTAGCATTGGAGGAATCAGTACCTCCAGTGGAACTGGAAAGTTATGGCTGGTTTCTCAAGCTCTTGGTGACATATCTTTCTCATTTCCATTCTCAACA
Chr08	62,356,391	A	C	A/A	C/C	TA=intron_variant	TGN=Phvul.008G283200	CTTGAAATAGGAGCACCCTATCTTGTAGACAGGCTCAATTATTTATTTTATCATAAAATTGTTTCTTGTGAACGTTTTGTTTTGACTGTCGAGGTGTTATGTTACAAGCTCTTCAACTTTCTTGTATTAATAATCTTTTACCTATGGTTACTGATCAATTTTTGGGAGGTATATTTTTAAGCATTTGAAGTGAAAAATATTCCTTCTTATAATGTTTATATATTAAGATGAGAATCCTTGGTAGATTGGGGAGAAAGAAGGGTTCAAAAGTTTGGTGGAATTTCAGGAACTATATATCAAGAACCGATTCAGTAAAATTGTTAAACTGAAGGAATATGGCCTTTGAAATTGGGCATTTGGACTATTGAATTCTATTATTTAGGCTATGCTTGTTAGTAAACTTGGAATCTCACTAATAATTAATTCTTCGATGTTTATAGTCGGTCTCCATTCTTTGTTGAATTGTTTTGGACTTGGGCTCAAACCCCTTTGATGATCCC	CATCCAACTTTTAACAAATGCATCAACTGGTAGTGTTTTCCTAAGGTGTGACTAACGTTTTGTGGGTCATAGGTCCCTCCTGACTTCTACATTCCGGATTTCGATGAAGATGAGCAGAACCCTGATGAGCGCATTGATCGTAAGGAAAAAGATAAAAATTATTAATTGCATTTCTGATCTCTATTTTAATCTGTGATCTTGGGATGTCTTATTTTGATTGCTCACATTTGGTCTTCATATTTTTCCAAATGTTCAAACTAAGTCGTAAACCTCAATTTTCAATTCAATAATTAGCATAAATATAATAAGGAAACGTATCATTGGTTTATTTAAATTAACCAAGAAAAATATGTAAAATGTTTGAGTAAAAATAGTATGAATGCACATGCATGCACCTCACACACAGAGGAAGAGAAAGAGAGTAACAGAGGAGATATTGTCCATCTCAAACCTTTATTTTCTGCATTTTCTGCAAGCAACTCATTTTATCAGTTAGATTA
Chr08	62,373,177	G	A	A/A	G/G	TA=synonymous_variant	TGN=Phvul.008G283500	ATAGTAACTAGTAGAGCCAACAGAAACCAAGTAAGGTTACAGTACATCAACATGCCACAACCTAACAAAGACATAGAAAGACAGGATTACGGACAAACTAAAATACGCACATTCTAGAAACTGTAGTTGATTACAAGATAAACACATGACTATTTCAAACGAGTTACTCAATTCCTAACTTCCAATGTACCACACAGAAATACGAACAGCGCTGCCTCATTTCTCGCAATAGCAACATCATCTTAAGCACCAATGTCTGAGAAACAAAACACGTAAAACAAACTCGAGAAAAACACGCTGTAACTACTCGAATTTACCTTGTCGTCAGCGGCAGAGAGGAGCCTGATCAAATGAGGGAAAGCTCCGGCGTCGAGAACGGCGCGGACGCCGGCATCGACGCCGCAGGCGAAGCTGCCAAGCGCGGCAGCGGACTGGACGATGAGGCTGGAGCCGGGAGCGGAATCGGCATCAGCGCCGGCGAGAACAGCAGCGAGGACGGG	CGGCGCCGAGCTTGATGTAGGAGAGTTTTTTGGTGCGGTTGCCGATGATCTGATTCTTCACCTCTCGAATGGCTTTGAGCTTGATTTCGCAATCGGAGGAGGCGAGGCGGTGGATAATCAGATCGGAGGAAGGACGCGTGGCCGGCATGGTGGCGGCGGAGGAAGGTCATGCACGGCGGAGGAGAAGGCGTGGTAACCTTCTCATTCCTGAAATTCGATGGCAGAAGAGAAGACAGAGATCTTTTTTGTTTGTTTGTTGAATTAATTAGTTATTAATTTTTATGAGAGGGAAAAAATTAGGGGCATTTATTTATGAACGGATAATATAATAGATAGATTGAAGCCGTGTTATTCCCTTTCACAATTTGGAAATGGCGCCCTTCATTTCAATTTCTGATTCCATACTCCTTTTATCTACACATAATACAATTTTTAAAATTTTATTATGTATTGCATCATGTACTCTACATTTTAAATAAATACATAAGTCATGTGTATTA
Chr08	62,395,046	C	T	C/C	T/T	TA=intron_variant	TGN=Phvul.008G283801	AGCAGAGGATATGATAGAGTTGTACAAGGAACTATGCTCGGGTACACCATTTTATCTTAAAATTTGACTAACGGGAATGTTATAATTTATTCAGCACAGGGCTTTGTGTTTATTTTGGATTTTTCTTTTTGAATTTGTAGAATACCCTATTGTGTCAATAGAAGATCCATTTGACAAGGAAGATTGGGAACATATCAAGTATATCTCAAGTCTTGGAATTTGTCAGGTATTTTTTTTAAAATTTTAATCAATTAATATTTTGTTTGAGTTAATGTCAATTCAATCTCTGTACTAGTTAATCTGTGCAGCAAAGATTGTGCTGTGCAGTAAGCTTTACCATTTTCGAGTTTGCATAATGTTTTAAATGATAACAGTGTACATATTTAATAAAAAATAAGAACAAATTGATTGTTAGTGTATAAATGCCATATTGACTCGTTAGCAGGCTATGTGGGCAAGGGAGGAATAAACATTTCTTATAAAATTGTTGTCTTTTTCCT	ATATCGGGATTGAGCATTAGATAGGTTAATTGCATATTGCGACTGAGATGTTTATTTTACATAAATTGGCTTCTGATGGAACTTCTGTTTATATAGTTTTTTCTTTATTTGAATGGTTCTGCAGATAGTTGGGGATGATCTCTTGATGTCAAATGCAAAACGCATTGAGAAAGCAATAGCTGAGTCTGCTTGTAATGCCCTTCTACTGAAGGTAAATTTTGTGACTTCAATAACTTTGAAGTATTTTGTTTTGCCTAATGAAAAAAATTAATCTTATATATATATATTTATTGTTTTGTTTTAAAATATTAAATACTAAACTCTGTTGTTTATGGAATGGAAGCAATCCTGTTTCAGAATTAAGTTGGGGTGTTGAATATCCACAAATATTGGAAAAAGTTGCTTTTCAAGAAAATACAAGATCATAAATTGCAAAGCTAGGTTATGTAAAAAGAAGAGAAATTGAAACACAACATGCCTGTTATTTTGAGTTGCCTGAGATG

Chr08	62,399,303	T	G	T|T	G|G	TA=downstream_transcript_variant	TGN=Phvul.008G283900	TCTTGATACATATTAAACTCTCTAATTATGAATTCTAGTCATTGTTTAACAATGAAGTCTAAA<br>AACACAAAAATACAGCAATGTTTTTTTTTTTTTTAATTTGAATTAGTTTTCTTTAAACAATTAG<br>TTAGCATTGCATGAATACTTTAATTTGTCTTTAAACCTTTTTCAAAAAGAATTAAAAATAGTT<br>GAATGAAATTTAGAATAGTTTAGTTTAAAAGATCTAATTTGGTTTGTTTGAACTGTAGAAAG<br>AGTTCAGTTAAATTGAAGTGAAGTATACGTTTTTGGTTTAGAACAAGTTTTGATAGTTTCGG<br>TCTAATCTTTCTTTTTTCCGATCCCACATTTTTTCCTACAGCAAATGACAGTAAAACACTGA<br>CATCAAAGCTTTTTAGTCACACAATATGAAATTCATCAATCCTGGCAAAATCTGTTCGGATC<br>ATGCTTGTATTTATAAAAAAGAGTAATCAATAAAGAATCTATTATCCATTGTGAAAACTATA	TCAGAACCACAAAATGTGCAAAGTAAAGTGGAAAGGAAGAAGATCACAAACACATGCAAAAC<br>AAATACTGAATTTCGAATTATTAGCTAAAAAGTATCTTGAAGAATGTCTTAGAAATCCTTCAAC<br>AGAATTCAAAATCTCATCAGATAAGAAATCCTACAACCGAATTTGAAATCCCATATCAGGTTTT<br>GTCTTCTCCTGAACCTCGTCCTGTTCAGTATTATTCTTCCCTAGGACGCAAAAAAAATCGAAC<br>ATAACCAGTCTCAGCAATTTTGTACTCGAACACAGAAGGCATCTCGCTCACAAAGCTGATGGT<br>GACTGTATTGCACAATGGCGCAGTCTTTGTGAAAGAATTTATCAACCTCAATGAAAATGTCAC<br>GCTCACACTCTCATTCATCTCTATCACAACTGCTTCCTCAGGCTGTATCCATGGCATAAACCC<br>ATCAGTCTGCAGTTCAAATTGCTGAATGCTATCCACACATAATTTCACACCCACATGA
Chr08	62,414,617	G	A	A/A	G/G	TA=intergenic_variant		GTAGGAATGTTGTTATCTTTATGGTCAAAAGAGATTATAAGAATAAGACTAAAAGTGTTCAC<br>TCAATCACCTAGGAGAGAGTGACTTACCTATTGTACATTTGTACACGTAAAAAAATGCAAA<br>CCTTTCCTAAAGTGAAAGAACATTTGAATTAGGAGACAAAACTTTCATCGTCAGACACATC<br>CTCCTTAGTGATCAGACCCAATGATCAATTAATCATTGAAACATGCAACCTTTAGACTTTAG<br>GCATGAGTGTTTGTTTGACAAATAATACTAAAAAGGTGGTCAGAAATGACCAGTTGTCAA<br>AAAAATACTCCTTAAACTAGGAATAACTTGGGAAAGAATAATCAATTGATCCAATTGCGCTT<br>AACATGATGCATGAAACAAATGGGATACAATCTGACTATTGATAAACTAAATCATCGTTTGA<br>ATACTATCATTTAGATGATATTTGTGAAAAGTTTTTAAATTTAATTAAATCAAAATTCAACTAT<br>CCTTT	TATGATTTTCCTGTTTTCTACACATACCTAACTATACATACAAGTTCTATTATTATAAATCCTTA<br>CTAATTTTATAGCACCACAAGACCTTTACATCAATTTTTTTCTTATTTAGAGAGATCTCAACAAA<br>ACTCAATAGGTAAAAGAAAATATTAAAATCTATGGCTTGGATTTTAAAAAGAATACTACAAATTA<br>GTAACATAACTTAACATGGTAAAAGGTACACTTTAAATTAGATTATGAGAAAAATATTTAAACTT<br>ACCATGAAAATAAATTAATTATATATTTTATCATTTAACTACATTATTTTGTGAATTTTATAATTT<br>GTACATGTTTTATTATTAAATCTTTTTATTCTCATGTGTTATAGTAATTCAATAAAATTATATCAT<br>ACAAATAATATACTGTAACAAAAAGTAAAACAAAGACTATTTTTTTTTTAAATATAGCTATATAG<br>AAATTTAATTGATTGGTAAATTATTTGAAGATTAAGATAGTTG
Chr08	62,439,928	T	G	G/G	T/T	TA=upstream_transcript_variant	TGN=Phvul.008G284200	TTTTCAGTTACTCAATCACTCATAAGTCTTAAGTTAACTACTTCTAACATCCTCAGTTTACC<br>GTTACAGTGTGGAACTCCGACAGAGCCACACGTGCTGCTGCTCAGCAACTCCCATTCTAC<br>TTTTAGCAACCTACATCAAGTGGGCCTACAATTCACACGTATTCACAGCCTGTGGGCCTCC<br>TCCTATGGCCCAAATTATAATGCCTCCAAAGTCCATTACCCTCCTCATGTCTCTCTCACTG<br>TCTCTTCTGAGACACAGTTCAATTTCAATTACCCACTCAGAAACAAAAAACTGTAATTGCTA<br>ATCATCACTAAAAATCAATTTTTGTTACCACTGAACAGTTTTCATTTGTGAATGAAGTTCAG<br>ATTCTTAGGTTGATGAGATATGTGATGTGGGGTCTCAAGTTCTGCAATGATCTTGTAGAGT<br>TCAAAATTAAAAATTTAATAATCTGCAAAATAAAAATTATTTTTTTGCATTAATTTTTATTATA<br>TATTTAT	GATAGTTGAATATCTTCTTTTTTAACCTTAAAAAATGATCTTATATTAAGTATTCTTATGTATGTA<br>TGACAGCACAATTCTTATTTGCTTTACATTTTATTTATCAGATTGATACAAAAGAGATTAAAACA<br>CAGGGATGATAATATTGTTAGAAGAAAGTAATTAGAGTGGCAGATTTGCAAAGTGTAACCTAT<br>TTGAAGGTGCATGTGTCCTAAACACAGCCTTTCAAGAGGTACTGAAACTGAAACAGGCATAAA<br>TAAAAAAATGTATACTTTGGCTCACCTACACTGGGGCTAATGCCCCTAGCATCAACCCTTAGC<br>TACACAATTTTTATTCACAAAGTAATGTCATGGCACTGCCCCTTCTGATGCTGCAGATTTTGTT<br>CACACAACAAGTCATTTGTCCACTTTCCACCAGCTTCAACATTTGAGCTTAAAGTTAAAACTGT<br>CACAAACTTGCACAGTGTGCAATGAGCAGCAATGCAAAACAACTTTGACTCT
Chr08	62,463,863	C	T	T|T	C|C	TA=intron_variant	TGN=Phvul.008G284500*	TTTAAGTGTTGTGTTTAATGTTTGAACATAGTTCTACTTGTTTACAGTAAATTATTTTCAGCT<br>TGAACAGGATTATATGCCAGAAAATACTTTCCCACTTCAAATGAACCAAGAAGATCCACCT<br>ACATCTGAAAATAAACCATGTTCTTCACAAGTCAATGATAACAACCAATCTATGTTGGGAA<br>GCACAGCTGAAAAAATTGAAACCAGAATAGAAGAGAAAATAGACAGAAAAAGTGAAAAGAT<br>ATCAGACATACAAGCGATGGACCAAAATTTTCCAGTCACTTCTTCAAACATGATTGAGGTA<br>TTTATATATTTTATTTTAATCTTCTAAAAACCTATACATTAATAAACTACGTGATGGAAGAAA<br>TTAAAATAACCACAACATAACGCCAACTTATGTTTTGGAGGTGAGAAATTCATCTCAAACTT<br>ATATGGTAACTCTCTTGCATAAAATGCATTTGTTTGTAAAATTAAAAATTACTAGGATTATAA<br>ACAGA	TCCTACTATACACACTAATTTTTGTTATGCTTTTTCAATATTTCAAATAAAAGTTGGTGCACAAA<br>GAATCAAACAATGAAACAGGTTTTTCACACCAAGATGCACACAGAGATATAGCAGTAACCACT<br>AAAATTGCTCAAATTATTGTTGTACTAACACATCATGTACGTTATGAAACAATTGTTTTTCAAAA<br>GCTATAACTTTAAAGATATGCAAACACTTCTCTTCCATTCGTGATGCACAGAATATTGAGGAAA<br>TAAGAAGACGAAAGAACGAAGACGGGCCTCCATCAGACAGTGGTGTTACTAAAACTTTGTCA<br>GGTTGTGTAGATAACATTAACTTGGGTAGAGGCGTTACAATTCAAGAGTCAAGTGGTGCATAT<br>ATACTTCCACAAGATTCCCAGGTTGTTAAACAAGATGATAAGATGAATGAAGACGAGGAAGGA<br>ATAATTCTTTTCTGGTGACCCTCTACAATTGCAACAACAACTCCAAGCATAAGTT
Chr08	62,466,863	G	T	T/T	G/G	TA=intergenic_variant		TTCTTGCACTCAAAGAATGTGAAATATTTGGGAAAAGAATAAAAGATGATCTATATATCTTC<br>ATTTTTTTCTATGGACAAGGTTACCTTTGCATGATATCTCCTTCATATGTTTATGCTCTAATT<br>TTTAATTAATTCATAAGTCCTCACAATATTTTTAAGAGGTAAGGTTTCATATTTTTCCTTAAA<br>ATCTTAGTTGCAAATTTCTTTAGATCAGATATTCAATATTCCTGATCAATGATACTGTTTCTT<br>GTAGCAGACTCTGAACCCTCTCTCTGGCCAAGTTACCCATTATTGCAGATAAAGAGGTAG<br>GTGATTCAAGCTTTCTGATTTTTTTATCTTCTATCCAGTTGCATTTTTTTTAATTTTAATTTTC<br>CCTAATTTCTCAGCACTCAAGGATTAGTTTTCCAATTTTATTGAAAGTAGGTTGAGATTTAG<br>ATATATAATGAGAATTAAATCTAAATGATGAATATCTTAAATGGAAAATATTATAGCCAACTA	TGATATAGCCAACTTAATCATTATACATACATATTTTGTAAAAGTTAAATTTTAGACTAAAGAAA<br>AACTTGAAGTATTTTTTTGACATCGTCACACCAGTGCAAAAATGCTTATTTACAACAACTATTAT<br>ACAACGTTTCATTATTGAACATTGTCTATCAAAATGCGGTAACATTTTTTCAAATTAAAACAGGT<br>AAATAGACAGCGGTTGTAAGACGAACCGTTGTCTATTTAAATCACATAAGGGATAGACAACGG<br>TTCTAACCCAACAACTGTTCATCTTATTTTCTTCAATTCTTCCATTGGTTCATTATTTAAGGTTC<br>TTGTTCATTGCTCCACCATCACACTCTGCTTTCCCTCCATCACTGTCGCCATTGCCCTTCATC<br>GCTGCCCTCTACCGCCAACGCTGCCATCACCTTAAGGTTGGTGCTGTTTTTTTTTTCCTATAT<br>GTGACTTATAGACTATGGTTTTAGAACTGTCATTTATACCTCAATAGAATA
Chr08	62,468,870	G	A	A/A	G/G	TA=intergenic_variant		GTGAAGAATACTAAGATTGATGCAGAAGAAGTGAATCACTTGCTCAAAGAGGCAAAAACC<br>AAAAAAAGTTGTTGTTTAGGGTACTGTCCAAATTGGATTTGGCGATACCGTTTACGGAAGA<br>AGTTGGCAAATAAAAAAATGGACCTTCAAAATATCATTCAAGAAGGTAAACAATATATACAG<br>CTTGAACGCATTGCTTCAATTCCTTCAAATACTCTTGATATTCTCACAGAAAAATGTATGAA<br>TTTTGATAGTAGAAAAAATGCTTCTGATCAACTAATGGAAGCACTGAAAGATGATGGAGTT<br>GCCATGATTGGATTGTATGGGATGGGGGGTTGTGGTAAAACCACATTAGCAATGGAAATT<br>AAGAAGATGGCAGAAGATGAACATCTTTTTGACAAAATTATTTTTGTGCCTGTGTCTAGTAT<br>AGTAGAAGTTCCAAGGATTCAAGAAAGAATTGCAAGTTCATTGCAGTATGAATTTCCAGAA<br>AATGAAGAAAT	AGAGAGCCCAACGCTTGTGCATGAGATTAACCTAAGAAAAAACAATTCTTATGATTCTAGATG<br>ATGTGTGGGAGAAGCTTGATTTTGGTCGCATAGGGATTCCCTCCTCTGAACACCATAAAGGTT<br>GCAAGATTCTCATTACCACTAGATCGGAAGAAGTTTGTACTTCAATGGATTGTCAAAGAAAAA<br>TTTATTTGCCAATCTTAACTGATGTAGAAGCATGGAGTCTGTTCCAAAACAAAGCAAATATATC<br>TGAAGACACCCCTAAACCCATCAAGCATTTAGCAAAAGCAATTTCAAATGAATGTAAAGGATT<br>GCCTGTTGCCATTGCAACTGTTGCTAGTAGCTTAAAGGCCAAAGAAGAAGTGATATGGAGTG<br>TTGCGTTGAATAGATTGAGAAGTTCTAAGCCAATCAATATTGAAAAAGGTTTGCAAGATCCCT<br>ATAAGTGTTTACAGTTGAGTTATGATAATCTAGATACGAAAGAAGCTAAATCACTATTC
Chr08	62,472,941	C	A	A|A	C|C	TA=intergenic_variant		CTTTGTTTAGTTAAAAATAAATTTCAATTCCTTTTTTGAAATTTCAGTTTTCAAAAAGAATATA<br>TTAAAAATCAATCACTTAAATAATTAAAGACACATTGTGTGAATTGCAGATATCTAGCTCTG<br>ATTCTCTTCACTTGTTTAAAAGTGCTGAATATCTTTCAGAGCAACCACAAGGTCTGAATTTT<br>CTCATCATGCATAATATTTGAGTAATTAATCTAAAAGGTTTTGATAATGCAAAATACCTTTTT<br>GATTTGTCTATTGCTTCATTATTCATGTTGCGAAATTTGCGTATTGAGAATTGTCCTAAACT<br>TCTGCACATAATAGACATTGGTGATGAATATGAGAGTAAGAATTTGGATGCTATCTTTCCA<br>AACTTAAGATCTCTTTCTGTGTACAATTGTGAGCAATTGAAATATATGATTGGTCAATGCCC<br>TCTTGATAATAAAAATGATAAGGAGATTCATCTTCATTTTCCAACATTGGAAAAACTCTATC<br>TT	TAAGCTACCAAACATTATTAGTATTTGTGCTACCAACTTTCTGTCCATGGCATGGCCCTCTTTG<br>AACATATTTGAGTGCTCTAGATGCTCCAAACTTGTTAATAATAGCCCAAGTCGCAAGGTTGTC<br>CTTATTCTTTTAACTATTAGTTATTAGTTCTTTTTTGTTTCCTATGTGGAATCTCTTTTTTTACAT<br>TTTCTATTAGTGTCTTGCAGGATACAATATGGGTTCAAAATCATTTTCTCACCGTGCAAACTCT<br>ATGCATGAACAATGTCAAAGTAGAAGTTATTTTTTGTCTCAATGGGTATCGGATGATGGGTCA<br>ACAAGTGAATTTAAAGCTAGAAGATTTAAAATTGAAAAATCTACCTCAAATGACTAATATTTGG<br>GAGGCTACCAAGAACACATTTACCCTCCAACATCTCACGTTATTAGAAATAATGAGATGTGAA<br>AAATTGGAAGTAATATTTCCTCAATCTATGCTGAGATGCCTACCAGAATTGAA
Chr08	62,477,326	C	T	T|T	C|C	TA=intergenic_variant		TTGTCGGATGGTGTAGATAACATTAGCTTGAGTAGAGATGTTACAATTCACAAGTCAAGTA<br>GTGCATATATACTTCCACAAGATTCCCAGGTTGTTAAACAAGATGATAAGATGAATGAAGA<br>CGAGGCAGGAATAACACCATATAAAAATATCAAAATCCAAGATGAGGTGAACATGTTGGAT<br>AAAACAGAGGGAATAGGAATTGTTTCTGATAATGGAGTTGAGGTTACTCCAGGTATGGCT<br>CTTTTCTTCTCTATTTTTCATATTTTATTTTTATGTCACCTACTTCTCAAACTTTTTACAACTT<br>ATACAGTTACTCGTACAAGATTGGAAGAGTATAAACACTTTGTTGATCTCGATGATTCACAA<br>ATTTCTCTGCTAGTGGAGGCAATAACAGCATATCCTCATCTTTGGAATGCTTGTGAGAAGT<br>TTAGGGATCGCTTCCAAGCATGGATGTTGAAAACATTGGCAGATATGTTGTTGTTTCTTCG<br>AAGTGAAAG	TTGATAGTGTTAATCCTCATAAAGAAAAGGAGTTCCATAAACTATGTGATGAAGTTATTCAACT<br>TGGATTTGAGAGGTTGTGGGTTGATGATATGCGCCAACGTGTTGTGGCAAGGAATCCTAAGT<br>TGGACCATGCAAAGGCACGGATTGGTGAGCTTATTAAGACACACGACCATTTAACTCAACAAT<br>TAGATAACATAAAGAAAGAACTTAGGAGTCTTAATGACTTTGTTGATGCTCAAACAAAATATTT<br>CGACTTTTTGTGATACGGAAGTCGCAGACAAACACTTTTCTATTTCTTCTTACAAATACATTTG<br>GCATTATTTGTGATTCTTCTAAAAATGAGTGTCACTTTCTCAATGTGATGTTATTTTCTGAATGT<br>AATGTTATTTGAGCTGGTAGAGCAAGATGTTTAAGTTTATTATACTTCATTTACCGTAGACATT<br>ATATCGTTTTTTGAATTTCTTCAAGCATGGTTTGATGTCTAATTTCTCCCTTAT
Chr08	62,485,641	G	T	G|G	T|T	TA=intron_variant	TGN=Phvul.008G284800	GAACATAGTGTTGTCTAATCAAGTTGGAAATTTGGTGGATGATGTTCAAGCTTGTGCTTGC<br>ATTATTTCATTTAATTCTGAATCTCATCTTCTCCCTCTTCTTGTCACAAGTTATCTGGTATTT<br>ATATTGAATTTTCAGTTTTTGCCTTGTTCCATTCTTCAGTTTGACACTCGCAGTTTCTAAGAT<br>CATTTCAAAATTGAGGGGTTTAATATTCTTTCAAGAACATAGTAGATTACATTGAAAGTTTA<br>CTTACCCTTTTGTGCAGCACACCATAGGTGTAAATCTTCGTTTGATGACTTCCCACTCCAA<br>AGCTATATTCATCTTTCTATGATCATTGGATTTTTGGACTCTTTTGTTGCAACTGGGTTTTG<br>GAAACATAGCATCTGAAACTAGTTTGCCAGTTTATTATTTTTATCTTGATTATGCCCTTAGT<br>CCATGTTCCTCTATCTGTGATTTATCCTTGTTGTCAGTTACTCCGTTAAGTATGTTATTAAG<br>CCCA	GCAAGGTATCATCTCGTAATTCTCCTTTGATGTGTGTATCAAGGTCCCAAGATTGCGGTATTA<br>TTTTACTCATGTATTGTGTATGGTTCTAGCTATGTGTTGTCAAGTGTTTAATACAAGCTTATTCA<br>GCACTATATAGGTCTAGACGCTTTAACAGAGTTGATGCACAGTTAATCAATGACTGATATTATC<br>TAGTCTGGAATGCTTCCTCATATTTTCGTTTAATTCATTTATGTTGCTAACTGTATGATGAATTG<br>TGCAAATTACACAAGAGTTATGTTGACAAGGATCCAAGCTTGGTGAATTAACCTTTCAATTTAC<br>TTCTTCTTGGTGAATATAAATGTTGTTGCTGCTATACAAGCATATCGTACAGTGCATCTCCATG<br>TAACAGCTGCATTCTTGCTTATCTCGTGTGTTTTATCAGGATGTTGGCTTGTGGCTGGAGACT<br>ATAAATCTTGGTAGTTACCGCCAAATTTTTAAAGAAAATGGTGTTAATGGAG
Chr08	62,492,823	C	T	T/T	C/C	TA=intron_variant	TGN=Phvul.008G284900	AGTTGTTACATGATGTGGATGTGTAACTCCATATATGCTTAATTTATCTGCCAACCTGCTTT<br>ATAAAATAAAATTAACCAGCTGCCTCTTTAGATCTATTTGTTACAGATTGTGCTGATGGTAA<br>AATAATCAAACTTTTCAGGATGACTTTGTGCAGAGTATATCATGAGTAATAGTAGAGTGTTC<br>TTTCACTAATAGCTTTTTGACTTTTGTAAATTGTGTGAAATGCATATGAATATAATCTCAATT<br>ATTATCATGCTCCTTTGACTCTTCATCATGTTTGAATAAAATCAAAGGCATGAAGTTTGTGC<br>TGGGACTAAATGATTAGAGGTTTAATTTCCTTATTTTTTAACCTCACGTGTGAGTTTTAAGA<br>AAAAAGTAAGCTTGATGATAGAGGGAAGTAGTAAATGAAATCCTAAAATAAATTTGGTTGTT<br>GACTAGATGACTTTTGTAGTTTAGATTTTAGGTATGCACATGGATTCTCAGTTCATGTTGTT<br>TGA	TCTTAGGGTGGGATTGTATGTGAAGGCACATTTTATTTATGATTTTGTAGTTGATGAATCCTGG<br>CAATCTTTTTAGTTGATGAATAGATTAAGGCAGTGAATAAATAATTATAGGTTCAGGTAAAATG<br>CAAGATAAAAACAGCACATTGACAACAAGAAGTGGATATTCCATGACTGTATGATGCTTTTTT<br>GATTGCCAGGACATGCCTTACAAATTATTTTAGAGCATGGTGAAAAGTTATCTGCAATGATTC<br>AGCTGAGGGAGCTTCAAAATATGATCAGCCAAAATCGATCAACTAATGTTGACTCCTCAAAAT<br>CTAGTTCTGACATTCAGATGTCAGGTGCCCTTTGGGATATGATACAGTTAGTTGGTGAGAGAG<br>CTCGTCGAAACACTGTCCTTCTGATGGATAGAGACAACGCTGAAGTGTTCTACAGTAAGGTTT<br>CAGATCTTGAAGATCTCTTCTACTGCTTAGATAAAGAGTTAGAGTATGTAATAAGAC

Chr08	62,515,805	T	C	T|T	C|C	TA=intron_variant	TGN=Phvul.008G285300*	ATCCAAGCTAATGTTATCTACACCACTTGACAAAGTTTTAGTAACAACTCCCTCTAATGGAGACCCTTCTTCGATCTTTTGTCGTCCTATTTCCTCAATATTCTGTACATCACGAATGAAAAATGAATGTTTGCGTATCTTTTAAGTTATAGCTTTGAGAAGCATTTGTTTCATAACATACATCATGTGTTAGTACAACAATAATCTGAGCAATTTTAGTGGTTACCATTATTTCTTCGTGTGCATGTTGGTGTGGAAGACCTGTTTCATTGTTTGATTCTTTGTGCACCAACTTTTATTTGAAATATTGAACAAGCATAACAAAAATTAGTGTTTATCGAAGGAGATTTGTTTACAATCCTAGTCATTTTTAAATTTATAAGCAAATGTATTTTATGCAAGAGAGTTACCATACAAGTGTTTGGGATGGACTTCTCACCTTAAAAACATAAGTTGGTGTTATTTTAATTTCTTCCATCATGTAGTTTACTAATGTATAA	TTTTAGAAGATTAAAATAAAATATATAAATACCTCAATCATGTTTGAAGAAGAAATGATTGGCAAATTTTGCTCAATCGCTTGTATGTCTGATATCTTTTCACTTGTTTTGTCTATTTTGTTCAACTTTTCTGATTCAGATACAAGTGCTTTGGTTTCTATTTTTTTATGTTCATGTAAGATTTCAGCTGTGCTTCCCAACATAGATTCAGTGTTATCATTAACTGGTGAAGAACATGGTTTATTTTCAGATGTAGGTGGATCTTCTTGGTTCATTTGAAGAGGAGAAGTGTTTTCTGACATATAACGTTTAAGCTGAAAATAATTAATTGTAAACAAGTTGAACTATGTTCAAAAGGAGAATTGAACACAACACTTAAACATAAACATGCATATATCTTTTCACTTAAACTAATAATTAAAATAATAAGAATTACCTTGCGAGTTGTGCTACTATTATCAAGTTGTGAGCATCCATCGATATAAAATCTCTTCAAAGAT
Chr08	62,515,994	C	T	C/C	T/T	TA=missense_variant	TGN=Phvul.008G285300*	TTAGTACAACAATAATCTGAGCAATTTTAGTGGTTACCATTATTTCTTCGTGTGCATGTTGGTGTGGAAGACCTGTTTCATTGTTTGATTCTTTGTGCACCAACTTTTATTTGAAATATTGAACAAGCATAACAAAAATTAGTGTTTATCGAAGGAGATTTGTTTACAATCCTAGTCATTTTTAAATTTATAAGCAAATGTATTTTATGCAAGAGAGTTACCATACAAGTGTTTGGGATGGACTTCTCACCTTAAAAACATAAGTTGGTGTTATTTTAATTTCTTCCATCATGTAGTTTACTAATGTATAATTTTTTAGAAGATTAAAATAAAATATATAAATACCTCAATCATGTTTGAAGAAGAAATGATTGGCAAATTTTGCTCAATCGCTTGTATGTCTGATATCTTTTCACTTGTTTTGTCTATTTTGTTCAACTTTTCTGATTCAGATACAAGTGCTTTGGTTTCTATTTTTTTATGTTCATGTAAGATTTCAG	GTGCTTCCCAACATAGATTCAGTGTTATCATTAACTGGTGAAGAACATGGTTTATTTTCAGATGTAGGTGGATCTTCTTGGTTCATTTGAAGAGGAGAAGTGTTTTCTGACATATAACGTTTAAGCTGAAAATAATTAATTGTAAACAAGTTGAACTATGTTCAAAAGGAGAATTGAACACAACACTTAAACATAAACATGCATATATCTTTTCACTTAAACTAATAATTAAAATAATAAGAATTACCTTGCGAGTTGTGCTACTATTATCAAGTTGTGAGCATCCATCGATATAAAATCTCTTCAAAGATGGCCATGCCTTGGACAGAGAGTTGGTAGCACAAATACTAATAATGTTTGGTAGCTTATCAAGATAGAGTTCTTCCAATGTTGGAAAATGAAGATGAATCTCCTTATCATTTTTATTATCAAGATGGCATTGGCCAATCATATATTTCAATTGGTCACAATTGTACACTGAAAGAGTTCTTAAGTTTGGA
Chr08	62,518,268	T	C	T|T	C|C	TA=missense_variant	TGN=Phvul.008G285300	TTTAGTTTACTTTATTCATTAAATAATATATAAAACTAAAAGAAATAAAAAAATGCTTAGTATTTTAGGGACTAAAATGAAATTGTCTTAATTGTATACACTAAAATATAAAATTTTGTAACAACAAAGATGAAATGAGTAAAAAATACCTGCGTTACGGTTTACACTGGTTGGTGATGGAATTGAAGTGGTTTTGAAGTGGTTAGATTCAAAATGTTGATGCTTATCTTCCTCACTAATTTCATCTCTTTCTTCTTTTTCATGAATAACATCCCCAATTATTTGTTCTAGCATATCACATTTGTTTATATAAAGCATTTTCAATTGAGACAGATCTCCTATGGTACTCACTGAGAATACATGATTTAATTTTCCACAATCATCTATCAACACTTCTTGAAGATTTTGGAAAATTTTGGATGGCAGGGAACACCCAACTGCATAGTCACCTGGAACAATGACATATGTCAACTCTGGACAATTTCTTAAACATAGTTTCT	AATTTTTCAAAAGACCCACTGCCAGGTACACAACCATGCCATAAAGCTCTTAGATATTTCATCTCCATGATGAACAGGGTATGCAACTTGGAGAAGAGATTTCTCACCTTATTCAAATCATTGCTAGTGTCAACCAAATACTCAATCTCTTCAGAATTCATTATTCTCAAATCATTCAACTCATTCATACCCCCTTCAATTTCAAATATATCAGGCATCATATTTTTTACATCTCCTTCAATATTTTCTATAAAGAGATCCTTTGCTTCTTTTGCCAAACCCTTAATAACCTCATTTGATATGTTAAAATTATGAAGCACTAAAGTTCTTTGACAAGAGATAAATTCTGATGATATTGAAATATGAGGAAATTGTTGTCCTAATAGAATTCCATACCTTTGTAGTGTTTGGACAAAACTAAACAAGTTAAAGCATTCAATGAACTTTCTATCATTGTACTTTGATTTTGTTTGAATGATATACAATTCTTCTAAAAGTGG
Chr08	62,518,337	T	C	T/T	C/C	TA=missense_variant	TGN=Phvul.008G285300*	GACTAAAATGAAATTGTCTTAATTGTATACACTAAAATATAAAATTTTGTAACAACAAAGATGAAATGAGTAAAAAATACCTGCGTTACGGTTTACACTGGTTGGTGATGGAATTGAAGTGGTTTTGAAGTGGTTAGATTCAAAATGTTGATGCTTATCTTCCTCACTAATTTCATCTCTTTCTTCTTTTTCATGAATAACATCCCCAATTATTTGTTCTAGCATATCACATTTGTTTATATAAAGCATTTTCAATTGAGACAGATCTCCTATGGTACTCACTGAGAATACATGATTTAATTTTCCACAATCATCTATCAACACTTCTTGAAGATTTTGGAAAATTTTGGATGGCAGGGAACACCCAACTGCATAGTCACCTGGAACAATGACATATGTCAACTCTGGACAATTTCTTAAACATAGTTTCTTTAATTTTTCAAAAGACCCACTGCCAGGTACACAACCATGCCATAAAGCTCTTAGATATTTCATCTCCA	ATGAACAGGGTATGCAACTTGGAGAAGAGATTTCTCACCTTATTCAAATCATTGCTAGTGTCAACCAAATACTCAATCTCTTCAGAATTCATTATTCTCAAATCATTCAACTCATTCATACCCCCTTCAATTTCAAATATATCAGGCATCATATTTTTTACATCTCCTTCAATATTTTCTATAAAGAGATCCTTTGCTTCTTTTGCCAAACCCTTAATAACCTCATTTGATATGTTAAAATTATGAAGCACTAAAGTTCTTTGACAAGAGATAAATTCTGATGATATTGAAATATGAGGAAATTGTTGTCCTAATAGAATTCCATACCTTTGTAGTGTTTGGACAAAACTAAACAAGTTAAAGCATTCAATGAACTTTCTATCATTGTACTTTGATTTTGTTTGAATGATATACAATTCTTCTAAAAGTGGGATGCTTTTGATCTCTTCAAGATTCTTCACTTCAATGTCACATTCATTAAACTCTAACAATTTCAAGTT
Chr08	62,518,346	G	A	G/G	A/A	TA=synonymous_variant	TGN=Phvul.008G285300*	GAAATTGTCTTAATTGTATACACTAAAATATAAAATTTTGTAACAACAAAGATGAAATGAGTAAAAAATACCTGCGTTACGGTTTACACTGGTTGGTGATGGAATTGAAGTGGTTTTGAAGTGGTTAGATTCAAAATGTTGATGCTTATCTTCCTCACTAATTTCATCTCTTTCTTCTTTTTCATGAATAACATCCCCAATTATTTGTTCTAGCATATCACATTTGTTTATATAAAGCATTTTCAATTGAGACAGATCTCCTATGGTACTCACTGAGAATACATGATTTAATTTTCCACAATCATCTATCAACACTTCTTGAAGATTTTGGAAAATTTTGGATGGCAGGGAACACCCAACTGCATAGTCACCTGGAACAATGACATATGTCAACTCTGGACAATTTCTTAAACATAGTTTCTTTAATTTTTCAAAAGACCCACTGCCAGGTACACAACCATGCCATAAAGCTCTTAGATATTTCATCTCCATGATGAACA	GTATGCAACTTGGAGAAGAGATTTCTCACCTTATTCAAATCATTGCTAGTGTCAACCAAATACTCAATCTCTTCAGAATTCATTATTCTCAAATCATTCAACTCATTCATACCCCCTTCAATTTCAAATATATCAGGCATCATATTTTTTACATCTCCTTCAATATTTTCTATAAAGAGATCCTTTGCTTCTTTTGCCAAACCCTTAATAACCTCATTTGATATGTTAAAATTATGAAGCACTAAAGTTCTTTGACAAGAGATAAATTCTGATGATATTGAAATATGAGGAAATTGTTGTCCTAATAGAATTCCATACCTTTGTAGTGTTTGGACAAAACTAAACAAGTTAAAGCATTCAATGAACTTTCTATCATTGTACTTTGATTTTGTTTGAATGATATACAATTCTTCTAAAAGTGGGATGCTTTTGATCTCTTCAAGATTCTTCACTTCAATGTCACATTCATTAAACTCTAACAATTTCAAGTTTTTAAGTTG
Chr08	62,518,616	C	A	C/C	A/A	TA=stop_gained	TGN=Phvul.008G285300*	CACTGAGAATACATGATTTAATTTTCCACAATCATCTATCAACACTTCTTGAAGATTTTGGAAAATTTTGGATGGCAGGGAACACCCAACTGCATAGTCACCTGGAACAATGACATATGTCAACTCTGGACAATTTCTTAAACATAGTTTCTTTAATTTTTCAAAAGACCCACTGCCAGGTACACAACCATGCCATAAAGCTCTTAGATATTTCATCTCCATGATGAACAGGGTATGCAACTTGGAGAAGAGATTTCTCACCTTATTCAAATCATTGCTAGTGTCAACCAAATACTCAATCTCTTCAGAATTCATTATTCTCAAATCATTCAACTCATTCATACCCCCTTCAATTTCAAATATATCAGGCATCATATTTTTTACATCTCCTTCAATATTTTCTATAAAGAGATCCTTTGCTTCTTTTGCCAAACCCTTAATAACCTCATTTGATATGTTAAAATTATGAAGCACTAAAGTTCTTTGACAAGAGATAAATT	GATGATATTGAAATATGAGGAAATTGTTGTCCTAATAGAATTCCATACCTTTGTAGTGTTTGGACAAAACTAAACAAGTTAAAGCATTCAATGAACTTTCTATCATTGTACTTTGATTTTGTTTGAATGATATACAATTCTTCTAAAAGTGGGATGCTTTTGATCTCTTCAAGATTCTTCACTTCAATGTCACATTCATTAAACTCTAACAATTTCAAGTTTTTAAGTTGTCTAAATGCAACATCAGTTTGCAAATCAATGAATGAAGGCCATGAACAACCATGCAATGAAAGACTTTGAAGTTTCTTCATATCTCTTACAAATGAGATGTCACTCAATTTCCAGTATTGAAGGACTAGACAACGAAGATTTCTTAATGTTTTGAAAGACCTTGTTGACAACCGCAATGCATAATCTCTATTTGGATTGGTAATAATCAAAACTCTAAGTTTTCCCATTCTTTCAAAAATTTCATCGGATACTTCCAATTTTGCTTTTAT
Chr08	62,531,605	C	T	T/T	C/C	TA=intron_variant	TGN=Phvul.008G285500	AACTCACCAAATAAGAAATGAGACCCATTAAATAGGACCTGCATAAATTTTGCTCAACTGAAGAGAGAATGTCAAAAGAAAAATGTTAAAAATTGTATTGCTAGTATTTATCATTACGTAATATTGGCAATTTGGGCTATACCATCTCATCCATATGACACACTGCAATTCATTGGCAAGTGACAAAAATGATGGTATGGATGATGGTTGAATGAGTAGTGTGCATGATGGTATACAGAACACCCAATTTGATTGAAGCTACTCTTGAGATGATGATAAATAAACCCAGCATATGGAACGTGCACTCCAATGTTATATCACATATAGATTCCCTGCAGGGCACACAAATTTTCAAATTTAGTAAAGGGTGATGATGCAGAAGACATAAGGGTAAGTTACTCAATTGAATGGCTAAAAGTCACAGTGGAAGCAGAGATGCATTTCACACGCATAAGGGTAAGGCATATCAATTGAAACTGGTGATAAAATTCGGTGACATG	GACTCAAATTTTTCGATGCTCAGACACCTATGCTGATGAGATTGAAAACCAACAAAGTAATAATCTTAAAAAGTGAAACATAGTATTTCAATATGAAACAAAAAAGAAGGAACAACTTCCTATGAAAAGAACAAAGCAGGGTGTTCCACAGAATTTATCAAGAGAAGGAATACCATTTGAATGTGGACACCCTGATTGGGTCAAAAGATACCTTCTGCTATTATATTATTGGCTGTTGATACAGTATAGCATGGCTTTTATGGTACCTACGATGAACTTGAAAGTGTAGGTGAGTGAGATACATTTCAGACTACAGAATACATCCATCTTAAAGTGAATTTTAGGCATAGTAAGGAGATATAGGAAGAAAAAAGAGAATTCAATGAGAGCAATGCTTTTAATAAATATGTTTCTTTTTTATTCAATTAATAATGCATAAAAATAGGAAGCTATATTTTTCCTGAACATAATACAGTAAATGGCTTCCAAATTAACAGGTA
Chr08	62,549,638	C	A	C/C	A/A	TA=intron_variant	TGN=Phvul.008G285800	GGGTATCAACTATCCTCTTCTGATTCTTTTCTCCGCAAGGTTTTTCTAGCATCACTTTATGTTGTAGAATCGGTTGATTTCTAAAAGTGACGGGTGCCCTCTTCCTAGATTTTTAAAACAAAACAGTAGTTGTTGCTGATATGAATATGAACAGTAAACAAAGTGATTGTCTTTATCCTCCATCTTACCTGTCTATATTGCAGATTTTGTTCAACTGTATTGATGAAAGAAGACTTCGGATGCTGTTAAGGAGTGGCATGGTCAGCACTCCACAGTTAATGTGTCCAGAGAATGAGGAGTCAAAAAAGGAGCGTATATGTGTAGCAGACACTGTAGAACCTATCGAACAATGTCCAATTCTCAATGATTATGTCCAGGCAAGACTTGAATTATCAGTCTCATTCAAAAATTATGTATCATTGGCCTCGGGCACTGCACTTTCGACTGATTTGGTTAGTTATTTCCTCCGTGGAAAGGTAGAATCTTTATTAAAGATAAAA	TGTATTTGTTTTGCTCAATAGTCCAATAATTTAAATTGTTGGTTCTCCTAAAAATTTGTTAGTATCTAAGAATTATAGTCTAGCTTACAATTCCCTTTCCTCACTGCTCAACCTATCCTAGGGGGCATAATATGGTGGTAGTTGATTGAATGGAGATAAAAGGGCATAGTGACGAACTTTAGGATTCCTAGCTTTCCTTTTCTGTGAGAATGGTGAGTTTGTGGAAGCTAGGGGAGAAATGCAACTACTAATAATGCATCATCTGATTCTACAGTGGTGCCATTTTGAATGCACTTGAATTGAATGTAATCAATAGAATCCCTTAAATTGCCTTCAGAATCATAACATTCTTTTTAGTTTGGCTATGCCATTAATAAATCTTGTCTTGGTGTGTAAAAGATATGACATTACCAGTGATAGTCTAGCATTTTAAATCATTCAACCTACAACCTAACATTATTATATAGTCATATTGATGGAAAACTAGTGGATTTTGGGT
Chr08	62,556,407	G	A	G/G	A/A	TA=intron_variant	TGN=Phvul.008G285800	TCCCTTGGCACTGAAAAAAATGAAGTAAATAGGAAGAAAGTATCCAAACTTGGAAGTTTATTAATGAACAAACAAATTTTGGTGGTTGATGATAATGCAGTGAACAGAAGAGTTGCAAAAGGTTTTCTACAGAAATACGAGGCAAAAGTTACTTCTGTGGAGAGTGGCATGGCTGCTCTGGAGGTGCTTAAACTGCCACATAATTTTGATGCTTGTTTTATGGATCTCCAAATGCCAGAAATGAATGGGTAATTTATCTTCTTATTCCTATTTTTTAAGTTTTCTGAATTTTTATTGCAAATTAAGACACTGTTGTCACCTATAGGAATAGAAATAAGGAGCAATGACTGGTCTAGTAAGTAGATAATAATCAGTAGAACTGGTGCCAAAAGTCAGGCCCCTATGCTTATTTTCTTTTGCTAAAGAACTTACTACCTATAATATTTTAACCGCAACTGATTTTTTCTGGGGGTCTACTACTCAATCTATTATAATTTAGA	TTAGGGTTTAAGGTTTCTGTGTCTATATAAATAGATATTAAGATTATTTCCATGCCTTGTAATTAATTTCATTAGAATATATAAAATATGTTATCACATTTTAGGTTATCCTCTGGGATTAGTTTCACAGTTTTGCCAGAGGCTCGGTTACTGTATTTAAGGGAGTTGATAAATCCTGCAATGTGCATGCTGTTATTATGTTATCCTTAGGGGTGACATATGACCAGTTAACTTATTTTAGAAATCTCAGACTCTGAATGCTTTTCTAGACATGATCTCACACACCTGGATCCATGACTGAGATCTAATTTAAAAGTATTTTTCTTTATTTGAAATGGTACAGTTTTGAAGCAACAAGGGAAATCCGCCGACTGGAGAGCGAGGTAAATGAGAAAATTGCGTGTGGGCAAGCATCTGCAGAGATGTATGGTAACATCTCTTATTGGCACATTCCAGTACTAGCAATGACAGCAGATGCAACTCAGAGTTCAAGTGAAGAA
Chr08	62,565,315	T	C	C/C	T/T	TA=intergenic_variant		ATGCTGTAGTTTGGATGAGTAATTGATTTGTTAAATTTAACATTTCAAAAGTTGTGTTGGATATAATTCTAAATTCCAAAAACCTGTCCCTTATTATTATTTGAACTCTTCAACAGTTAAAAATGCACTGAACCTTGGTGAAATGAGTAGAAGATGAAACATGGCATTATTAGACAACTTTAATCTTTGGATTAGAATTGTACTACTTTATGATTAAAGTACATTACATGGATTAAAAGGATAGTTGAACTGTTTTACTTTTTTTATCTTTTTAACCAATGTTGTAAGCAACAAAATTAAGTCACTGTTAACAAGATGAAAAAGTTAAAATTATGATAGTATTTAAAACCTTCATCCTTTTTGAACTCAGAGTTCCACCACCACATGAATGAAACAGATTATTATGTGTTCTAAATTTAGAATGAATGGTAAGAAAATTAGAGATGAAGATGATATGTATGAATTTTGTTAAATAATTTTGTTTGCTTTATTTTTGTATG	TTACTAAACCCTTATTTTAGGAAGAAAGATGTTGTGTGTTTTGCAGATTGATTGTAGGCAGTCCGAGTATCTGGATGGAGTAAAGAGATTCATTGCAATAGGAGCATCAGCTCCTTACATTAGATTAATATGACTCTTTGGAGGTGTATTTGTATCATACAAATATTATATTTTTTTTGTTTGTGTTCGTGATTTGTTAAGGTATTGTGTTTTTTCTATAAGGGATTAAAAAAGAAAAGATCTTTTATGGTTGGAGGCTATAGTTTGGTGAACTACTTTATGTATTTTGAAGTTTGATGTGATGACATACCCTCGTTGCTGTCAAGGACGAACCTCTTTAGTTGTTTTTTGGAGATAAGAATTTGTTGGTAATTTCTCTTGAAAGTTTGGGATGAACTGCAGAGTGAGGTTCTACATTATTTTTATATATAATTCTGTATAAAGATTAAGTTACTCATTAAATCATTCATATTTTTTTTTCTGATAAACAAAACTTTGAG

Chr08	62,619,023	G	A	G/G	A/A	TA=intergenic_variant		CATTCGTCATTGTGTTTACTGAGTTTTGGGTGAGATTTGAGCCCCAGACTCGTCCCACAAGATTCGCAATAAATTTTTTATTCATCAGTGTCCGGGCTGAAAGGAAGGTTCGCTTGTGTGTGAAACTGTTTGATTTATTTCCTGGGTGAATACTCATCCGTTTGACCTGAAATCTGTCGCGTTTTGTCCCAAACTCAGTGGAGATTCTTACGCGGAATTTCAATAAAAAACTATTTCGGGAGCTTTTTTCAGAAATTTTTTGCAAACCAAGGCTATCCTCTAGCAAAACTTGTGAAATTTCGCCCTACTTTCTGAAAAAAAAAAAAATTTCGCGTCCGTATTGCGCTGAAAAAATTCGCAAAAGTACTTTCGTATGTTTTTTGCACCCAGGTGAGGAGGCAGGGCCGTAAACAAATCACAGAAAGATGATACGGGGAAGAAAAGCCAGGACGTTTTGTTTTCGGAAAATCGGTTTTGTTCACTCTCGGTCTGGGACTTACT	GCCTTACCCCTTGGGTATTTTGGTCTGAAATTTTTACCAGACATTCTTCATTGTGTCTACTGAGTTTTGGCTGAGATTTGAGCCCCAGACTCGTCCTACAAGATTCGCAATAAATCTTTTATTCGTCAGTGTCCGGGCTGAAAGGAAGGTTCGCTTGTGTGTGATGCTGTTTGATTTTTTTCCTGGGTGAATACTCATCCGTTTGACCTGAAATCTGTCGCGTTTTGTCCAAAACTCAGTGAAGATTCTTACGCGGAATTTCAGGAAAAAACCATTTCGGGAGATTTTTTCAGAAATTTTGTGCAAACCAAGGCAATCCTTCAGCAAAACTTGTGAAATTTCGCCCTACTTTTTGAAGAAAAATTTTTGCGTCCGTATTGCGCTGAAAAAATTCGCACAAGTACTTTCTTATGTTGTTTCCACCCAGGTGAGGAGGCACAGCCATAAACAAATCACAGAAAGAAGATACGGGGAAGAAAAGTCAGGACGTTTTGTTTTCG
Chr08	62,621,935	C	A	C/C	A/A	TA=intergenic_variant		CTTACGCTGAATTTCAGGAAAAAACTATTTCTGAAGATTTTTTCAGAAATTTTGTGCAAATCAAGGCTATCCTCTAGCAAAACTTGTGAAATTTCGCCCTACTTTCTGAAAAAAAAAAAATTTGCGTCCGTATTGCGCTGAAAAAATTCGCACAAGTACTTTCGTATGTTGTTTGCACCAAGGTGAGGAGGCACGGCCATAAACAAATCACAGAAAGAAGATACGGGGAAGAAAAGCCAGGACGTTTTGTTTTCGGAAAACCGGTTTTGTTCACTCTCGGTCTGGGACTTACTGCGCCTTTCCCCTTTGGTATTTTGGTCTGAAATTTTTACCAGACATTCGTCATTGTGTCTAATGAGTTTTGCCTGAGATTTGAGCCCAAGACTCGTCCCAGAGGATTCGCAATAAATTTGTTATTCATCAGTGTCCGGGCTGAAAGGAGATTCGCTTGTGTGTGAAACTGTTTGATTTTTTTCCTGGGTGAATACTCATCCGTTTGAC	GAAATCTGTCGCGTTTTGTCCCAAACTCAGTGGAGATTCTTACGCGGAATTTTAGGAAAAAACCATTTCGGGAGATTTTTTCTGAAATTTTGTGCAAACCAAGGTTATCCTCTAGCAAAACTTGTGAAATTTCGCCCTACTTTCTGAAAAAAAAAAATTCGCTTCCGTATTGCGCTGAAAAAATTCACACAAGTACTTTCGTATGTTGTCTGCATCCAGGTGAGGAGGCACGGCCATAAACAAATAACAGAAAGAAGATACGGGAAAGAAAAGCCAGGACGTTTTGTTTTCGGAAAATCGGTTTTGTTCACTCTCGGTCTGGGACTTACTAGGGTTTACCCCTTGGGTAATTTGCTCTGAAATTTTTACCAGACATTCGTCATTGTGTCTACTGAGTTTTGGCTGAGATTTGAGCCCCAGACACGTCCCACAAGGTCCGCAATAAATTTTTTTTTCATCAGTGTCCGGGCTGAAAGGAGATTCACTTGTGTGTGAAACTGT
Chr08	62,641,938	C	T	T/T	C/C	TA=intron_variant	TGN=Phvul.008G287100	GCTAGAATTATCAATTATCTGAACTTCACATAAATGCCCATTAGGTCATTGCTGCACTGACAGTTCTTCCTAATTGATAAAAAATGATACTCCAATTGTGTCATGGGTTTTCCATTTTAAATTATATGCTTTAAAAGAGGTTCGGCATCTAAGGTATGGGCCTTTAAAACACCAGTATATTTGTTGACCAATTAAATTGAAACAGTCTTCCTTCTGAACTCTTAATCAGTTCTGCTTTAAGACAATTTCAAACTCTAGGCTACTTTCTGGAATACCCACAAGATCACGTGGGTTGTCTTGATTCTCCAAGCATCCTCTTAAACAATGTTTTCACTTTGAAGCATGTAATCCATACATGTAATCTTCAAGCATAAATAACACCTAATCTAATACTGCTGTGCTTCTTGGTGTGATTGATAGCTATTGTATTTGGTTTGCGTACCAATTGACTCAGGATAAAAGCTAAAAAGCAGTAAGAGAGGGCCCAGATATCATCTGAC	TAGAATTACAAAGTTGTAACTCTGGAAAACCTTGAAGTACGAAATAATATTTGTTCTCACCAAAAAAAAAAAACTCTGCAAAAACTACATGAAAATTCATTAGGATGTGGTTGGTCCCGTTCCTGTATCATCACTACTTAAAATTGATGGAAATTGTTTTATTACTGTATTTCTTTTCAATAGCAGAAAATGATTGTATTTAATCCTCTTAATTTCAGATAAGTGTATCATGATAACTTATTTTATATCTTGTGCAGCAAAACATGCTGAGATATGGAGTATAGCAACTTCCATGGAGGACCATAAATCGGAAATTGAAAACTGGGAGTTGGGAGCTGGAATTTATATTTCATTTTATTTGATGAGAAACTCACTTCAGGGTGATACCAATTCCATGACCAAATTGGTATGTGAATATCATCCCCATCATCATTTATGTGAATAATGGTTGTTACTTGATTCTGTCATAGTTTGATTGTAAAGTGTAACTAAAAGTTAAT
Chr08	62,660,069	C	A	A/A	C/C	TA=synonymous_variant	TGN=Phvul.008G287400	TCACAAATAAATCGGTCCAAAATGGTCAAGTTTGCTATGACAAAAGGTTGGCATCATAATGTCATCTTAGAAGAACTAAATGCACCAGGAATCAAATTCTTGATGCTGAAGCTCCCACAATAGCAACAGCACATTGAAAATTTTCCTCTGAAATGCTATGACAGCAGATTTGATCATATGCTTTCACTGATGATAACAGAGGCTACAAGGAAACTATATAAATAACATAAAAGTTCATATATTCAGATAACAACATCTTTCATCATCGTTAAGATATTCAGATTGTGATTTTTTTTATATGATTGCCCAACTGCAGTCACAATCTAGAATAGCAGCAATAACACAAAAGGGCCTAATTGAGAGTTCCATATGGTATGCAAACTTTTAAATCCAATAATTGACTTGTAATCTAGGTAACTAATCTCACCTGGCACTGCTCTACCATACATAGCTGCTAAAGCCACATATAAATCTTCGGGATCTTTGGGCTGAAAATCAAA	CCAGAACCTGAAAAGGCATTATCATTTTTGTGACTAGCTTAGAACTCAAGCTTGAACAAAATGGCAGTCATTGGTGATATTAAATTACTGAAACAAAAAGAAGCCAATGTTTCTATTCCCAATAAGCAGTAAATCAAATTAAACAAACTACAAAAGACTGGTTTTGTACCGGTTGGTCCGTACGACTGAGTGAAAGCCCGAAAGCCCAATACTTTGACCGAAACCATCACATCCAAAAAGGAATAATGAGATTAATAATGATCAAGCACTAGATAACACATTCTTAACCACGAACCATATCTTTAATCCGGCCCAATAACAAAGGACTCATGATATCATATAAATAAACAGAGATTCCAAGAATCAGGTAGATCATCATTCAGTAATAATAGAATCGAGTCTCAAACACCAAAGTCTTACTTGAATGTCGGAACGTCTTTTGCAGTTACTATCCGAATCCGGATATCACGAAGACAAGTAGTAGAGGAGCGAGAAGAGTA

*disease resistant genes.
